# Supplementary material for: Bayesian Inference of State-Level COVID-19 Basic Reproduction Numbers across the United States
Source: Viruses. 2022 Jan 15;14(1):157. doi: 10.3390/v14010157 (PMC8780010; doi:10.3390/v14010157)
Supplement: Supplementary file 1 [file viruses-14-00157-s001.zip › viruses-1512103-supplementary.pdf]

Supplementary Information: Bayesian Inference of State-Level COVID-19 Basic Reproduction Numbers across the United States

Abhishek Mallela[a], Jacob Neumann[b,1], Ely F. Miller [b], Ye Chen[c], Richard G. Posner[b], Yen Ting Lin[d], and William S. Hlavacek[e,2*]

[a]Department of Mathematics, University of California, Davis, CA 95616, USA

[b]Department of Biological Sciences, Northern Arizona University, Flagstaff, AZ 86011, USA

[c]Department of Mathematics and Statistics, Northern Arizona University, Flagstaff, AZ 86011, USA

[d]Computer, Computational and Statistical Sciences Division, Los Alamos National Laboratory, Los Alamos, NM 87545, USA

[e]Theoretical Division, Los Alamos National Laboratory, Los Alamos, NM 87545, USA

[1]Current address: Department of Chemistry and Chemical Biology, Cornell University, Ithaca, NY 14853, USA

[2]To whom correspondence may be addressed. Email: wish@lanl.gov

Reduced Model

We derive $\mathcal{R}_0$ from a simplified form of the compartmental model of Lin et al. [1]. The reduced model is obtained by omitting variables and terms for interventions, including social distancing, quarantine, and self-isolation. Thus, the reduced model describes disease transmission dynamics in the absence of interventions. The equations of the reduced model are as follows:

$$\frac{dS}{dt} = -\frac{\beta S}{S_0}[I + \rho_E(E_2 + E_3 + E_4 + E_5) + \rho_A A], \quad (1)$$

$$\frac{dE_1}{dt} = \frac{\beta S}{S_0}[I + \rho_E(E_2 + E_3 + E_4 + E_5) + \rho_A A] - k_L E_1, \quad (2)$$

$$\frac{dE_i}{dt} = k_L(E_{i-1} - E_i), \text{ for } i = 2,3, \dots, m \quad (3)$$

$$\frac{dA}{dt} = f_A k_L E_m - c_A A, \quad (4)$$

$$\frac{dI}{dt} = (1 - f_A) k_L E_m - c_I I, \quad (5)$$

$$\frac{dH}{dt} = f_H c_I I - c_H H, \quad (6)$$

$$\frac{dR}{dt} = c_A A + (1 - f_H) c_I I + f_R c_H H, \quad (7)$$

$$\frac{dD}{dt} = (1 - f_R) c_H H, \quad (8)$$

where t denotes time, β, S_0, ρ_E, ρ_A, k_L, f_A, f_H, f_R, c_A, c_I, and c_H are positive-valued time-invariant parameters, as defined in Lin et al. [1], and m denotes the number of stages taken to comprise the incubation period. Here and in the study of Lin et al. [1], $m = 5$. The values of β (a rate constant characterizing disease transmission) and S_0 (the total population) are taken to be region-specific; the other parameters have values that are taken to be universal (i.e., applicable to all regions of interest). The variable S denotes the population of susceptible persons. The variables E_1 to E_m denote populations of exposed persons, e.g., persons incubating virus but not symptomatic. As noted earlier, the incubation period is divided into m stages. The variable A denotes the population of persons who have progressed through the incubation period but will never develop symptoms (i.e., persons with asymptomatic

infections). The variable I denotes the population of persons with mild symptomatic disease. The variable H denotes the population of persons with severe disease who are hospitalized or isolated at home. The variable R denotes the population of recovered persons, and the variable D denotes the population of deceased persons.

Basic Reproduction Number

The basic reproduction number, $\mathcal{R}_0$, is defined as the number of secondary infections caused by an infected person during the entire period of infectiousness when introduced into a population consisting of susceptible persons only and there are no interventions to limit disease transmission. Here, we use the next-generation matrix method to compute $\mathcal{R}_0$ [2]. The model has a disease-free equilibrium (DFE) x_0 with $S = S_0$, where S_0 is the total population and the remaining populations $(E_1, E_2, \dots, E_m, A, I, H, R, D)$ are equal to 0.

To use the next-generation matrix method, we let $x = (E_1, E_2, E_3, E_4, E_5, A, I)$ denote the vector of state variables corresponding to compartments containing infected persons. For each infected compartment i, we define f_i as the rate of entry of newly infected persons into compartment i and v_i as the net transfer of persons out of the ith compartment. Then, we have $dx_i/dt = f_i(x) - v_i(x)$. Now, we let F and V denote the Jacobians of f and v evaluated at the disease-free equilibrium x_0. The (i, j) entry of the matrix F is the rate at which infected persons in the jth compartment produce a new infection in the ith compartment. The (j, k) entry of the matrix V^{-1} is the expected amount of time that a person introduced to the kth compartment will spend in a single visit to the jth compartment. The matrix F, which is non-negative, is defined as follows:

$$F \equiv \begin{pmatrix} 0 & \rho_E\beta & \rho_E\beta & \rho_E\beta & \rho_E\beta & \rho_A\beta & \beta \\ 0 & 0 & 0 & 0 & 0 & 0 & 0 \\ 0 & 0 & 0 & 0 & 0 & 0 & 0 \\ 0 & 0 & 0 & 0 & 0 & 0 & 0 \\ 0 & 0 & 0 & 0 & 0 & 0 & 0 \\ 0 & 0 & 0 & 0 & 0 & 0 & 0 \\ 0 & 0 & 0 & 0 & 0 & 0 & 0 \end{pmatrix}$$

The matrix V, which is non-singular (i.e., invertible), is defined as follows:

$$V \equiv \begin{pmatrix} k_L & 0 & 0 & 0 & 0 & 0 & 0 \\ -k_L & k_L & 0 & 0 & 0 & 0 & 0 \\ 0 & -k_L & k_L & 0 & 0 & 0 & 0 \\ 0 & 0 & -k_L & k_L & 0 & 0 & 0 \\ 0 & 0 & 0 & -k_L & k_L & 0 & 0 \\ 0 & 0 & 0 & 0 & -f_A k_L & c_A & 0 \\ 0 & 0 & 0 & 0 & -(1-f_A)k_L & 0 & c_I \end{pmatrix}$$

We find $\mathcal{R}_0$ as the spectral radius (i.e., the dominant eigenvalue) of the matrix FV^{-1} [2], which is given by Eq. 1 in the main text.

Epidemic growth rate

The epidemic growth rate λ is defined as the dominant eigenvalue of the Jacobian of the reduced model linearized at the disease-free equilibrium (DFE). Thus, λ is the largest root of the characteristic polynomial for the 7-dimensional Jacobian matrix J, which is equivalent to $F - V$. We used the *CharacteristicPolynomial* function in Mathematica [3] to find J:

$$p_J(x) = \beta(1-f_A)k_L^5(c_A + x) + [\beta f_A k_L^5 \rho_A + (c_A + x)(-k_L^5 + 4\beta k_L^4 \rho_E - 5k_L^4 x + 6\beta k_L^3 \rho_E x - 10k_L^3 x^2 + 4\beta k_L^2 \rho_E x^2 - 10k_L^2 x^3 + \beta k_L \rho_E x^3 - 5k_L x^4 - x^5)](c_I + x) \quad (9)$$

The largest root was found numerically. Solutions were based on state-specific estimates for β and the estimates of Lin et al. [1] for other parameters in Eq. 9.

Progress toward herd immunity

In this section, we explain the assumptions and derive the formula for our metric of progress toward herd immunity (Eq. 2 in the main text). First, we define the variables used in our analysis. For a

given region, S_0 denotes the total population size, N_d denotes the cumulative number of cases detected, N_a denotes the cumulative number of asymptomatic cases, N_v denotes the cumulative number of vaccinations completed, $N_{v,s}$ denotes the number of persons who were susceptible at the time of vaccination, $N_{v,r}$ denotes the number of persons who had recovered from infection at the time of vaccination, N_c denotes the cumulative number of all cases, ε_v denotes the fraction of vaccinated individuals protected from productive infection (i.e., an infection that can be transmitted to others), ε_r denotes the fraction of recovered individuals protected from productive infection, N_r denotes the number of individuals who have recovered from infection, HIT denotes the herd immunity threshold for ancestral strains, Y_{Delta} denotes the infectiousness of SARS-CoV-2 variant Delta relative to ancestral strains, $S_h \equiv \text{HIT} \times S_0$ denotes the threshold number of persons with immunity needed for herd immunity (in the face of ancestral strains), S_i denotes the estimated number of persons with immunity, $f_A \equiv N_a/N_c$ denotes the fraction of all cases that are asymptomatic, $f_r \equiv N_r/S_0$ denotes the fraction of the population with immunity acquired through infection, and $f_v \equiv N_v/S_0$ denotes the fraction of the population that has been vaccinated.

We assume that S_0 is constant. We take $N_r = N_c$ to be a good approximation. We assume that we know S_0, N_d, and N_v. We assume that susceptible and recovered individuals have the same probability of being vaccinated. From our assumption that susceptible and recovered individuals have the same probability of being vaccinated, it follows that $N_{v,s} = (1 - f_r)N_v$ and $N_{v,r} = f_r N_v$. These relations are consistent with $N_v \equiv N_{v,s} + N_{v,r}$. The number of individuals with immunity (protection from productive infection) is given by

$$S_i = \varepsilon_{\text{v}} N_{v,s} + \varepsilon_r N_r = \varepsilon_{\text{v}}(1 - f_r)N_v + \varepsilon_r N_r \tag{10}$$

We assume that Y_{Delta} gives the value of β for SARS-CoV-2 variant Delta relative to β for ancestral strains. We assume all other model parameters are the same for Delta. Thus, $Y_{\text{Delta}}\mathcal{R}_0$ is the basic

reproduction number in the face of Delta. We define $\mathcal{P}$, percent progress toward herd immunity, as

$$\mathcal{P} = \frac{S_i}{S_0}\left(1 - \frac{1}{Y_{\text{Delta}}\mathcal{R}_0}\right)^{-1} \times 100\%$$

Using the expression given above for S_i (Eq. 10), $1 - 1/(Y_{\text{Delta}}\mathcal{R}_0)$ as the Delta-adjusted HIT, and $S_h = \text{HIT} \times S_0$, we find Eq. 2 in the main text.

References

1. Y. T. Lin *et al.*, Daily forecasting of regional epidemics of coronavirus disease with Bayesian uncertainty quantification. *Emerg Infect Dis*. **27**, 767-778 (2021).
2. O. Diekmann, J. A. Heesterbeek, M. G. Roberts, The construction of next-generation matrices for compartmental epidemic models. *J R Soc Interface*. **7**, 873-875 (2010).
3. Wolfram S., *Mathematica: A System for Doing Mathematics by Computer* (Addison Wesley Longman Publishing Co., Inc., Boston, MA, 1991).
4. E. D. Mitra *et al.*, PyBioNetFit and the biological property specification language. *iScience*. **19**, 1012-1036 (2019).

Table S1. Estimates of state-specific model parameters. MAP estimates and 95% credible intervals (within parentheses) are given for the inferred parameters.

State	S_0	I_0	m_b	\rho_E	\rho_A	k_L	k_Q	j_Q	f_A	f_H	f_R	c_A	c_I	c_H	t_0	tau_1-sigma	beta	lambda_0	p_0	lambda_1	p_1	f_D	r
Alabama	4903185	1	0.1	1.1	0.9	0.94	0.0038	0.4	0.44	0.054	0.79	0.26	0.12	0.17	30.984(22.360-34.336)	N/A	0.391(0.347-0.436)	3.499(1.001-9.868)	0.413(0.369-0.452)	N/A	N/A	0.967(0.239-1.000)	7.552(5.428-9.683)
Alaska	731545	1	0.1	1.1	0.9	0.94	0.0038	0.4	0.44	0.054	0.79	0.26	0.12	0.17	0.389(0.000-8.320)	24.592(20.416-31.857)	0.209(0.208-0.226)	8.181(1.843-9.917)	0.424(0.388-0.545)	0.252(0.153-9.449)	0.042(0.008-0.102)	0.946(0.538-1.000)	3.105(1.884-4.686)
Arizona	7278717	1	0.1	1.1	0.9	0.94	0.0038	0.4	0.44	0.054	0.79	0.26	0.12	0.17	34.393(31.555-40.781)	61.085(57.441-63.526)	0.544(0.453-0.595)	6.487(0.816-6.945)	0.518(0.469-0.552)	5.772(0.660-9.581)	0.422(0.375-0.476)	0.136(0.039-0.931)	10.369(7.094-12.786)
Arkansas	3017804	1	0.1	1.1	0.9	0.94	0.0038	0.4	0.44	0.054	0.79	0.26	0.12	0.17	17.086(0.673-27.769)	N/A	0.283(0.241-0.350)	7.148(1.130-9.832)	0.276(0.210-0.369)	N/A	N/A	0.977(0.244-0.992)	2.493(1.777-3.268)
California	39512223	1	0.1	1.1	0.9	0.94	0.0038	0.4	0.44	0.054	0.79	0.26	0.12	0.17	18.929(12.922-20.468)	N/A	0.387(0.363-0.410)	0.091(0.079-0.106)	0.552(0.529-0.570)	N/A	N/A	0.955(0.304-1.000)	13.758(9.699-18.231)
Colorado	5758736	1	0.1	1.1	0.9	0.94	0.0038	0.4	0.44	0.054	0.79	0.26	0.12	0.17	11.114(4.946-18.122)	N/A	0.373(0.331-0.417)	0.164(0.093-0.261)	0.486(0.435-0.555)	N/A	N/A	0.047(0.029-0.156)	12.605(9.102-16.019)
Connecticut	3565287	1	0.1	1.1	0.9	0.94	0.0038	0.4	0.44	0.054	0.79	0.26	0.12	0.17	24.579(19.192-34.957)	N/A	0.458(0.366-0.529)	0.163(0.067-0.256)	0.537(0.487-0.694)	N/A	N/A	0.069(0.049-0.910)	3.867(2.720-4.822)
Delaware	973764	1	0.1	1.1	0.9	0.94	0.0038	0.4	0.44	0.054	0.79	0.26	0.12	0.17	15.707(1.352-25.519)	N/A	0.276(0.247-0.315)	0.063(0.037-0.125)	0.513(0.377-0.569)	N/A	N/A	0.539(0.052-0.935)	3.984(2.789-5.207)
Florida	21477737	1	0.1	1.1	0.9	0.94	0.0038	0.4	0.44	0.054	0.79	0.26	0.12	0.17	33.729(26.441-34.913)	63.884(63.829-72.644)	0.551(0.478-0.590)	0.169(0.130-0.202)	0.674(0.637-0.685)	0.044(0.003-8.701)	0.548(0.380-0.505)	0.889(0.158-0.999)	10.547(7.282-13.066)
Georgia	10617423	1	0.1	1.1	0.9	0.94	0.0038	0.4	0.44	0.054	0.79	0.26	0.12	0.17	30.334(24.584-33.012)	N/A	0.448(0.398-0.500)	0.176(0.134-0.235)	0.586(0.554-0.613)	N/A	N/A	0.960(0.303-1.000)	6.577(4.880-8.300)
Hawaii	1415872	1	0.1	1.1	0.9	0.94	0.0038	0.4	0.44	0.054	0.79	0.26	0.12	0.17	0.250(0.005-18.577)	43.824(40.640-50.707)	0.243(0.227-0.275)	8.435(1.200-9.819)	0.462(0.429-0.531)	0.368(0.208-9.438)	0.065(0.000-0.133)	0.269(0.148-0.989)	7.416(3.538-12.138)
Idaho	1787065	1	0.1	1.1	0.9	0.94	0.0038	0.4	0.44	0.054	0.79	0.26	0.12	0.17	30.384(22.035-41.502)	26.118(16.787-44.416)	0.402(0.321-0.491)	1.650(0.579-9.408)	0.556(0.451-0.647)	0.128(0.002-8.144)	0.524(0.327-0.561)	0.206(0.063-0.967)	3.660(2.494-4.612)
Illinois	12671821	1	0.1	1.1	0.9	0.94	0.0038	0.4	0.44	0.054	0.79	0.26	0.12	0.17	23.804(20.353-27.454)	N/A	0.511(0.462-0.574)	0.271(0.186-0.404)	0.506(0.476-0.539)	N/A	N/A	0.047(0.043-0.051)	16.940(11.817-20.504)
Indiana	6733219	1	0.1	1.1	0.9	0.94	0.0038	0.4	0.44	0.054	0.79	0.26	0.12	0.17	30.947(28.069-34.415)	N/A	0.534(0.482-0.599)	0.273(0.185-0.383)	0.582(0.553-0.620)	N/A	N/A	0.061(0.043-0.097)	23.028(16.373-27.360)
Iowa	3155070	1	0.1	1.1	0.9	0.94	0.0038	0.4	0.44	0.054	0.79	0.26	0.12	0.17	20.864(4.957-26.906)	N/A	0.284(0.265-0.311)	0.051(0.031-0.077)	0.513(0.400-0.559)	N/A	N/A	0.914(0.070-0.976)	6.652(4.727-8.375)
Kansas	2913314	1	0.1	1.1	0.9	0.94	0.0038	0.4	0.44	0.054	0.79	0.26	0.12	0.17	9.116(0.040-21.513)	N/A	0.327(0.294-0.393)	8.963(0.715-9.752)	0.288(0.236-0.394)	N/A	N/A	0.020(0.016-0.031)	2.446(1.811-3.124)
Kentucky	4467673	1	0.1	1.1	0.9	0.94	0.0038	0.4	0.44	0.054	0.79	0.26	0.12	0.17	14.497(23.657-31.344)	N/A	0.330(0.336-0.371)	0.247(1.525-6.814)	0.426(0.430-0.476)	N/A	N/A	0.065(0.409-0.809)	2.832(2.534-2.852)
Louisiana	4648794	1	0.1	1.1	0.9	0.94	0.0038	0.4	0.44	0.054	0.79	0.26	0.12	0.17	29.321(23.099-34.600)	23.063(22.072-41.478)	0.487(0.396-0.561)	0.068(0.050-0.143)	0.991(0.724-0.999)	0.044(0.009-9.149)	0.621(0.428-0.647)	0.449(0.166-0.996)	3.851(2.476-4.454)
Maine	1344212	1	0.1	1.1	0.9	0.94	0.0038	0.4	0.44	0.054	0.79	0.26	0.12	0.17	0.056(0.001-7.602)	N/A	0.226(0.225-0.244)	9.273(2.139-9.983)	0.232(0.226-0.271)	N/A	N/A	0.982(0.519-1.000)	6.302(4.256-8.785)
Maryland	6045680	1	0.1	1.1	0.9	0.94	0.0038	0.4	0.44	0.054	0.79	0.26	0.12	0.17	20.912(16.191-25.682)	N/A	0.409(0.369-0.455)	0.180(0.110-0.286)	0.464(0.420-0.502)	N/A	N/A	0.057(0.047-0.071)	13.997(10.360-18.506)
Massachusetts	6892503	1	0.1	1.1	0.9	0.94	0.0038	0.4	0.44	0.054	0.79	0.26	0.12	0.17	22.670(11.016-25.519)	N/A	0.380(0.348-0.420)	0.055(0.042-0.096)	0.680(0.481-0.706)	N/A	N/A	0.946(0.059-0.914)	4.847(3.404-5.928)
Michigan	9986857	1	0.1	1.1	0.9	0.94	0.0038	0.4	0.44	0.054	0.79	0.26	0.12	0.17	36.349(29.724-39.519)	N/A	0.599(0.506-0.754)	0.219(0.154-0.357)	0.685(0.641-0.721)	N/A	N/A	0.952(0.105-0.968)	2.915(2.098-3.639)
Minnesota	5639632	1	0.1	1.1	0.9	0.94	0.0038	0.4	0.44	0.054	0.79	0.26	0.12	0.17	0.105(0.000-3.412)	N/A	0.300(0.298-0.311)	9.571(2.160-10.000)	0.212(0.204-0.238)	N/A	N/A	0.023(0.021-0.026)	8.521(6.229-10.348)
Mississippi	2976149	1	0.1	1.1	0.9	0.94	0.0038	0.4	0.44	0.054	0.79	0.26	0.12	0.17	27.049(17.259-40.172)	N/A	0.475(0.350-0.645)	9.133(0.804-9.688)	0.450(0.367-0.583)	N/A	N/A	0.048(0.033-0.269)	2.518(1.782-3.260)
Missouri	6137428	1	0.1	1.1	0.9	0.94	0.0038	0.4	0.44	0.054	0.79	0.26	0.12	0.17	37.235(30.639-41.290)	N/A	0.475(0.406-0.581)	0.937(0.298-7.115)	0.523(0.479-0.566)	N/A	N/A	0.985(0.228-0.996)	8.290(5.755-10.350)
Montana	1068778	1	0.1	1.1	0.9	0.94	0.0038	0.4	0.44	0.054	0.79	0.26	0.12	0.17	0.754(0.002-16.742)	32.524(27.801-36.190)	0.220(0.220-0.262)	9.528(1.637-9.996)	0.495(0.476-0.612)	5.366(0.234-9.455)	0.056(0.033-0.157)	0.971(0.257-1.000)	4.025(1.952-5.840)
Nebraska	1934408	1	0.1	1.1	0.9	0.94	0.0038	0.4	0.44	0.054	0.79	0.26	0.12	0.17	0.036(0.000-14.804)	N/A	0.246(0.236-0.256)	0.020(0.011-0.032)	0.628(0.464-0.938)	N/A	N/A	0.180(0.088-0.917)	6.131(4.385-7.965)
Nevada	3080156	1	0.1	1.1	0.9	0.94	0.0038	0.4	0.44	0.054	0.79	0.26	0.12	0.17	26.225(17.540-32.919)	65.190(58.061-72.778)	0.356(0.331-0.437)	0.508(0.237-9.075)	0.452(0.408-0.493)	6.065(0.219-9.464)	0.320(0.267-0.418)	0.802(0.093-0.981)	8.104(5.467-10.151)
New Hampshire	1359711	1	0.1	1.1	0.9	0.94	0.0038	0.4	0.44	0.054	0.79	0.26	0.12	0.17	0.002(0.000-3.737)	N/A	0.287(0.284-0.298)	9.176(1.949-9.968)	0.267(0.255-0.294)	N/A	N/A	0.026(0.021-0.033)	10.142(6.935-12.710)
New Jersey	8882190	1	0.1	1.1	0.9	0.94	0.0038	0.4	0.44	0.054	0.79	0.26	0.12	0.17	32.076(30.841-36.593)	N/A	0.652(0.592-0.706)	0.143(0.113-0.165)	0.710(0.686-0.768)	N/A	N/A	0.173(0.102-0.917)	16.197(11.032-20.226)
New Mexico	2096829	1	0.1	1.1	0.9	0.94	0.0038	0.4	0.44	0.054	0.79	0.26	0.12	0.17	2.728(0.179-20.746)	N/A	0.280(0.248-0.302)	0.173(0.057-0.273)	0.342(0.325-0.451)	N/A	N/A	0.052(0.032-0.910)	10.695(7.485-13.903)
New York	19453561	1	0.1	1.1	0.9	0.94	0.0038	0.4	0.44	0.054	0.79	0.26	0.12	0.17	26.493(22.339-28.209)	6.945(5.447-8.628)	0.537(0.503-0.580)	3.341(0.002-9.310)	0.000(0.000-0.047)	0.173(0.141-0.236)	0.693(0.649-0.718)	0.842(0.266-1.000)	15.497(10.271-17.131)
North Carolina	10488084	1	0.1	1.1	0.9	0.94	0.0038	0.4	0.44	0.054	0.79	0.26	0.12	0.17	31.206(25.599-36.723)	N/A	0.408(0.381-0.512)	0.430(0.239-4.668)	0.460(0.417-0.498)	N/A	N/A	0.916(0.178-0.996)	9.592(6.512-11.786)
North Dakota	762062	1	0.1	1.1	0.9	0.94	0.0038	0.4	0.44	0.054	0.79	0.26	0.12	0.17	0.121(0.000-6.987)	N/A	0.211(0.211-0.225)	0.044(0.025-0.089)	0.389(0.316-0.484)	N/A	N/A	0.987(0.504-1.000)	6.428(4.278-8.309)
Ohio	11689100	1	0.1	1.1	0.9	0.94	0.0038	0.4	0.44	0.054	0.79	0.26	0.12	0.17	24.363(15.560-29.743)	N/A	0.374(0.340-0.412)	0.114(0.091-0.147)	0.558(0.520-0.586)	N/A	N/A	0.521(0.110-0.990)	13.676(9.929-17.656)
Oklahoma	3956971	1	0.1	1.1	0.9	0.94	0.0038	0.4	0.44	0.054	0.79	0.26	0.12	0.17	31.752(22.607-36.912)	69.291(66.405-72.574)	0.368(0.329-0.437)	0.655(0.398-5.037)	0.455(0.420-0.680)	0.675(0.197-9.220)	0.230(0.016-0.302)	0.854(0.148-0.992)	7.919(5.391-10.034)
Oregon	4217737	1	0.1	1.1	0.9	0.94	0.0038	0.4	0.44	0.054	0.79	0.26	0.12	0.17	16.689(8.407-26.374)	62.379(58.500-66.910)	0.312(0.283-0.342)	0.680(0.342-9.302)	0.400(0.343-0.416)	1.426(0.348-9.533)	0.239(0.179-0.295)	0.302(0.114-0.997)	10.100(6.422-13.594)
Pennsylvania	12801989	1	0.1	1.1	0.9	0.94	0.0038	0.4	0.44	0.054	0.79	0.26	0.12	0.17	33.882(28.338-35.164)	N/A	0.499(0.470-0.529)	0.119(0.108-0.135)	0.682(0.657-0.694)	N/A	N/A	0.897(0.148-0.980)	24.128(17.365-30.657)
Rhode Island	1059361	1	0.1	1.1	0.9	0.94	0.0038	0.4	0.44	0.054	0.79	0.26	0.12	0.17	22.434(5.055-30.168)	N/A	0.307(0.268-0.356)	0.059(0.024-0.187)	0.595(0.300-0.672)	N/A	N/A	0.616(0.050-0.925)	1.722(1.190-2.274)
South Carolina	5148714	1	0.1	1.1	0.9	0.94	0.0038	0.4	0.44	0.054	0.79	0.26	0.12	0.17	27.886(20.463-34.331)	53.614(50.857-61.892)	0.377(0.345-0.447)	0.301(0.182-1.122)	0.497(0.450-0.531)	0.327(0.000-9.392)	0.370(0.256-0.423)	0.578(0.080-0.985)	10.621(7.582-13.534)
South Dakota	884659	1	0.1	1.1	0.9	0.94	0.0038	0.4	0.44	0.054	0.79	0.26	0.12	0.17	33.934(21.169-39.697)	N/A	0.311(0.284-0.363)	0.244(0.124-1.721)	0.444(0.375-0.473)	N/A	N/A	0.943(0.096-0.963)	4.223(2.790-5.426)
Tennessee	6829174	1	0.1	1.1	0.9	0.94	0.0038	0.4	0.44	0.054	0.79	0.26	0.12	0.17	31.625(23.354-37.080)	N/A	0.435(0.375-0.575)	0.334(0.151-5.156)	0.519(0.468-0.560)	N/A	N/A	0.996(0.118-0.969)	7.464(5.170-9.169)
Texas	28995881	1	0.1	1.1	0.9	0.94	0.0038	0.4	0.44	0.054	0.79	0.26	0.12	0.17	24.420(21.333-29.131)	61.271(60.063-80.583)	0.415(0.386-0.445)	0.123(0.101-0.167)	0.578(0.542-0.596)	0.083(0.000-9.067)	0.486(0.262-0.504)	0.337(0.131-0.963)	12.400(7.972-15.013)
Utah	3205958	1	0.1	1.1	0.9	0.94	0.0038	0.4	0.44	0.054	0.79	0.26	0.12	0.17	15.334(3.259-26.542)	56.229(49.991-62.279)	0.323(0.280-0.368)	0.421(0.161-2.055)	0.396(0.329-0.428)	0.008(0.000-8.346)	0.727(0.167-0.670)	0.210(0.018-0.897)	13.792(9.650-16.554)
Vermont	623989	1	0.1	1.1	0.9	0.94	0.0038	0.4	0.44	0.054	0.79	0.26	0.12	0.17	5.435(2.059-32.206)	43.446(35.682-49.688)	0.317(0.245-0.357)	9.312(0.546-9.589)	0.437(0.408-0.564)	4.301(0.224-9.482)	0.172(0.170-0.363)	0.011(0.012-0.938)	2.375(1.397-2.952)
Virginia	8535519	1	0.1	1.1	0.9	0.94	0.0038	0.4	0.44	0.054	0.79	0.26	0.12	0.17	11.702(4.752-20.999)	N/A	0.353(0.313-0.411)	0.178(0.060-0.480)	0.393(0.335-0.479)	N/A	N/A	0.036(0.027-0.066)	5.054(3.505-6.291)
Washington	7614893	1	0.1	1.1	0.9	0.94	0.0038	0.4	0.44	0.054	0.79	0.26	0.12	0.17	15.968(5.728-20.448)	N/A	0.368(0.315-0.428)	0.157(0.110-0.242)	0.527(0.477-0.569)	N/A	N/A	0.982(0.264-1.000)	4.574(3.333-5.664)
West Virginia	1792147	1	0.1	1.1	0.9	0.94	0.0038	0.4	0.44	0.054	0.79	0.26	0.12	0.17	6.096(0.001-19.932)	N/A	0.230(0.221-0.269)	8.721(1.382-9.895)	0.251(0.233-0.324)	N/A	N/A	0.932(0.231-1.000)	4.054(2.716-5.348)
Wisconsin	5822534	1	0.1	1.1	0.9	0.94	0.0038	0.4	0.44	0.054	0.79	0.26	0.12	0.17	19.169(8.024-29.143)	N/A	0.416(0.328-0.486)	8.494(0.409-9.195)	0.411(0.337-0.487)	N/A	N/A	0.046(0.031-0.164)	5.939(4.220-7.690)
Wyoming	578759	1	0.1	1.1	0.9	0.94	0.0038	0.4	0.44	0.054	0.79	0.26	0.12	0.17	0.192(0.000-10.292)	N/A	0.209(0.208-0.227)	9.361(1.886-9.972)	0.213(0.203-0.259)	N/A	N/A	0.984(0.517-1.000)	2.564(1.670-3.464)

Table S2. State-specific MAP estimates and 95% credible intervals (within parentheses) of the initial epidemic growth rate λ (/d), basic reproduction number $\mathcal{R}_0$, herd immunity threshold (HIT), and Delta-adjusted HIT for SARS-CoV-2 ancestral strains. Estimates are based on state-specific daily reports of new COVID-19 cases from January 21, 2020 to June 21, 2020 (inclusive dates).

State	λ (/d)	$\mathcal{R}_0$	HIT	Delta-adjusted HIT*
Alabama	0.278(0.245-0.310)	4.250(3.770-4.735)	0.765(0.735-0.789)	0.904(0.892-0.914)
Alaska	0.127(0.126-0.142)	2.275(2.257-2.452)	0.560(0.557-0.592)	0.821(0.820-0.834)
Arizona	0.382(0.322-0.414)	5.910(4.921-6.463)	0.831(0.797-0.845)	0.931(0.917-0.937)
Arkansas	0.193(0.157-0.247)	3.078(2.622-3.800)	0.675(0.619-0.737)	0.868(0.845-0.893)
California	0.275(0.257-0.292)	4.204(3.948-4.456)	0.762(0.747-0.776)	0.903(0.897-0.909)
Colorado	0.264(0.232-0.297)	4.049(3.601-4.536)	0.753(0.722-0.780)	0.900(0.887-0.910)
Connecticut	0.325(0.260-0.373)	4.977(3.982-5.752)	0.799(0.749-0.826)	0.918(0.898-0.929)
Delaware	0.187(0.162-0.220)	2.996(2.687-3.425)	0.666(0.628-0.708)	0.864(0.849-0.881)
Florida	0.387(0.339-0.411)	5.994(5.195-6.413)	0.833(0.808-0.844)	0.932(0.922-0.937)
Georgia	0.319(0.283-0.354)	4.872(4.324-5.438)	0.795(0.769-0.816)	0.917(0.906-0.925)
Hawaii	0.158(0.144-0.186)	2.638(2.471-2.988)	0.621(0.595-0.665)	0.846(0.835-0.864)
Idaho	0.286(0.224-0.348)	4.374(3.488-5.338)	0.771(0.713-0.813)	0.907(0.883-0.924)
Illinois	0.361(0.328-0.401)	5.557(5.017-6.242)	0.820(0.801-0.840)	0.927(0.919-0.935)
Indiana	0.376(0.342-0.416)	5.808(5.241-6.512)	0.828(0.809-0.846)	0.930(0.922-0.938)
Iowa	0.194(0.178-0.216)	3.088(2.884-3.376)	0.676(0.653-0.704)	0.868(0.859-0.880)
Kansas	0.229(0.203-0.279)	3.557(3.201-4.269)	0.719(0.688-0.766)	0.886(0.873-0.905)
Kentucky	0.232(0.236-0.263)	3.588(3.648-4.031)	0.721(0.726-0.752)	0.887(0.889-0.899)
Louisiana	0.345(0.282-0.393)	5.294(4.303-6.093)	0.811(0.768-0.836)	0.923(0.906-0.933)
Maine	0.143(0.142-0.159)	2.454(2.450-2.653)	0.593(0.592-0.623)	0.834(0.834-0.847)
Maryland	0.291(0.261-0.324)	4.446(4.006-4.951)	0.775(0.750-0.798)	0.909(0.899-0.918)
Massachusetts	0.270(0.246-0.299)	4.127(3.783-4.567)	0.758(0.736-0.781)	0.901(0.893-0.911)
Michigan	0.416(0.358-0.505)	6.510(5.498-8.194)	0.846(0.818-0.878)	0.938(0.926-0.950)
Minnesota	0.207(0.205-0.217)	3.261(3.237-3.386)	0.693(0.691-0.705)	0.875(0.874-0.880)
Mississippi	0.337(0.247-0.444)	5.167(3.804-7.012)	0.806(0.737-0.857)	0.921(0.893-0.942)
Missouri	0.337(0.289-0.405)	5.169(4.411-6.316)	0.807(0.773-0.842)	0.921(0.908-0.936)
Montana	0.137(0.137-0.175)	2.390(2.392-2.852)	0.582(0.582-0.649)	0.830(0.830-0.857)
Nebraska	0.161(0.152-0.170)	2.671(2.565-2.788)	0.626(0.610-0.641)	0.848(0.841-0.854)
Nevada	0.252(0.232-0.311)	3.870(3.595-4.750)	0.742(0.722-0.789)	0.895(0.887-0.914)
New Hampshire	0.196(0.194-0.206)	3.117(3.092-3.238)	0.679(0.677-0.691)	0.870(0.869-0.874)
New Jersey	0.448(0.412-0.479)	7.093(6.440-7.673)	0.859(0.845-0.870)	0.943(0.937-0.947)
New Mexico	0.190(0.162-0.209)	3.042(2.692-3.280)	0.671(0.629-0.695)	0.866(0.849-0.876)
New York	0.378(0.356-0.405)	5.842(5.469-6.310)	0.829(0.817-0.842)	0.930(0.926-0.936)
North Carolina	0.290(0.270-0.362)	4.433(4.141-5.567)	0.774(0.758-0.820)	0.908(0.902-0.927)
North Dakota	0.129(0.128-0.142)	2.296(2.289-2.443)	0.564(0.563-0.591)	0.823(0.822-0.834)
Ohio	0.265(0.239-0.293)	4.063(3.698-4.475)	0.754(0.730-0.777)	0.900(0.890-0.909)
Oklahoma	0.261(0.231-0.311)	3.998(3.579-4.748)	0.750(0.721-0.789)	0.898(0.886-0.914)

Oregon	0.217(0.193-0.241)	3.389(3.078-3.721)	0.705(0.675-0.731)	0.880(0.868-0.891)
Pennsylvania	0.353(0.334-0.373)	5.423(5.108-5.753)	0.816(0.804-0.826)	0.925(0.920-0.929)
Rhode Island	0.213(0.180-0.251)	3.338(2.911-3.865)	0.700(0.656-0.741)	0.878(0.860-0.895)
South Carolina	0.268(0.244-0.318)	4.102(3.755-4.862)	0.756(0.734-0.794)	0.901(0.892-0.916)
South Dakota	0.216(0.194-0.257)	3.377(3.091-3.947)	0.704(0.677-0.747)	0.880(0.868-0.897)
Tennessee	0.310(0.266-0.401)	4.730(4.079-6.247)	0.789(0.755-0.840)	0.914(0.900-0.935)
Texas	0.295(0.274-0.317)	4.509(4.197-4.841)	0.778(0.762-0.793)	0.910(0.903-0.916)
Utah	0.226(0.191-0.261)	3.510(3.046-4.004)	0.715(0.672-0.750)	0.884(0.867-0.898)
Vermont	0.221(0.160-0.253)	3.446(2.665-3.884)	0.710(0.625-0.743)	0.882(0.847-0.895)
Virginia	0.250(0.218-0.293)	3.839(3.404-4.469)	0.739(0.706-0.776)	0.894(0.881-0.909)
Washington	0.261(0.220-0.304)	4.004(3.428-4.648)	0.750(0.708-0.785)	0.898(0.881-0.913)
West Virginia	0.146(0.138-0.181)	2.496(2.406-2.927)	0.599(0.584-0.658)	0.837(0.831-0.861)
Wisconsin	0.296(0.230-0.344)	4.527(3.570-5.278)	0.779(0.720-0.811)	0.910(0.886-0.923)
Wyoming	0.127(0.126-0.144)	2.272(2.260-2.467)	0.560(0.558-0.595)	0.821(0.820-0.835)

*Based on Delta being 2.46 times more infectious than ancestral strains.

Table S3. State-specific MAP estimates of β obtained using different datasets. Each 4-month dataset consists of state-specific daily case counts from January 21, 2020 to May 21, 2020. Each 5-month dataset consists of state-specific daily case counts from January 21, 2020 to June 21, 2020. Each 6-month dataset consists of state-specific daily case counts from January 21, 2020 to July 21, 2020.

	β		
State	4-month dataset	5-month dataset	6-month dataset
Alabama	0.344425688	0.391000116	0.381114062
Alaska	0.207653693	0.209267205	0.210030786
Arizona	0.529445483	0.543664926	0.532514897
Arkansas	0.32298932	0.28311954	0.279105356
California	0.329080014	0.386768663	0.366219921
Colorado	0.381094706	0.372505137	0.377135113
Connecticut	0.449485806	0.457835438	0.442979285
Delaware	0.280036868	0.27564221	0.284739604
Florida	0.526921359	0.551352675	0.514304171
Georgia	0.430531087	0.448214949	0.438936107
Hawaii	0.23746958	0.242697953	0.247395063
Idaho	0.380490164	0.402373556	0.36290268
Illinois	0.498497271	0.511225388	0.50775665
Indiana	0.541857154	0.534310158	0.534529103
Iowa	0.311378197	0.284051758	0.271908187
Kansas	0.300249291	0.327199897	0.244883859
Kentucky	0.346224636	0.330082083	0.331292779
Louisiana	0.488353117	0.487004484	0.504124433
Maine	0.225236222	0.225787998	0.230958926
Maryland	0.398259593	0.409012941	0.410424365
Massachusetts	0.386526299	0.379636348	0.387649785
Michigan	0.602060282	0.59887731	0.608052362
Minnesota	0.263631203	0.299947476	0.255634342
Mississippi	0.446758703	0.475310237	0.505863769
Missouri	0.444622556	0.475476797	0.439740679
Montana	0.220783058	0.219834578	0.219902109
Nebraska	0.244391787	0.2457406	0.244258417
Nevada	0.359673276	0.35601223	0.361269375
New Hampshire	0.273648163	0.286714521	0.284787414
New Jersey	0.656582201	0.652491027	0.676182424
New Mexico	0.289726267	0.279879781	0.275387246
New York	0.535623994	0.537427785	0.675754416
North Carolina	0.409013369	0.407806714	0.416748691
North Dakota	0.213348899	0.211192131	0.211282563

Ohio	0.407742398	0.373799884	0.396832606
Oklahoma	0.367641453	0.367742577	0.368838695
Oregon	0.30011376	0.311724057	0.305668351
Pennsylvania	0.497075654	0.49886789	0.498607161
Rhode Island	0.319663632	0.307048589	0.317887767
South Carolina	0.382005702	0.377319773	0.378889298
South Dakota	0.315190829	0.310612853	0.311311794
Tennessee	0.451102391	0.435139557	0.422615302
Texas	0.418736232	0.414805718	0.429223299
Utah	0.31236819	0.32286498	0.307458597
Vermont	0.265564455	0.317029863	0.303010374
Virginia	0.347873875	0.353117278	0.335591603
Washington	0.351809052	0.368329925	0.353636894
West Virginia	0.238034407	0.229590329	0.235258658
Wisconsin	0.386164126	0.416429043	0.373319249
Wyoming	0.210764739	0.209001836	0.219153861

Table S4. Sensitivity of MAP estimate of β to training dataset. Sensitivity was assessed by computing the relative error between the β estimates obtained from the 5-month dataset and the average β estimate over all datasets considered (i.e., 4-, 5-, and 6-month periods). Each 4-month dataset consists of state-specific daily case counts from January 21, 2020 to May 21, 2020. Each 5-month dataset consists of state-specific daily case counts from January 21, 2020 to June 21, 2020. Each 6-month dataset consists of state-specific daily case counts from January 21, 2020 to July 21, 2020.

State	Relative error (%)
Alabama	5.056736841
Alaska	0.135565694
Arizona	1.580036956
Arkansas	4.050499367
California	7.230353936
Colorado	1.169110891
Connecticut	1.71856443
Delaware	1.605396619
Florida	3.860395675
Georgia	2.046222168
Hawaii	0.07301952
Idaho	5.354867104
Illinois	1.067352626
Indiana	0.482148062
Iowa	1.750513215
Kansas	12.52579428
Kentucky	1.722236898
Louisiana	1.248313993
Maine	0.677311718
Maryland	0.767179733
Massachusetts	1.291664711
Michigan	0.683144998
Minnesota	9.842300289
Mississippi	0.140202483
Missouri	4.896925896
Montana	0.153819893
Nebraska	0.385488999
Nevada	0.828093273
New Hampshire	1.774059408
New Jersey	1.399445444
New Mexico	0.633608589
New York	7.806630712

North Carolina	0.822704993
North Dakota	0.353431338
Ohio	4.835068796
Oklahoma	0.090108154
Oregon	1.9254369
Pennsylvania	0.137363347
Rhode Island	2.482979345
South Carolina	0.549584631
South Dakota	0.563102005
Tennessee	0.262716142
Texas	1.453009268
Utah	2.747788206
Vermont	7.394371088
Virginia	2.196551742
Washington	2.906929126
West Virginia	2.007787943
Wisconsin	6.239810825
Wyoming	1.86485316

Table S5. Percent progress $\mathcal{P}$ toward herd immunity for each of the 50 states. This table provides the inputs to Eq. 2 that were used to generate the results plotted in Fig. 8. In Case 1, f_r is given in column 6. In Case 2, f_r is given in column 8. Note that $f_A = 0.44$. In both cases, $\varepsilon_v = 0.66$ and $\varepsilon_r = 1.0$.

State	S_0	Y_{Delta}	$1 - \frac{1}{Y_{\text{Delta}}\mathcal{R}_0}$	N_d/S_0	$\left(\frac{N_d}{S_0}\right) \times 5.8$	f_s	$f_s/(1-f_A)$	f_v	$\mathcal{P}$ (Case 1)	$\mathcal{P}$ (Case 2)
Alabama	4903185	2.46	0.9044	0.1575	0.9136	0.292	0.5214	0.4127	103.6217	72.0726
Alaska	731545	2.46	0.8213	0.1390	0.8063	0.093	0.1661	0.4909	105.8143	53.1165
Arizona	7278717	2.46	0.9312	0.1468	0.8517	0.297	0.5304	0.5029	96.7460	73.6934
Arkansas	3017804	2.46	0.8679	0.1609	0.9330	0.252	0.4500	0.4460	109.7714	70.5032
California	39512223	2.46	0.9033	0.1180	0.6844	0.211	0.3768	0.5788	89.1154	68.0675
Colorado	5758736	2.46	0.8996	0.1141	0.6616	0.096	0.1714	0.5857	88.0859	54.6621
Connecticut	3565287	2.46	0.9183	0.1082	0.6276	0.065	0.1161	0.6774	86.4714	55.6717
Delaware	973764	2.46	0.8643	0.1322	0.7665	0.215	0.3839	0.5679	98.8090	71.1368
Florida	21477737	2.46	0.9322	0.1631	0.9462	0.254	0.4536	0.5584	103.6342	70.2618
Georgia	10617423	2.46	0.9166	0.1416	0.8212	0.124	0.2214	0.4407	95.2713	48.8630
Hawaii	1415872	2.46	0.8459	0.0520	0.3014	0.016	0.0286	0.5701	66.7006	46.5866
Idaho	1787065	2.46	0.9071	0.1363	0.7908	0.109	0.1946	0.4078	93.3873	45.3530
Illinois	12671821	2.46	0.9269	0.1265	0.7339	0.319	0.5696	0.5270	89.1686	77.6101
Indiana	6733219	2.46	0.9300	0.1393	0.8080	0.195	0.3482	0.4768	93.3745	59.4952
Iowa	3155070	2.46	0.8684	0.1376	0.7983	0.127	0.2268	0.5318	100.0827	57.3692
Kansas	2913314	2.46	0.8857	0.1375	0.7972	0.085	0.1518	0.5020	97.5952	48.8669
Kentucky	4467673	2.46	0.8867	0.1484	0.8607	0.255	0.4554	0.5083	102.3341	71.9576
Louisiana	4648794	2.46	0.9232	0.1568	0.9093	0.103	0.1839	0.4436	101.3706	45.8047
Maine	1344212	2.46	0.8344	0.0624	0.3621	0.05	0.0893	0.6748	77.4412	59.3086
Maryland	6045680	2.46	0.9086	0.0861	0.4993	0.293	0.5232	0.6328	77.9720	79.5039
Massachusetts	6892503	2.46	0.9015	0.1154	0.6694	0.086	0.1536	0.6725	90.5334	58.7074
Michigan	9986857	2.46	0.9376	0.1117	0.6480	0.281	0.5018	0.5172	81.9332	71.6587
Minnesota	5639632	2.46	0.8753	0.1213	0.7036	0.112	0.2000	0.5752	93.2384	57.5475
Mississippi	2976149	2.46	0.9213	0.1600	0.9278	0.338	0.6036	0.4213	102.8846	77.4756
Missouri	6137428	2.46	0.9214	0.1335	0.7744	0.237	0.4232	0.4700	91.6428	65.3515
Montana	1068778	2.46	0.8299	0.1324	0.7678	0.085	0.1518	0.4774	101.3321	50.4968

Nebraska	1934408	2.46	0.8478	0.1340	0.7772	0.109	0.1946	0.5367	100.9768	56.6056
Nevada	3080156	2.46	0.8950	0.1339	0.7764	0.267	0.4768	0.4982	94.9681	72.4986
New Hampshire	1359711	2.46	0.8696	0.0849	0.4923	0.065	0.1161	0.6099	80.1139	54.2680
New Jersey	8882190	2.46	0.9427	0.1278	0.7410	0.303	0.5411	0.6347	90.1171	77.7900
New Mexico	2096829	2.46	0.8664	0.1174	0.6811	0.12	0.2143	0.6215	93.7109	61.9322
New York	19453561	2.46	0.9304	0.1221	0.7080	0.123	0.2196	0.6256	89.0551	58.2348
North Carolina	10488084	2.46	0.9083	0.1286	0.7456	0.226	0.4036	0.4868	91.0880	65.5284
North Dakota	762062	2.46	0.8229	0.1654	0.9594	-	-	0.4333	117.9901	34.7532
Ohio	11689100	2.46	0.9000	0.1157	0.6709	0.333	0.5946	0.4961	86.5202	80.8211
Oklahoma	3956971	2.46	0.8983	0.1511	0.8766	0.111	0.1982	0.4647	101.7986	49.4386
Oregon	4217737	2.46	0.8800	0.0742	0.4306	0.071	0.1268	0.5987	74.4978	53.6129
Pennsylvania	12801989	2.46	0.9250	0.1080	0.6265	0.226	0.4036	0.5691	82.8942	67.8431
Rhode Island	1059361	2.46	0.8782	0.1599	0.9272	0.103	0.1839	0.6696	109.2411	62.0112
South Carolina	5148714	2.46	0.9009	0.1613	0.9353	0.262	0.4679	0.4595	105.9958	69.8451
South Dakota	884659	2.46	0.8796	0.1587	0.9205	0.341	0.6089	0.5076	107.6770	84.1227
Tennessee	6829174	2.46	0.9141	0.1703	0.9880	0.266	0.4750	0.4411	108.4681	68.6860
Texas	28995881	2.46	0.9098	0.1358	0.7879	0.319	0.5696	0.5019	94.3166	78.2778
Utah	3205958	2.46	0.8842	0.1542	0.8946	0.293	0.5232	0.4954	105.0729	76.8066
Vermont	623989	2.46	0.8820	0.0509	0.2952	0.018	0.0321	0.6893	69.8241	53.5661
Virginia	8535519	2.46	0.8941	0.0980	0.5682	0.173	0.3089	0.5953	82.5214	64.9181
Washington	7614893	2.46	0.8985	0.0828	0.4802	0.07	0.1250	0.6223	77.2079	53.9088
West Virginia	1792147	2.46	0.8371	0.1252	0.7263	0.09	0.1607	0.4015	95.4285	45.7627
Wisconsin	5822534	2.46	0.9102	0.1339	0.7769	0.285	0.5089	0.5563	94.3549	75.7240
Wyoming	578759	2.46	0.8211	0.1477	0.8568	0.308	0.5500	0.4063	109.0295	81.6820

Table S6. Comparison of estimates of the basic reproduction number $\mathcal{R}_0$ for ancestral strains of SARS-CoV-2 from four different studies for each of the 30 states considered in all of the studies.

	$\mathcal{R}_0$ estimate			
State	This study	Ref. [16]	Ref. [17]	Ref. [18]
Kansas	4.25	1.418	2.363	4.212
Michigan	5.91	1.299	2.234	4.166
Montana	4.204	2.447	2.196	3.294
Maine	4.049	2.077	2.286	3.463
Colorado	4.977	3.18	2.675	5.143
Tennessee	2.996	1.387	1.886	3.109
Louisiana	5.994	1.803	2.285	5.284
Kentucky	4.872	2.914	2.311	5.629
Iowa	5.294	4.558	2.376	3.703
Connecticut	4.446	3.145	2.584	4.133
Arizona	4.127	3.011	2.805	3.309
Ohio	6.51	5.083	2.002	7.533
Hawaii	3.261	1.599	2.467	3.013
Massachusetts	5.167	1.314	2.247	7.707
Mississippi	5.169	1.258	2.234	5.277
Nebraska	3.87	1.217	2.131	3.759
Alaska	7.093	1.357	2.961	6.814
South Dakota	3.042	4.558	1.899	2.9
Alabama	5.842	6.463	3.275	4.246
Florida	4.433	2.774	2.558	4.895
Delaware	4.063	1.473	2.596	4.938
Nevada	3.998	2.368	2.118	4.903
California	5.423	1.028	2.714	4.839
Maryland	3.338	2.82	2.247	2.817
Oregon	4.102	1.321	1.963	4.647
Idaho	4.73	1.191	2.428	5.07
Illinois	4.509	0.947	2.402	4.396
New Jersey	3.839	1.403	2.079	3.453
Arizona	4.004	1.652	1.274	3.46
Georgia	4.527	2.267	2.493	5.406

Figure S1. Consistency of results obtained from different codes used to perform Markov chain Monte Carlo (MCMC) sampling. Shown here are 1-dimensional marginal posteriors of parameters for Wyoming ($n = 0$) derived using the Python code of Lin et al. [1] (blue) and PyBioNetFit [4] (red).

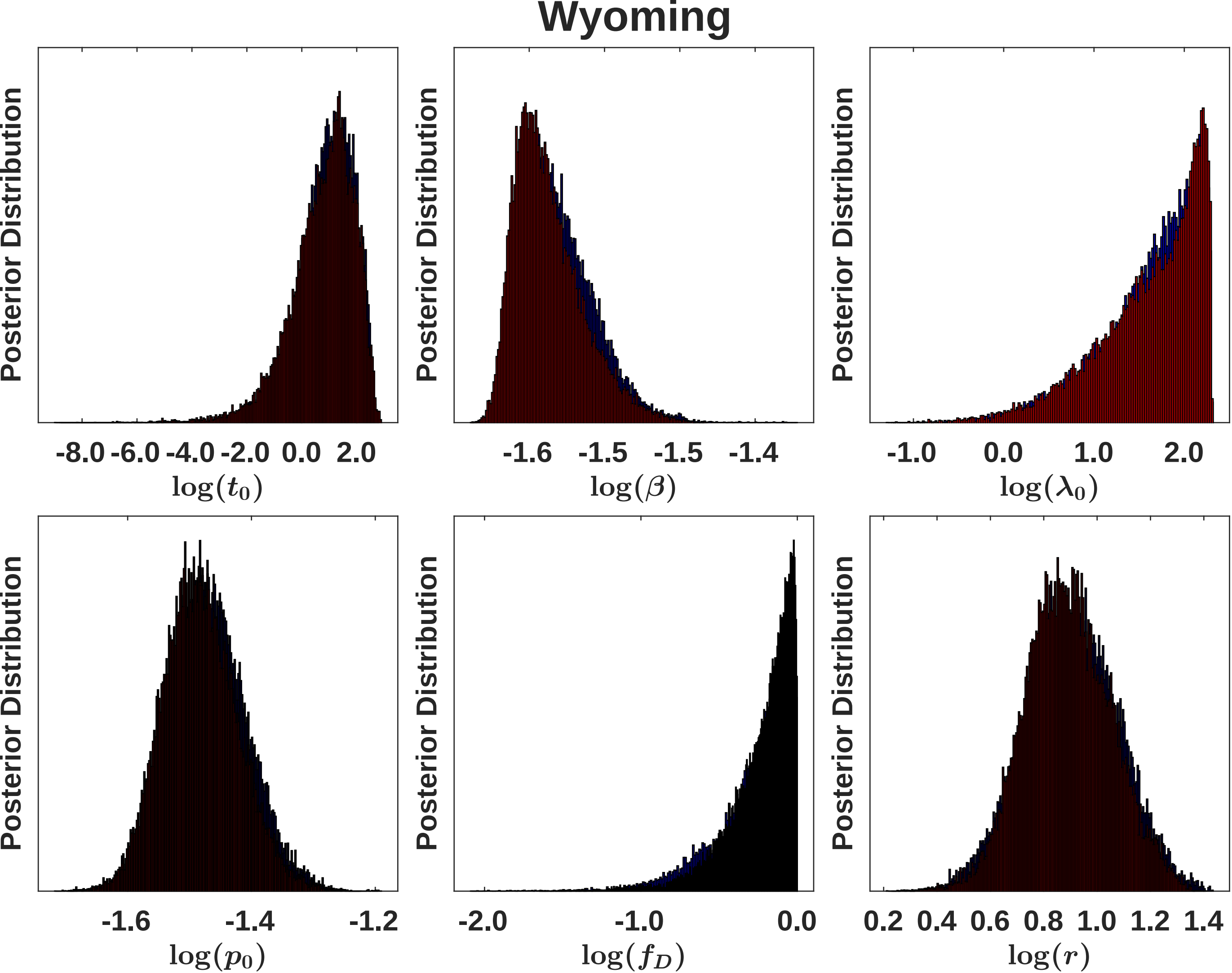

Wyoming
Posterior Distribution
-8.0 -6.0 -4.0 -2.0 0.0 2.0
log(t_0)
-1.6 -1.5 -1.5 -1.4
log(β)
-1.0 0.0 1.0 2.0
log(λ_0)
-1.6 -1.4 -1.2
log(p_0)
-2.0 -1.0 0.0
log(f_D)
0.2 0.4 0.6 0.8 1.0 1.2 1.4
log(r)

Figure S2. Markov chain log-likelihood trace plots for each of the 50 US states. Bayesian inference was conditioned on the compartmental model of Lin et al. [1]. Bayesian inference was performed as described by Lin et al. [1] except that training data consisted of daily COVID-19 case counts for states (vs. case counts for metropolitan statistical areas). The compartmental model accounts for an initial social distancing period followed by n additional periods. We considered $n = 0, 1$ and 2 and selected the best n using the model selection procedure described by Lin et al. [1]. The number of epochs (or iterations) used for each state was chosen so that convergence was achieved in each case. Inferences are based on daily reports of new cases of COVID-19 from January 21 to June 21, 2020.

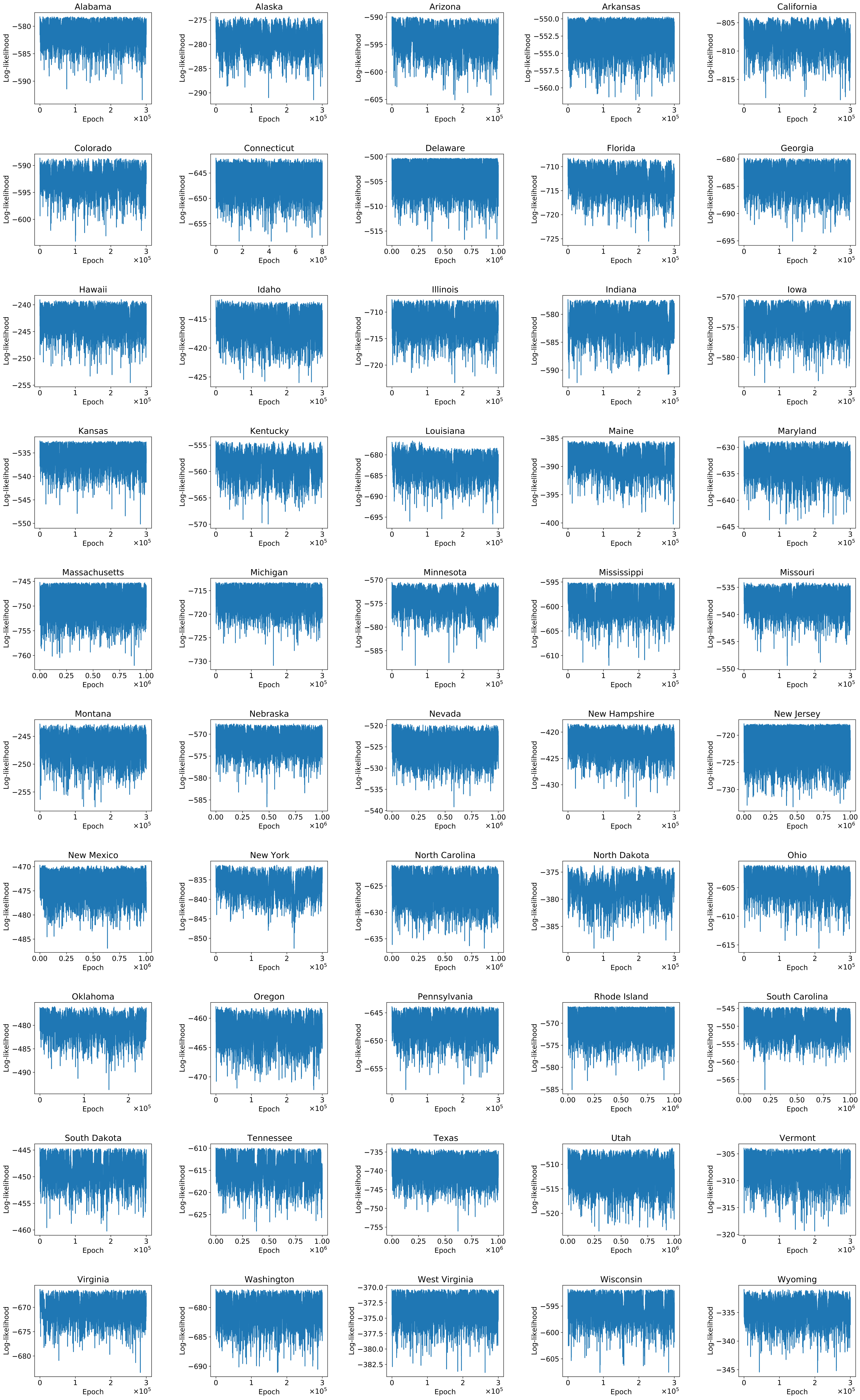
Alabama
Alaska
Arizona
Arkansas
California
Colorado
Connecticut
Delaware
Florida
Georgia
Hawaii
Idaho
Illinois
Indiana
Iowa
Kansas
Kentucky
Louisiana
Maine
Maryland
Massachusetts
Michigan
Minnesota
Mississippi
Missouri
Montana
Nebraska
Nevada
New Hampshire
New Jersey
New Mexico
New York
North Carolina
North Dakota
Ohio
Oklahoma
Oregon
Pennsylvania
Rhode Island
South Carolina
South Dakota
Tennessee
Texas
Utah
Vermont
Virginia
Washington
West Virginia
Wisconsin
Wyoming
Log-likelihood
Epoch

Figure S3. Parameter trace plots for each of the 50 US states. These parameter trace plots are matched to the likelihood trace plots of Fig S2. It should be noted that the number of parameters varies across the states depending on the selected value of n. See the caption of Fig S2 for additional details.

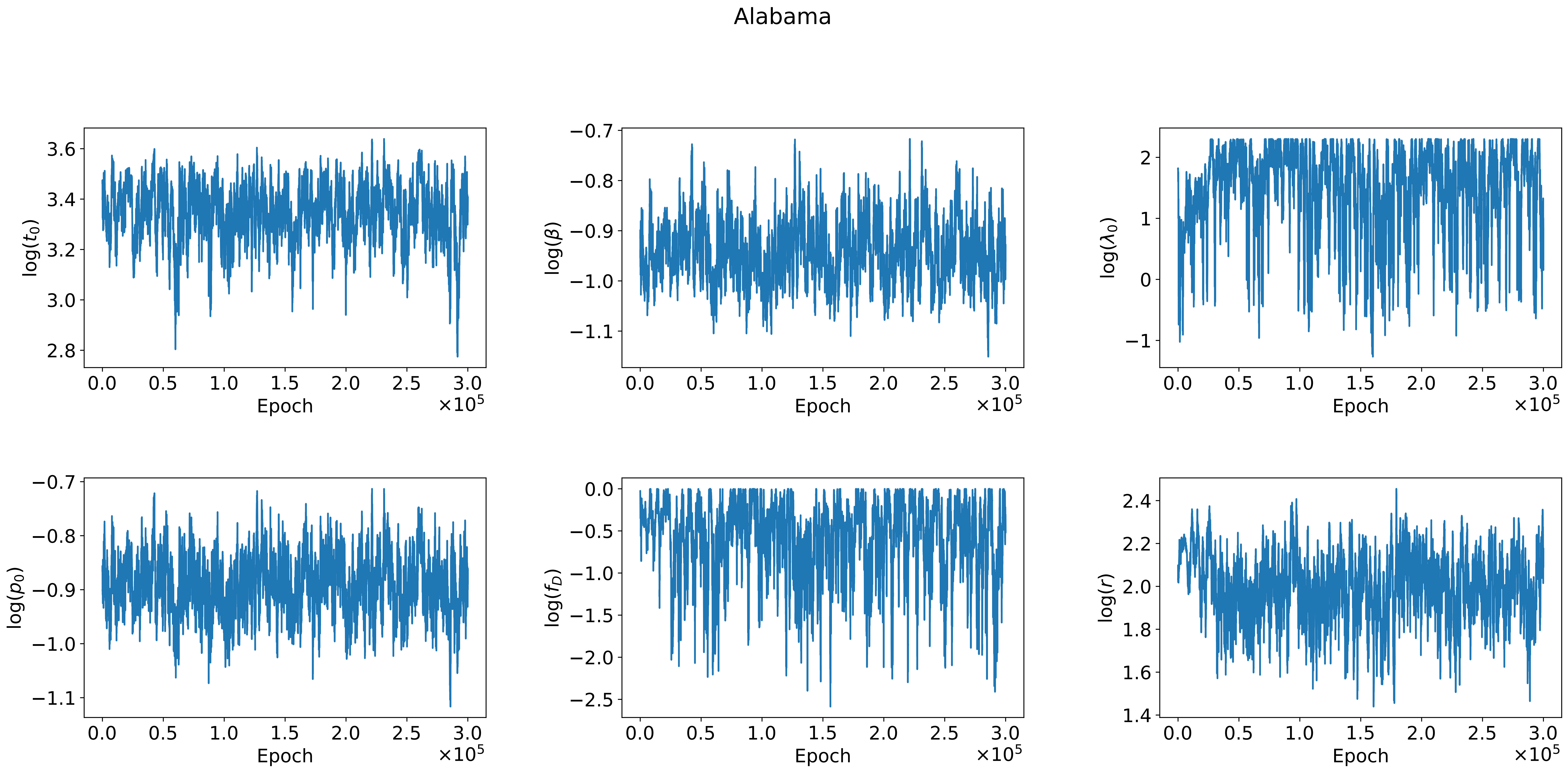

Alabama
log(t_0)
log(β)
log(λ_0)
log(p_0)
log(f_D)
log(r)
Epoch
$\times 10^5$

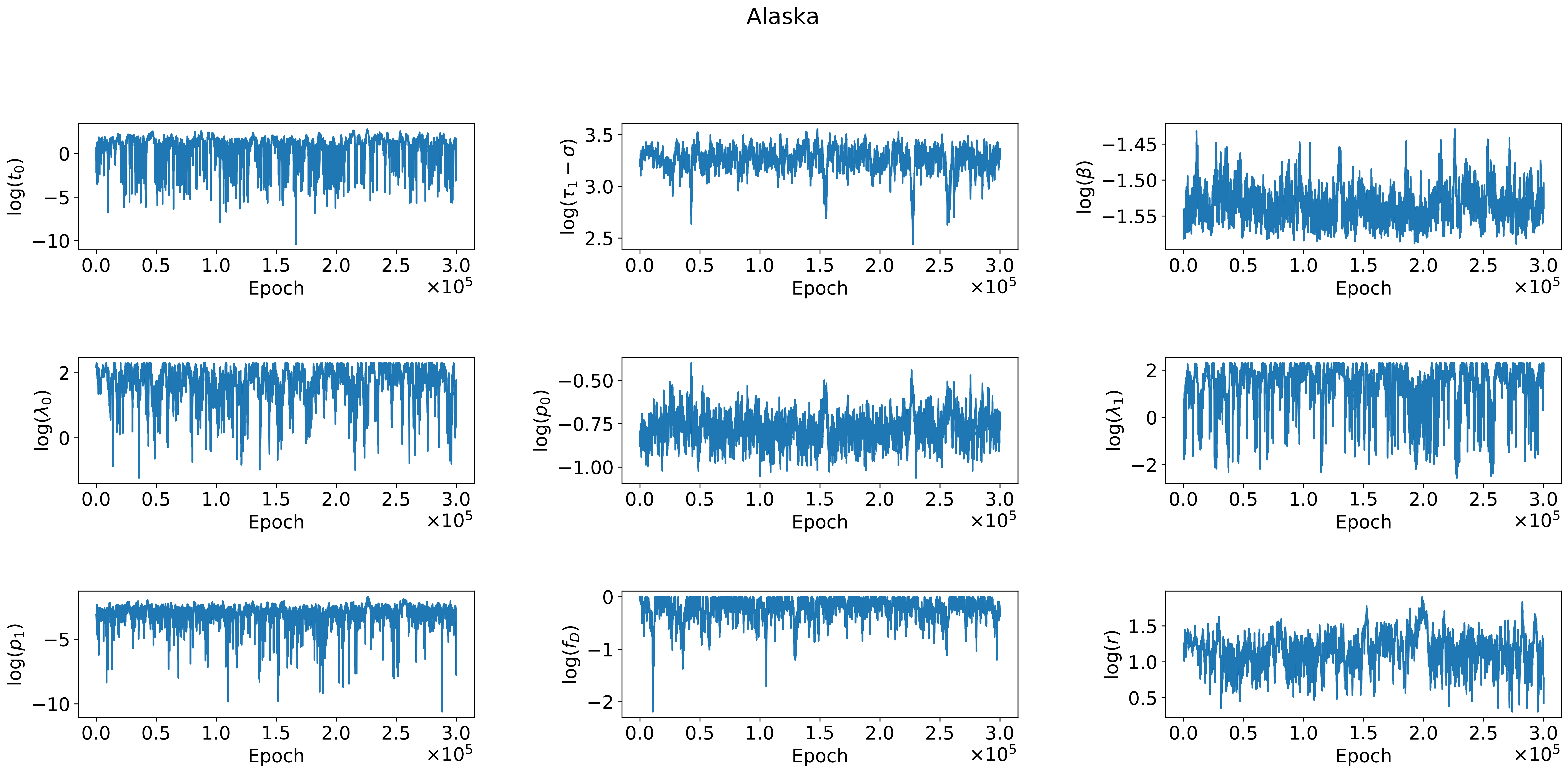

Alaska
log(t_0)
log($\tau_1 - \sigma$)
log(β)
log(λ_0)
log(p_0)
log(λ_1)
log(p_1)
log(f_D)
log(r)
Epoch
$\times 10^5$

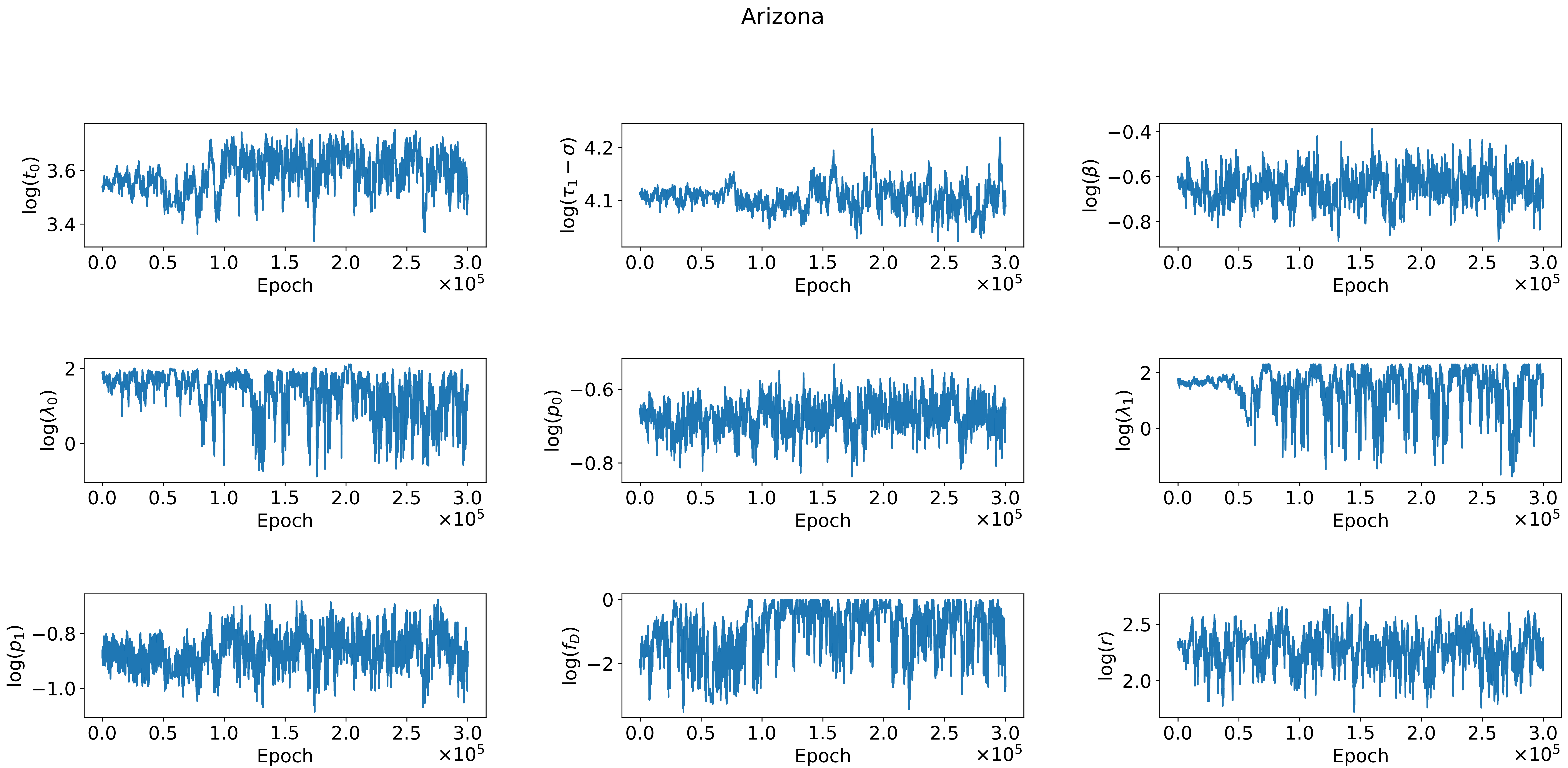

Arizona
log(t_0)
log($\tau_1 - \sigma$)
log(β)
log(λ_0)
log(p_0)
log(λ_1)
log(p_1)
log(f_D)
log(r)
Epoch
$\times 10^5$

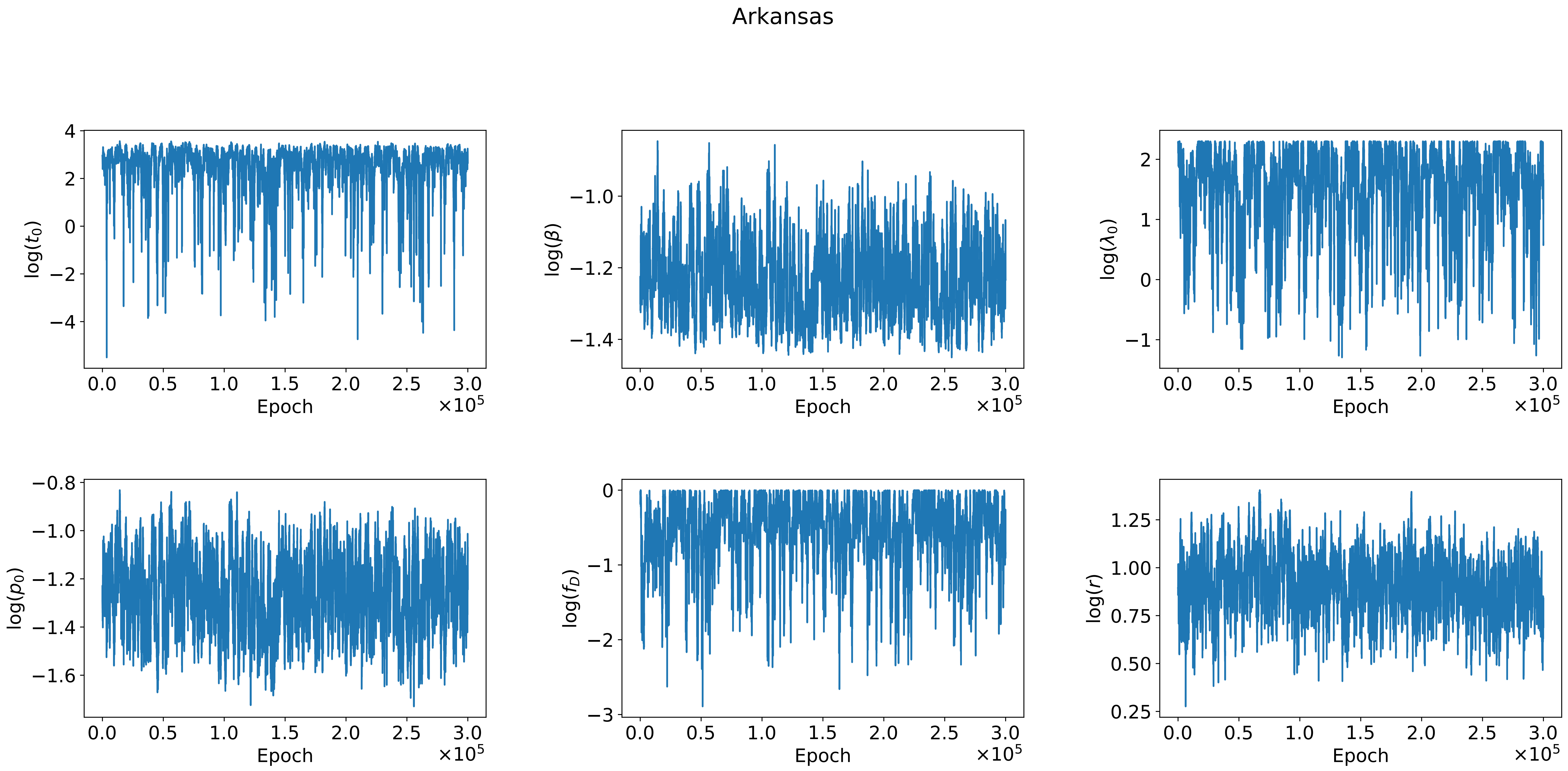

Arkansas
log(t_0)
log(β)
log(λ_0)
log(p_0)
log(f_D)
log(r)
Epoch
$\times 10^5$

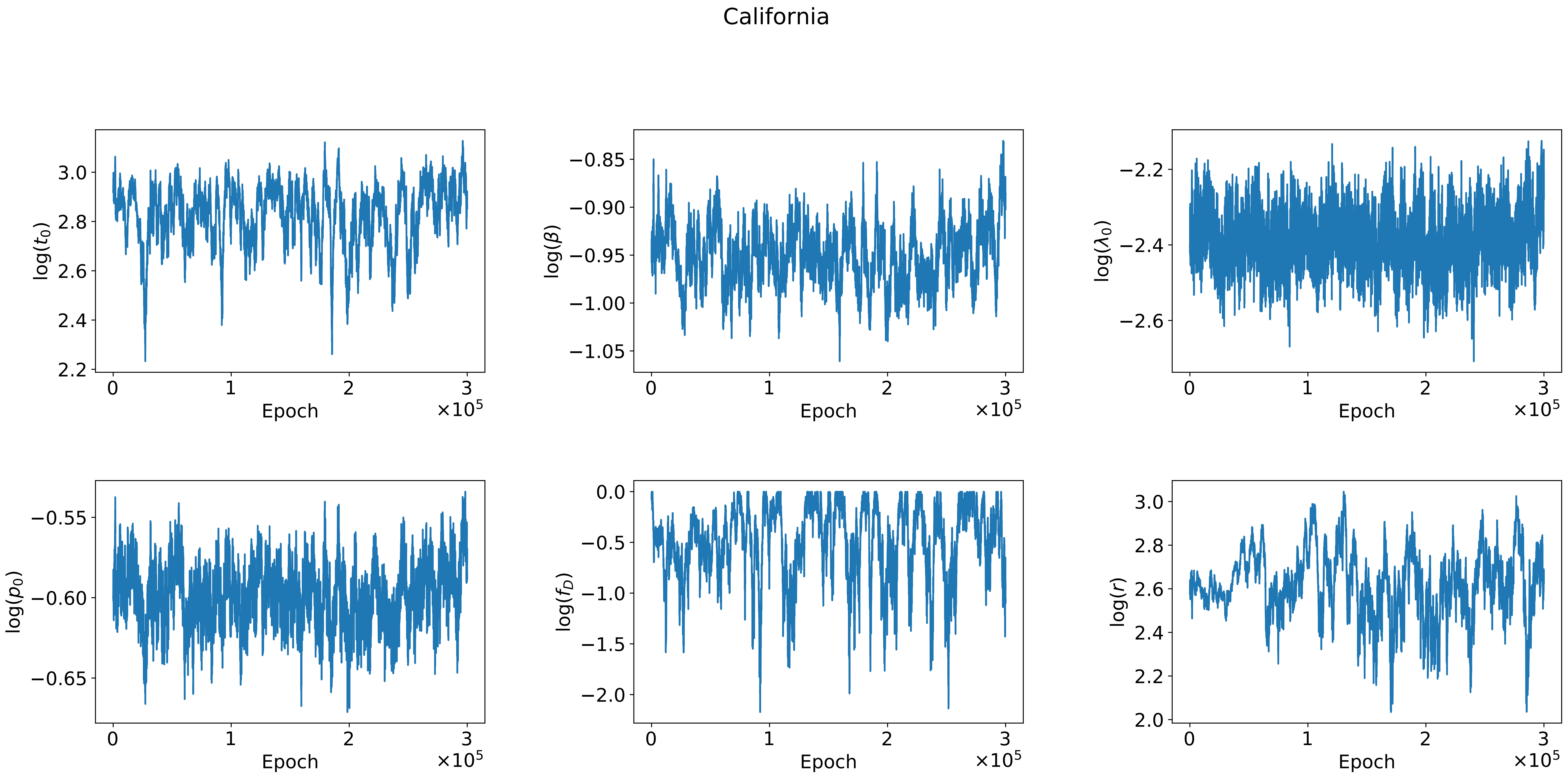
California
log(t_0)
log(β)
log(λ_0)
log(p_0)
log(f_D)
log(r)
Epoch
×10^5

Colorado

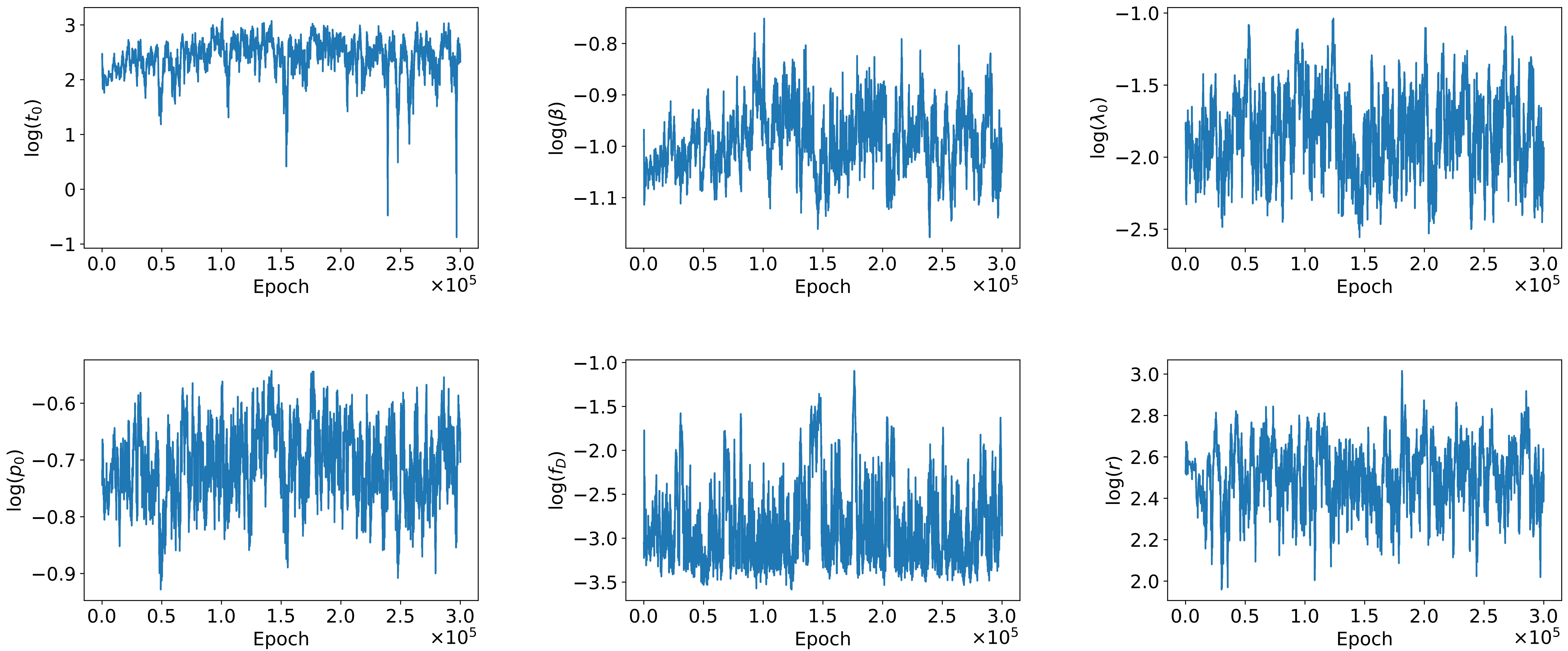

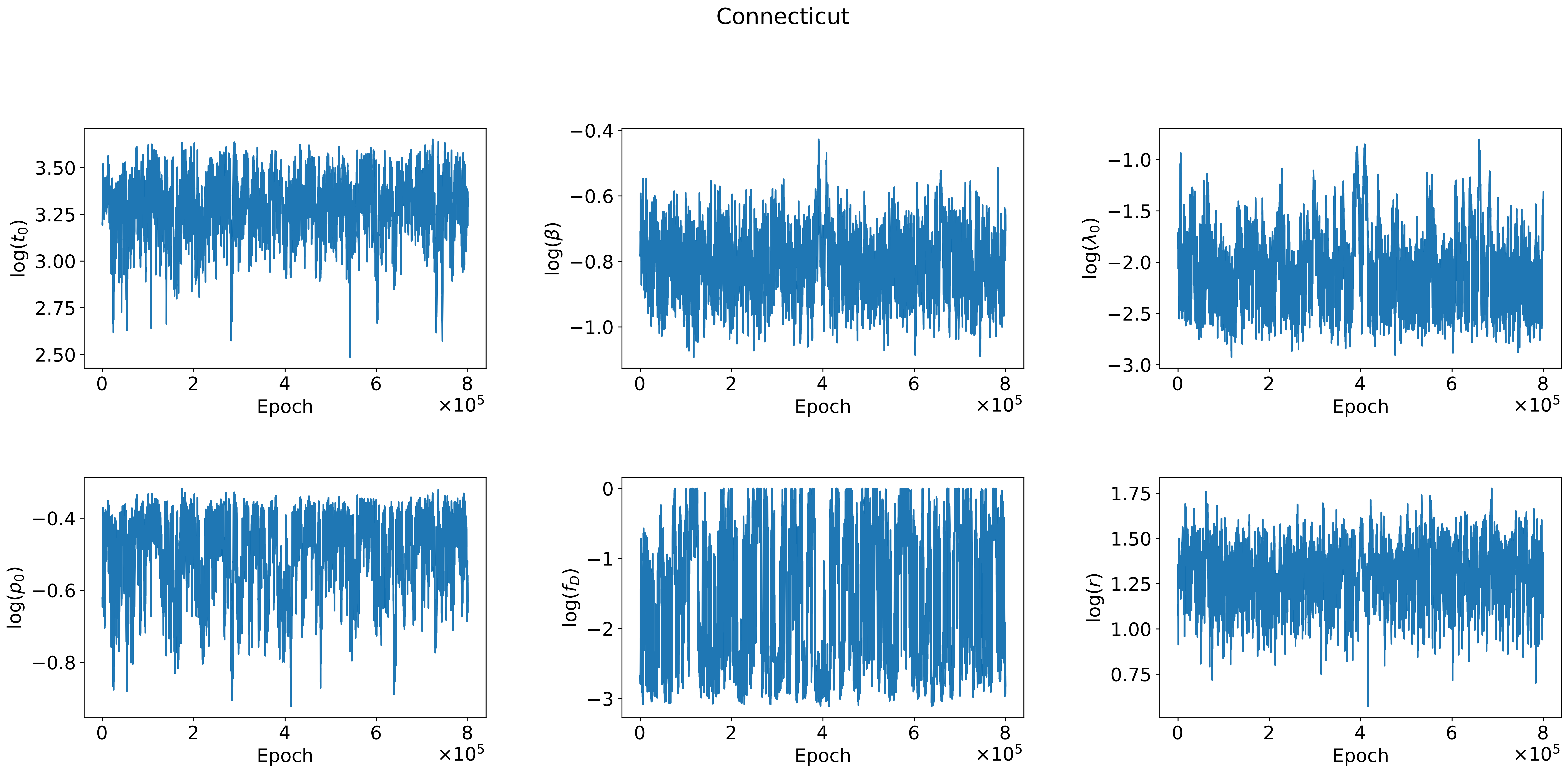
Connecticut
log(t_0)
log(β)
log(λ_0)
log(p_0)
log(f_D)
log(r)
Epoch
$\times 10^5$

Delaware

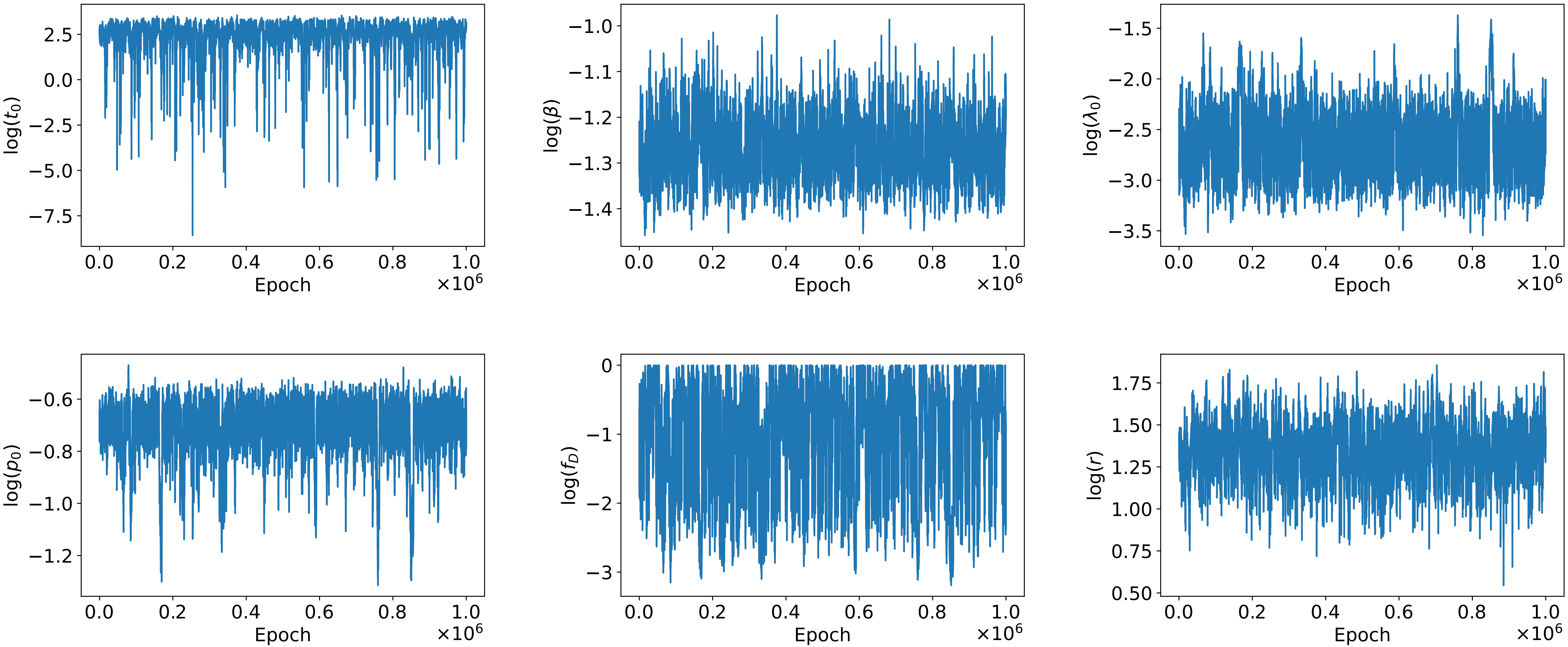

log(t_0)
2.5
0.0
−2.5
−5.0
−7.5
0.0 0.2 0.4 0.6 0.8 1.0
Epoch
×10^6
log(β)
−1.0
−1.1
−1.2
−1.3
−1.4
log(λ_0)
−1.5
−2.0
−2.5
−3.0
−3.5
log(p_0)
−0.6
−0.8
−1.0
−1.2
log(f_D)
0
−1
−2
−3
log(r)
1.75
1.50
1.25
1.00
0.75
0.50

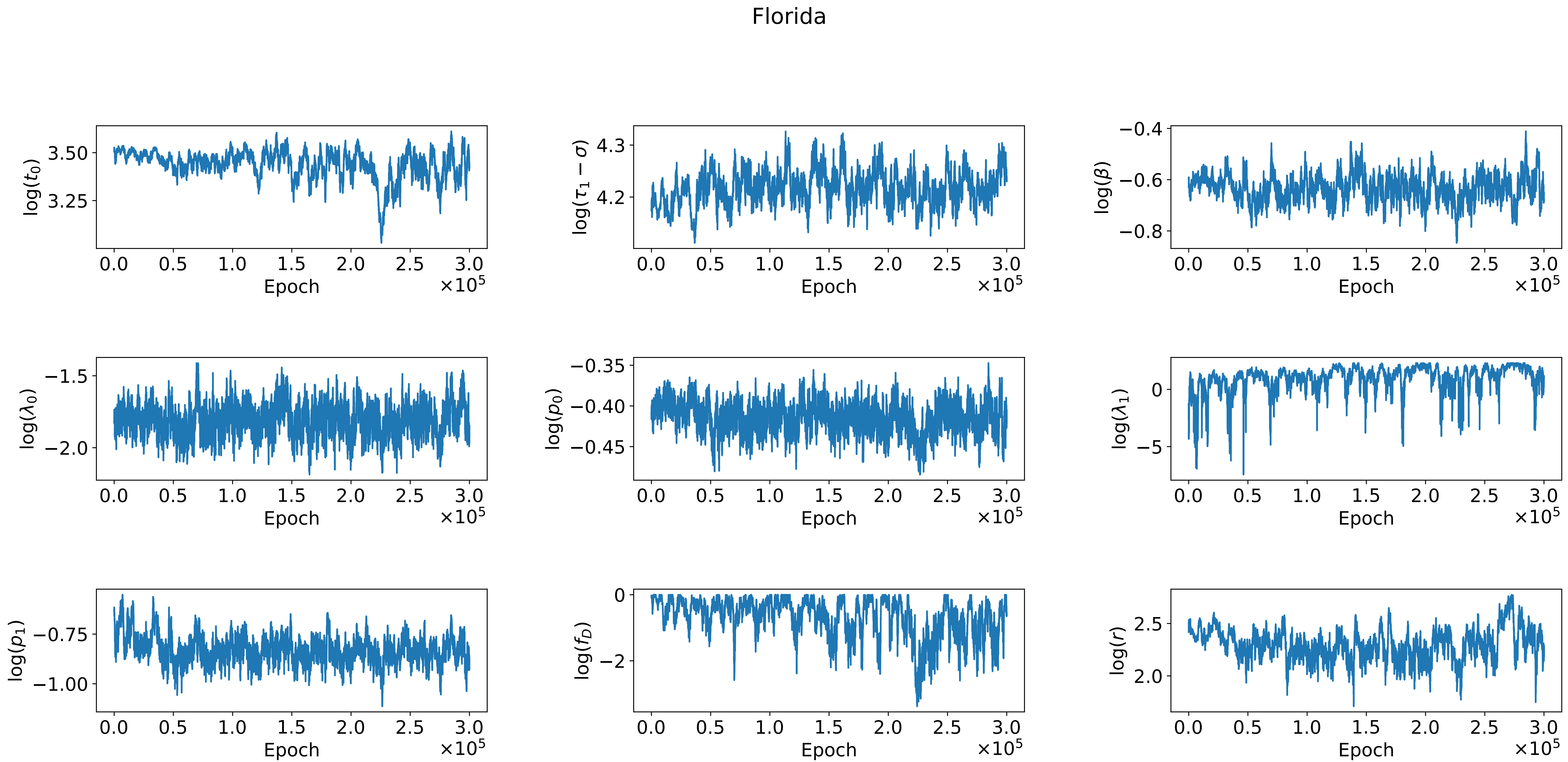

Florida
$\log(t_0)$
$\log(\tau_1 - \sigma)$
$\log(\beta)$
$\log(\lambda_0)$
$\log(p_0)$
$\log(\lambda_1)$
$\log(p_1)$
$\log(f_D)$
$\log(r)$
Epoch
$\times 10^5$

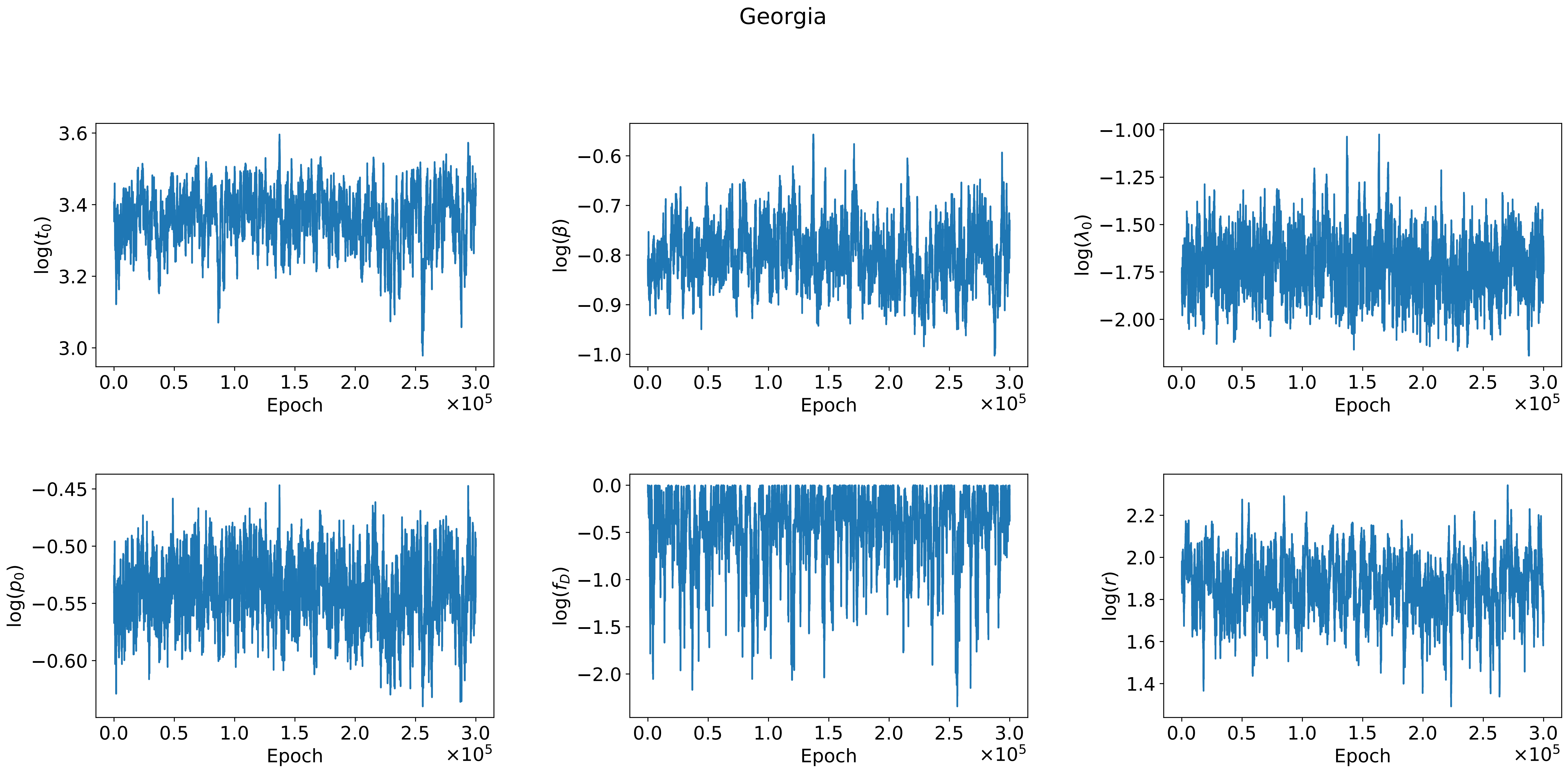

Georgia
$\log(t_0)$
$\log(\beta)$
$\log(\lambda_0)$
$\log(p_0)$
$\log(f_D)$
$\log(r)$
Epoch
$\times 10^5$

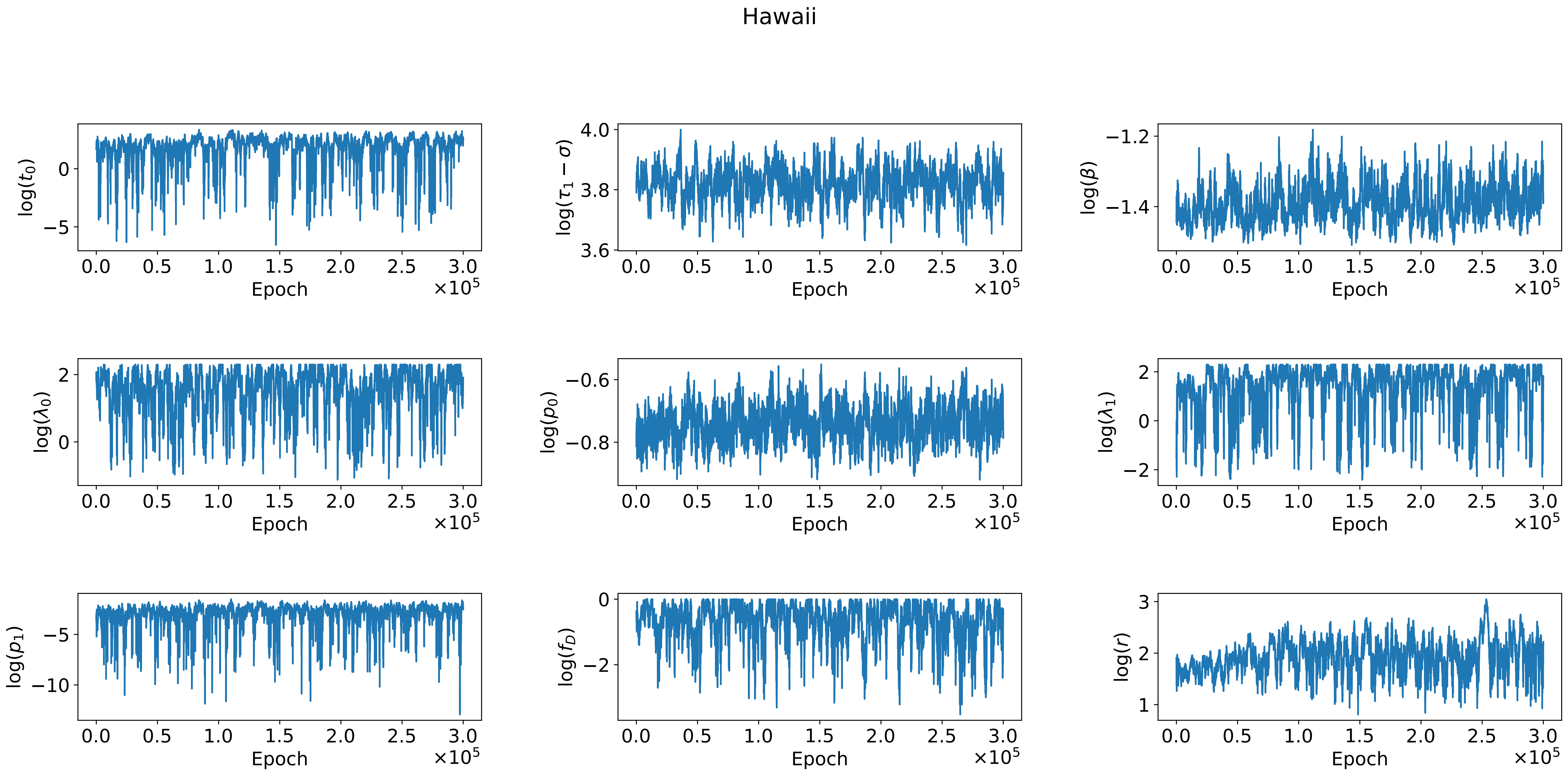
Hawaii
log(t_0)
log($\tau_1 - \sigma$)
log(β)
log(λ_0)
log(p_0)
log(λ_1)
log(p_1)
log(f_D)
log(r)
Epoch
$\times10^5$

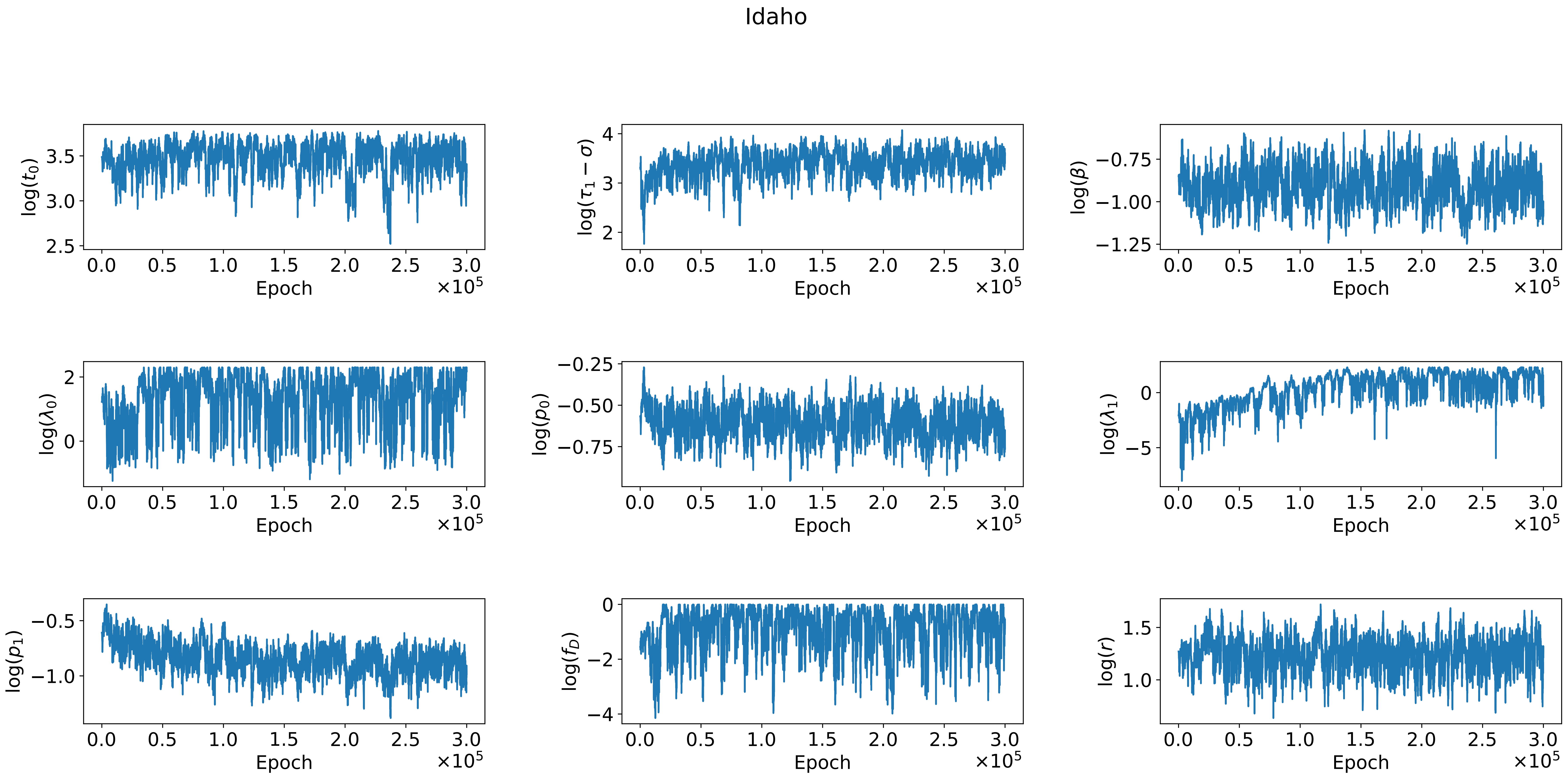
Idaho
log(t_0)
log($\tau_1 - \sigma$)
log(β)
log(λ_0)
log(p_0)
log(λ_1)
log(p_1)
log(f_D)
log(r)
Epoch
$\times 10^5$

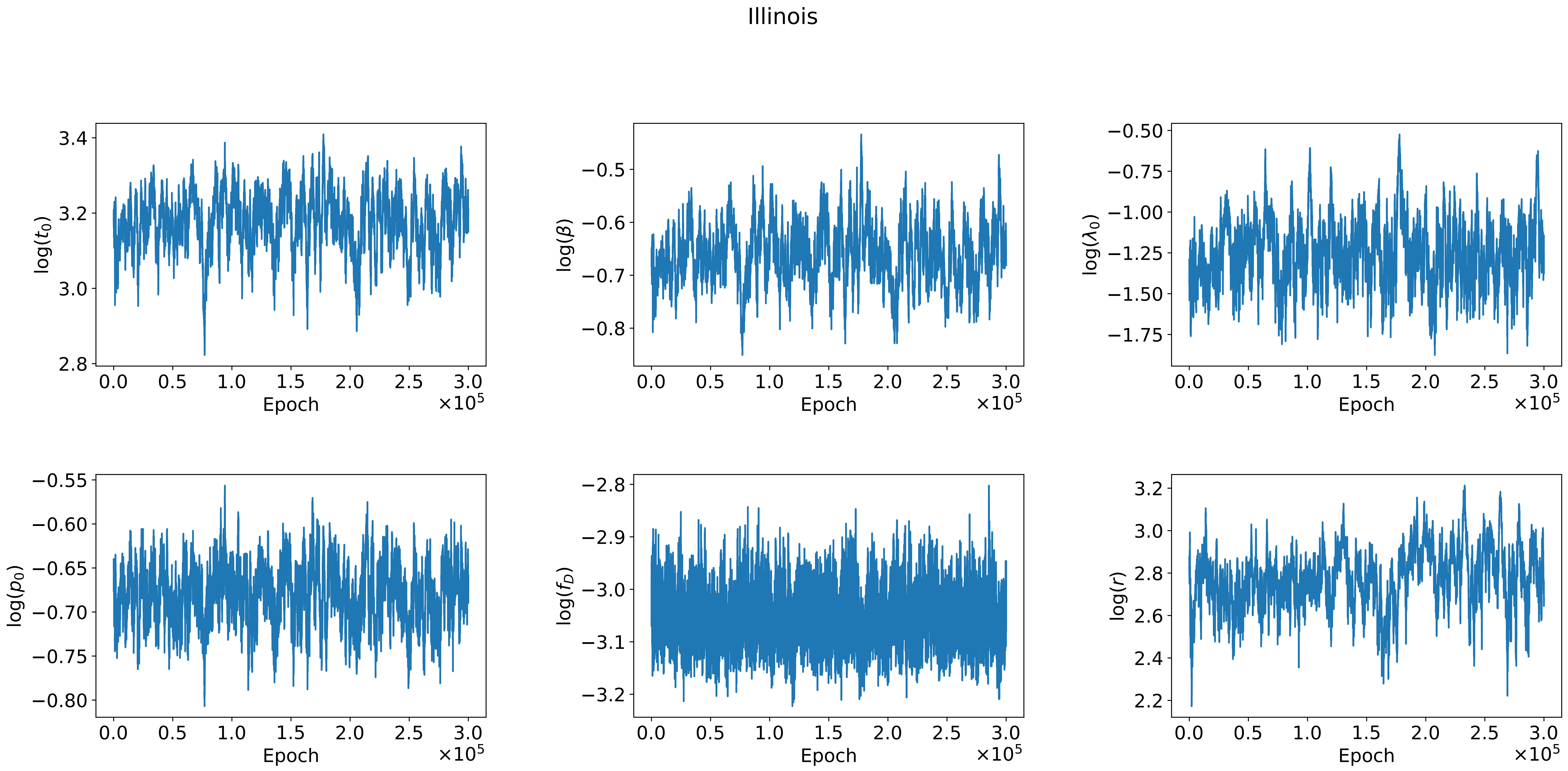

Illinois
log(t_0)
log(β)
log(λ_0)
log(p_0)
log(f_D)
log(r)
Epoch
$\times 10^5$

Indiana

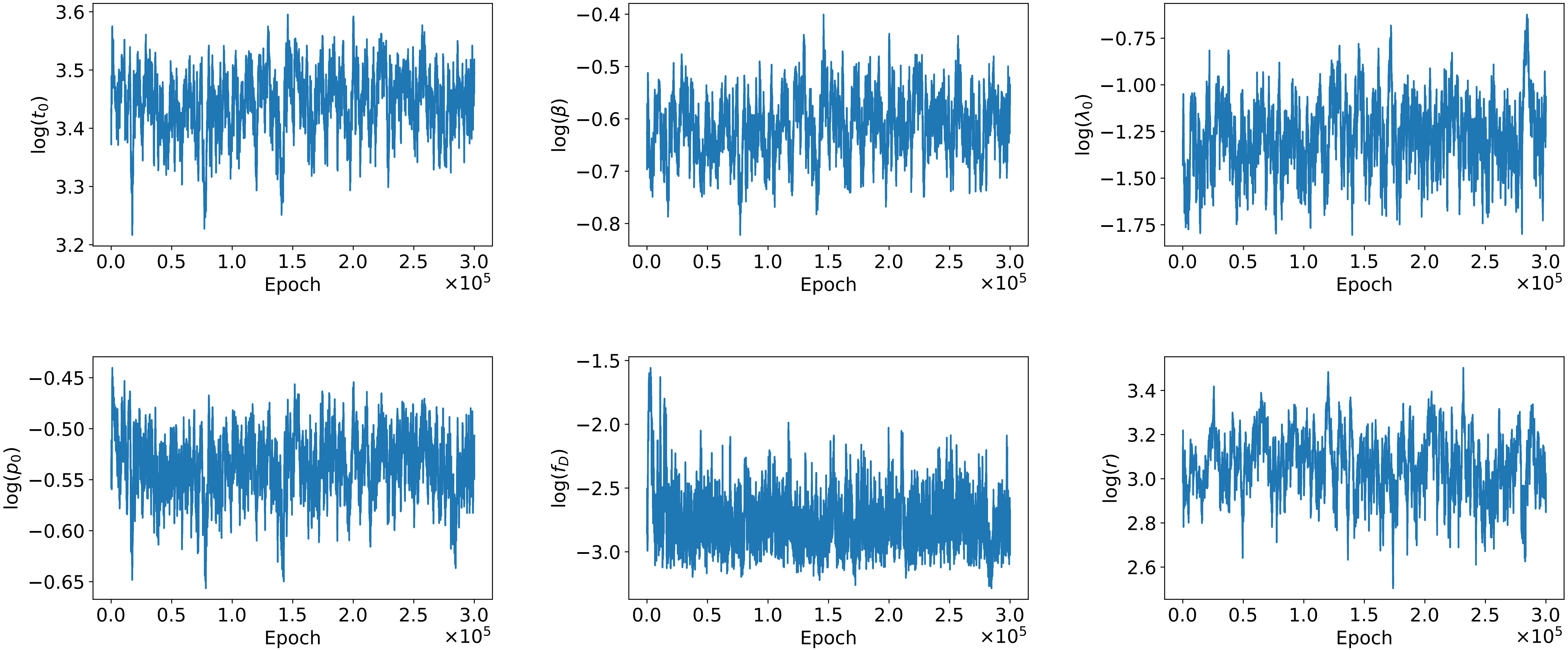

log(t_0)
log(β)
log(λ_0)
log(p_0)
log(f_D)
log(r)
Epoch
×10^5
0.0 0.5 1.0 1.5 2.0 2.5 3.0
3.2 3.3 3.4 3.5 3.6
−0.8 −0.7 −0.6 −0.5 −0.4
−1.75 −1.50 −1.25 −1.00 −0.75
−0.65 −0.60 −0.55 −0.50 −0.45
−3.0 −2.5 −2.0 −1.5
2.6 2.8 3.0 3.2 3.4

Iowa

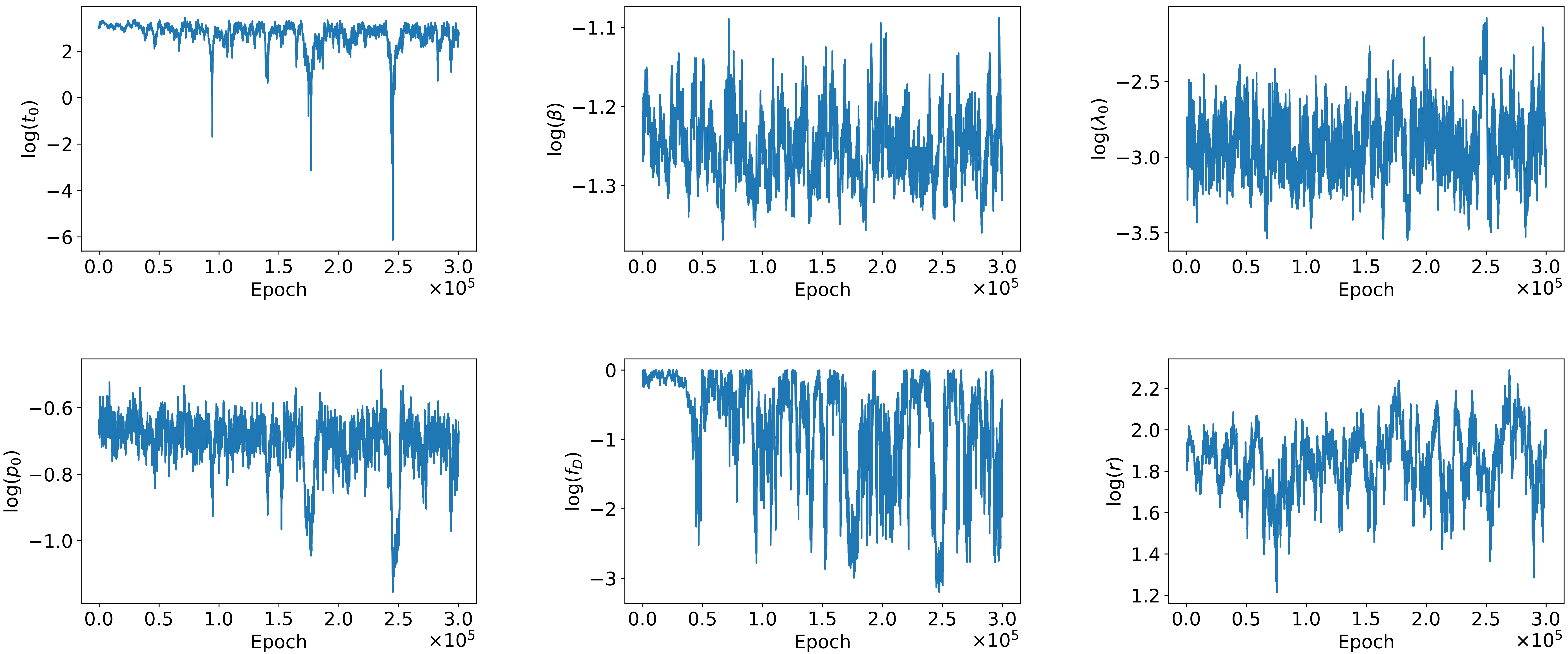

log(t_0)
2
0
−2
−4
−6
0.0 0.5 1.0 1.5 2.0 2.5 3.0
Epoch
×10^5
log(β)
−1.1
−1.2
−1.3
Epoch
×10^5
log(λ_0)
−2.5
−3.0
−3.5
Epoch
×10^5
log(p_0)
−0.6
−0.8
−1.0
Epoch
×10^5
log(f_D)
0
−1
−2
−3
Epoch
×10^5
log(r)
2.2
2.0
1.8
1.6
1.4
1.2
Epoch
×10^5

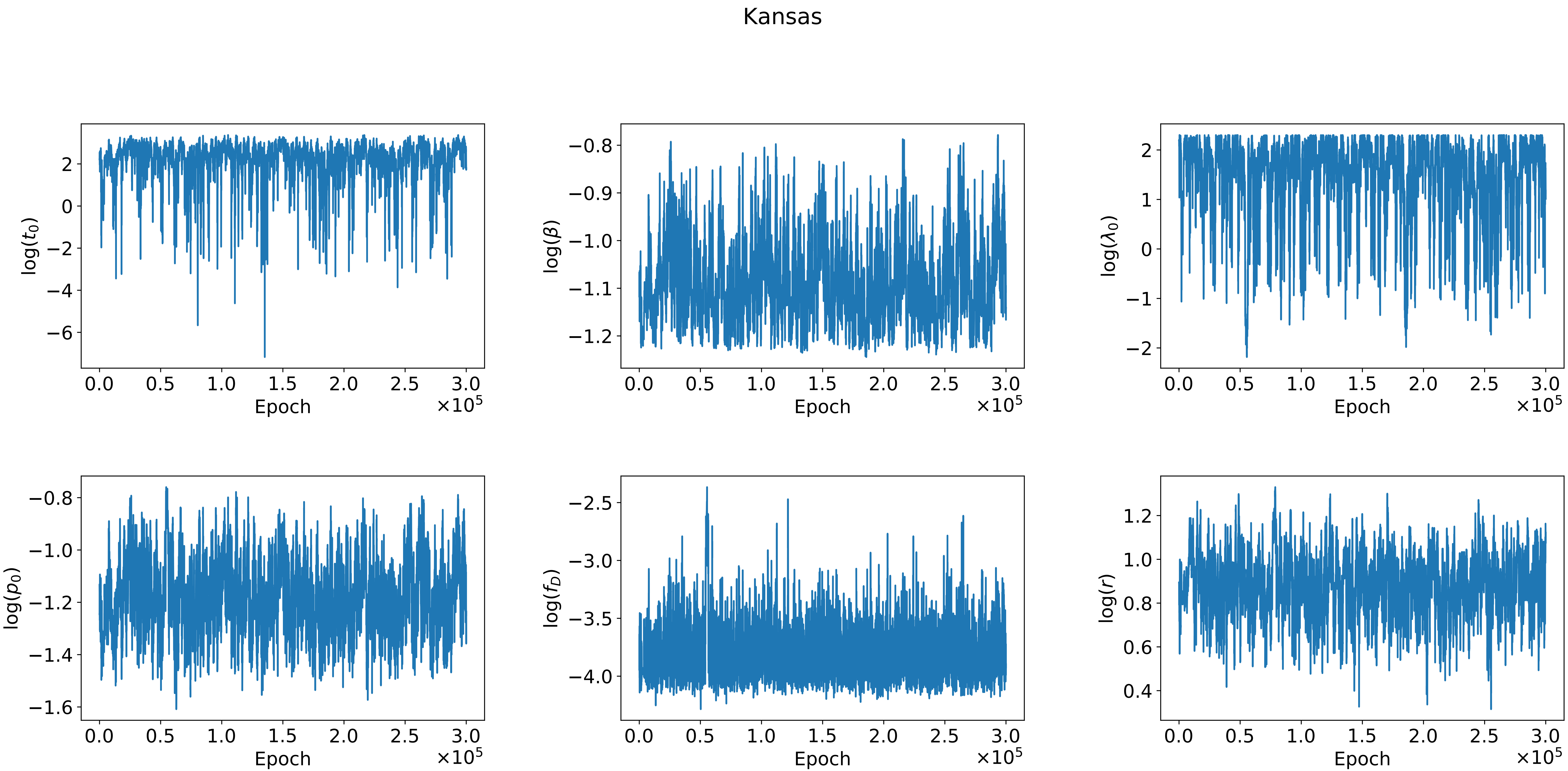

Kansas
log(t_0)
log(β)
log(λ_0)
log(p_0)
log(f_D)
log(r)
Epoch
$\times 10^5$

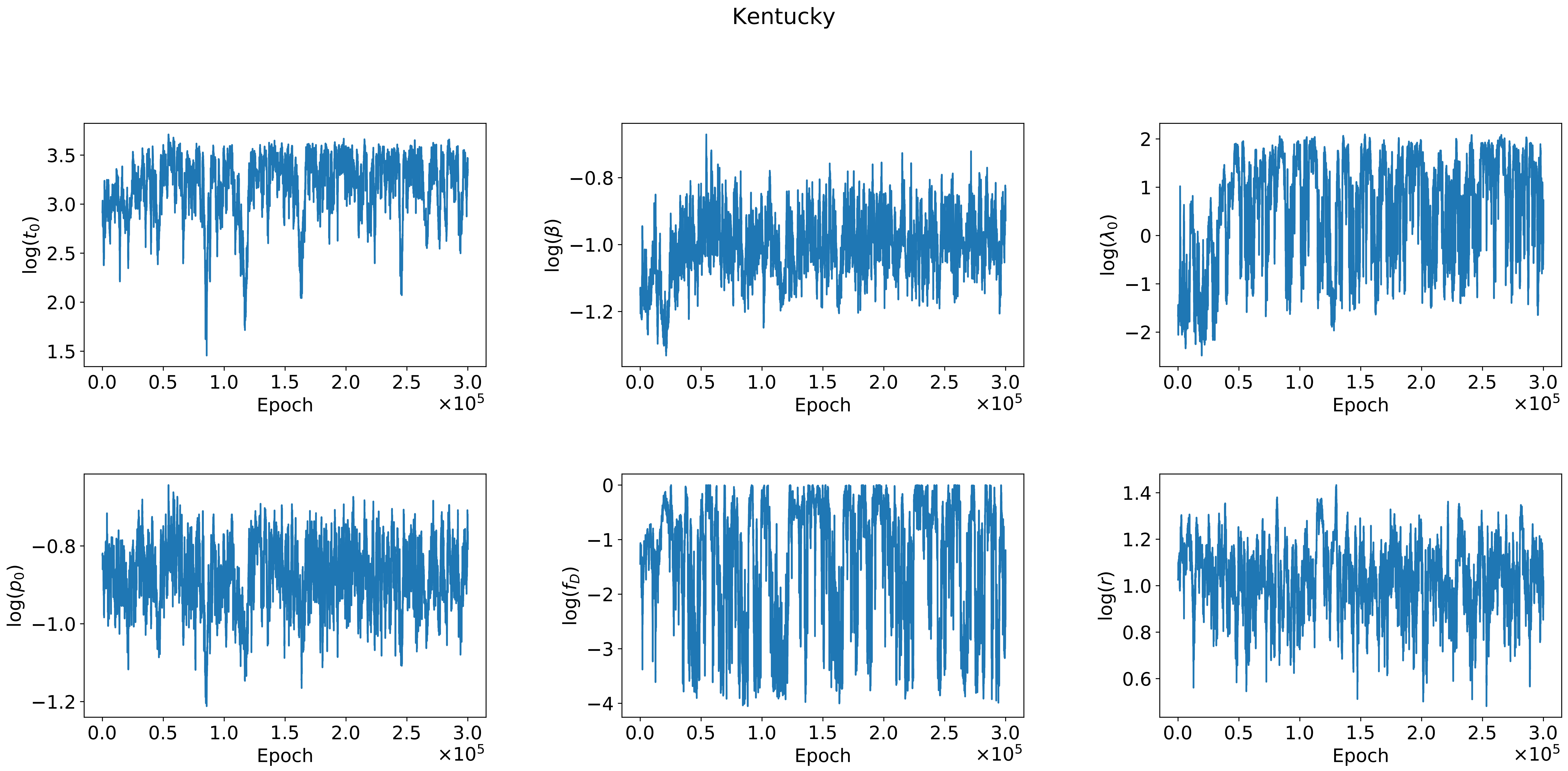
Kentucky
log(t_0)
log(β)
log(λ_0)
log(p_0)
log(f_D)
log(r)
Epoch
$\times 10^5$

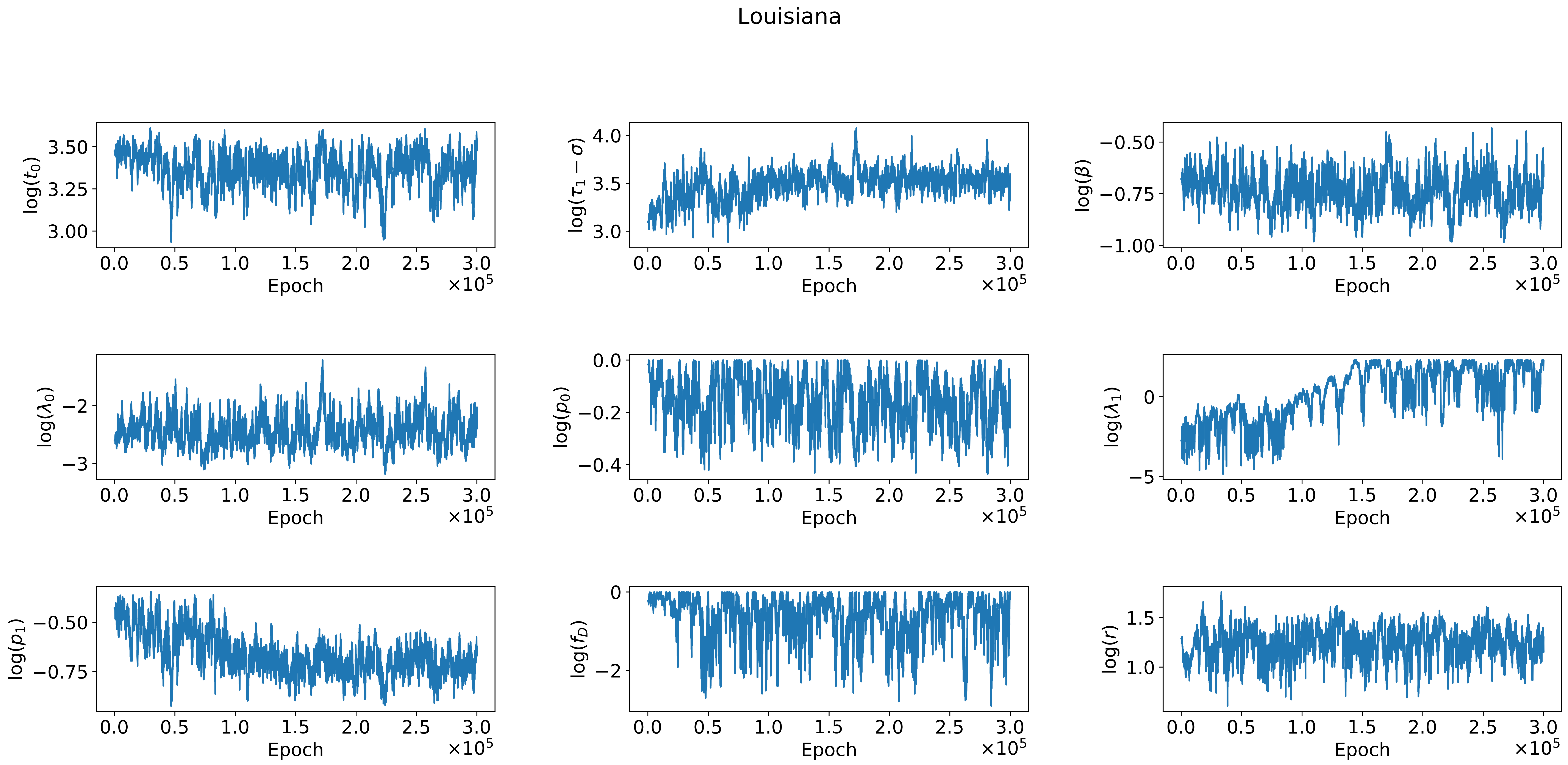

Louisiana
log(t_0)
log($\tau_1 - \sigma$)
log(β)
log(λ_0)
log(p_0)
log(λ_1)
log(p_1)
log(f_D)
log(r)
Epoch
$\times 10^5$

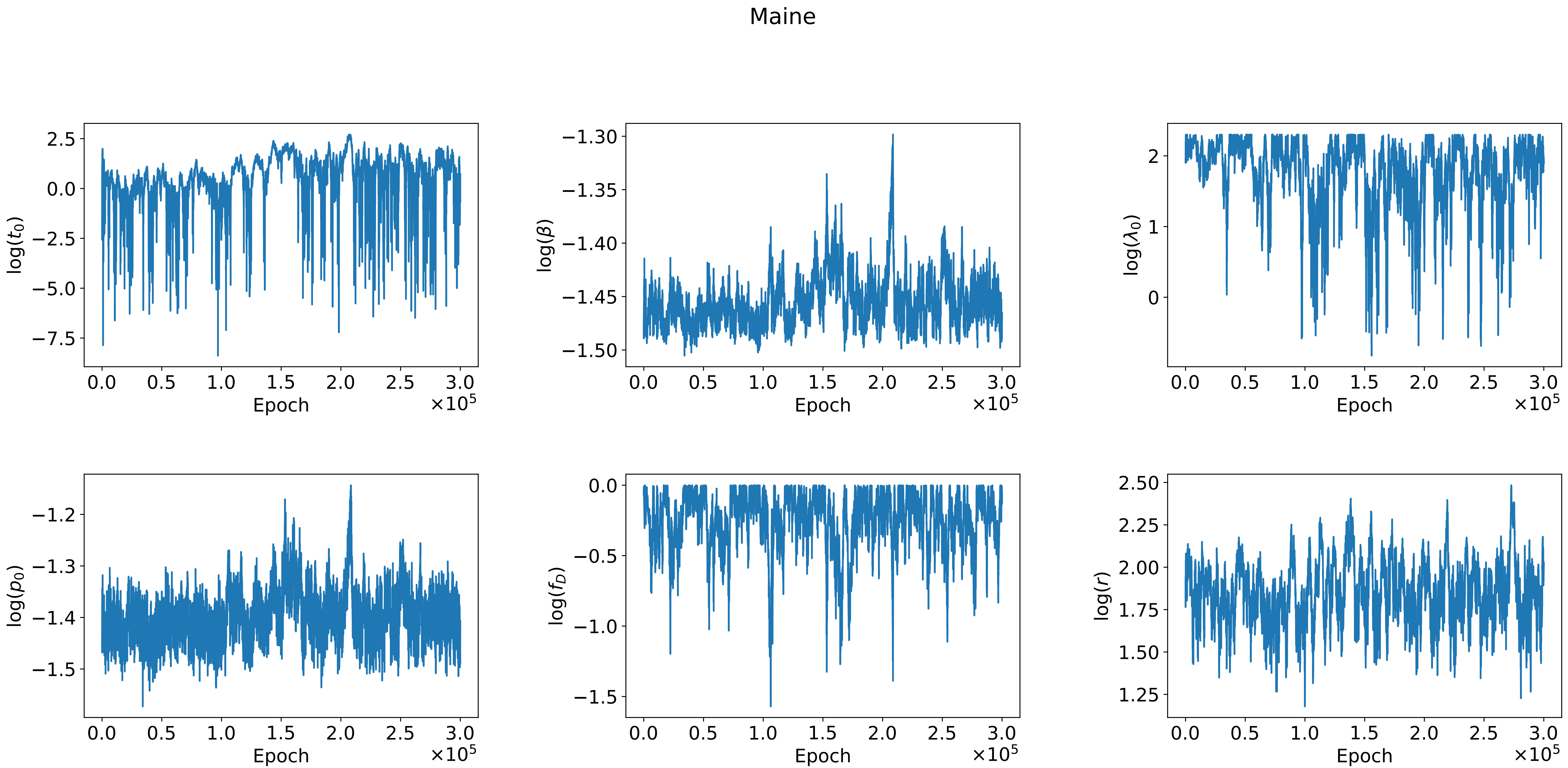

Maine
log(t_0)
log(β)
log(λ_0)
log(p_0)
log(f_D)
log(r)
Epoch
×10^5

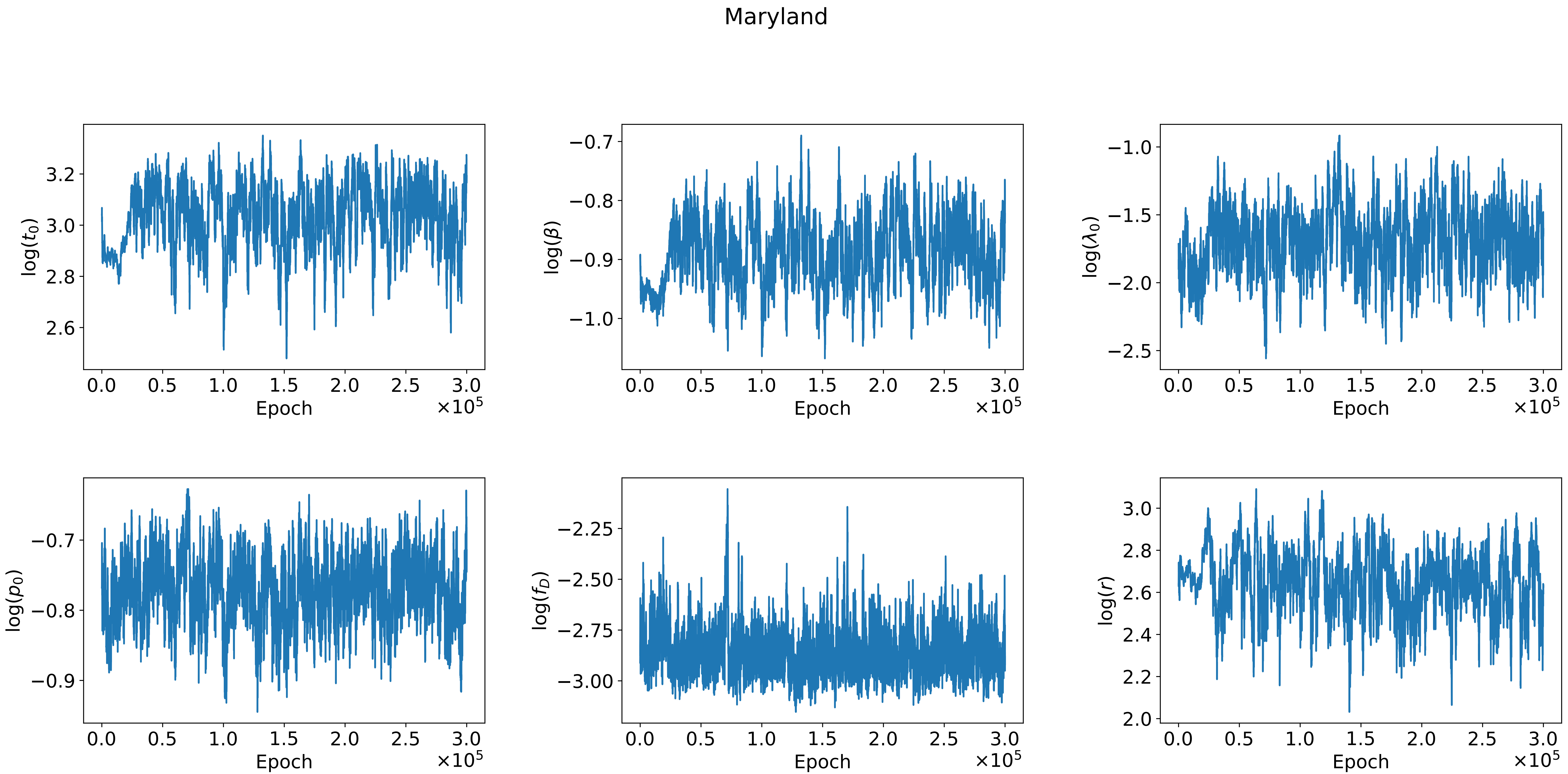
Maryland
log(t_0)
log(β)
log(λ_0)
log(p_0)
log(f_D)
log(r)
Epoch
×10^5

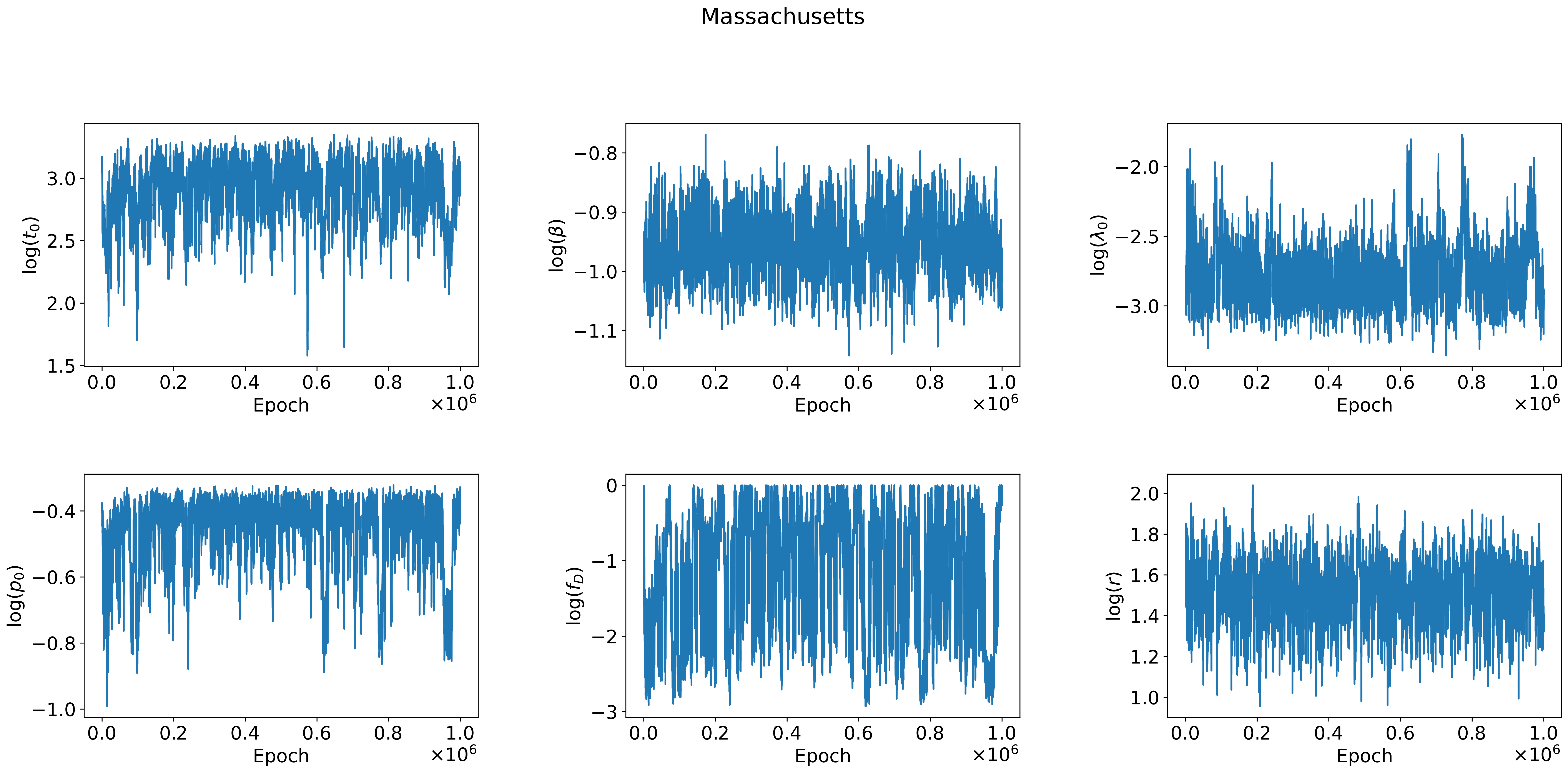

Massachusetts
log(t_0)
log(β)
log(λ_0)
log(p_0)
log(f_D)
log(r)
Epoch
×10^6

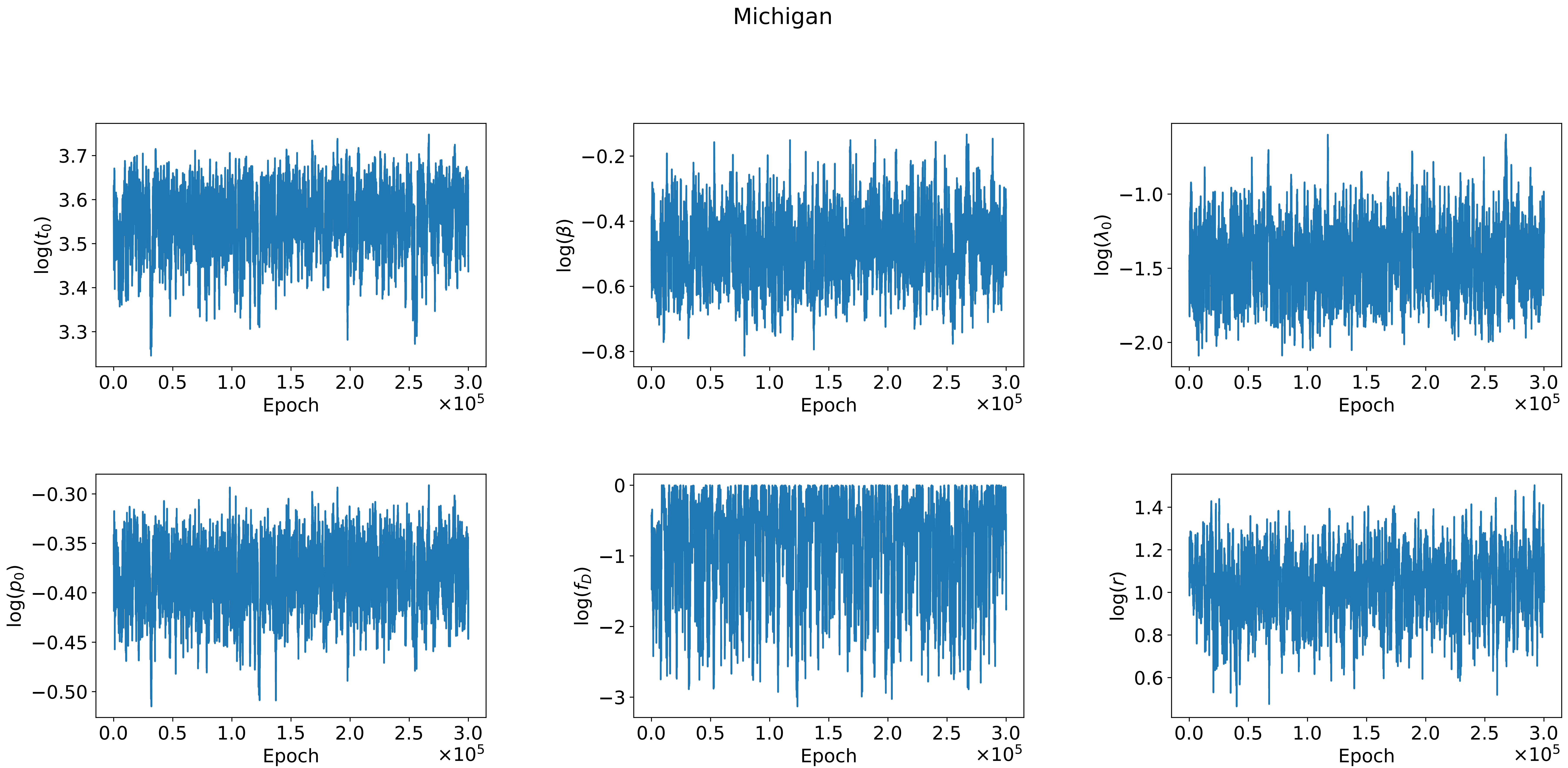

Michigan
log(t_0)
log(β)
log(λ_0)
log(p_0)
log(f_D)
log(r)
Epoch
$\times10^5$

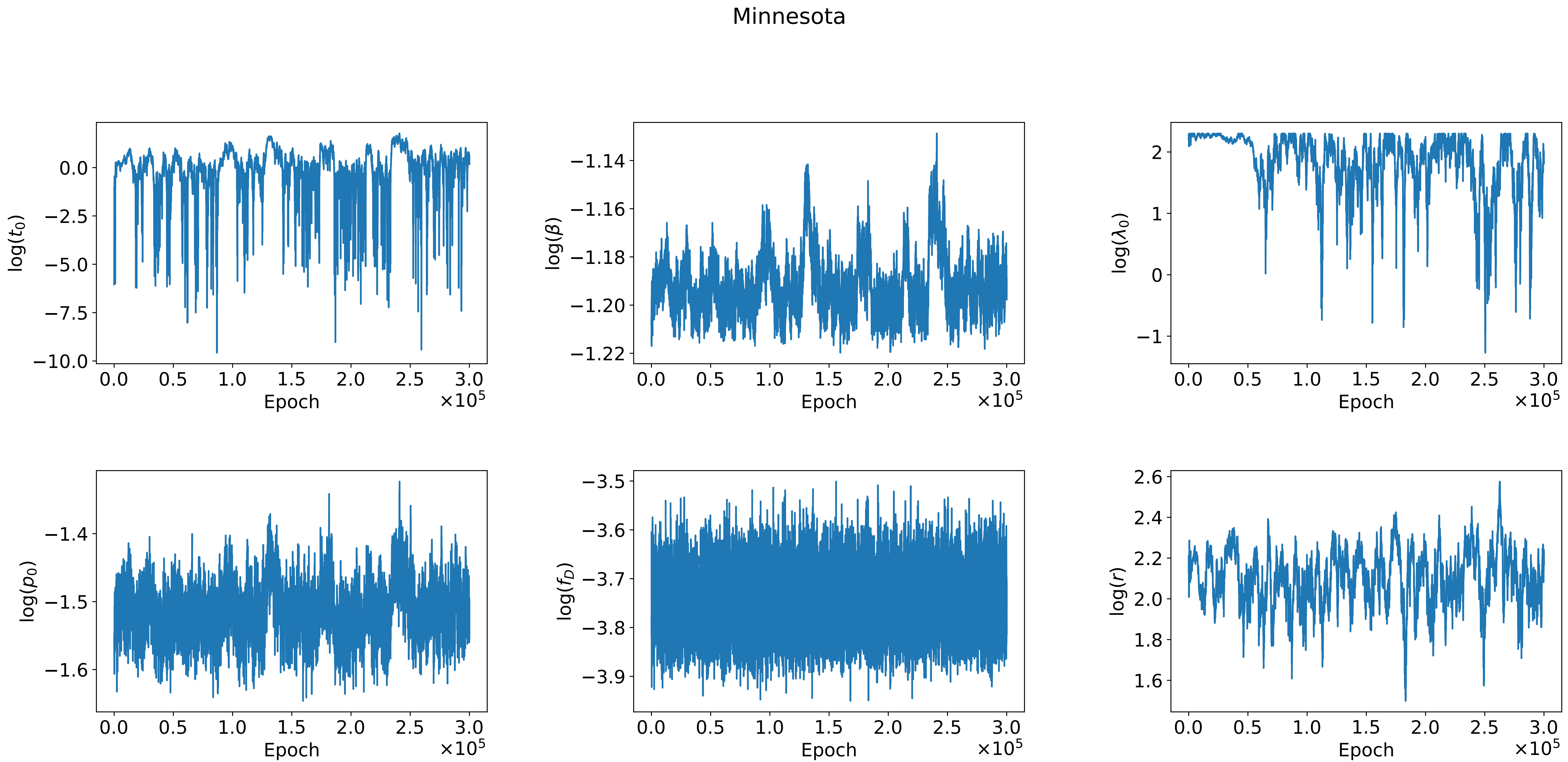

Minnesota
log(t_0)
log(β)
log(λ_0)
log(p_0)
log(f_D)
log(r)
Epoch
$\times 10^5$

Mississippi

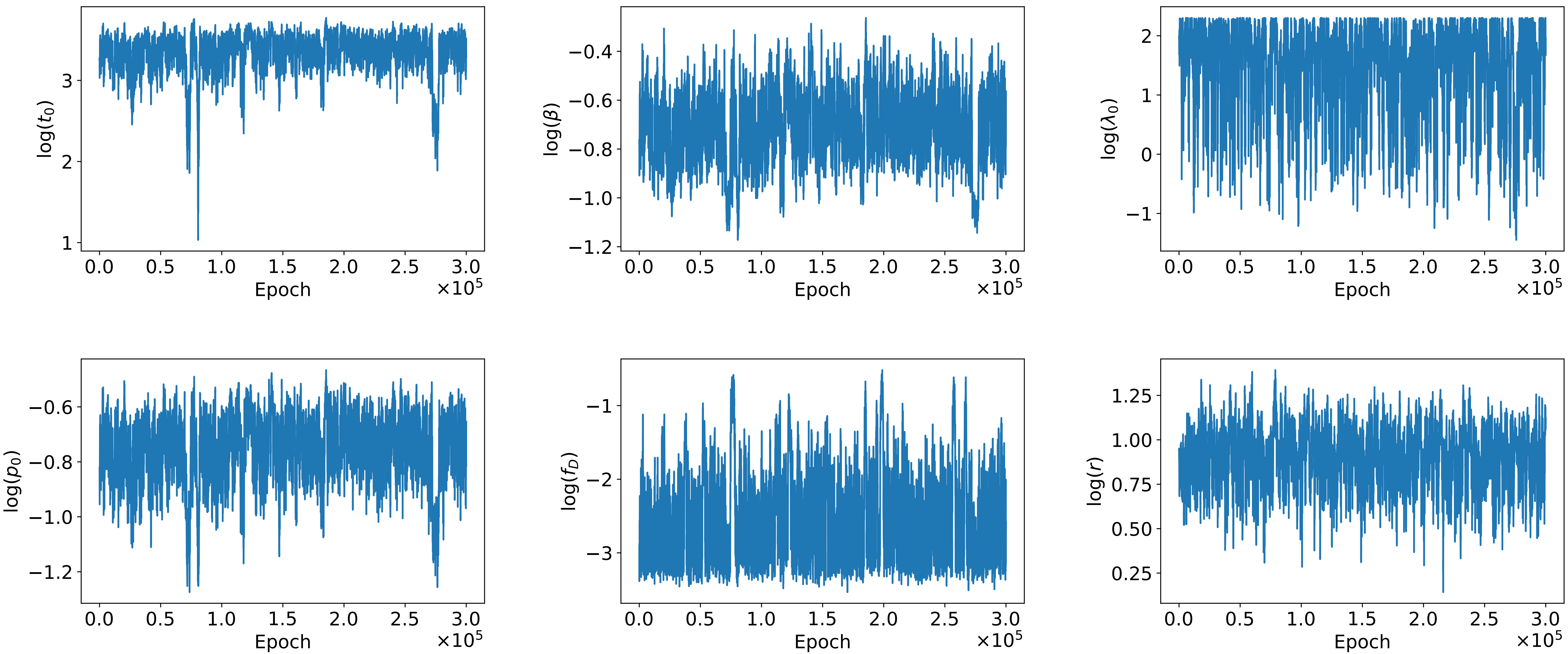

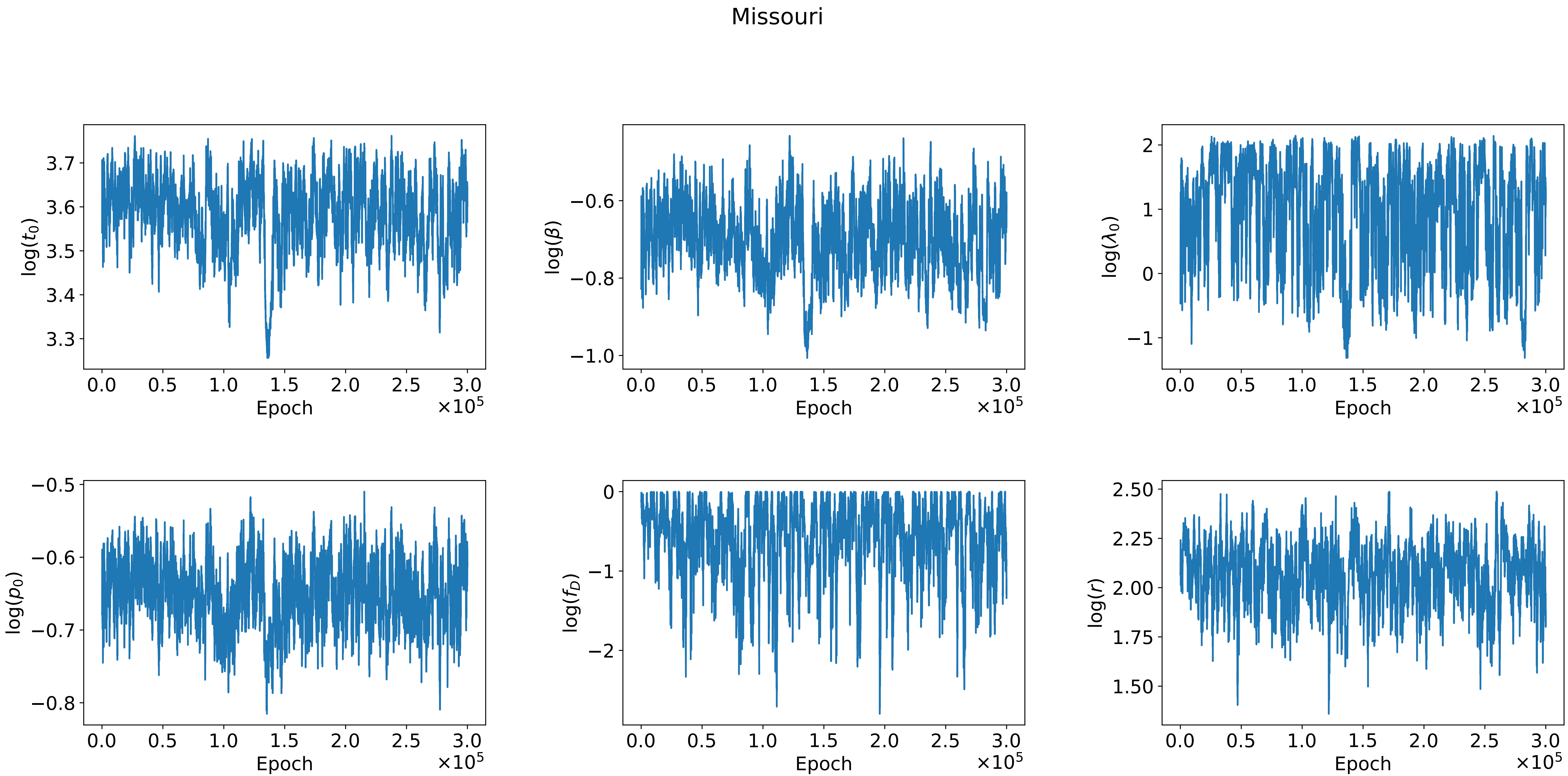

Missouri
log(t_0)
3.7
3.6
3.5
3.4
3.3
Epoch
0.0
0.5
1.0
1.5
2.0
2.5
3.0
×10^5
log(β)
−0.6
−0.8
−1.0
log(λ_0)
2
1
0
−1
log(p_0)
−0.5
−0.6
−0.7
−0.8
log(f_D)
0
−1
−2
log(r)
2.50
2.25
2.00
1.75
1.50

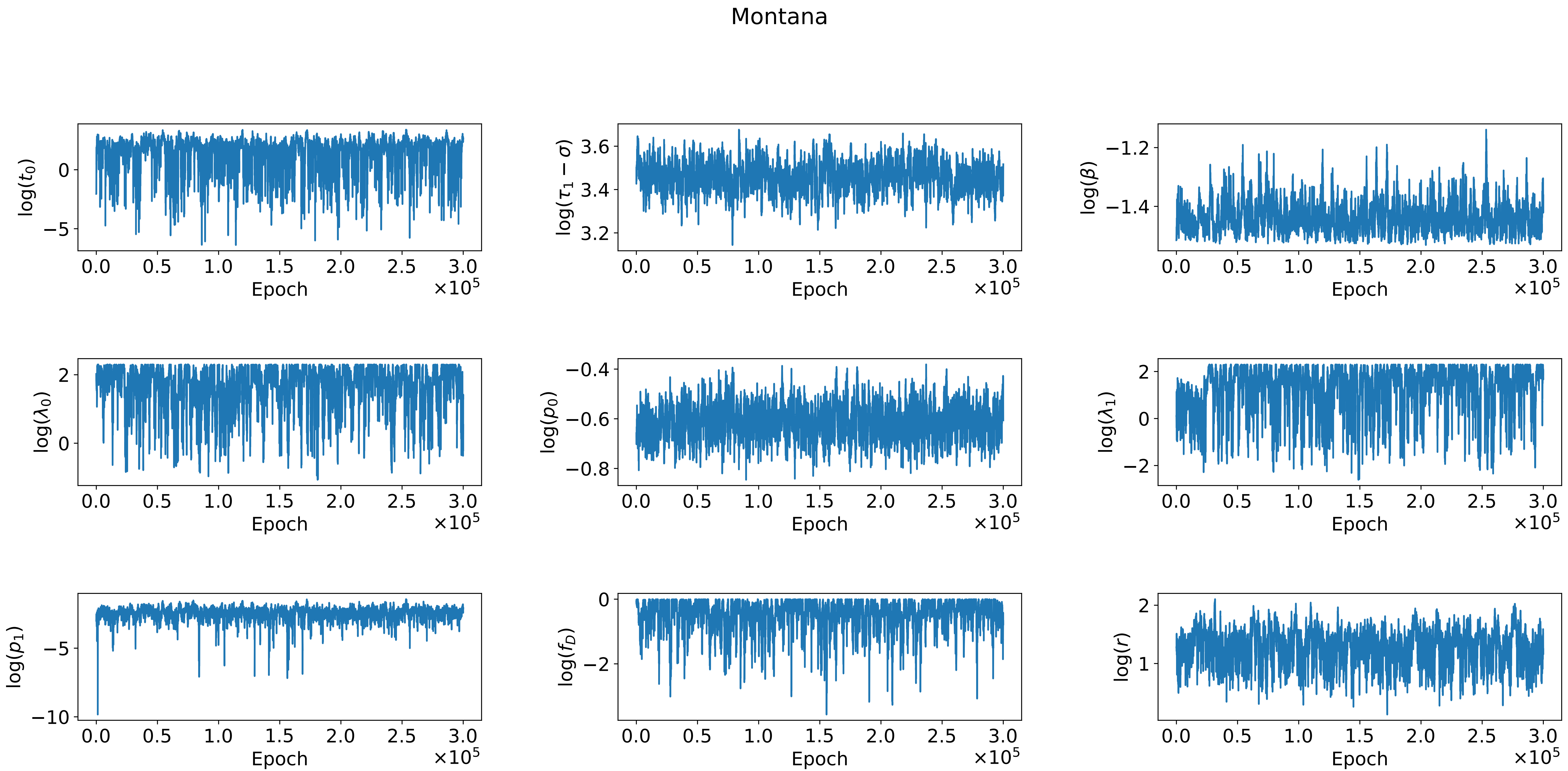
Montana
log(t0)
log(τ1 − σ)
log(β)
log(λ0)
log(p0)
log(λ1)
log(p1)
log(fD)
log(r)
Epoch
×10^5

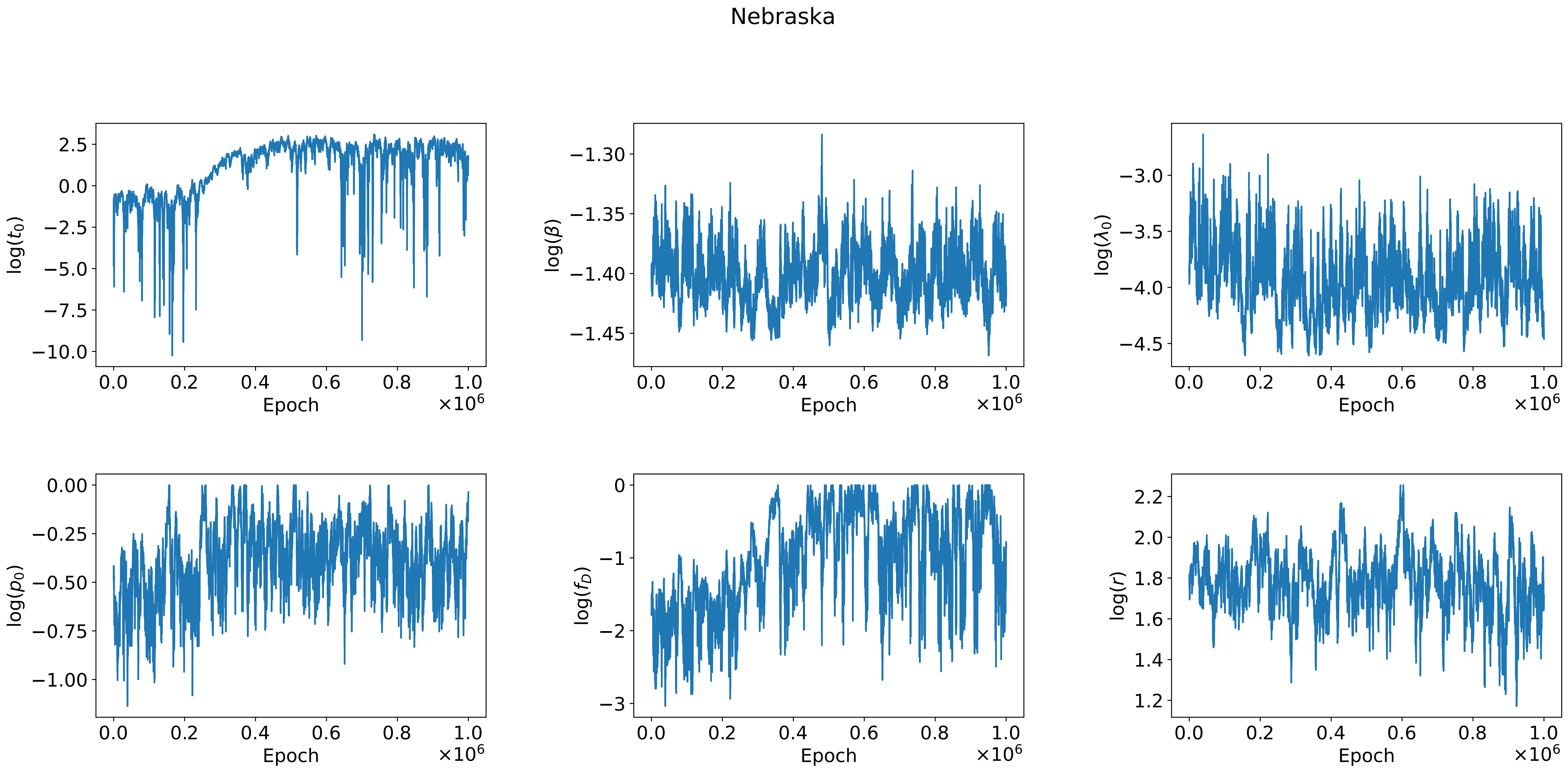
Nebraska
log(t_0)
log(β)
log(λ_0)
log(p_0)
log(f_D)
log(r)
Epoch
$\times10^6$
0.0
0.2
0.4
0.6
0.8
1.0
2.5
0.0
−2.5
−5.0
−7.5
−10.0
−1.30
−1.35
−1.40
−1.45
−3.0
−3.5
−4.0
−4.5
0.00
−0.25
−0.50
−0.75
−1.00
0
−1
−2
−3
2.2
2.0
1.8
1.6
1.4
1.2

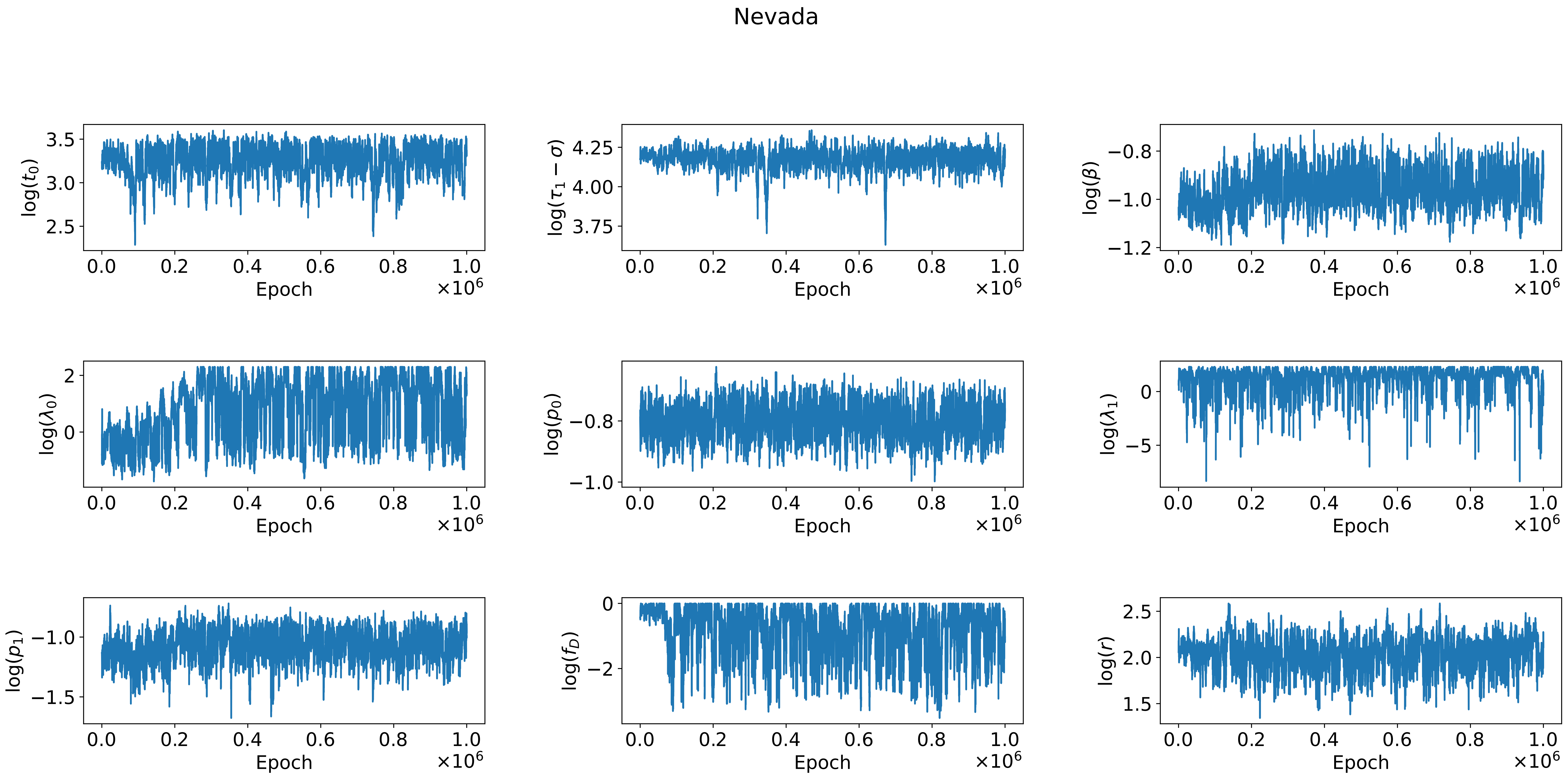
Nevada
log(t_0)
log($\tau_1 - \sigma$)
log(β)
log(λ_0)
log(p_0)
log(λ_1)
log(p_1)
log(f_D)
log(r)
Epoch
×10^6

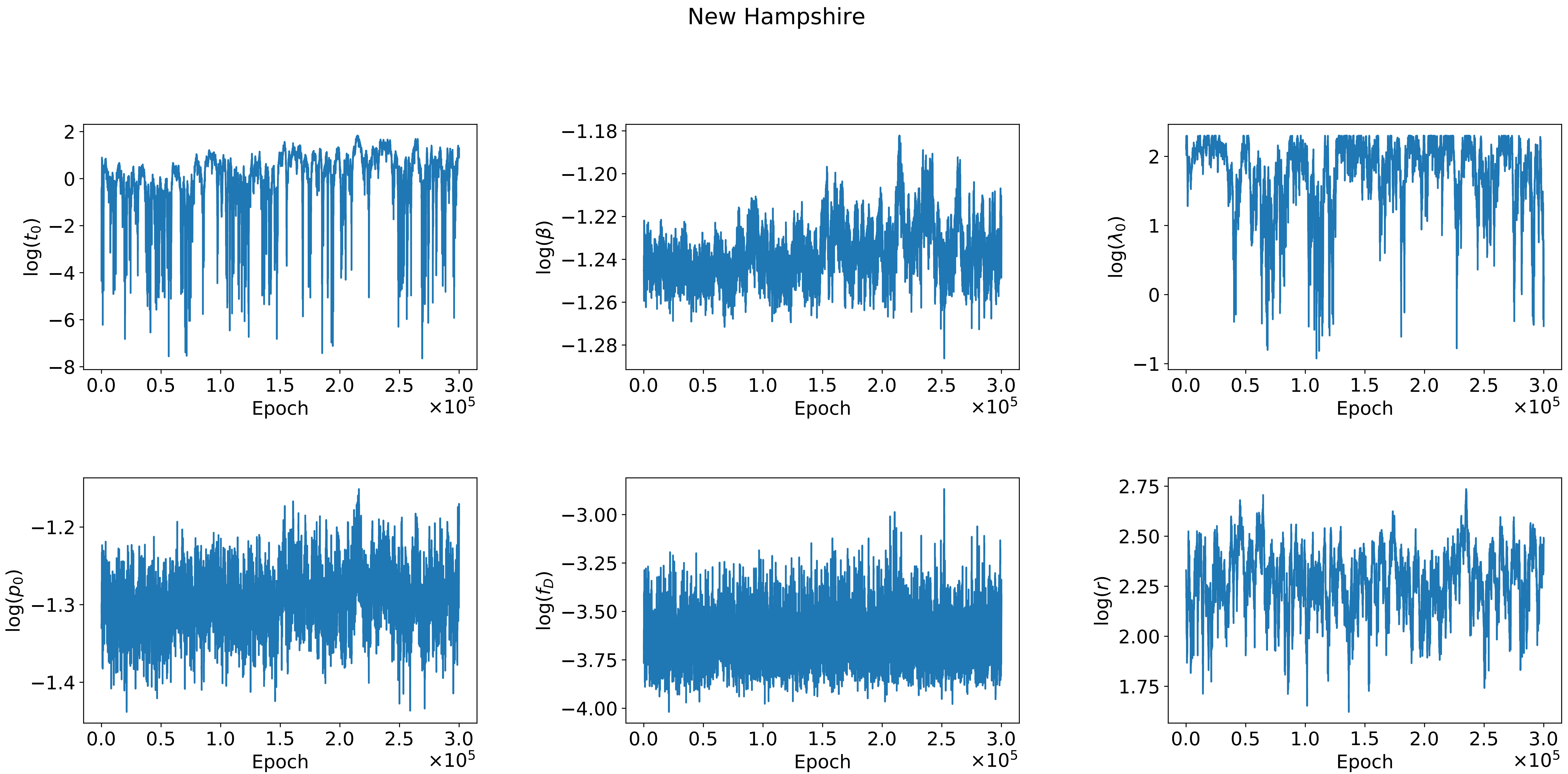
New Hampshire
log(t_0)
log(β)
log(λ_0)
log(p_0)
log(f_D)
log(r)
Epoch
$\times 10^5$

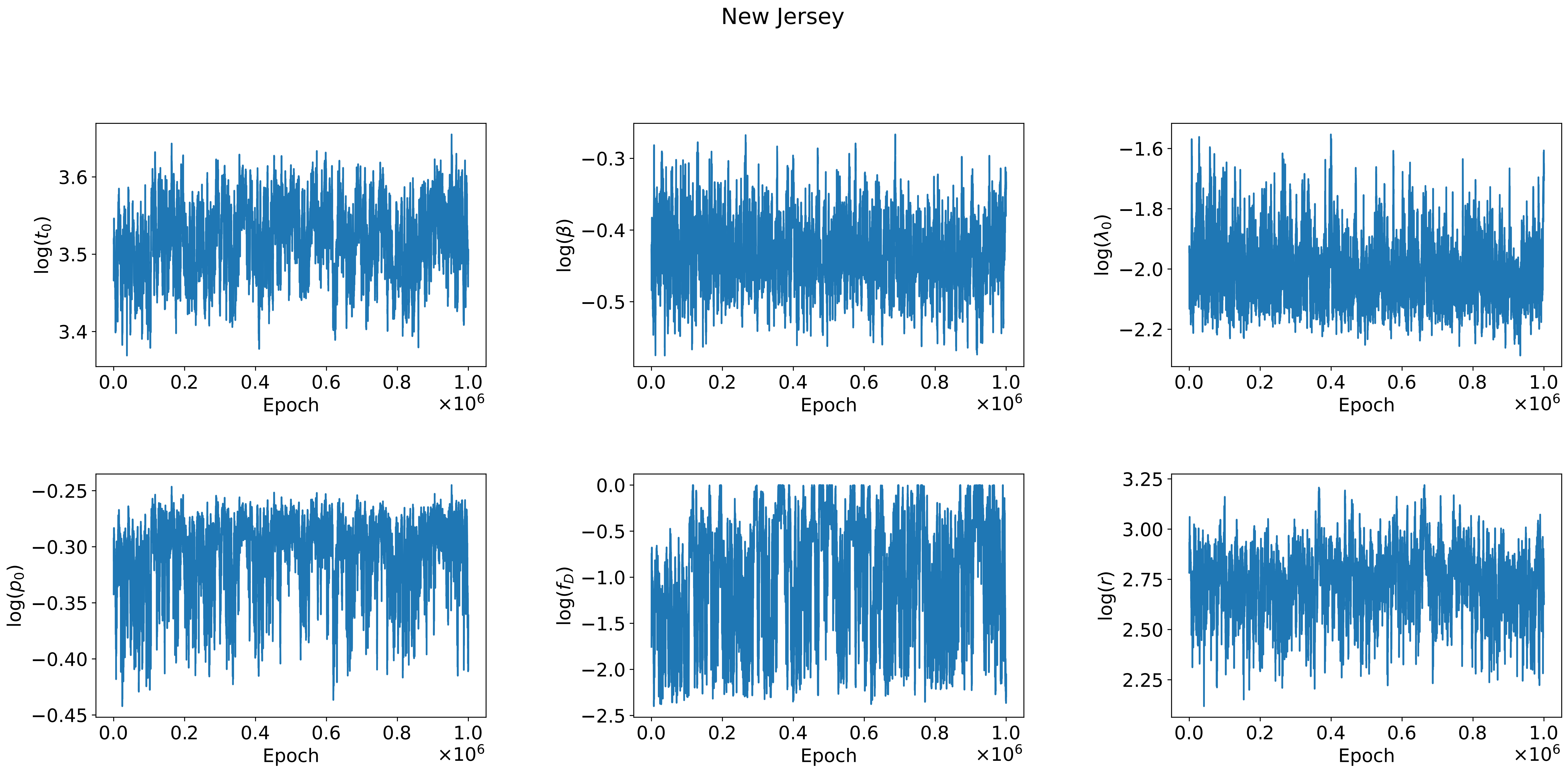
New Jersey
$\log(t_0)$
$\log(\beta)$
$\log(\lambda_0)$
$\log(p_0)$
$\log(f_D)$
$\log(r)$
Epoch
$\times 10^6$

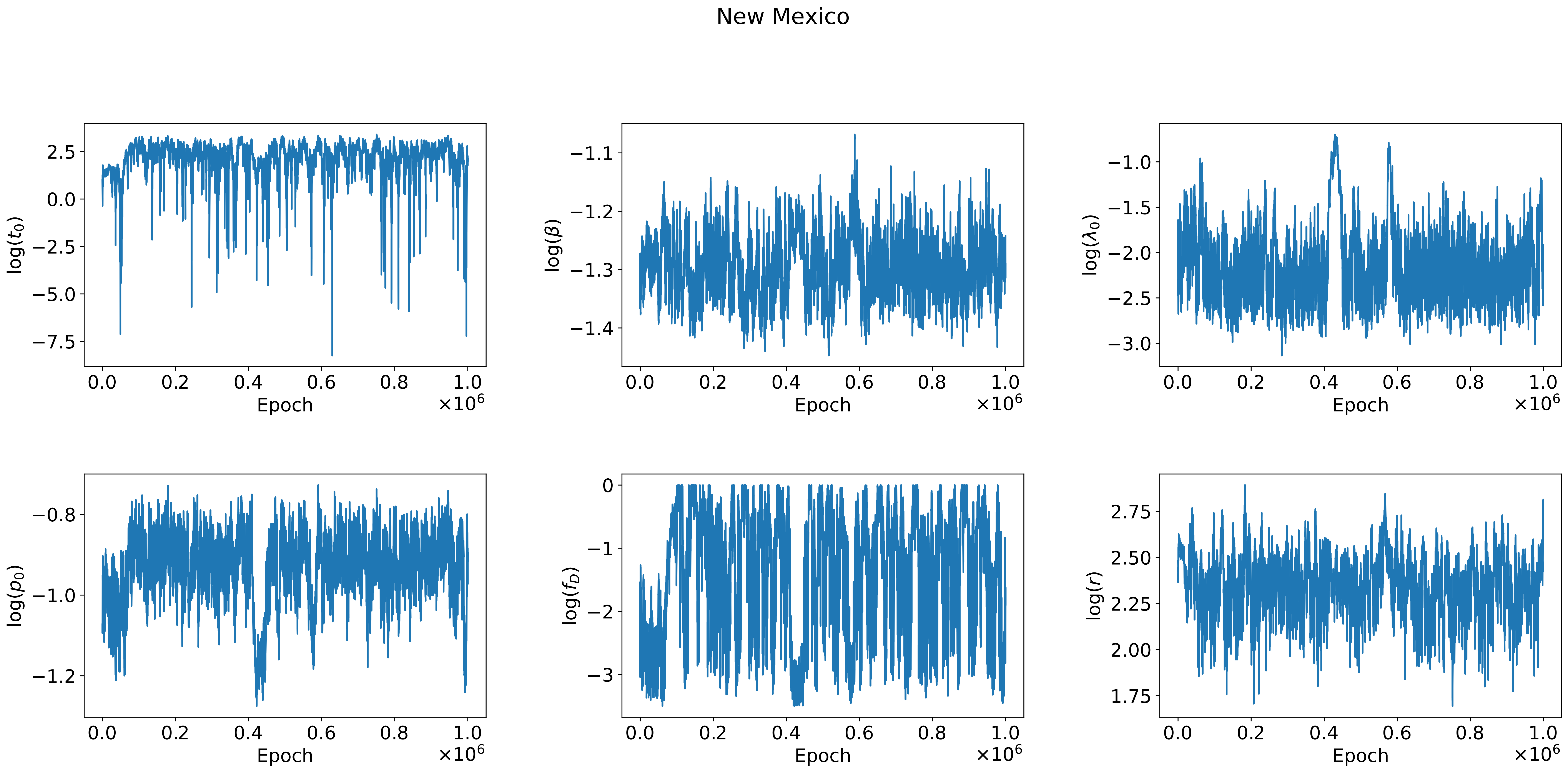
New Mexico
log(t_0)
log(β)
log(λ_0)
log(p_0)
log(f_D)
log(r)
Epoch
$\times 10^6$
2.5
0.0
−2.5
−5.0
−7.5
−1.1
−1.2
−1.3
−1.4
−1.0
−1.5
−2.0
−2.5
−3.0
−0.8
−1.0
−1.2
0
−1
−2
−3
2.75
2.50
2.25
2.00
1.75
0.0
0.2
0.4
0.6
0.8
1.0

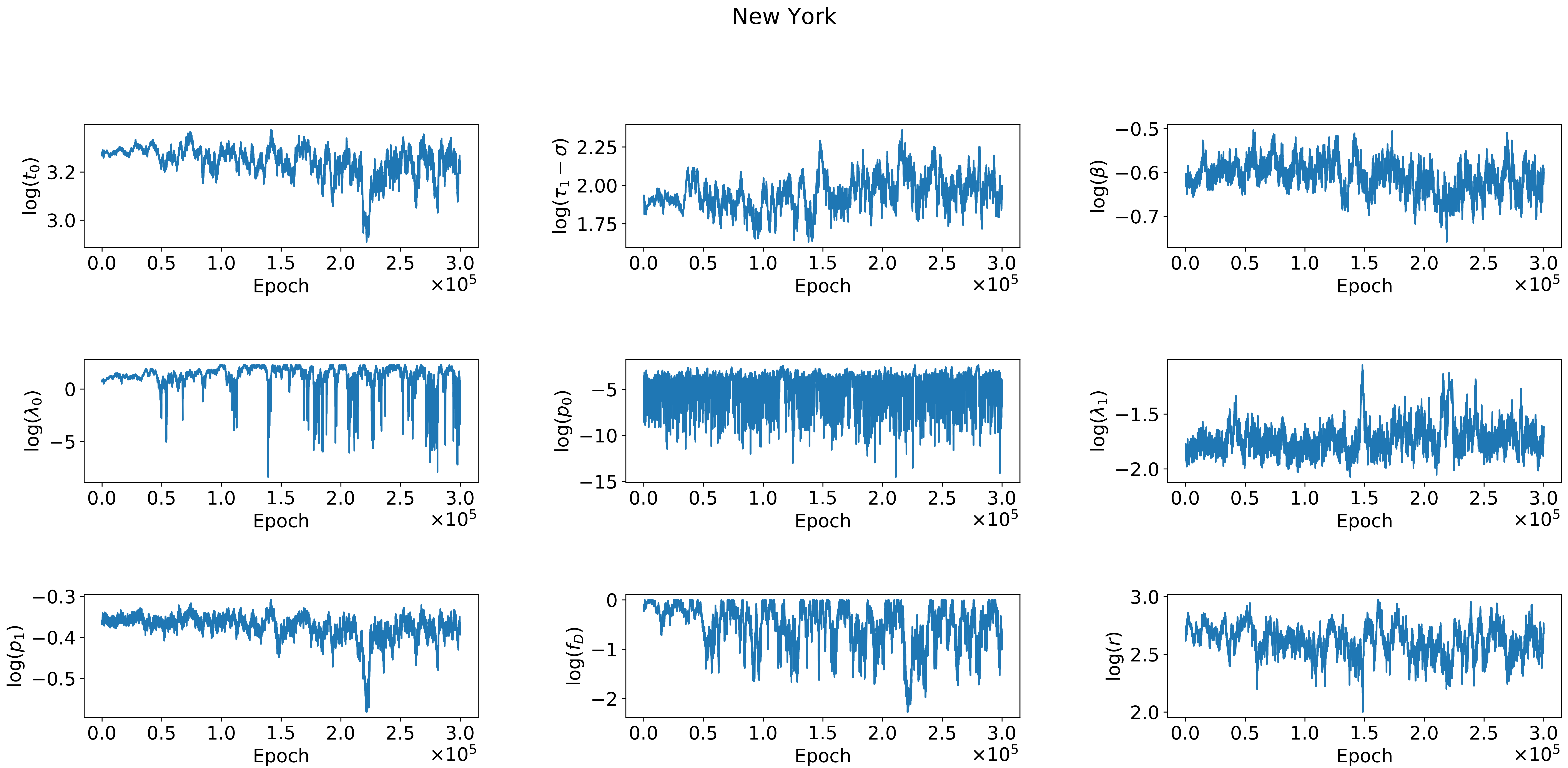
New York
log(t_0)
log($\tau_1-\sigma$)
log(β)
log(λ_0)
log(p_0)
log(λ_1)
log(p_1)
log(f_D)
log(r)
Epoch
$\times 10^5$

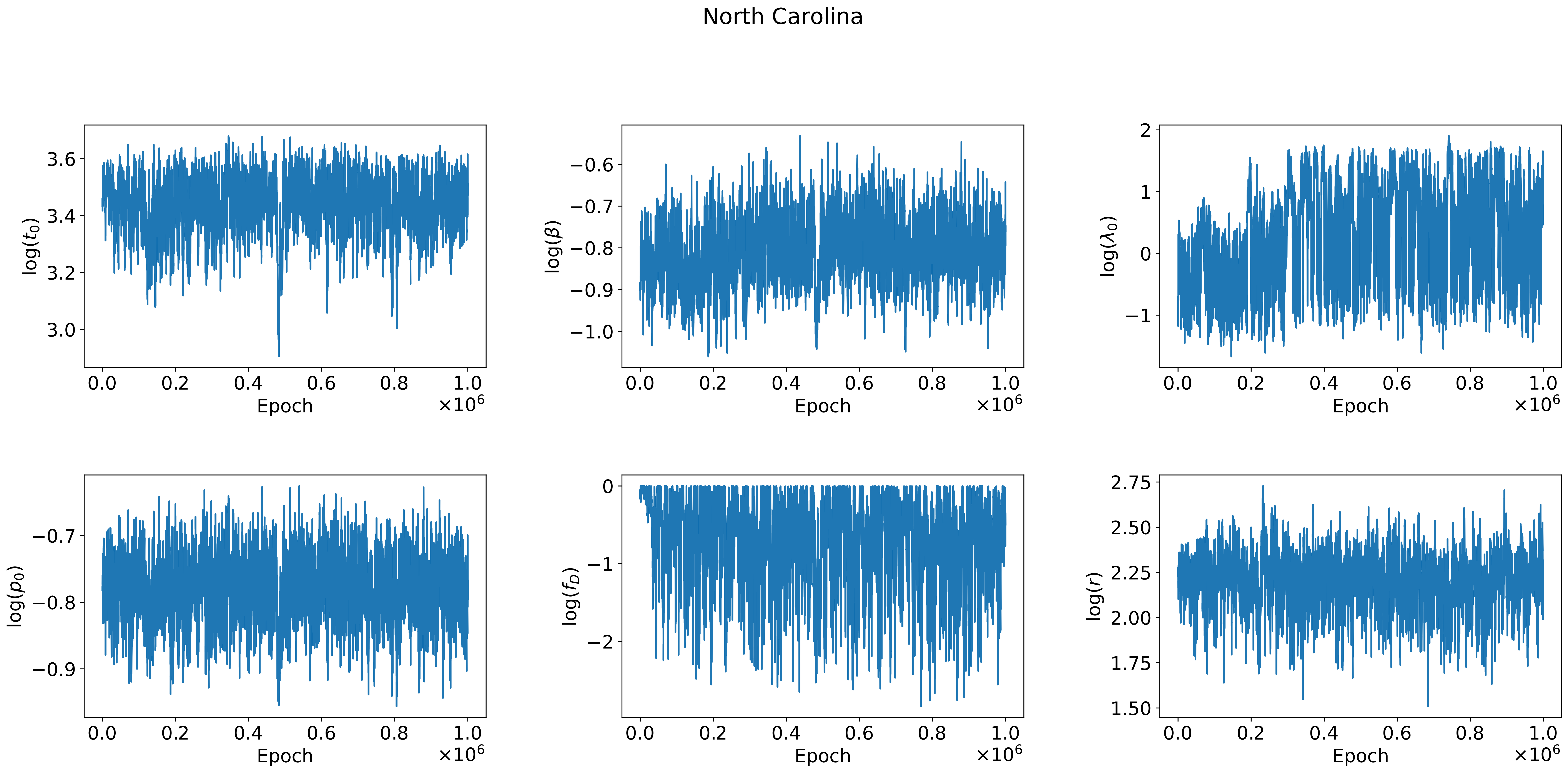
North Carolina
log(t_0)
log(β)
log(λ_0)
log(p_0)
log(f_D)
log(r)
Epoch
×10^6

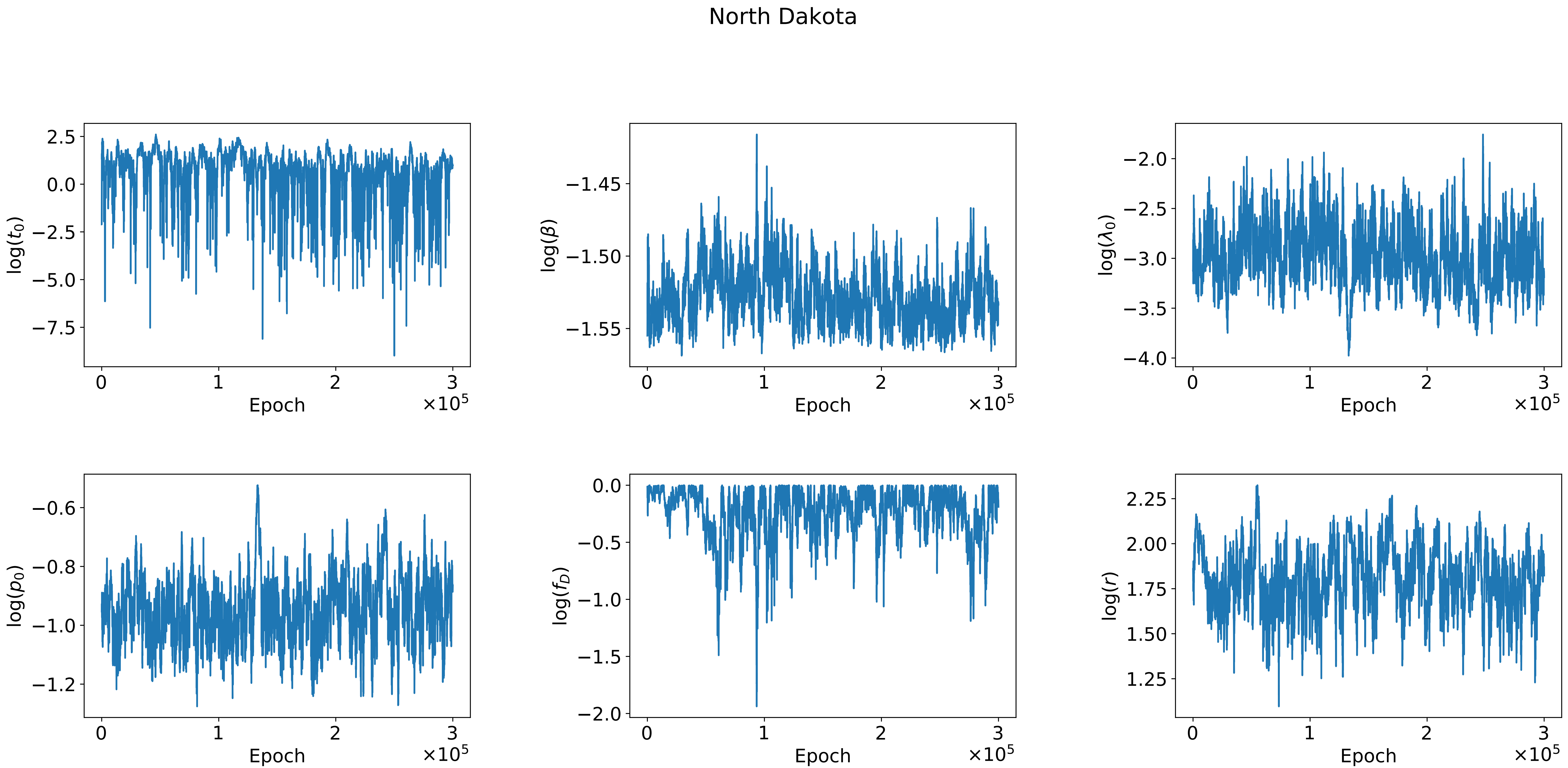

North Dakota
log(t_0)
log(β)
log(λ_0)
log(p_0)
log(f_D)
log(r)
Epoch
$\times 10^5$

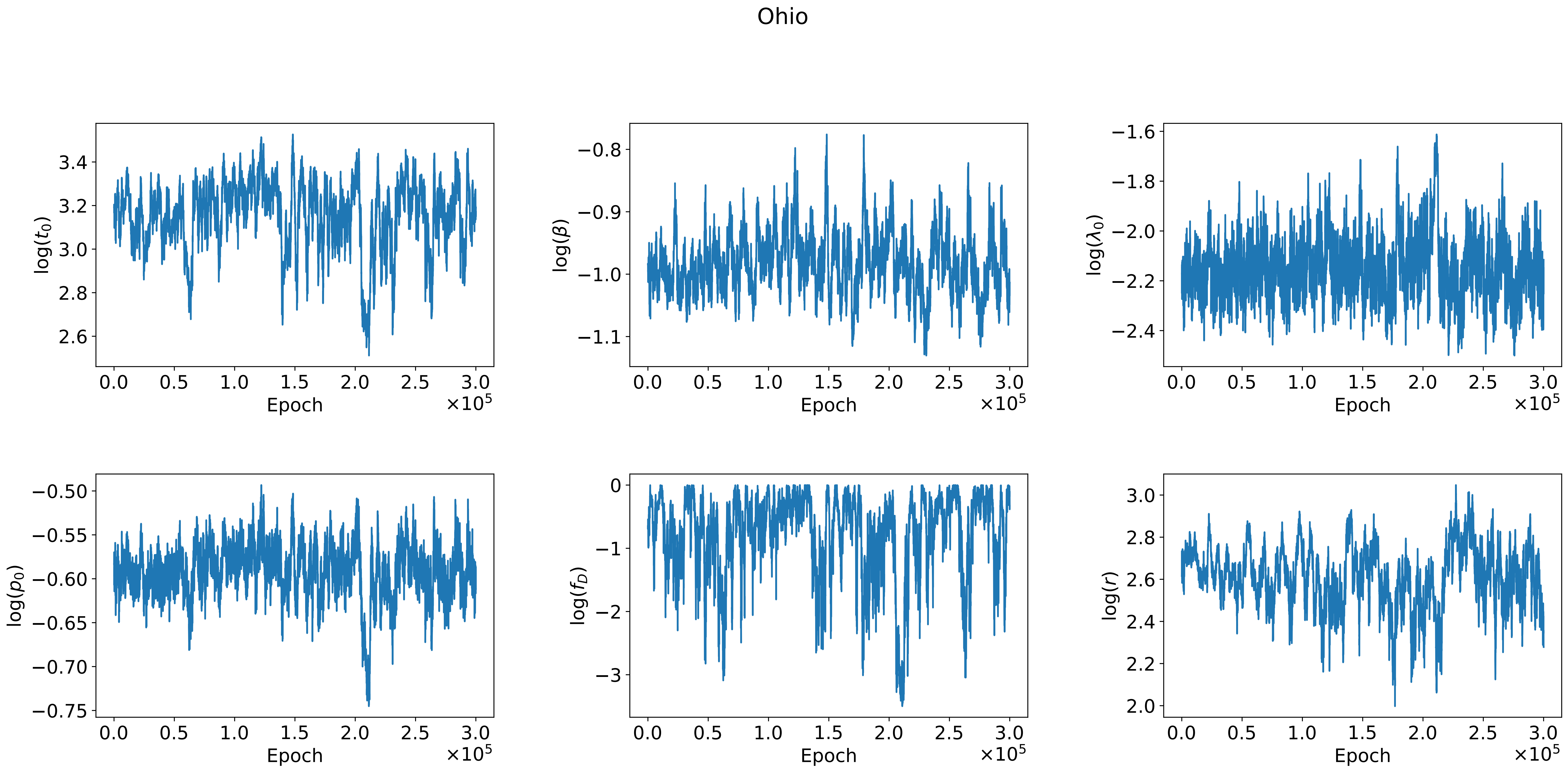

Ohio
log(t_0)
log(β)
log(λ_0)
log(p_0)
log(f_D)
log(r)
Epoch
$\times 10^5$

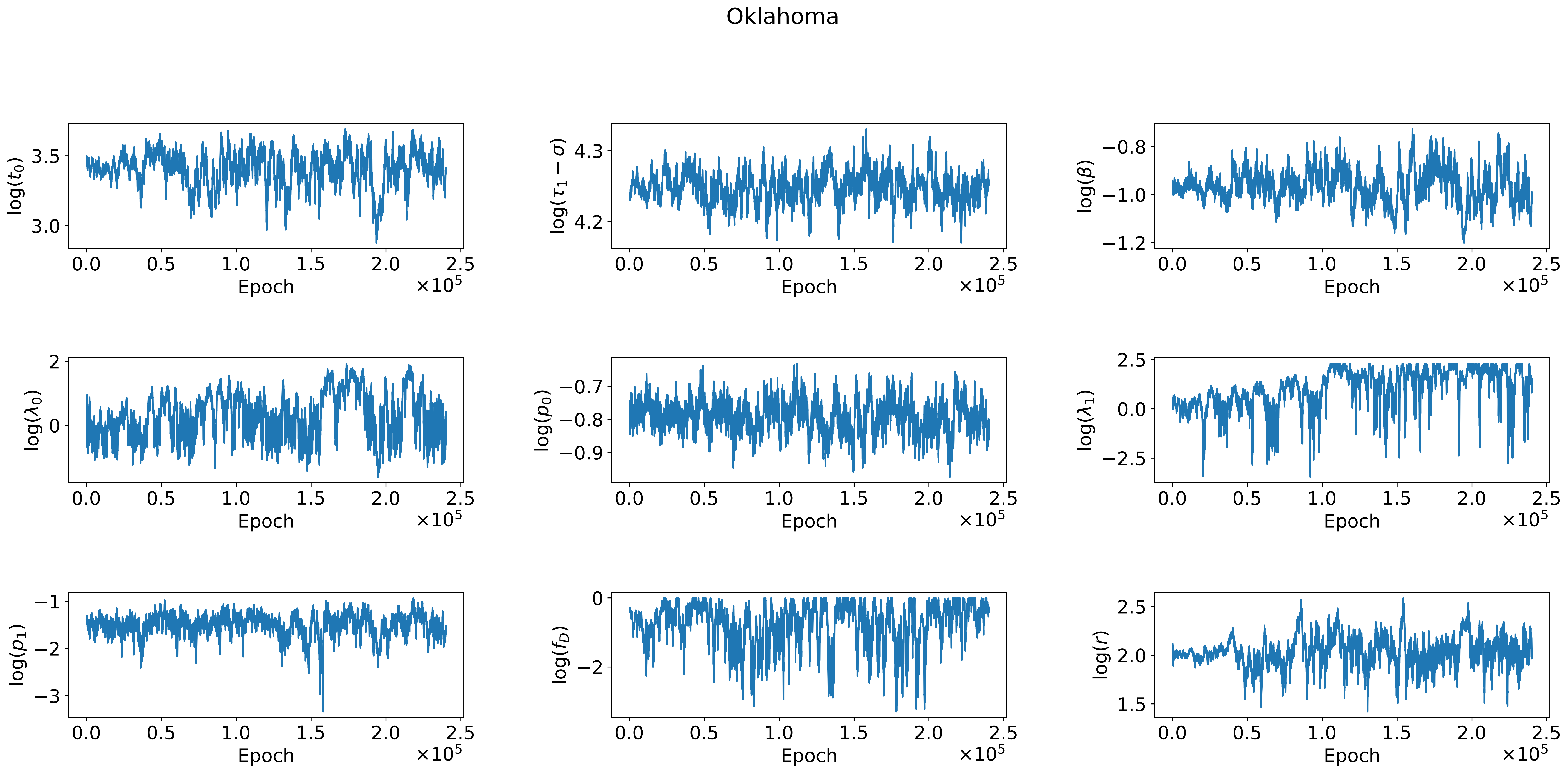

Oklahoma
$\log(t_0)$
$\log(\tau_1-\sigma)$
$\log(\beta)$
$\log(\lambda_0)$
$\log(p_0)$
$\log(\lambda_1)$
$\log(p_1)$
$\log(f_D)$
$\log(r)$
Epoch
$\times10^5$

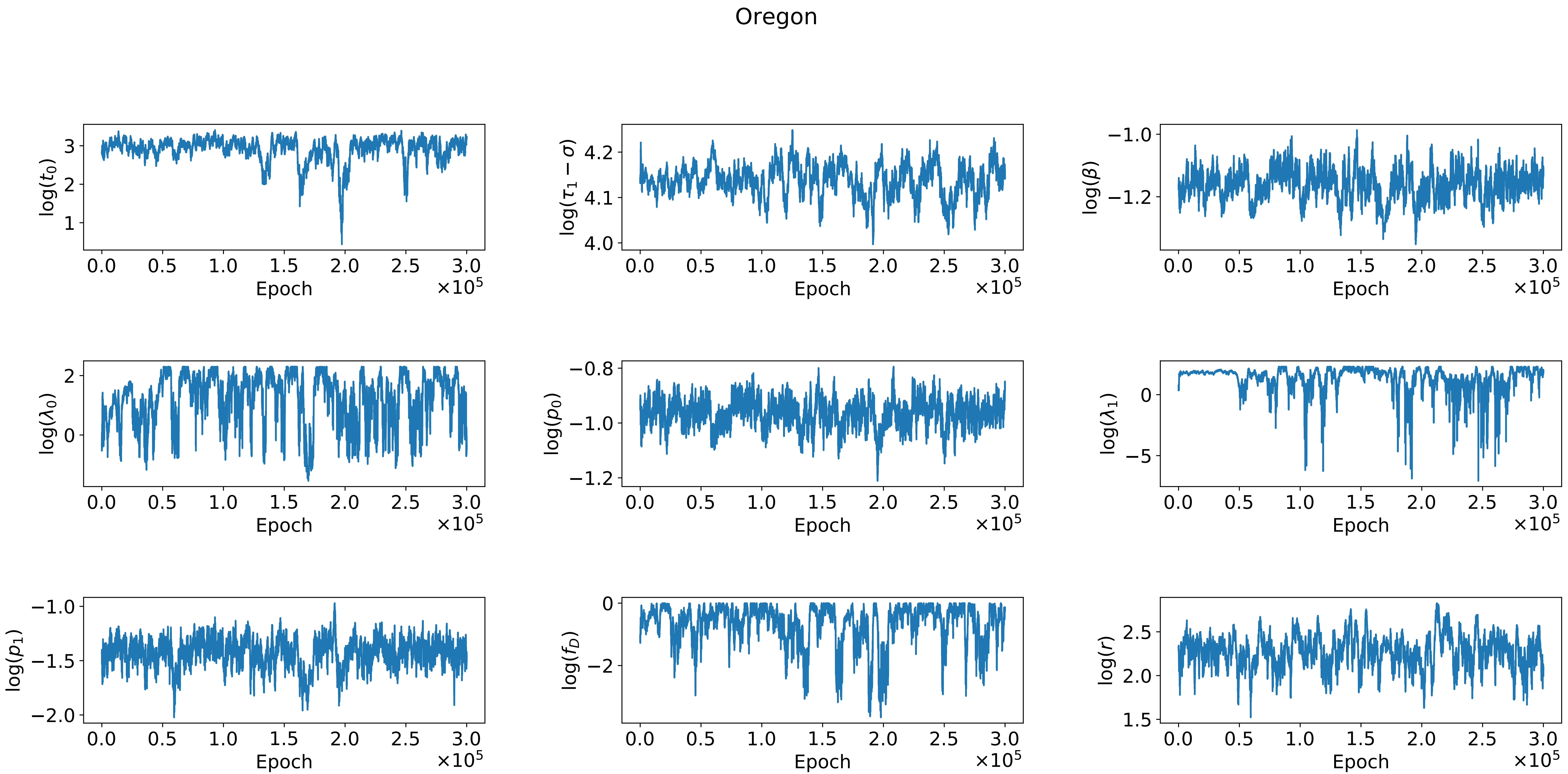
Oregon
log(t_0)
log($\tau_1 - \sigma$)
log(β)
log(λ_0)
log(p_0)
log(λ_1)
log(p_1)
log(f_D)
log(r)
Epoch
0.0 0.5 1.0 1.5 2.0 2.5 3.0
$\times 10^5$

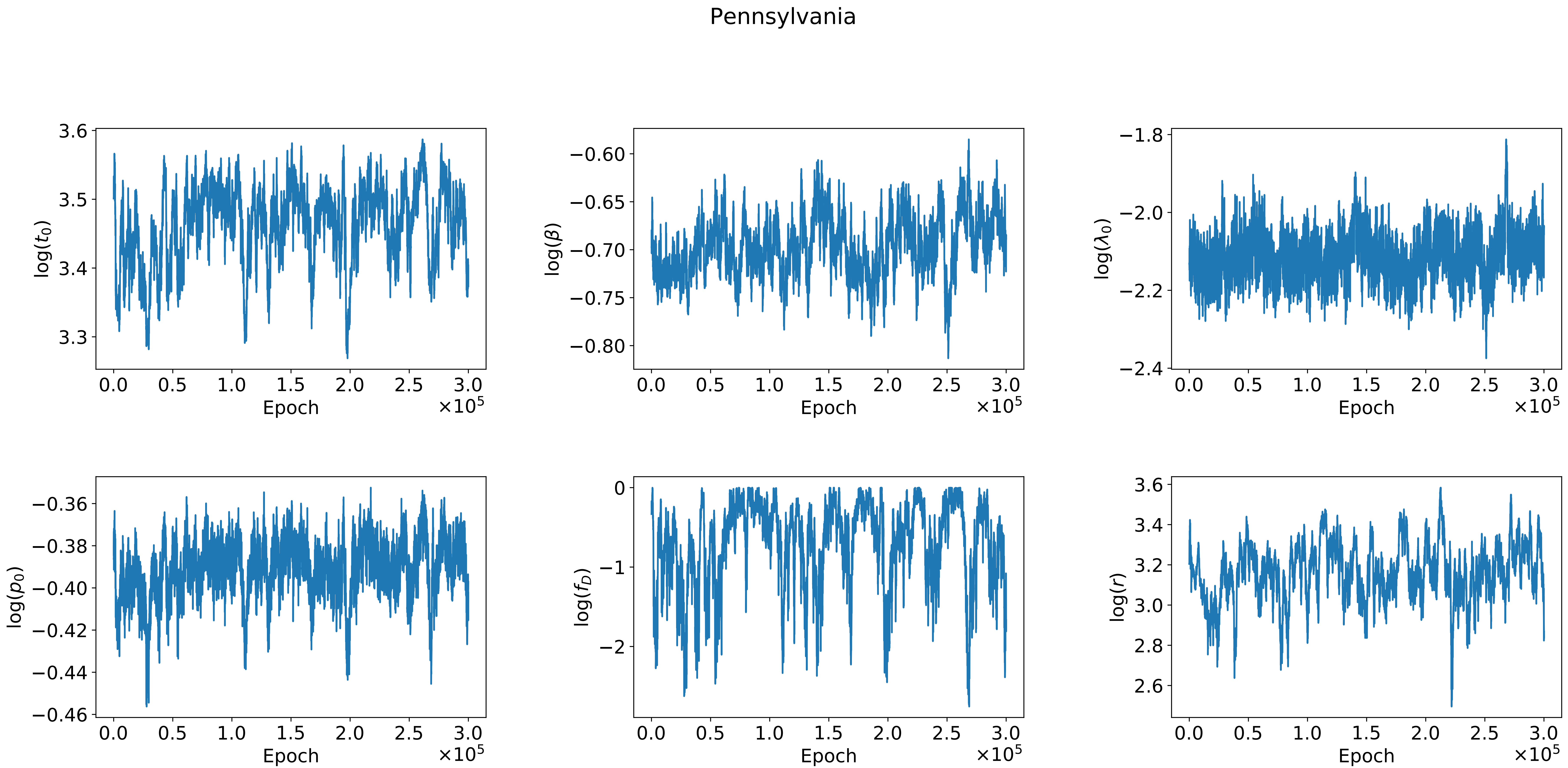
Pennsylvania
log(t_0)
log(β)
log(λ_0)
log(p_0)
log(f_D)
log(r)
Epoch
×10^5

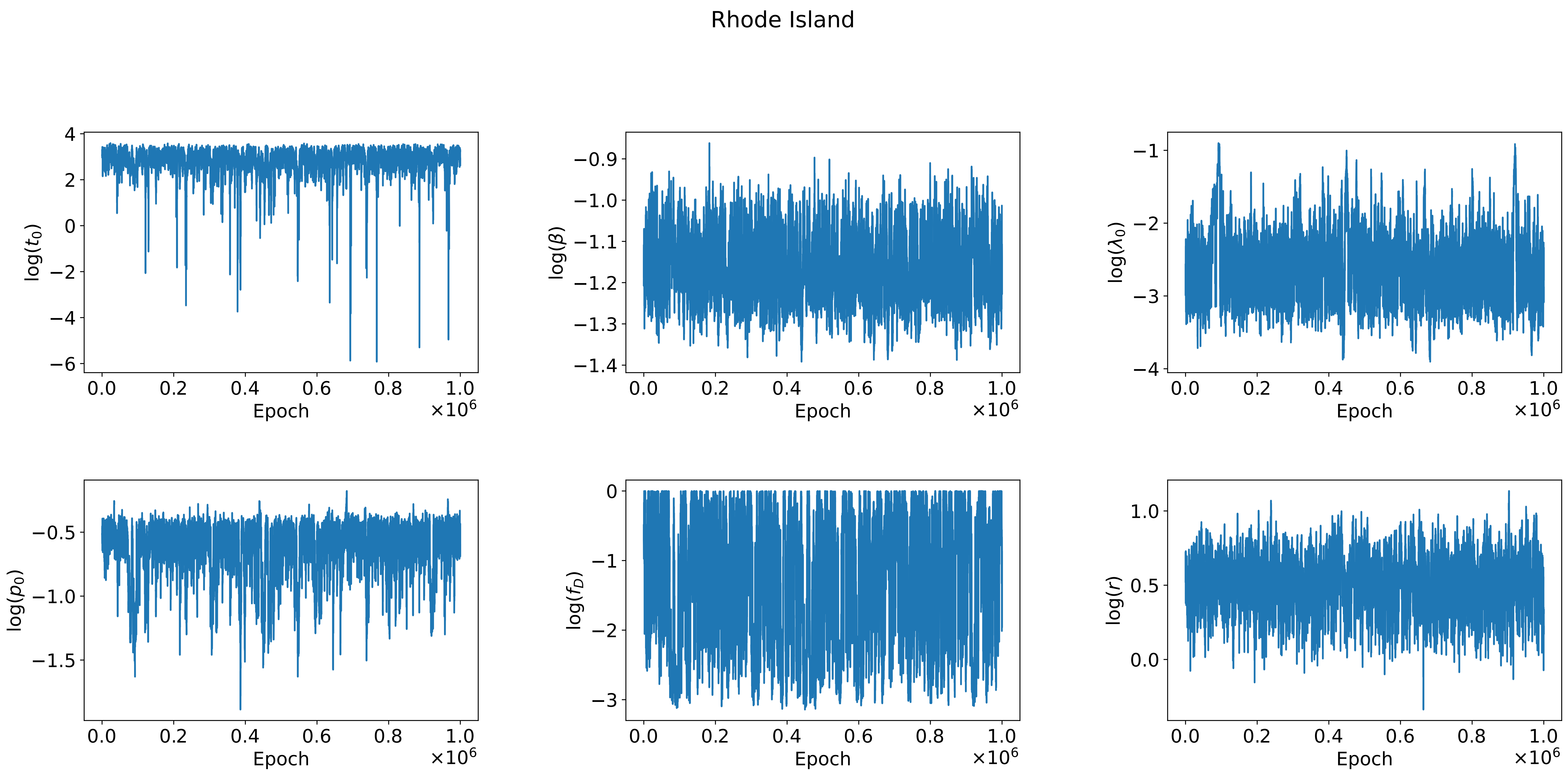

Rhode Island
log(t_0)
log(β)
log(λ_0)
log(p_0)
log(f_D)
log(r)
Epoch
×10^6

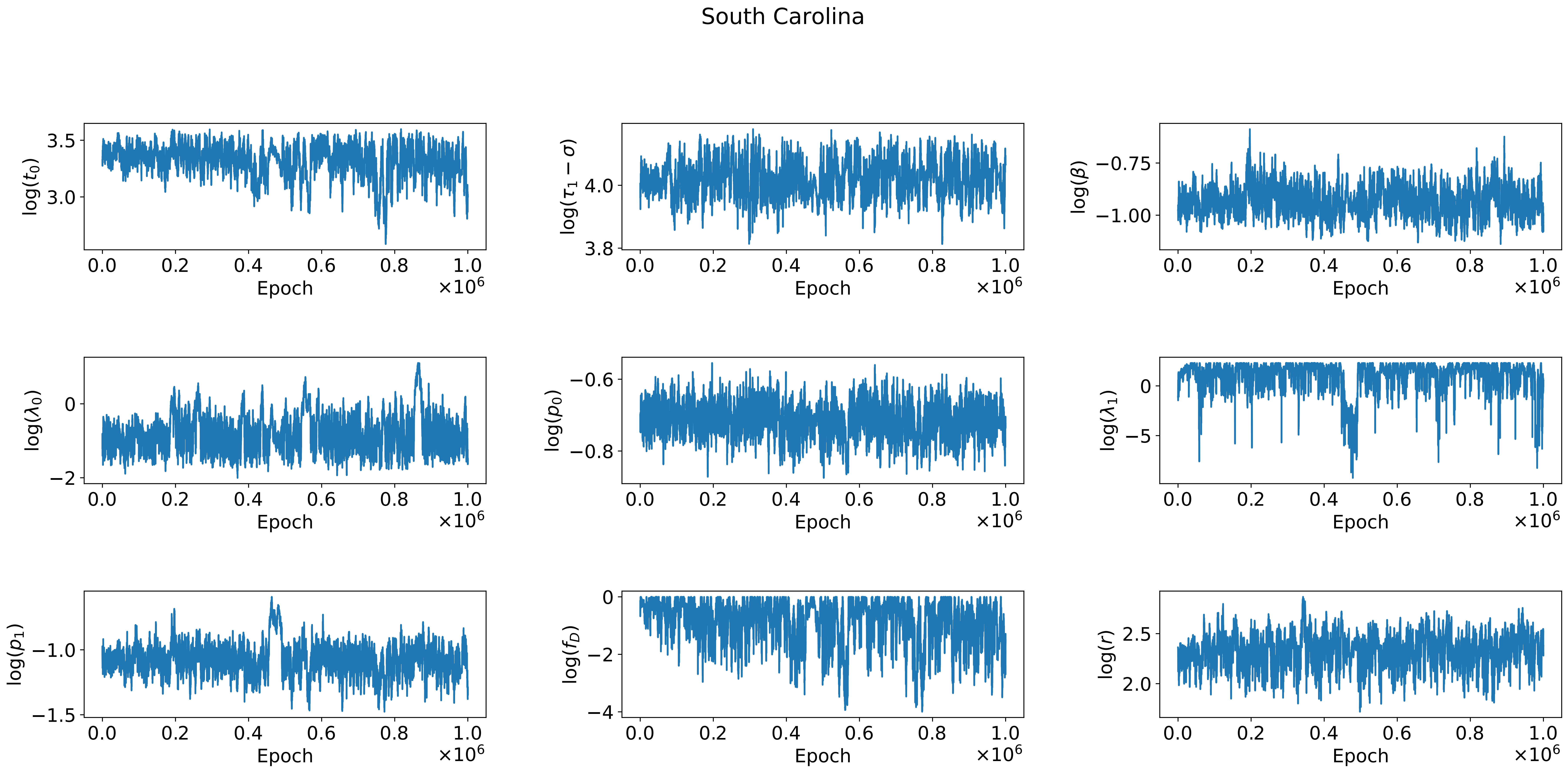

South Carolina
log(t_0)
log($\tau_1 - \sigma$)
log(β)
log(λ_0)
log(p_0)
log(λ_1)
log(p_1)
log(f_D)
log(r)
Epoch
$\times 10^6$

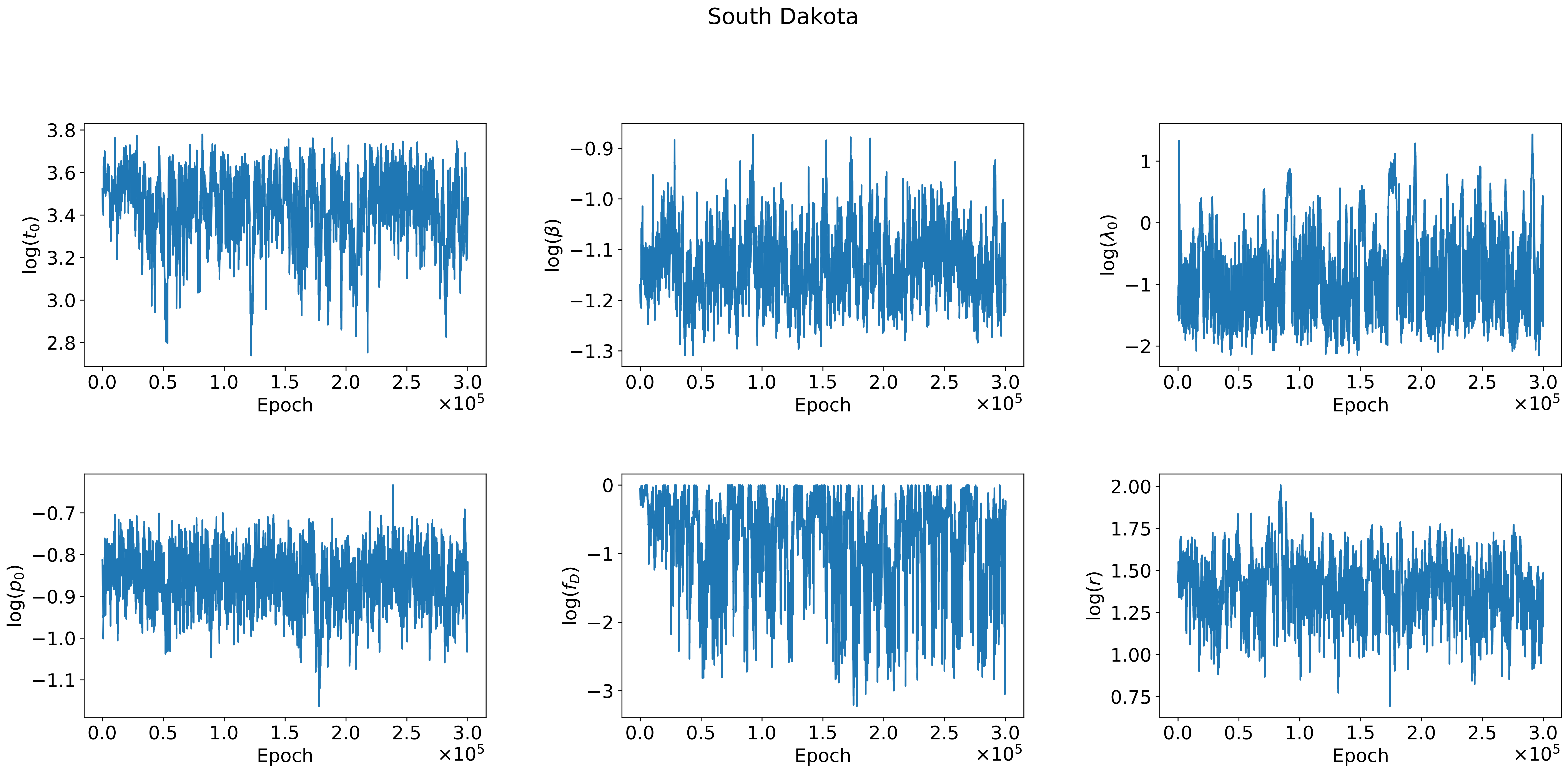

South Dakota
log(t_0)
log(β)
log(λ_0)
log(p_0)
log(f_D)
log(r)
Epoch
×10^5

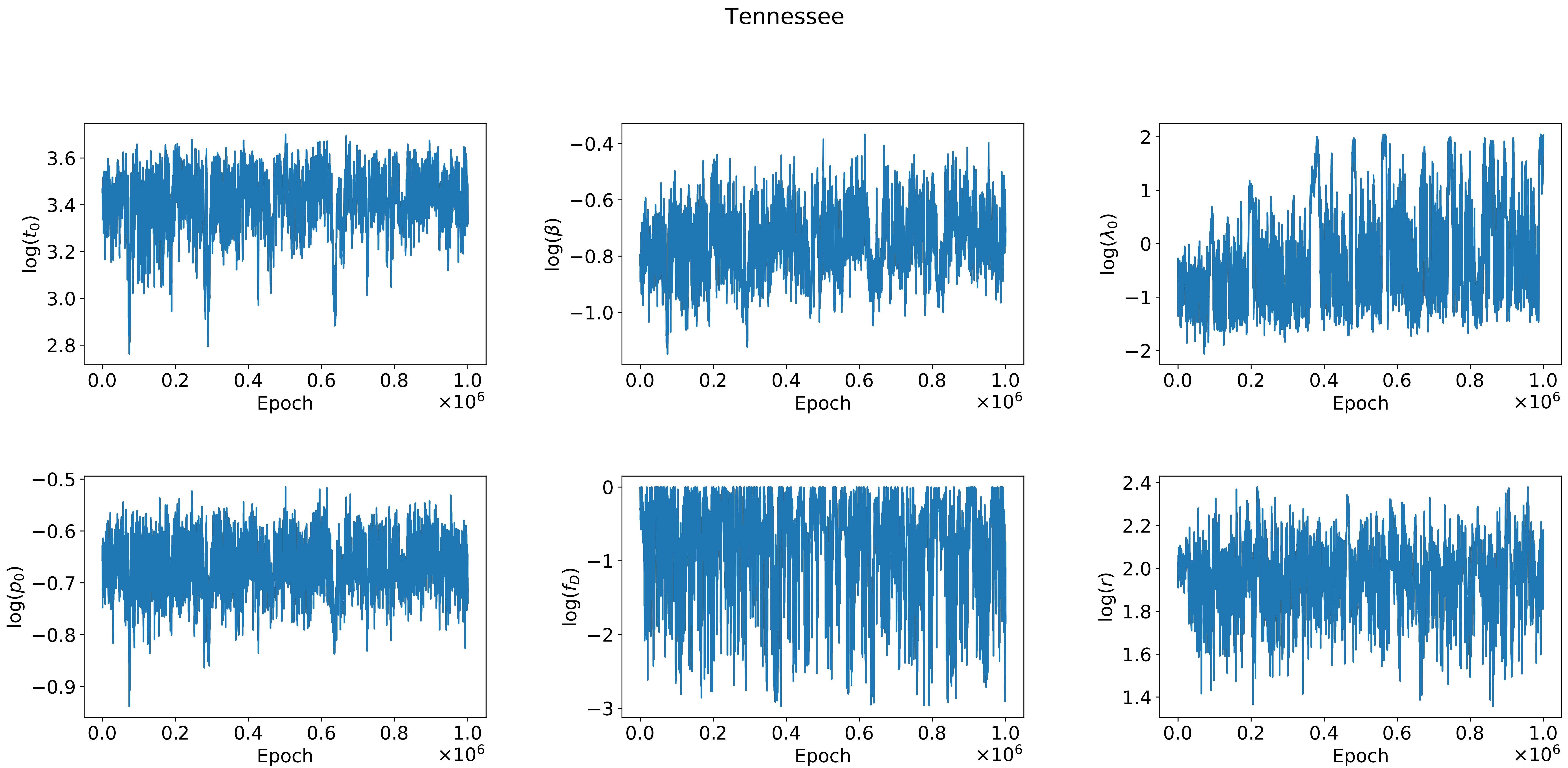
Tennessee
log(t_0)
3.6
3.4
3.2
3.0
2.8
log(β)
−0.4
−0.6
−0.8
−1.0
log(λ_0)
2
1
0
−1
−2
log(p_0)
−0.5
−0.6
−0.7
−0.8
−0.9
log(f_D)
0
−1
−2
−3
log(r)
2.4
2.2
2.0
1.8
1.6
1.4
0.0
0.2
0.4
0.6
0.8
1.0
Epoch
$\times 10^6$

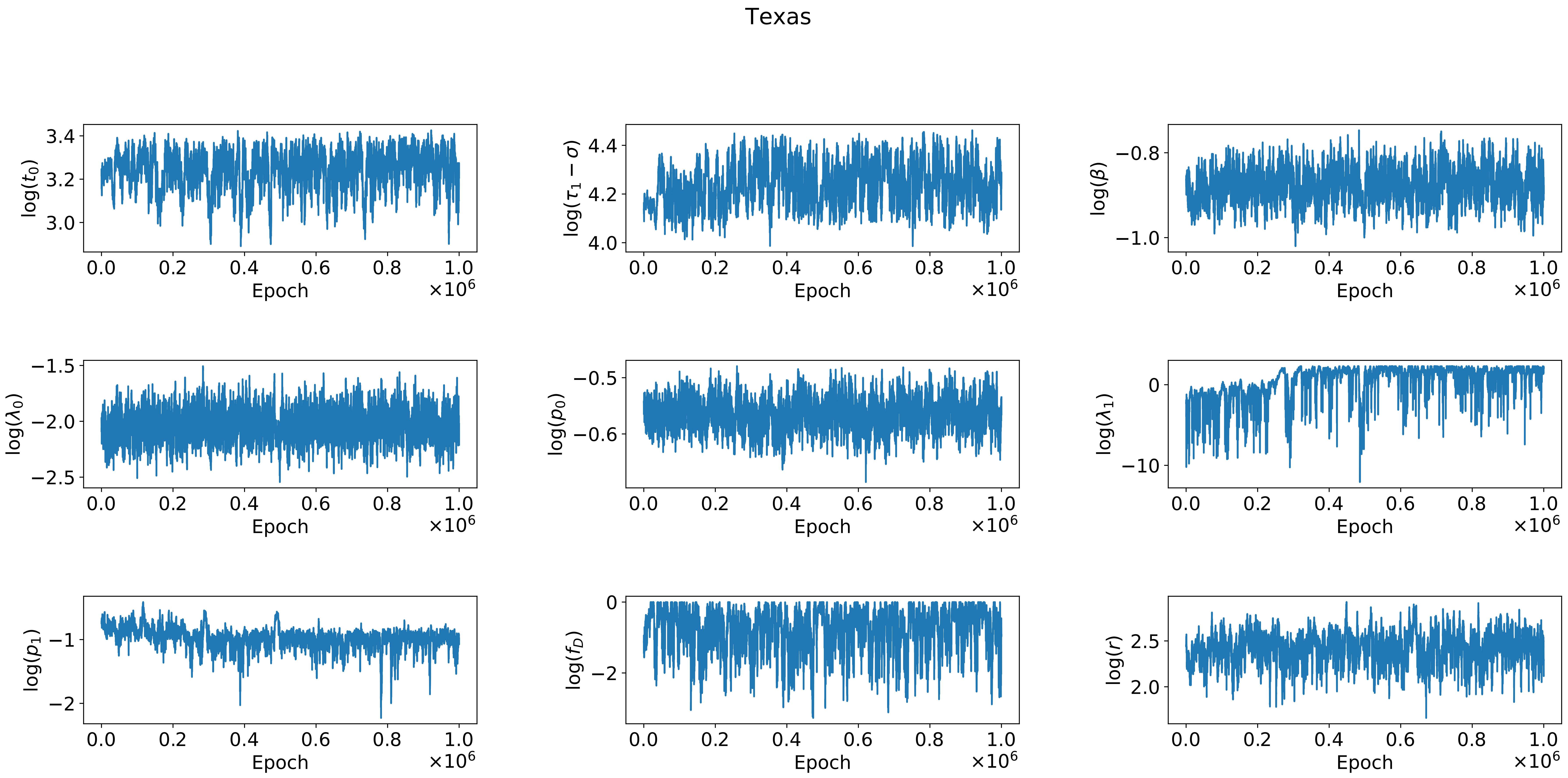
Texas
log(t_0)
3.4
3.2
3.0
log($\tau_1 - \sigma$)
4.4
4.2
4.0
log(β)
−0.8
−1.0
log(λ_0)
−1.5
−2.0
−2.5
log(p_0)
−0.5
−0.6
log(λ_1)
0
−10
log(p_1)
−1
−2
log(f_D)
0
−2
log(r)
2.5
2.0
0.0 0.2 0.4 0.6 0.8 1.0
Epoch
×10^6

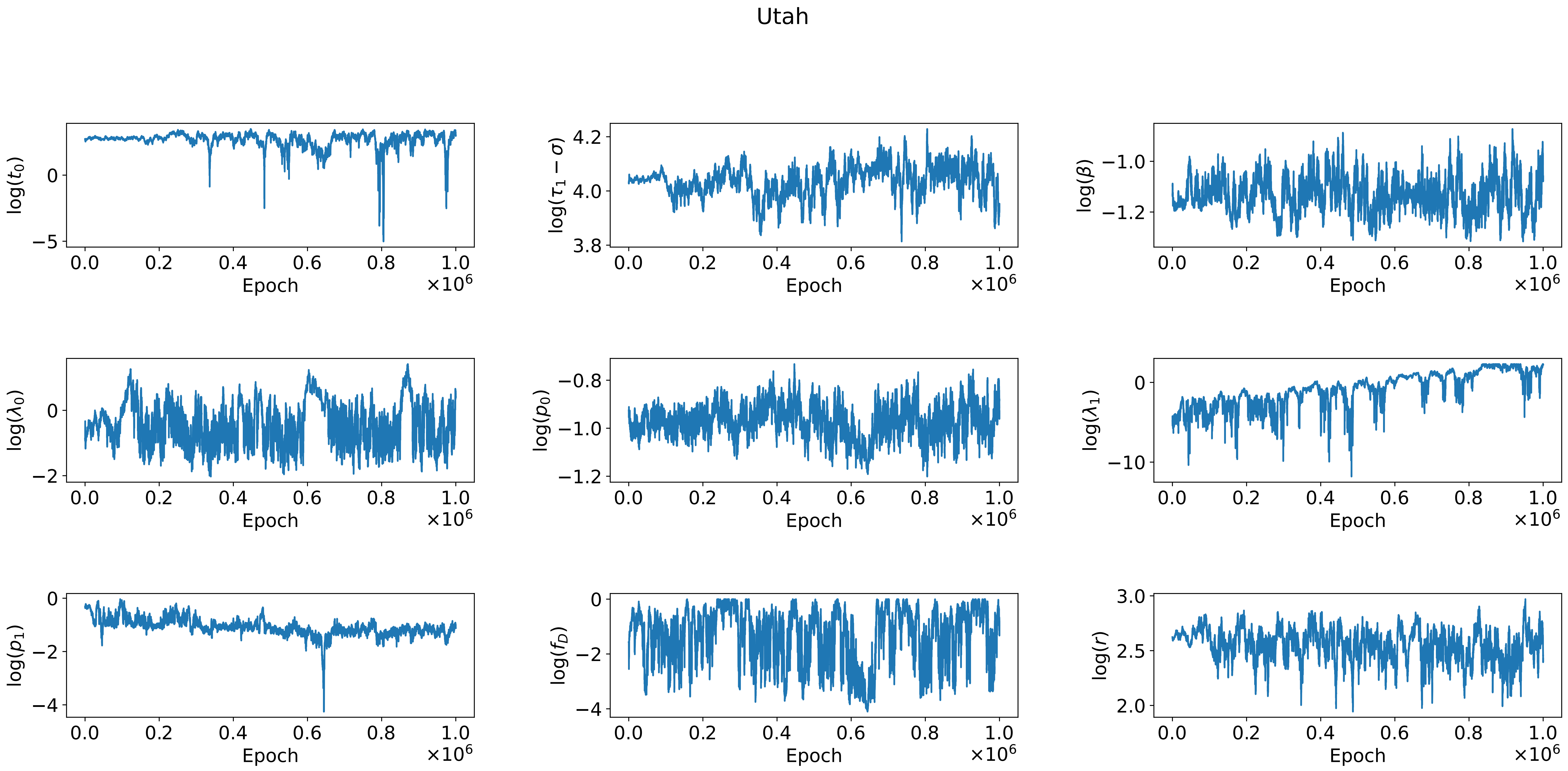
Utah
log(t_0)
0
−5
log($\tau_1 - \sigma$)
4.2
4.0
3.8
log(β)
−1.0
−1.2
log(λ_0)
0
−2
log(p_0)
−0.8
−1.0
−1.2
log(λ_1)
0
−10
log(p_1)
0
−2
−4
log(f_D)
0
−2
−4
log(r)
3.0
2.5
2.0
0.0 0.2 0.4 0.6 0.8 1.0
Epoch
×10^6

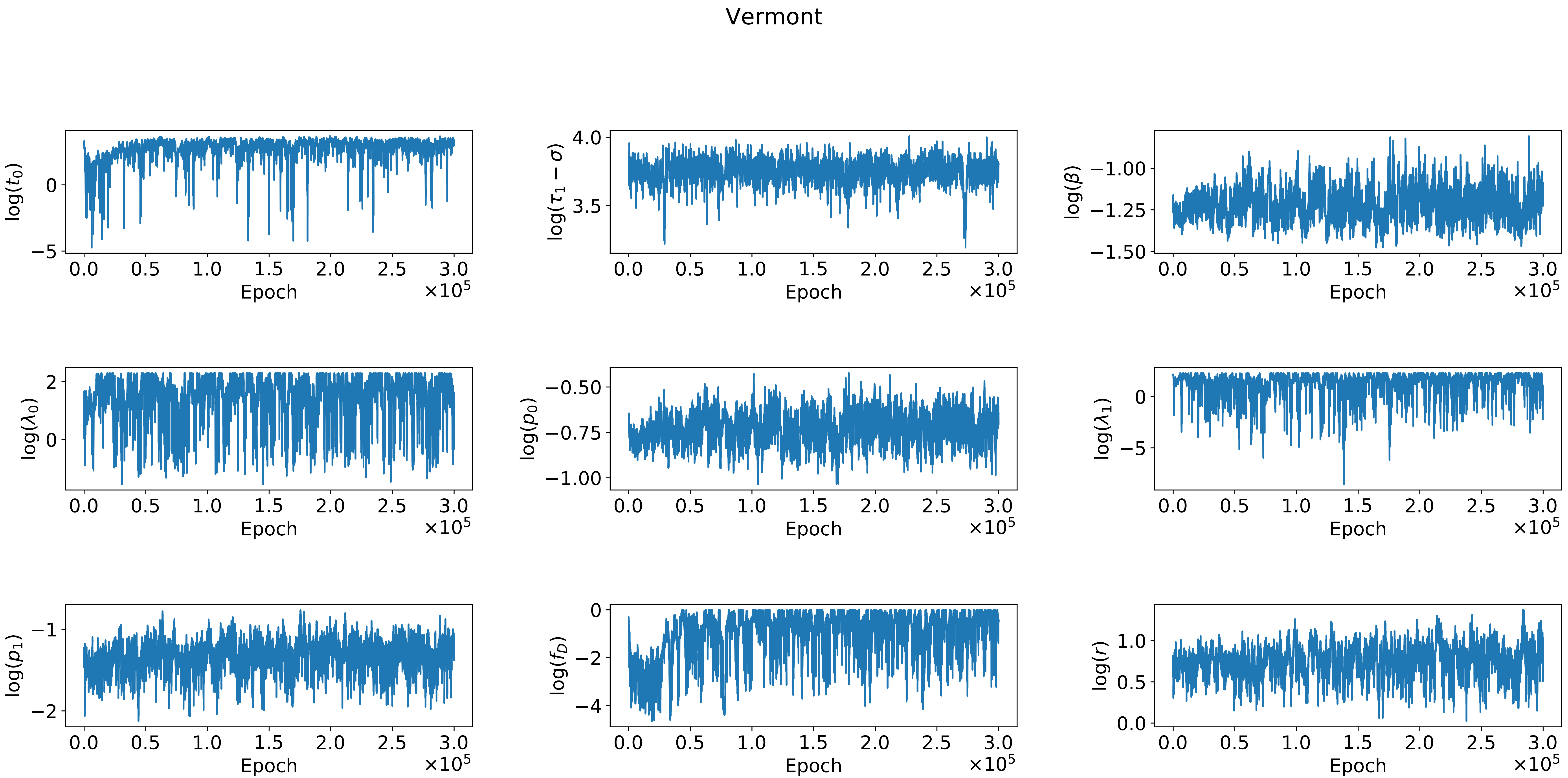
Vermont
log(t_0)
log($\tau_1 - \sigma$)
log(β)
log(λ_0)
log(p_0)
log(λ_1)
log(p_1)
log(f_D)
log(r)
Epoch
$\times 10^5$

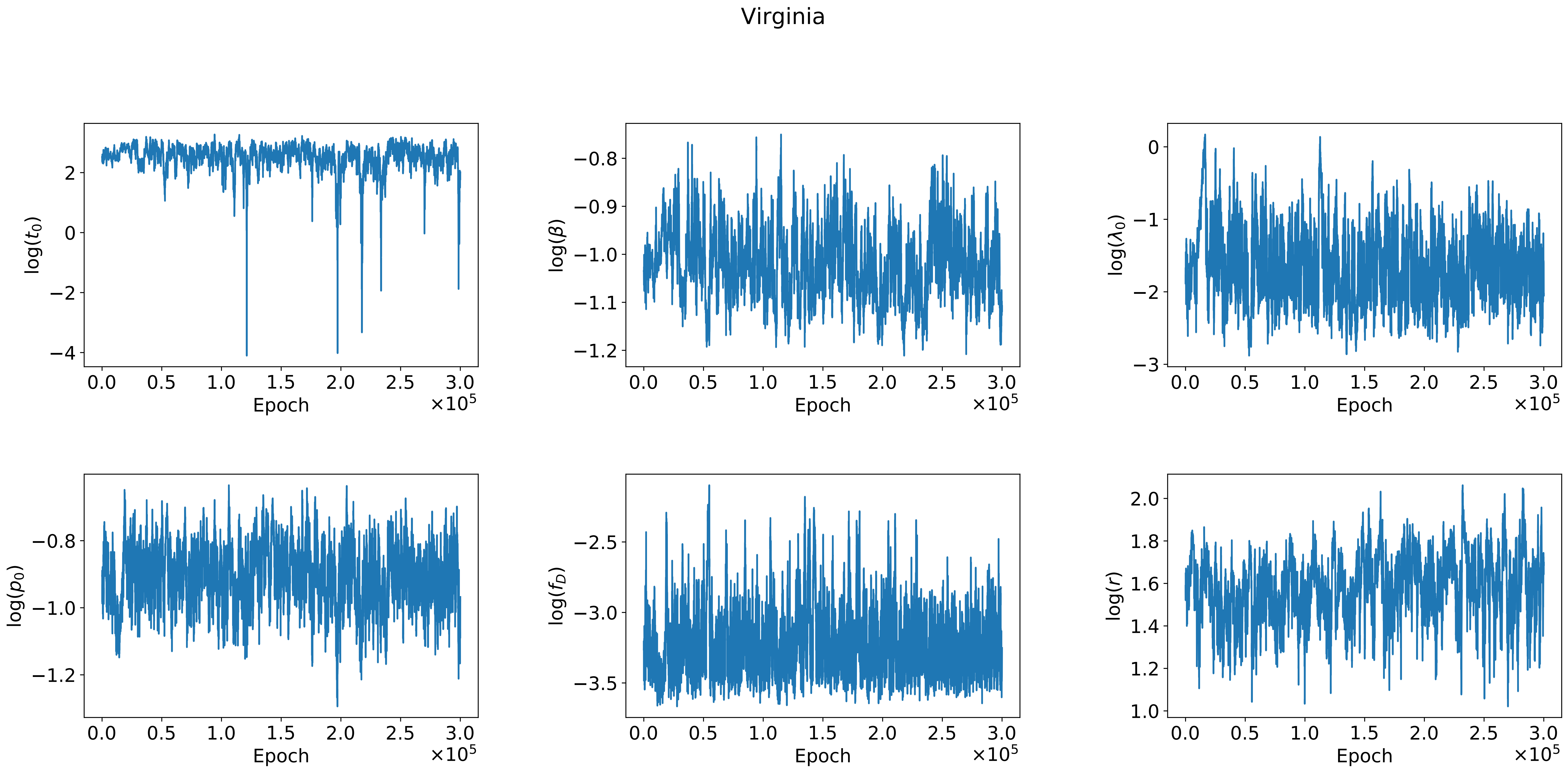

Virginia
log(t_0)
log(β)
log(λ_0)
log(p_0)
log(f_D)
log(r)
Epoch
$\times 10^5$

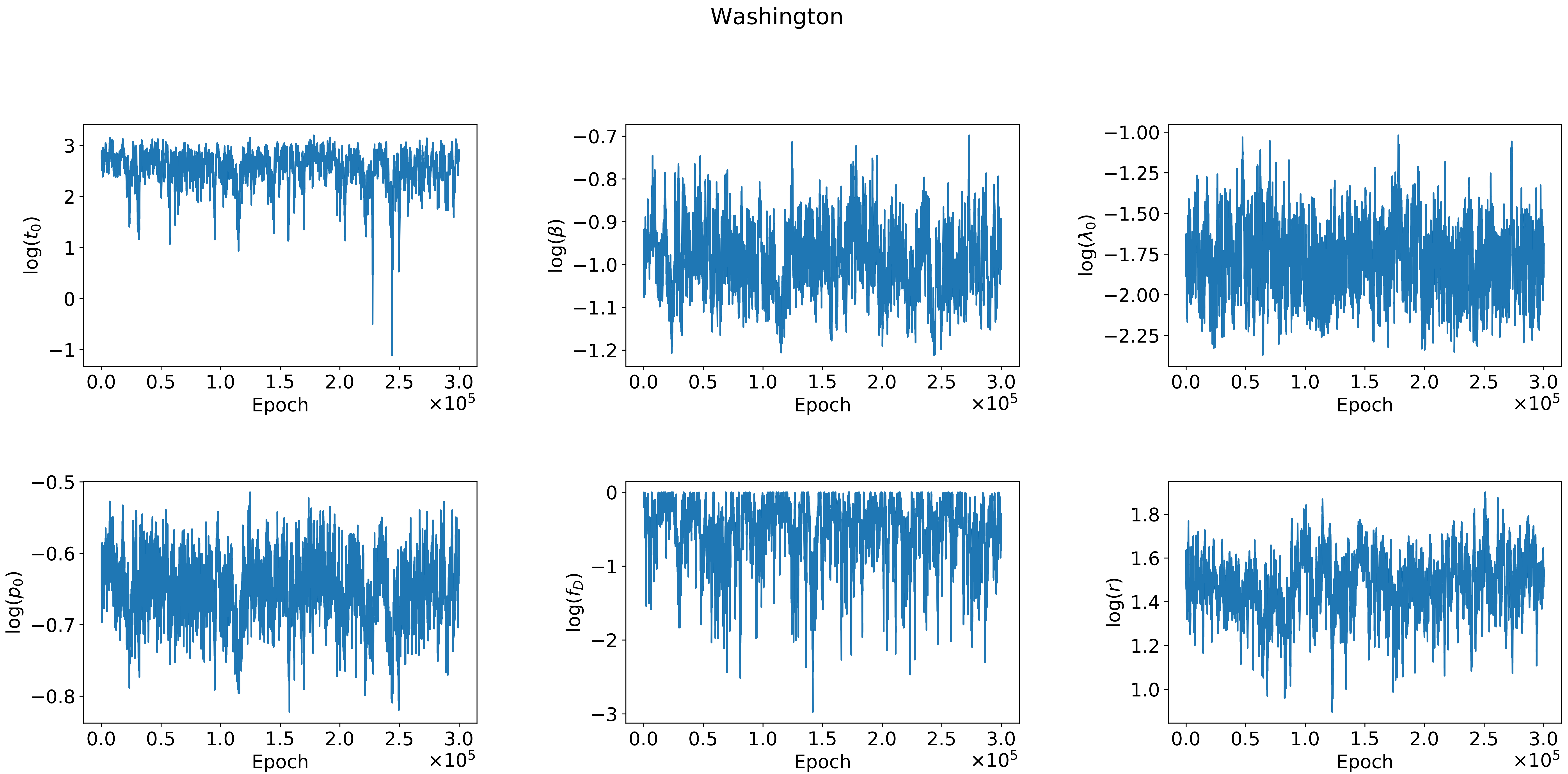

Washington
log(t_0)
log(β)
log(λ_0)
log(p_0)
log(f_D)
log(r)
Epoch
$\times 10^5$

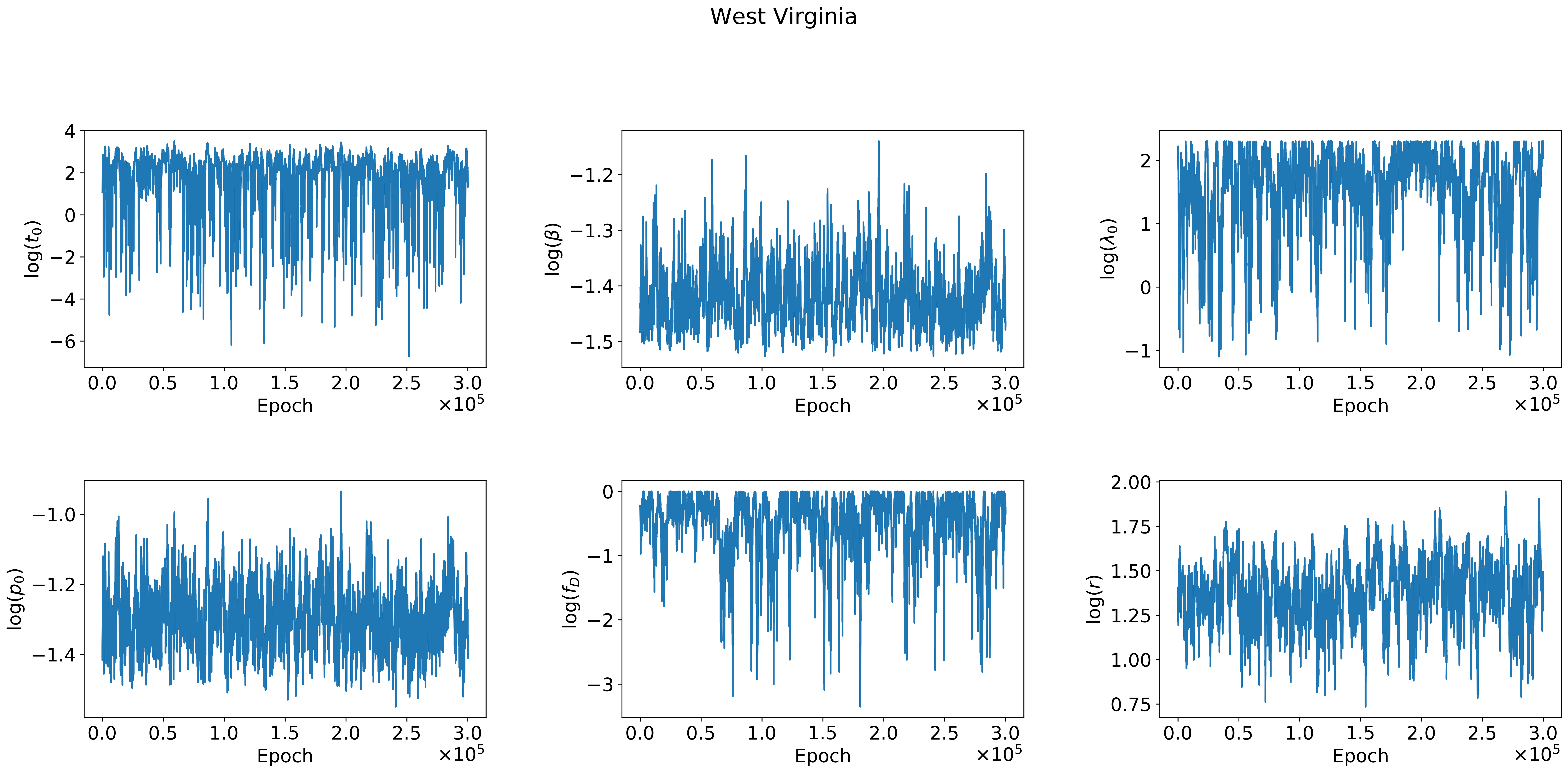
West Virginia
log(t_0)
log(β)
log(λ_0)
log(p_0)
log(f_D)
log(r)
Epoch
$\times 10^5$

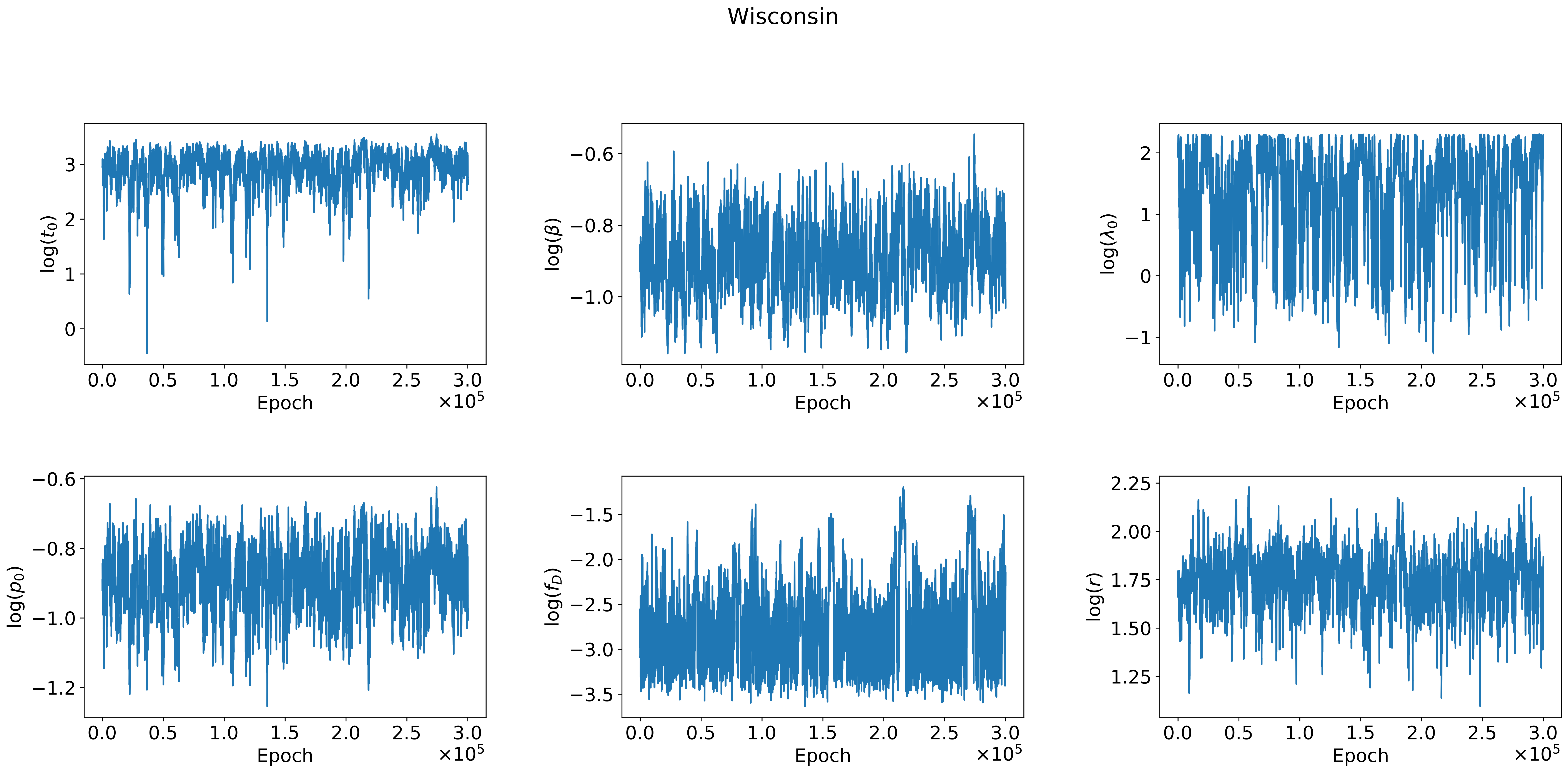
Wisconsin
log(t_0)
log(β)
log(λ_0)
log(p_0)
log(f_D)
log(r)
Epoch
$\times 10^5$

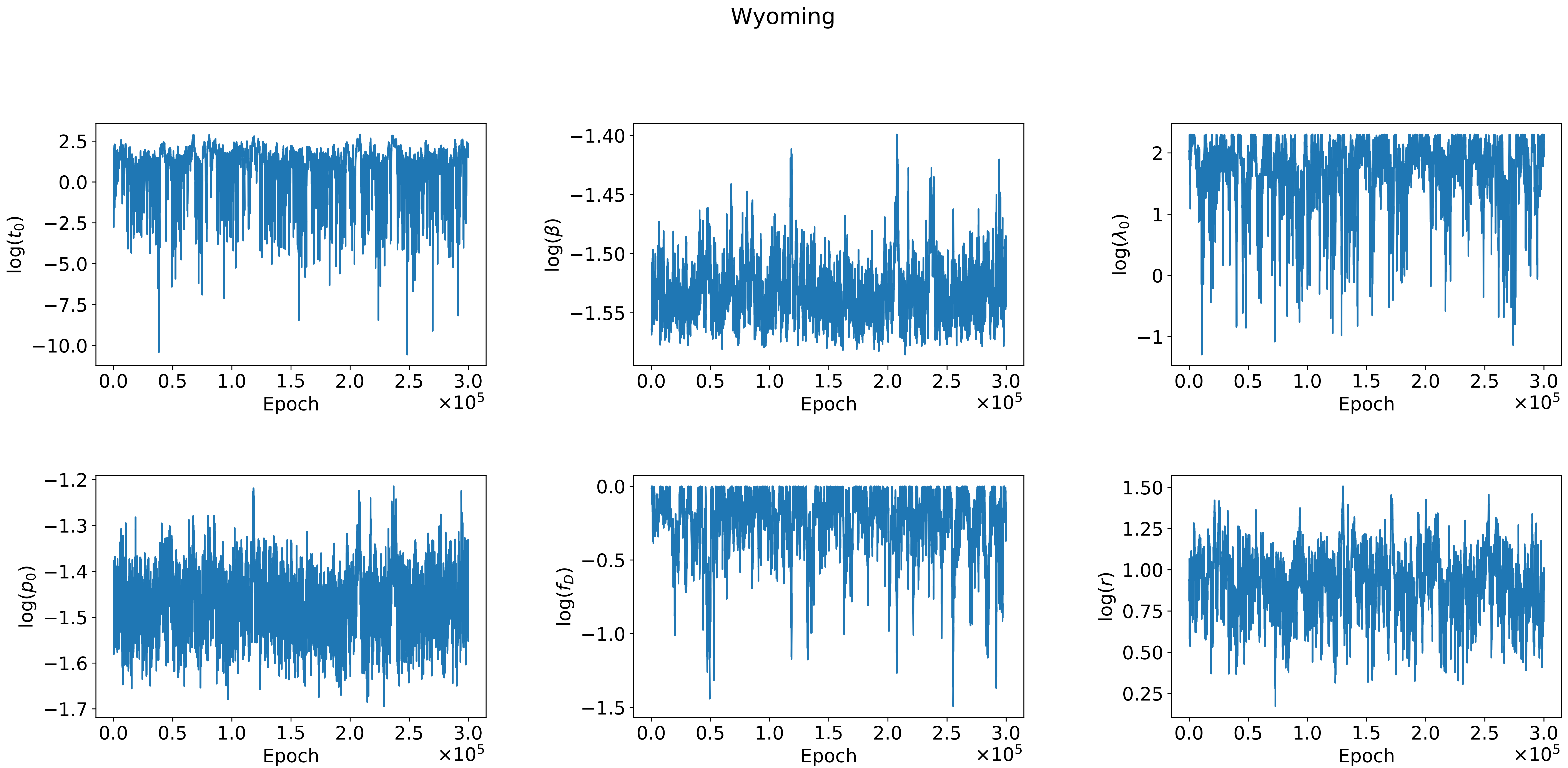

Wyoming
$\log(t_0)$
$\log(\beta)$
$\log(\lambda_0)$
$\log(p_0)$
$\log(f_D)$
$\log(r)$
Epoch
$\times 10^5$

Figure S4. Matrix of 1- and 2-dimensional marginalizations of the posterior samples obtained for the adjustable parameters associated with the compartmental model for each of the 50 US states. Inferences are based on daily reports of new cases of COVID-19 from January 21 to June 21, 2020. Plots of marginal posteriors (1-dimensional marginalizations) are shown on the diagonal from top left to bottom right. Other plots are 2-dimensional marginalizations (presented as histograms) indicating the correlations between parameter estimates. Brightness indicates higher probability density. A compact bright area indicates low correlation. An extended, asymmetric bright area indicates high correlation. The pairs plots shown here are matched to the trace plots of Figs S3 and S4. See the caption of Fig S2 for additional details.

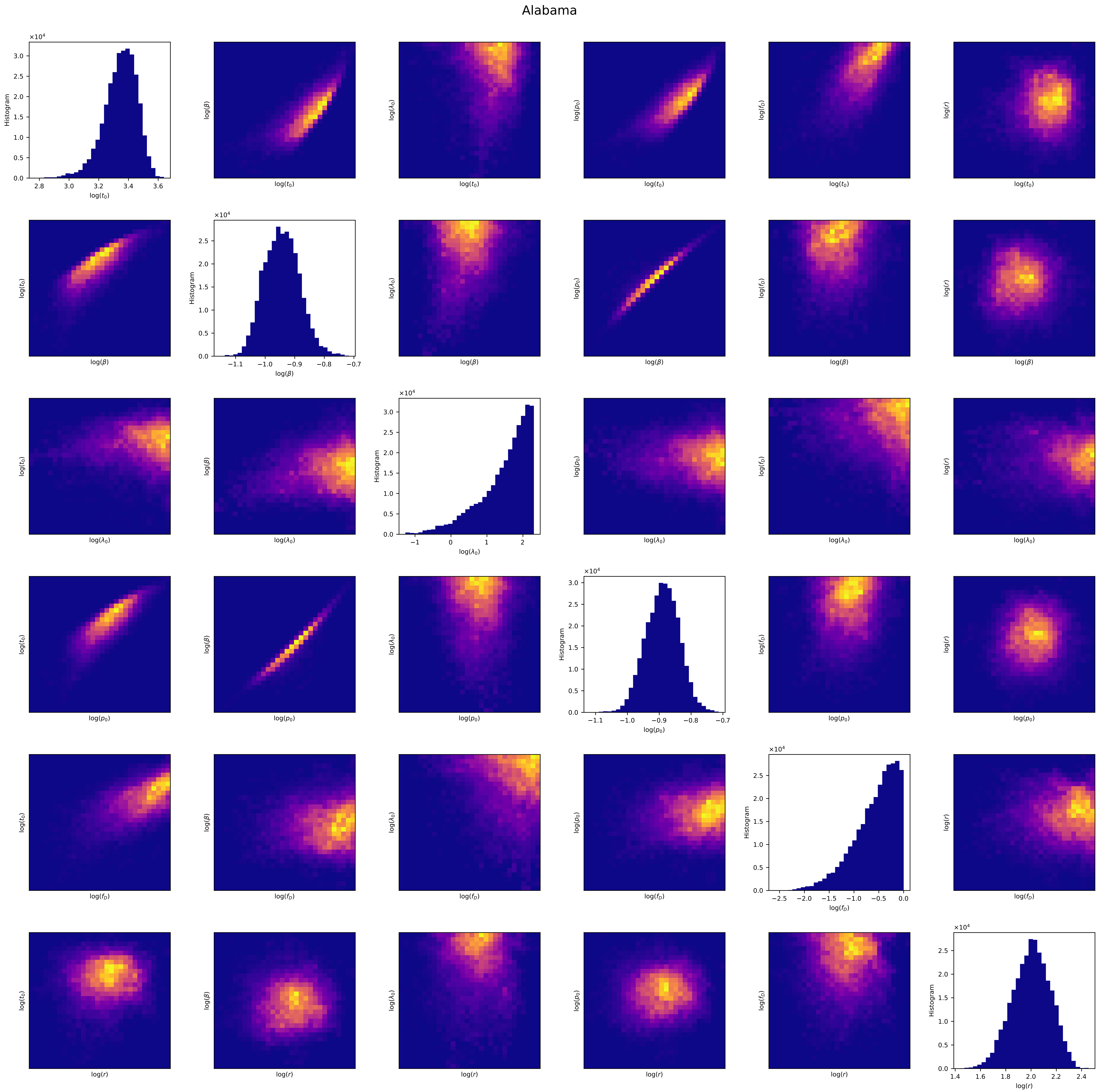

Alabama
Histogram
log(t_0)
log(β)
log(λ_0)
log(p_0)
log(f_D)
log(r)

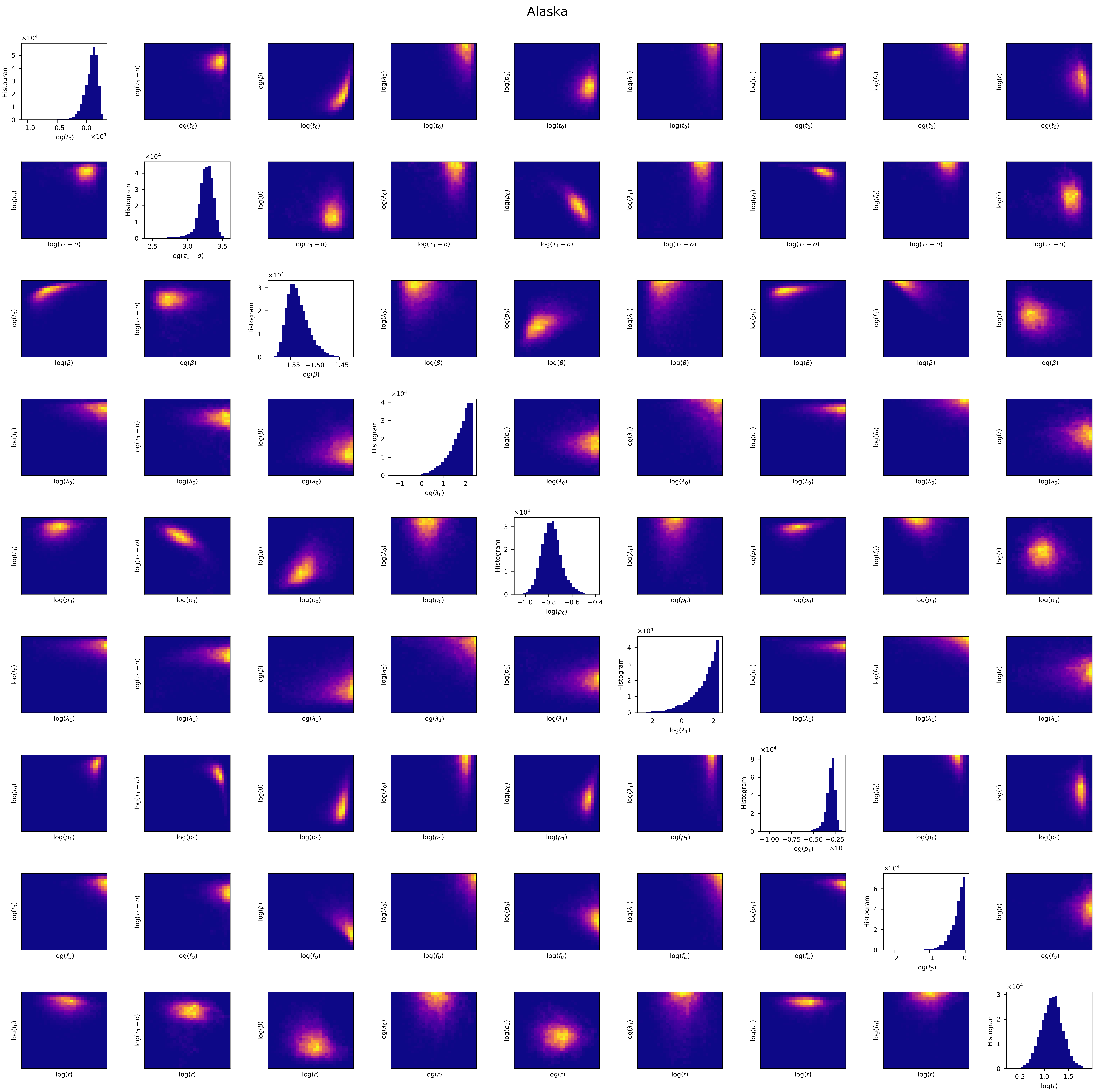
Alaska
Histogram
log(t_0)
log($\tau_1 - \sigma$)
log(β)
log(λ_0)
log(p_0)
log(λ_1)
log(p_1)
log(f_D)
log(r)

Arizona

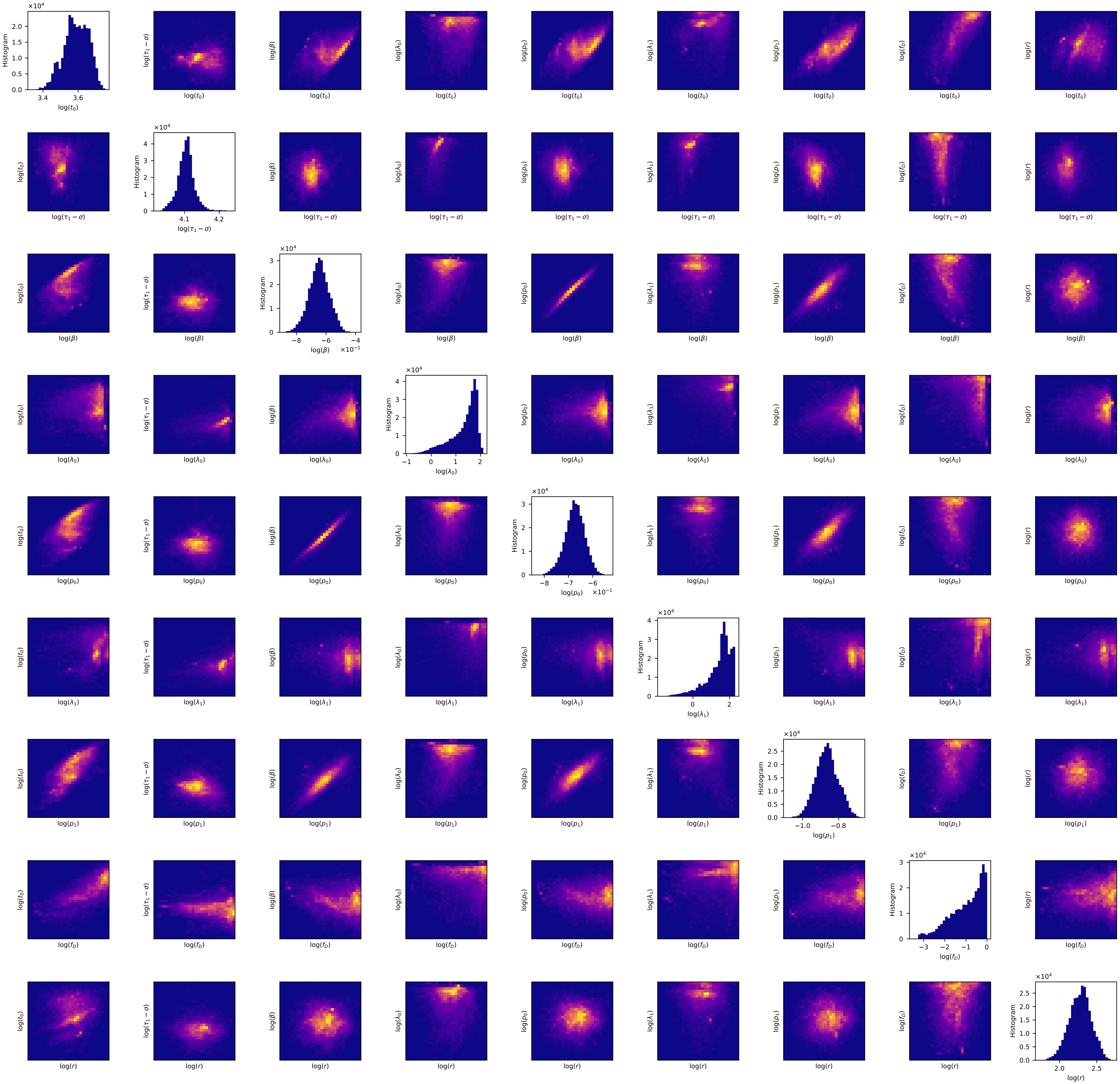

Histogram
×10^4
log(t_0)
log($\tau_1 - \sigma$)
log(β)
log(λ_0)
log(p_0)
log(λ_1)
log(p_1)
log(f_D)
log(r)
0.0 0.5 1.0 1.5 2.0
3.4 3.6
0 1 2 3 4
4.1 4.2
−8 −6 −4 ×10^{-1}
−1 0 1 2
−8 −7 −6 ×10^{-1}
0 2
−1.0 −0.8
−3 −2 −1 0
2.0 2.5

Arkansas

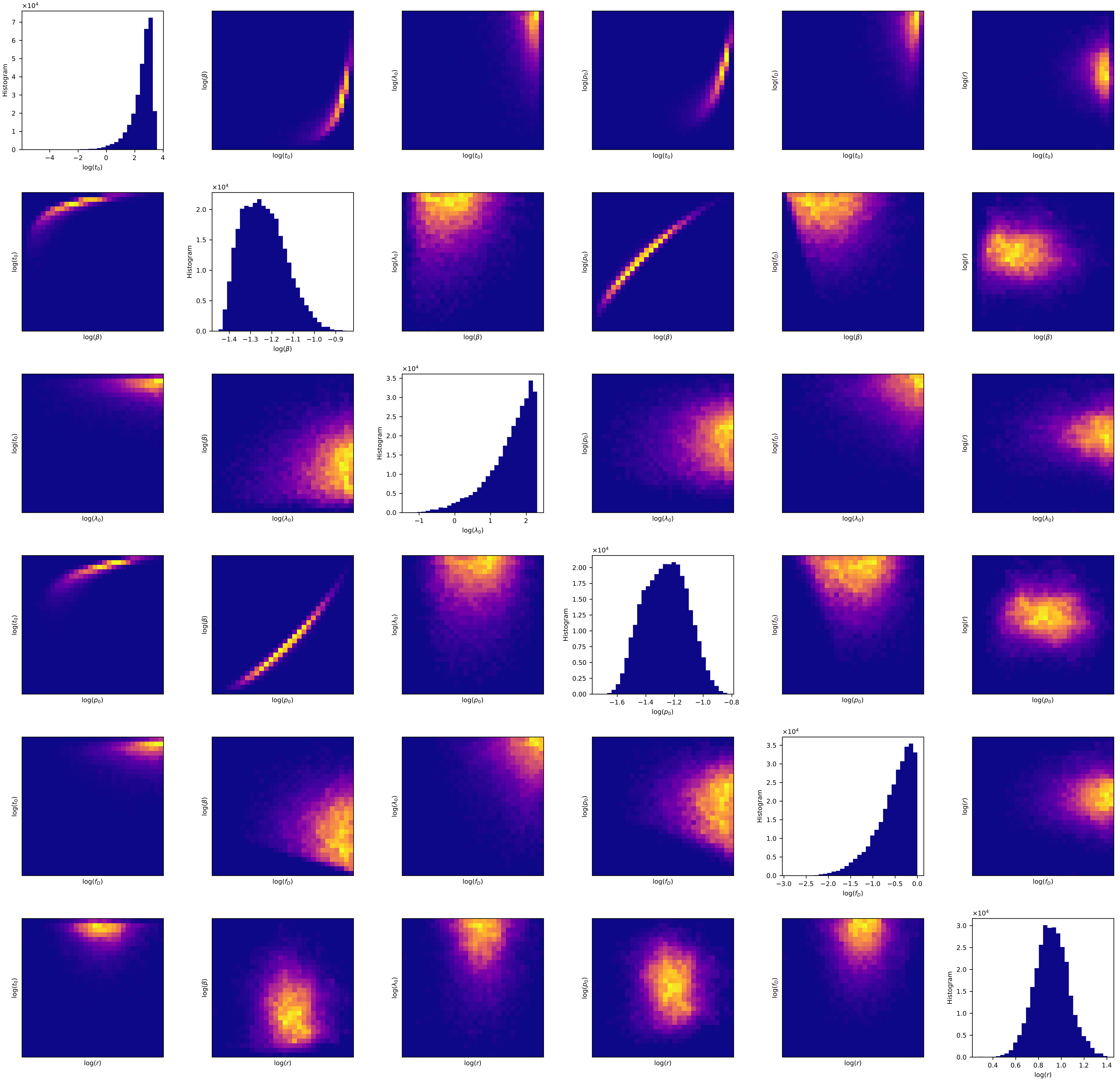

California

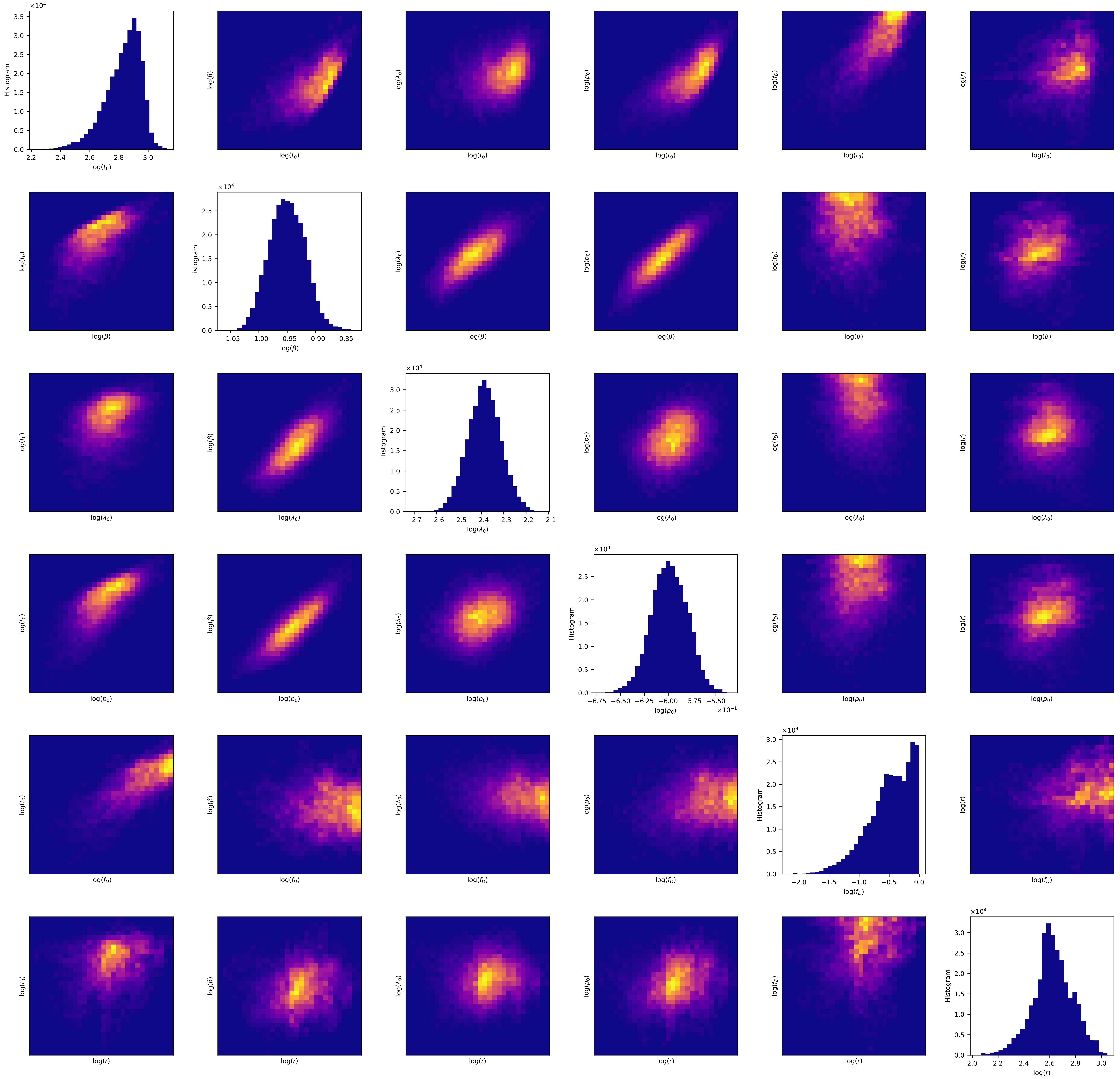

Colorado

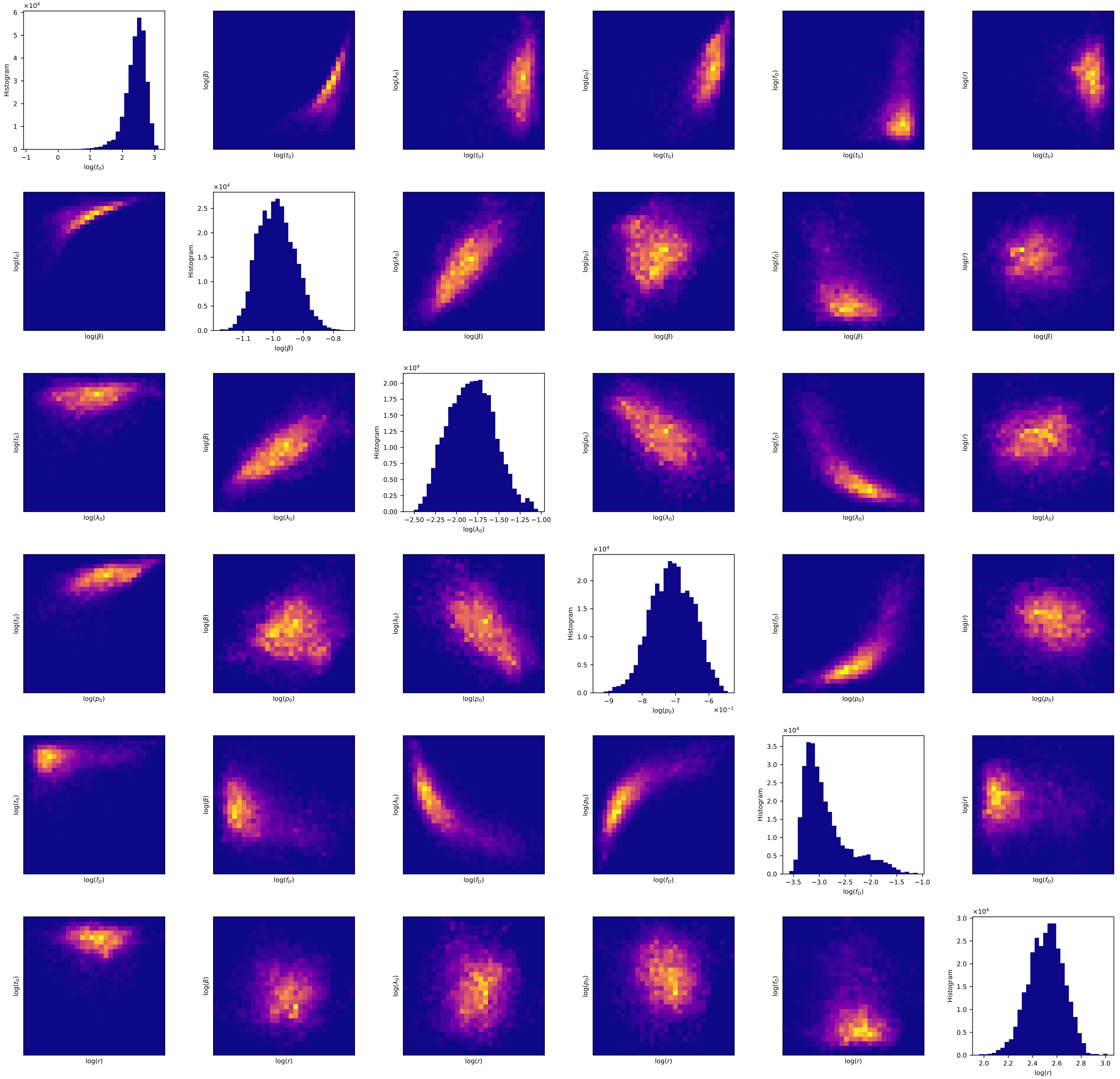

Histogram
log(t_0)
log(β)
log(λ_0)
log(p_0)
log(f_D)
log(r)
$\times10^4$
$\times10^{-1}$

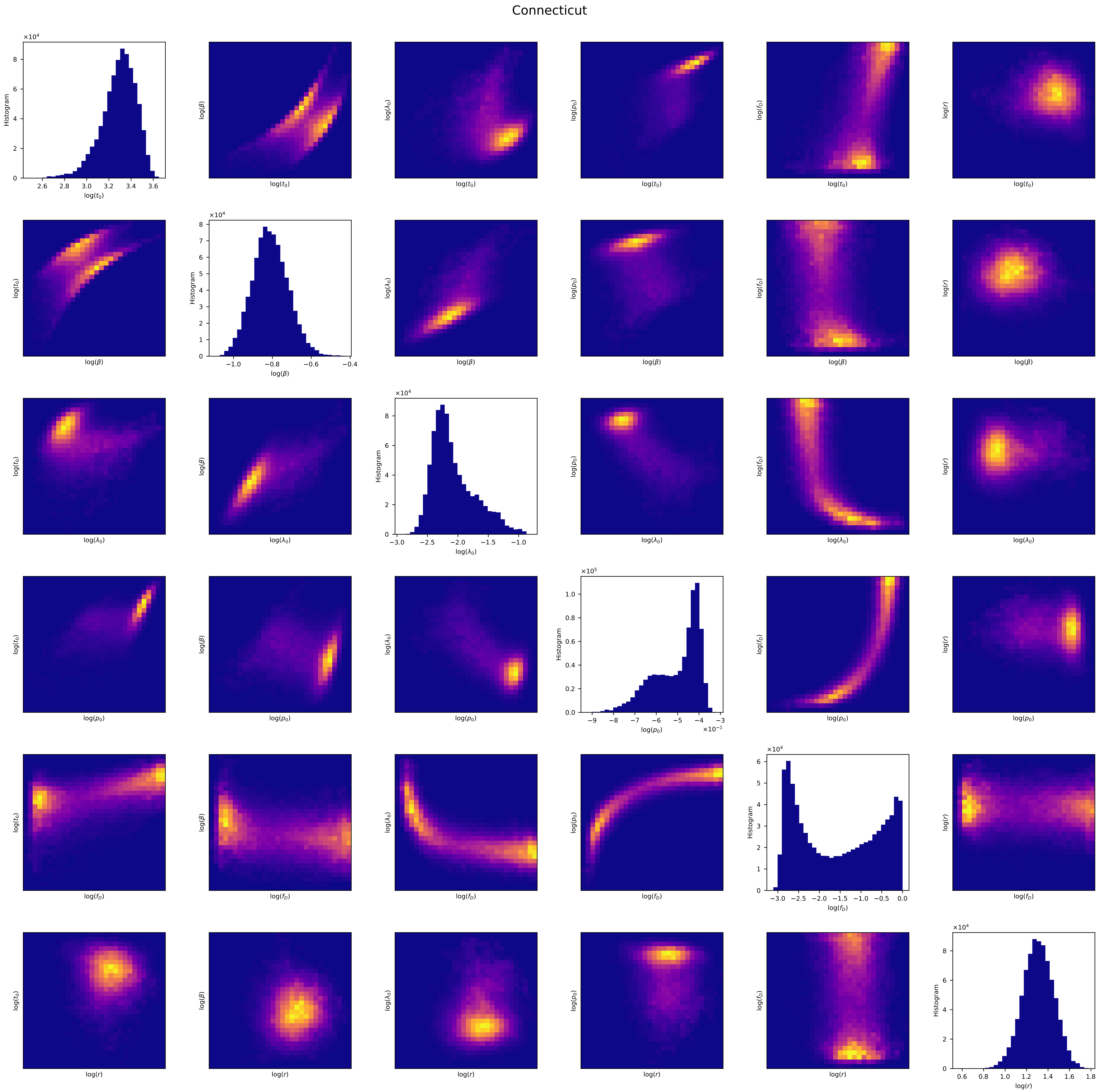

Connecticut
Histogram
log(t_0)
log(β)
log(λ_0)
log(p_0)
log(f_D)
log(r)
×10^4
×10^5
×10^-1

Delaware

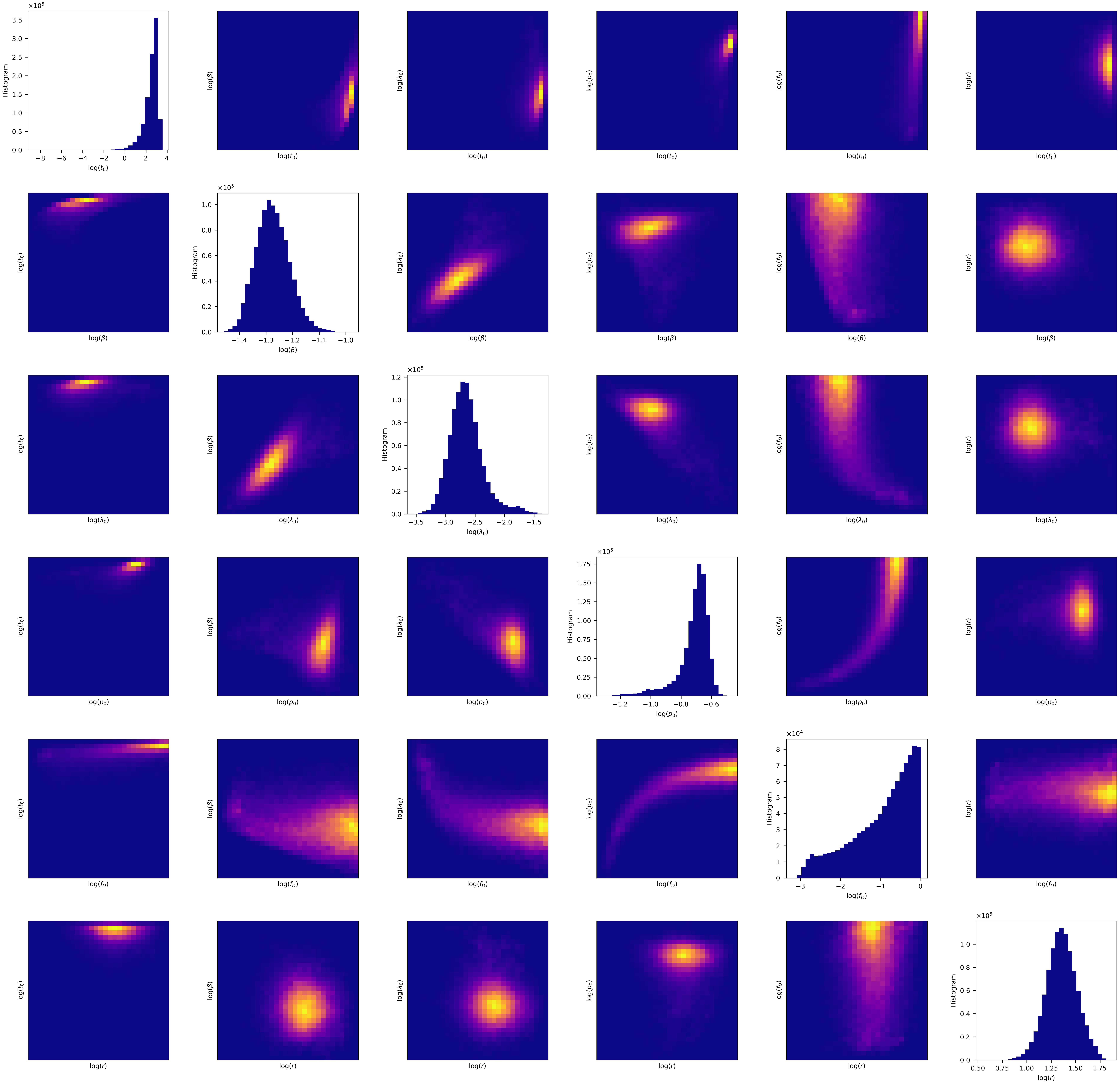

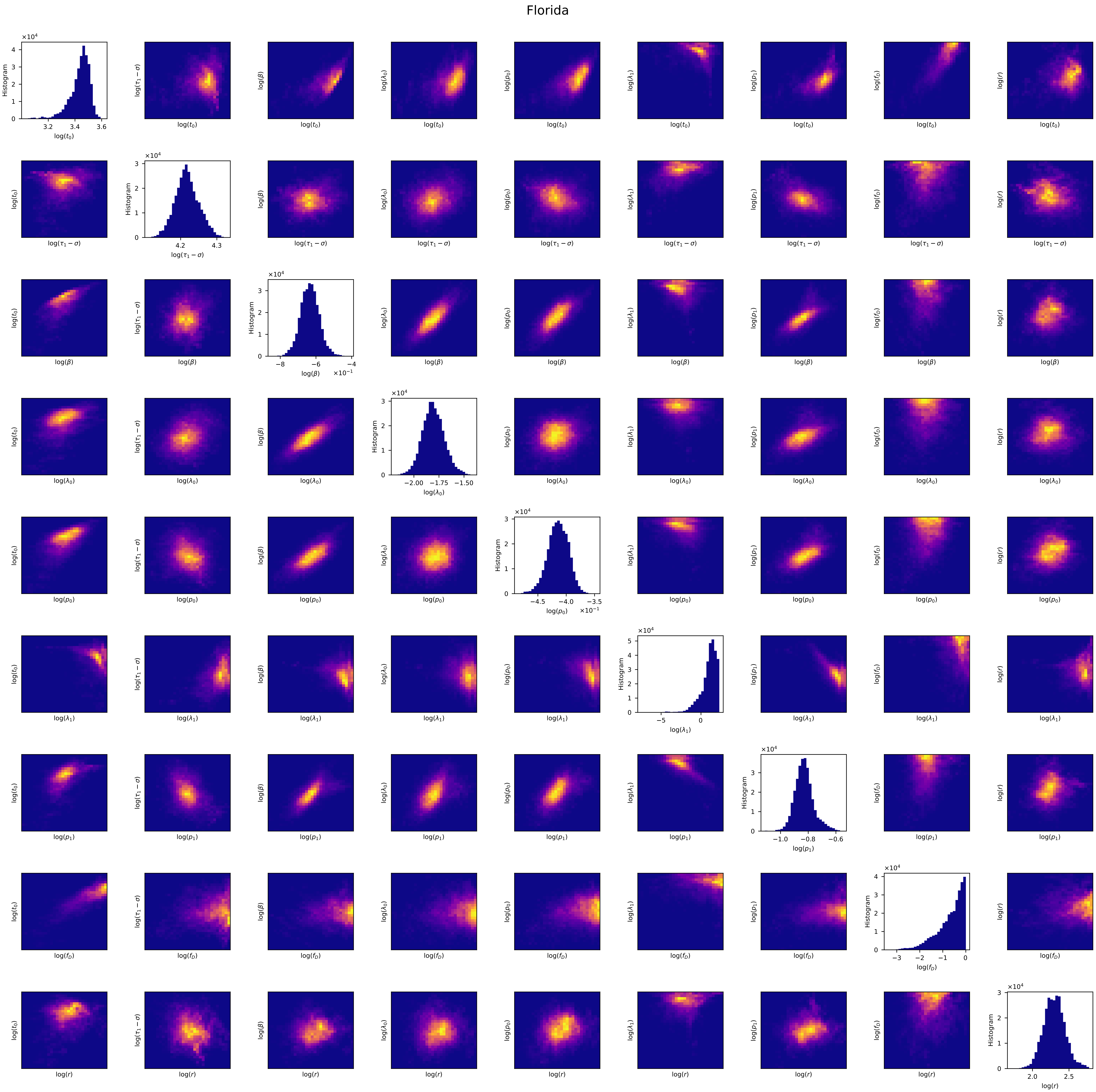
Florida
Histogram
log(t_0)
log($\tau_1 - \sigma$)
log(β)
log(λ_0)
log(p_0)
log(λ_1)
log(p_1)
log(f_D)
log(r)
$\times 10^4$
$\times 10^{-1}$

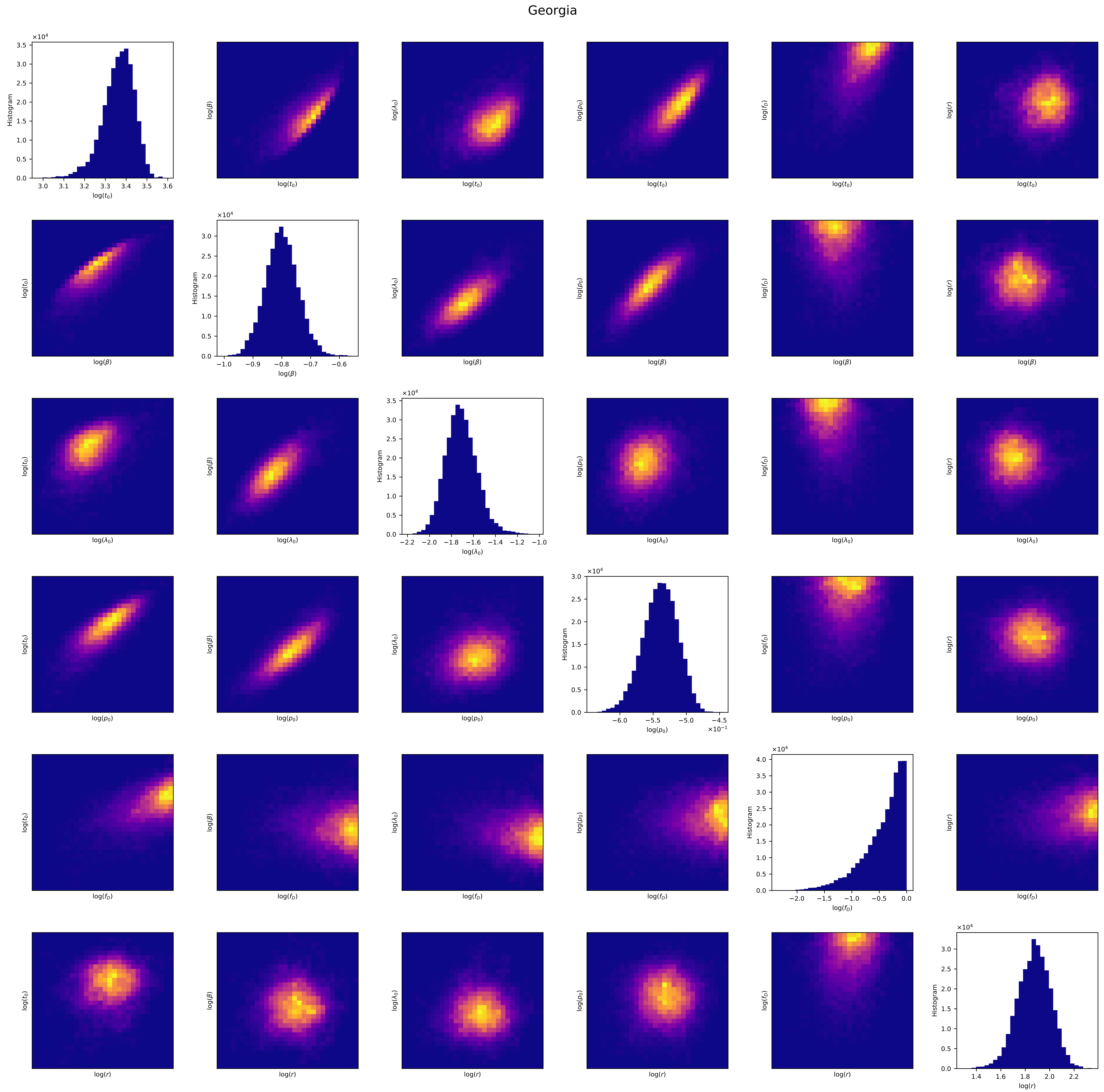
Georgia
Histogram
log(t_0)
log(β)
log(λ_0)
log(p_0)
log(f_D)
log(r)

Hawaii

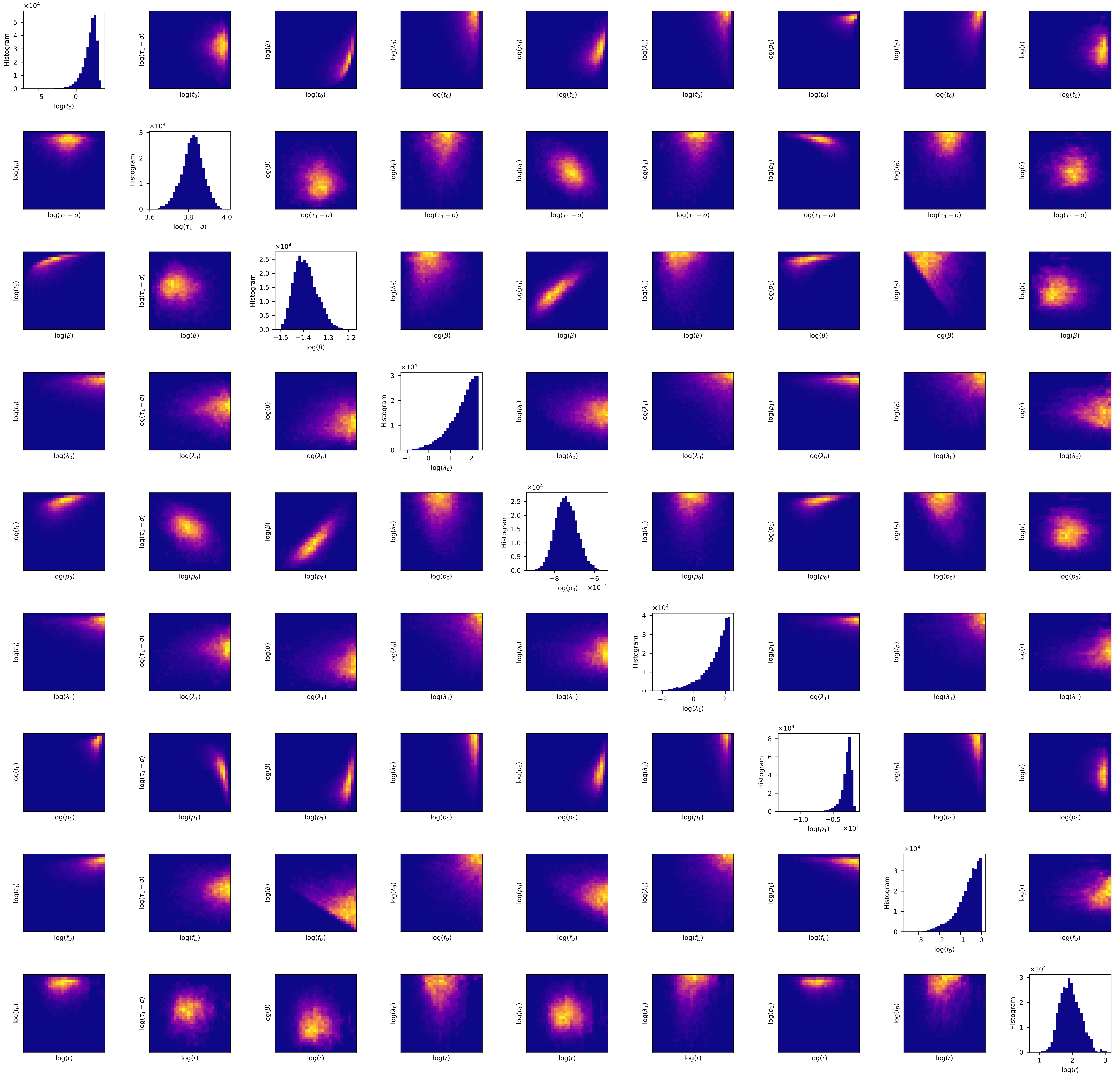

Histogram
log(t_0)
log($\tau_1-\sigma$)
log(β)
log(λ_0)
log(p_0)
log(λ_1)
log(p_1)
log(f_D)
log(r)

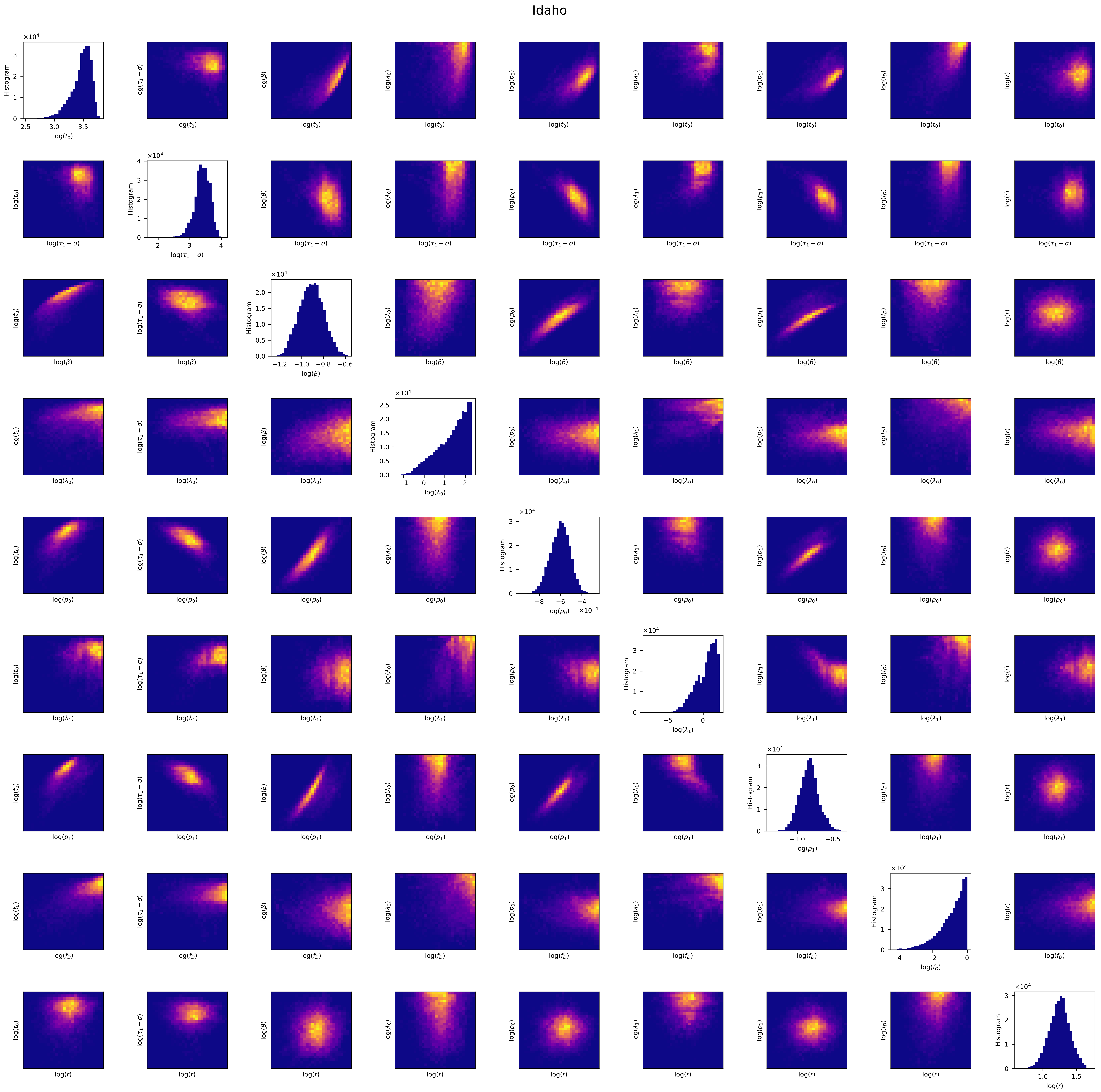
Idaho
log(t_0)
log($\tau_1 - \sigma$)
log(β)
log(λ_0)
log(p_0)
log(λ_1)
log(p_1)
log(f_D)
log(r)
Histogram

Illinois

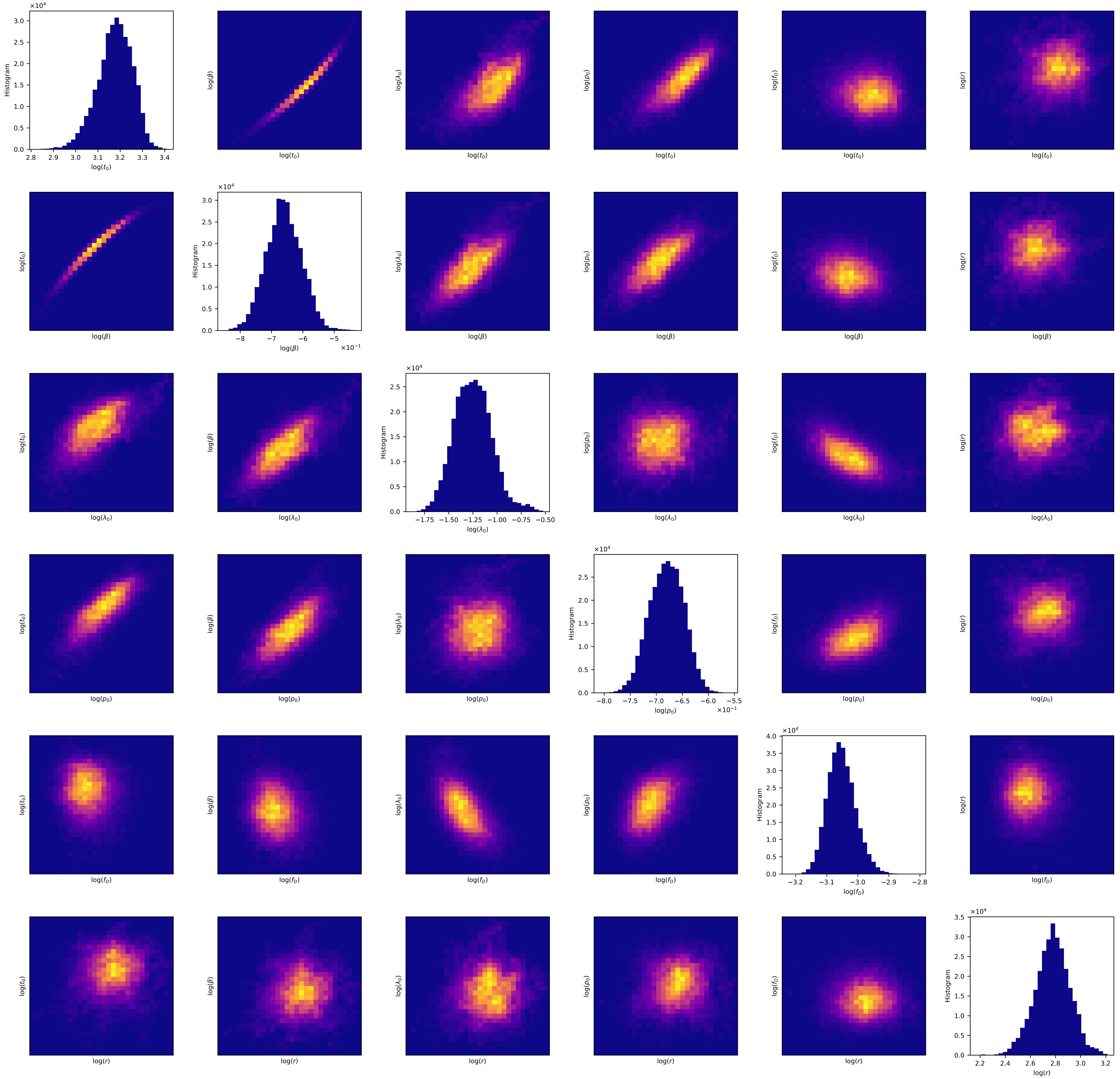

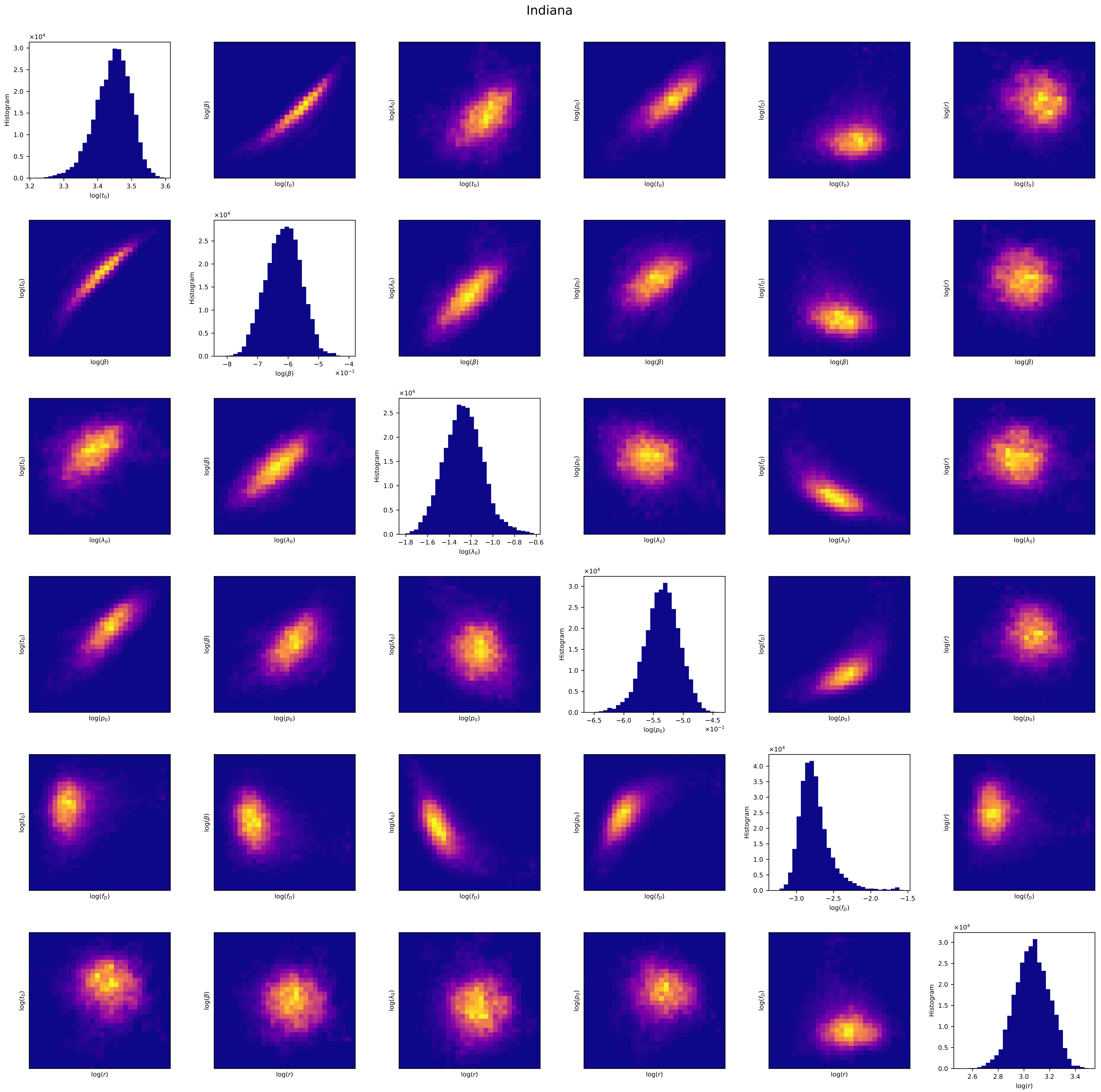
Indiana
Histogram
log(t_0)
log(β)
log(λ_0)
log(p_0)
log(f_D)
log(r)
$\times 10^4$
$\times 10^{-1}$

Iowa

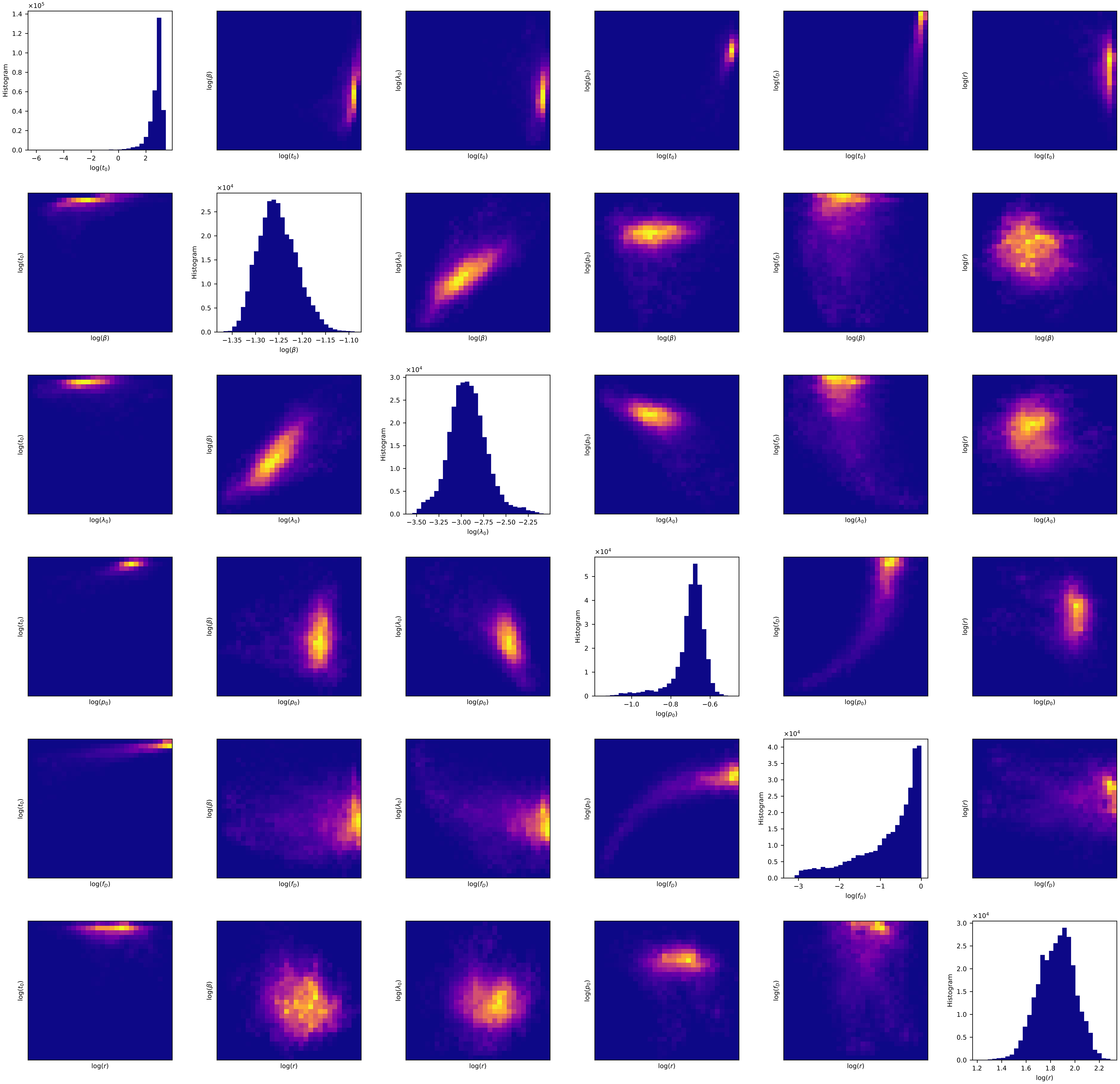

Kansas

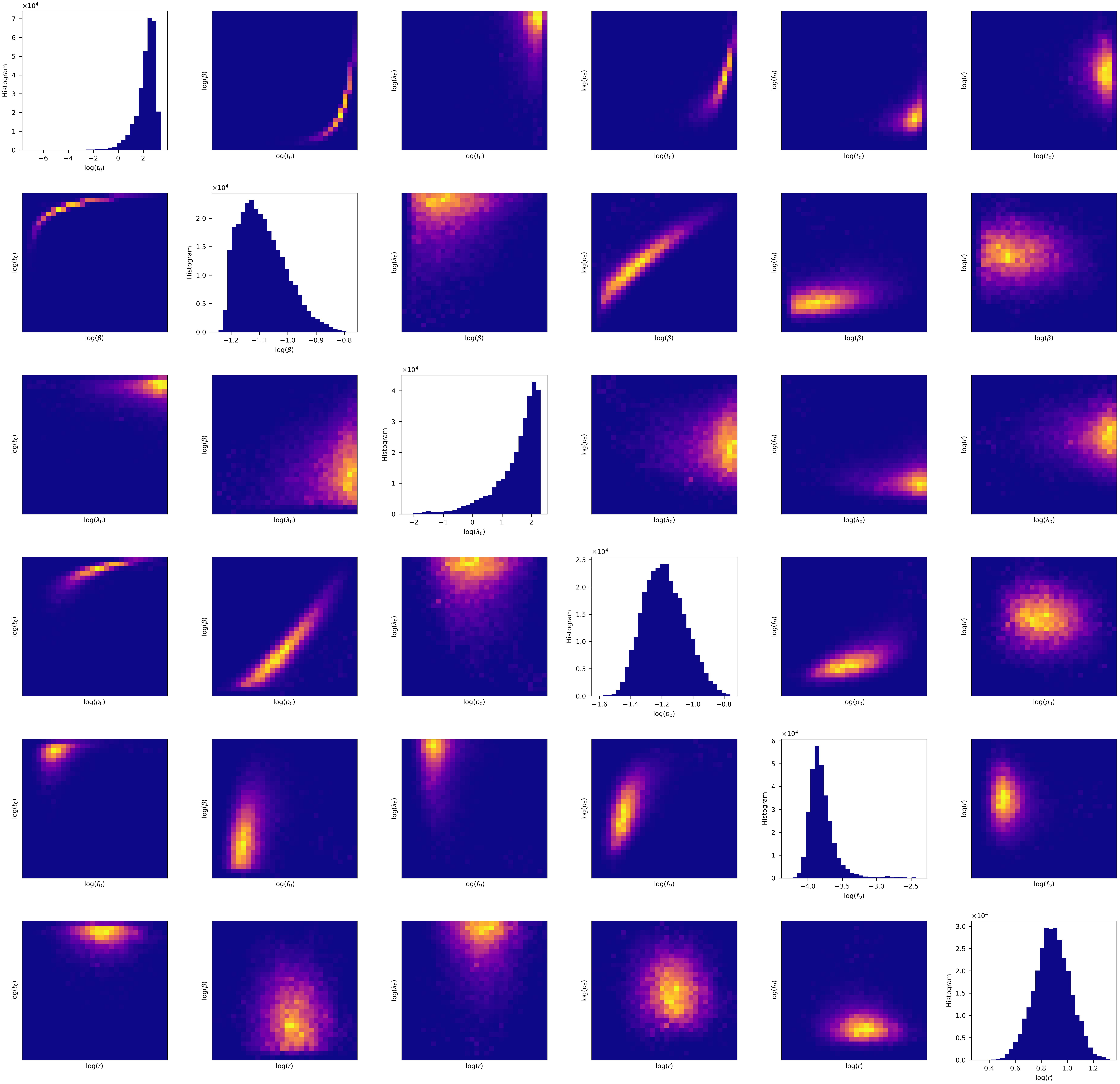

Histogram
log(t_0)
log(β)
log(λ_0)
log(p_0)
log(f_D)
log(r)
×10^4
−6 −4 −2 0 2
−1.2 −1.1 −1.0 −0.9 −0.8
−2 −1 0 1 2
−1.6 −1.4 −1.2 −1.0 −0.8
−4.0 −3.5 −3.0 −2.5
0.4 0.6 0.8 1.0 1.2

Kentucky

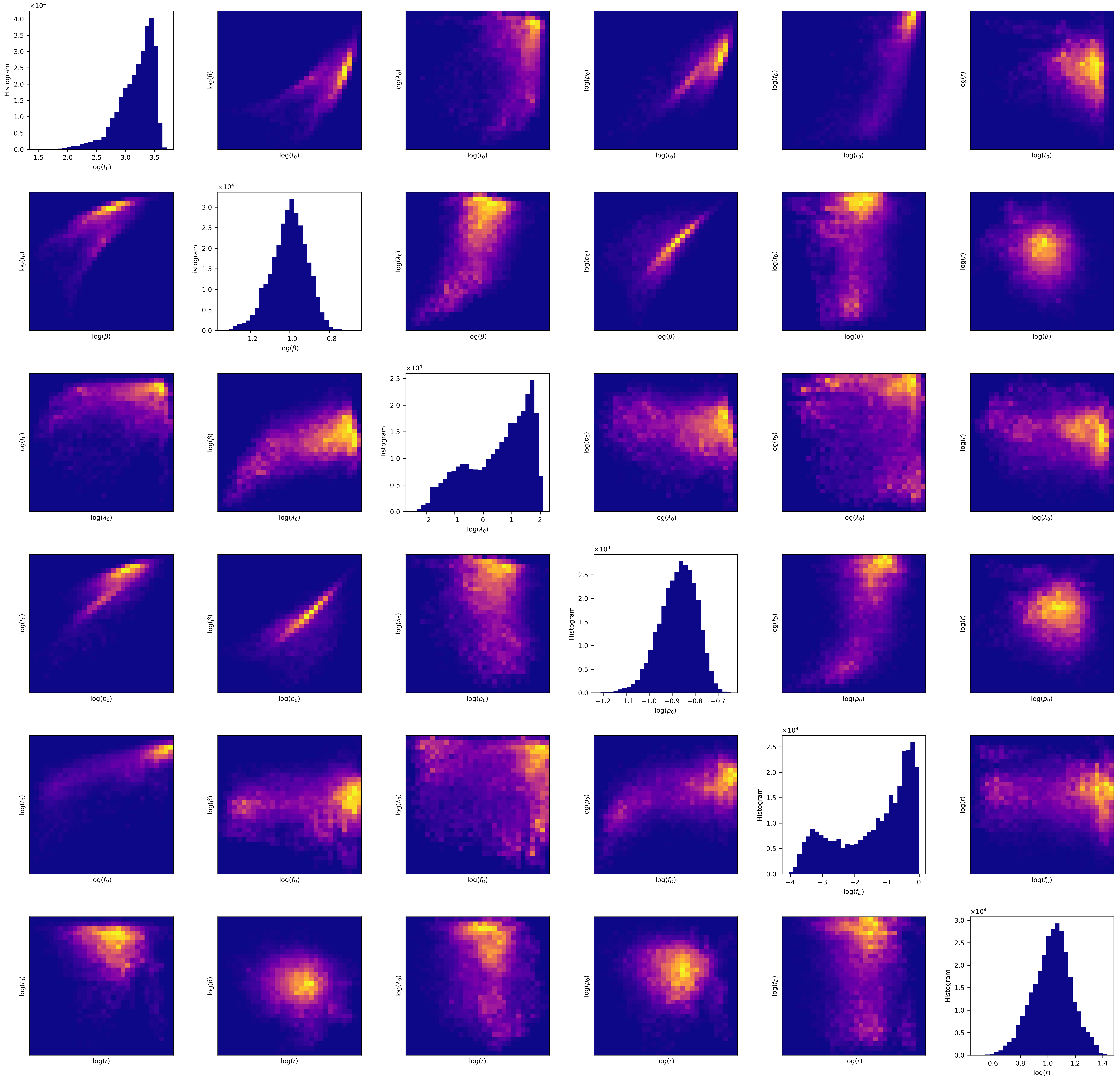
×10^4
Histogram
log(t_0)
log(β)
log(λ_0)
log(p_0)
log(f_D)
log(r)
1.5 2.0 2.5 3.0 3.5
−1.2 −1.0 −0.8
−2 −1 0 1 2
−1.2 −1.1 −1.0 −0.9 −0.8 −0.7
−4 −3 −2 −1 0
0.6 0.8 1.0 1.2 1.4

Louisiana

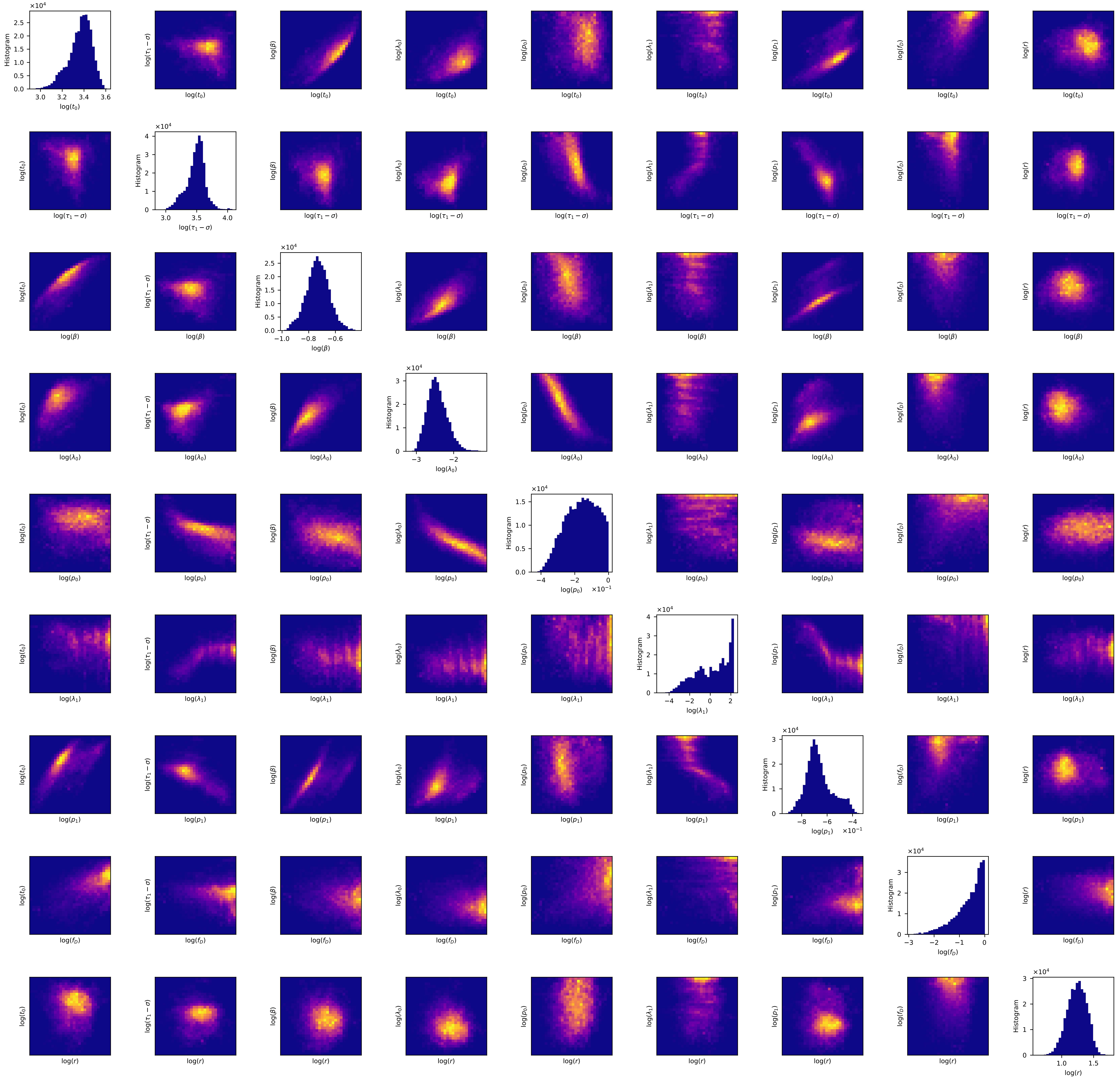

log(t_0)
log($\tau_1 - \sigma$)
log(β)
log(λ_0)
log(p_0)
log(λ_1)
log(p_1)
log(f_D)
log(r)
Histogram

Maine

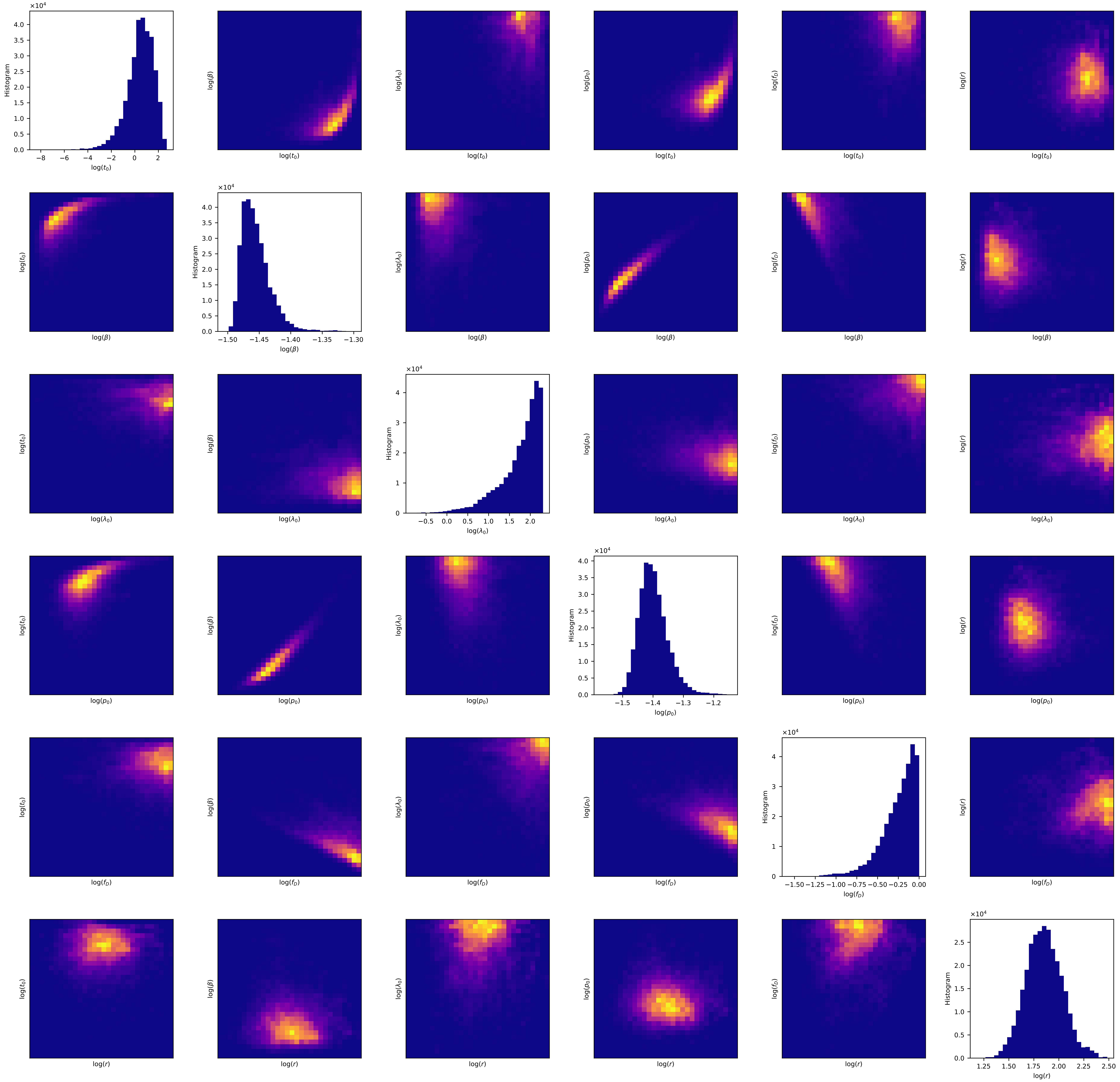

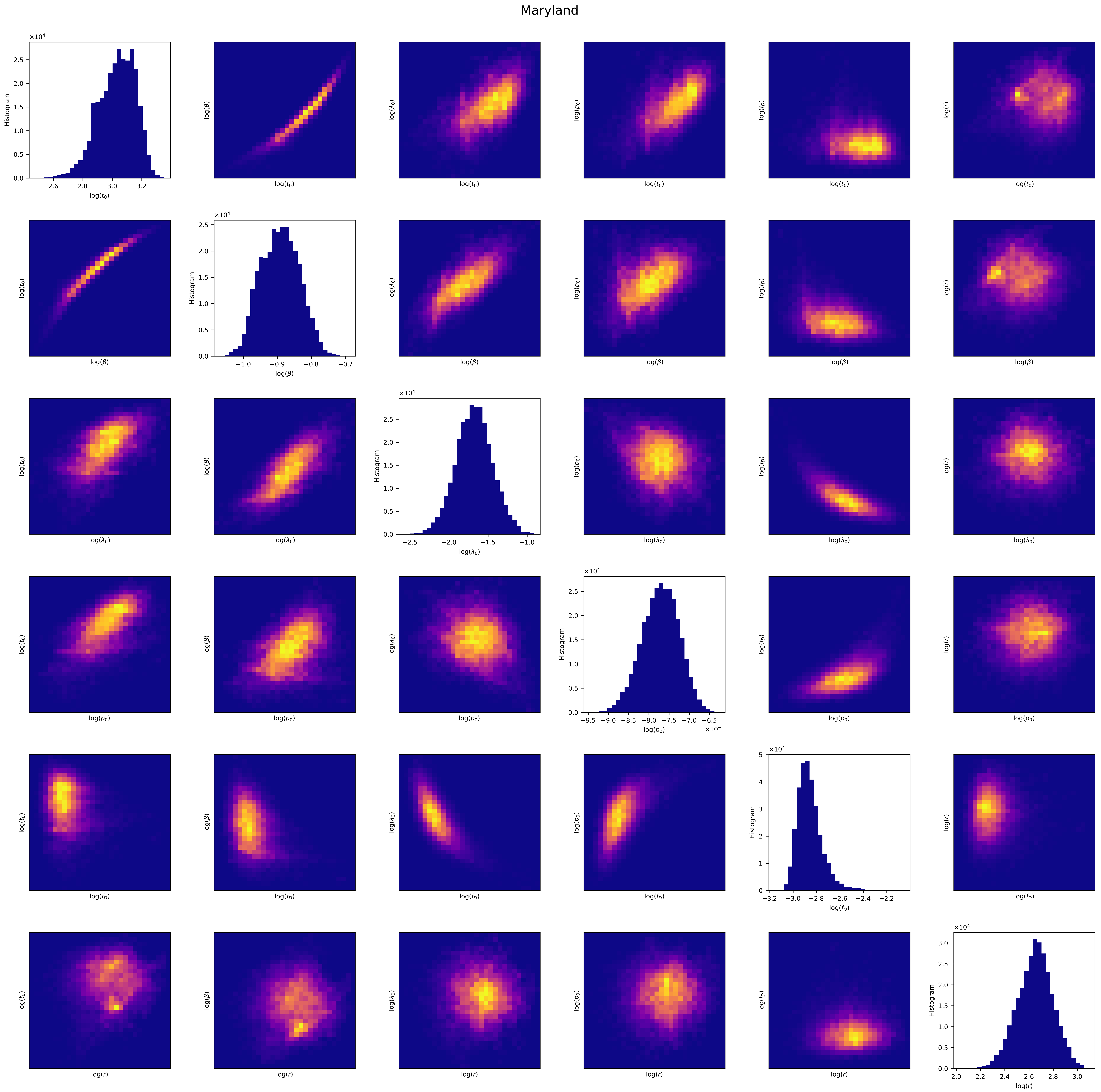
Maryland
Histogram
log(t_0)
log(β)
log(λ_0)
log(p_0)
log(f_D)
log(r)
×10^4
×10^-1

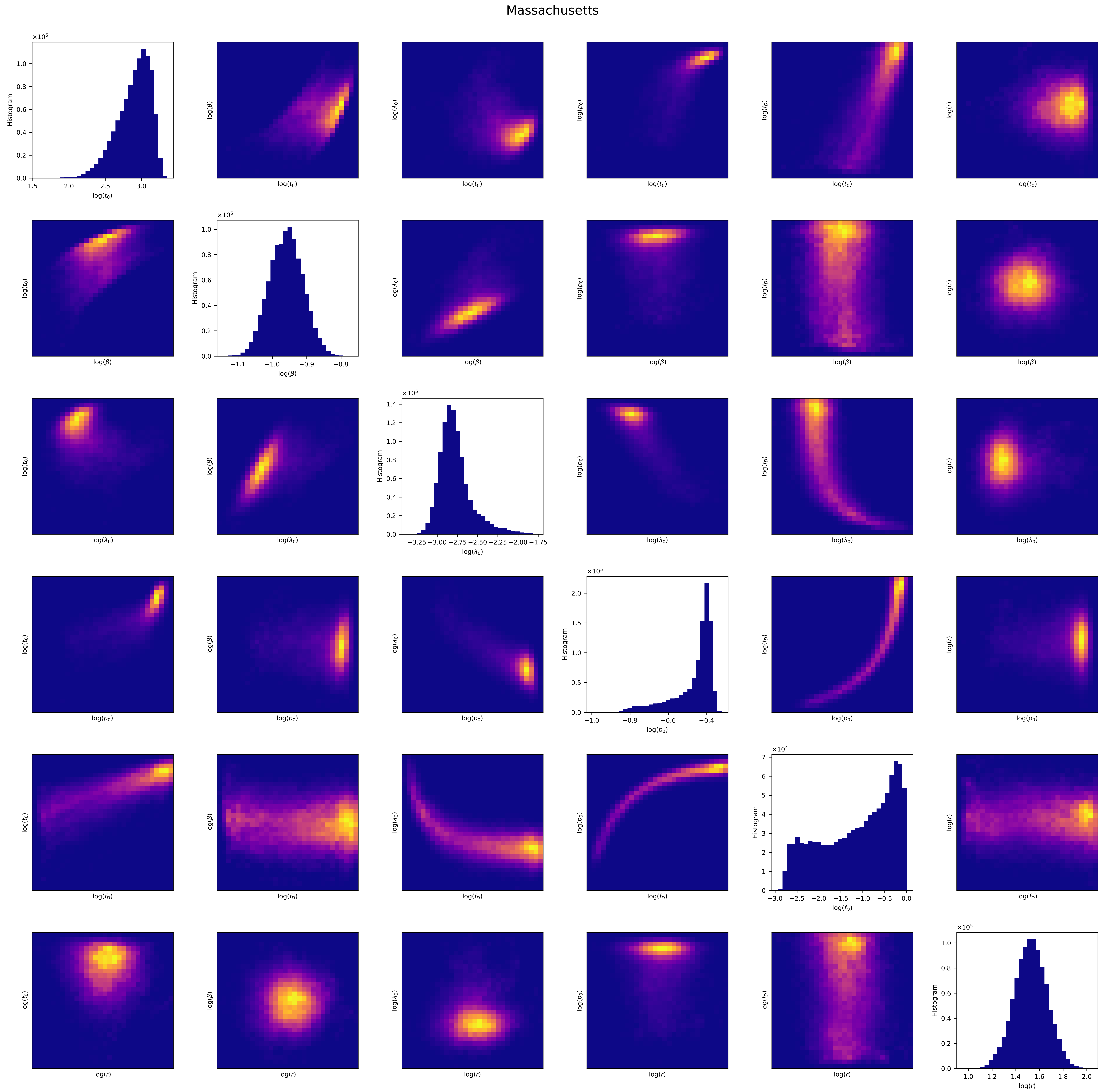
Massachusetts
Histogram
log(t_0)
log(β)
log(λ_0)
log(p_0)
log(f_D)
log(r)
×10^5
×10^4

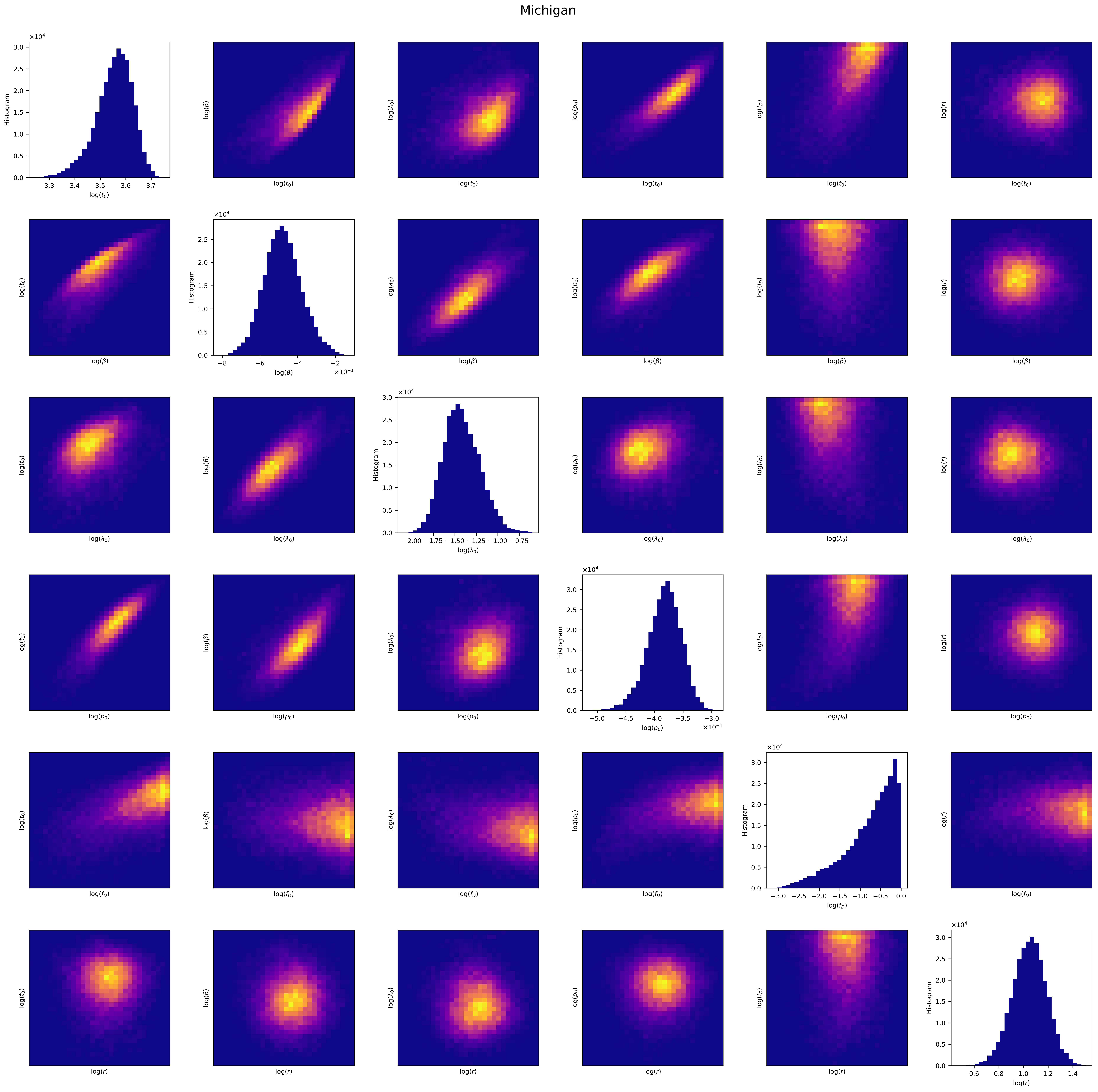

Michigan
Histogram
log(t_0)
log(β)
log(λ_0)
log(p_0)
log(f_D)
log(r)

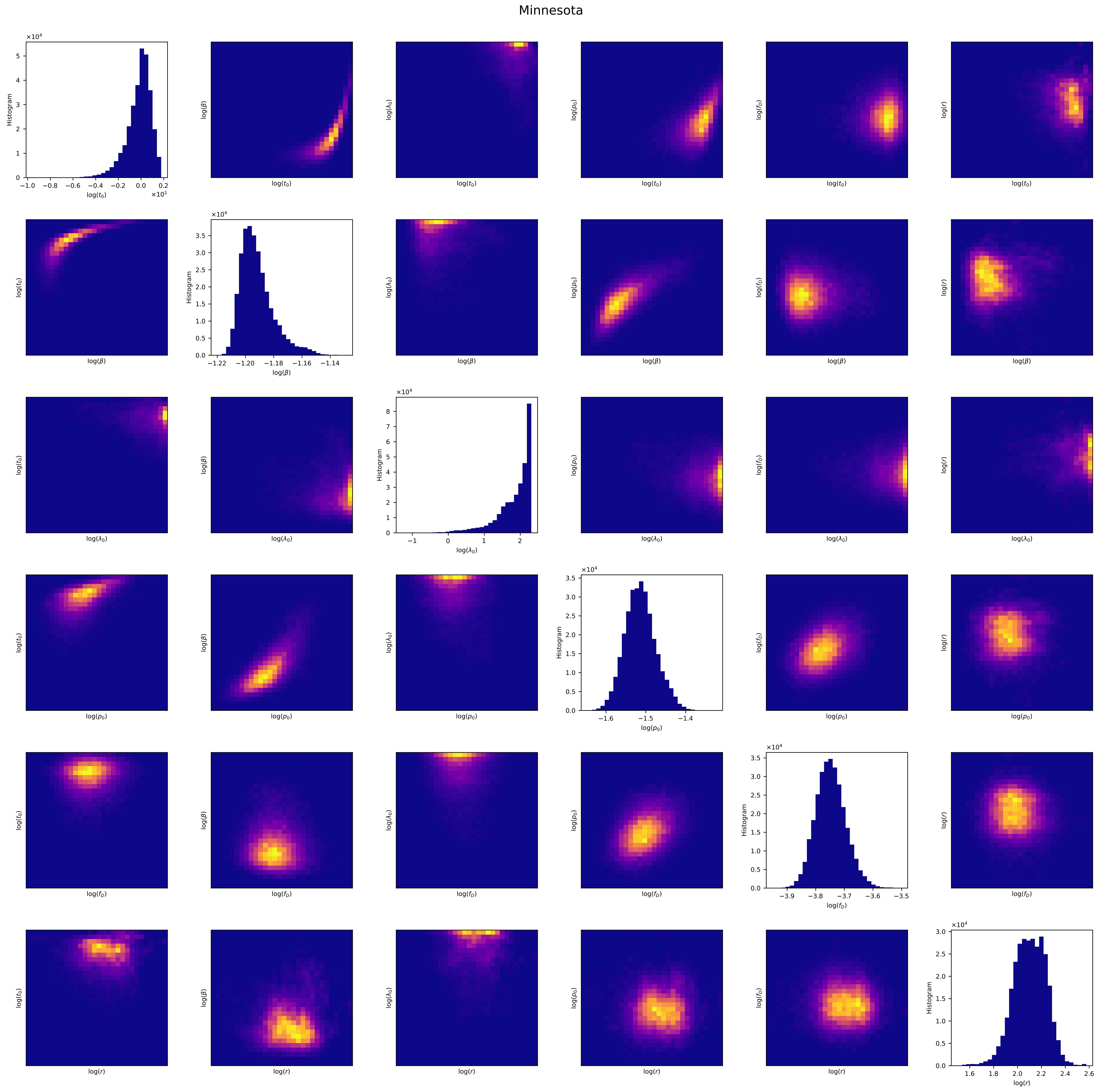
Minnesota
Histogram
log(t_0)
log(β)
log(λ_0)
log(p_0)
log(f_D)
log(r)

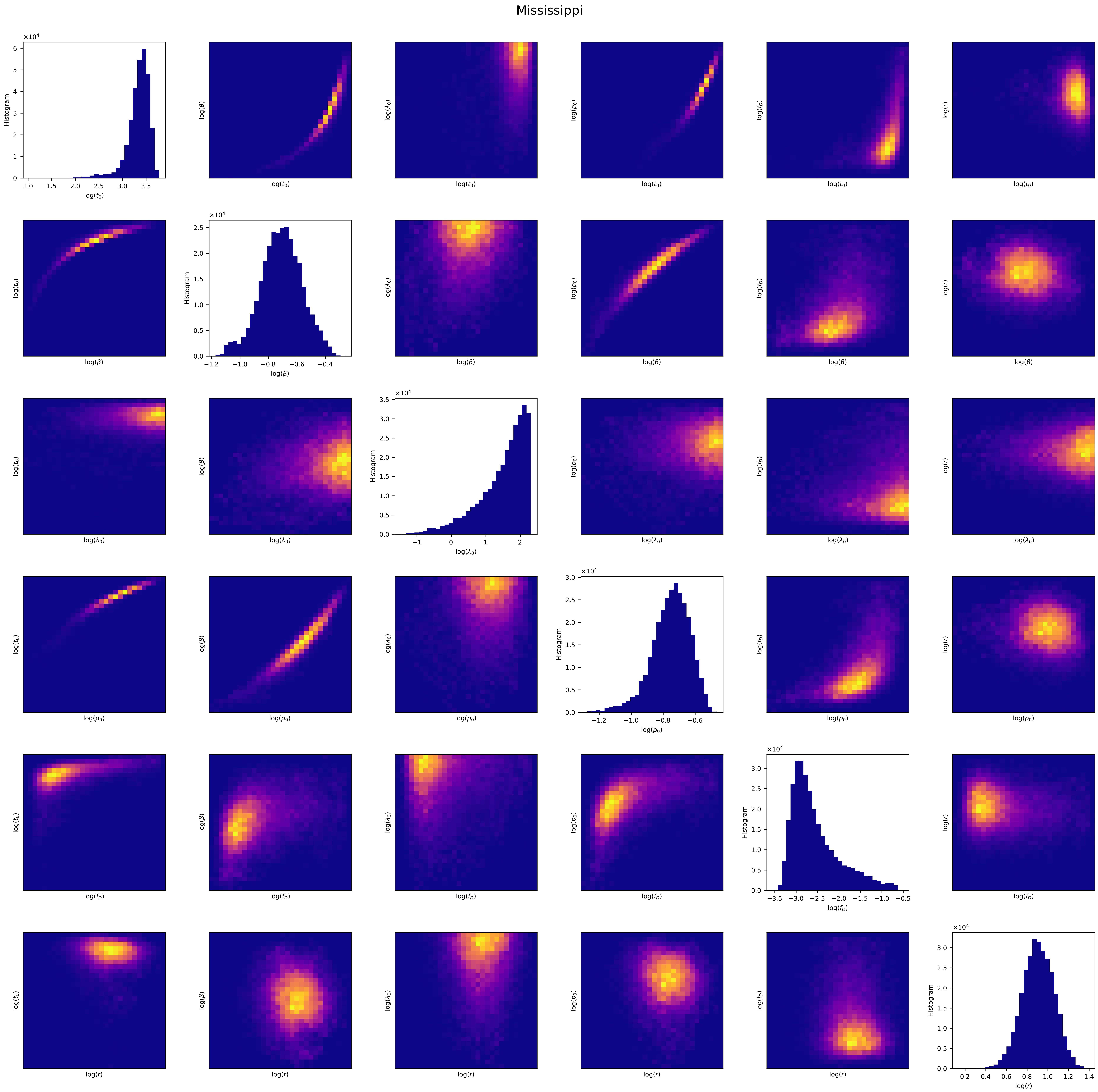
Mississippi
Histogram
log(t_0)
log(β)
log(λ_0)
log(p_0)
log(f_D)
log(r)

Missouri

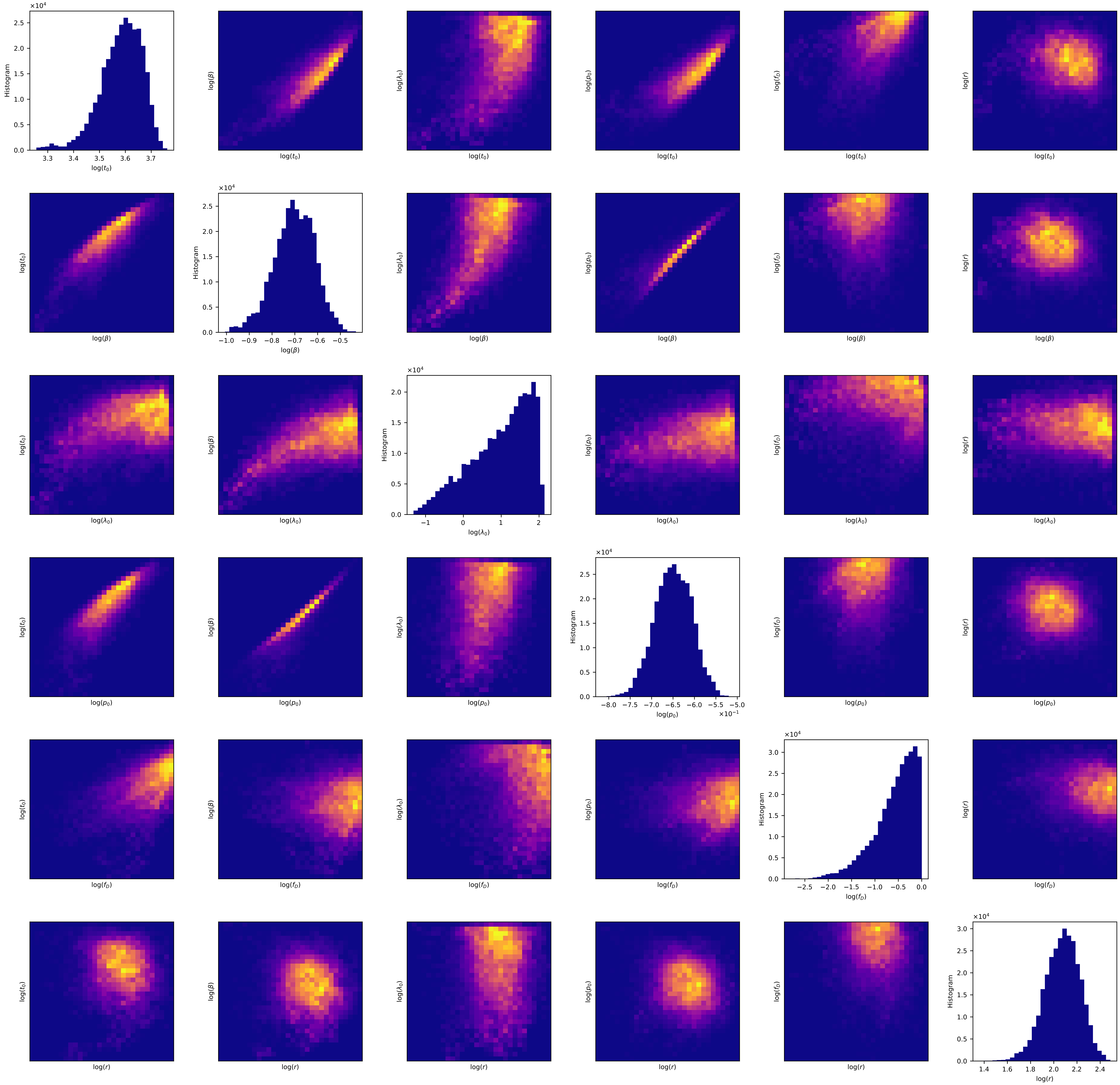

Histogram
log(t_0)
log(β)
log(λ_0)
log(p_0)
log(f_D)
log(r)
×10^4
×10^-1

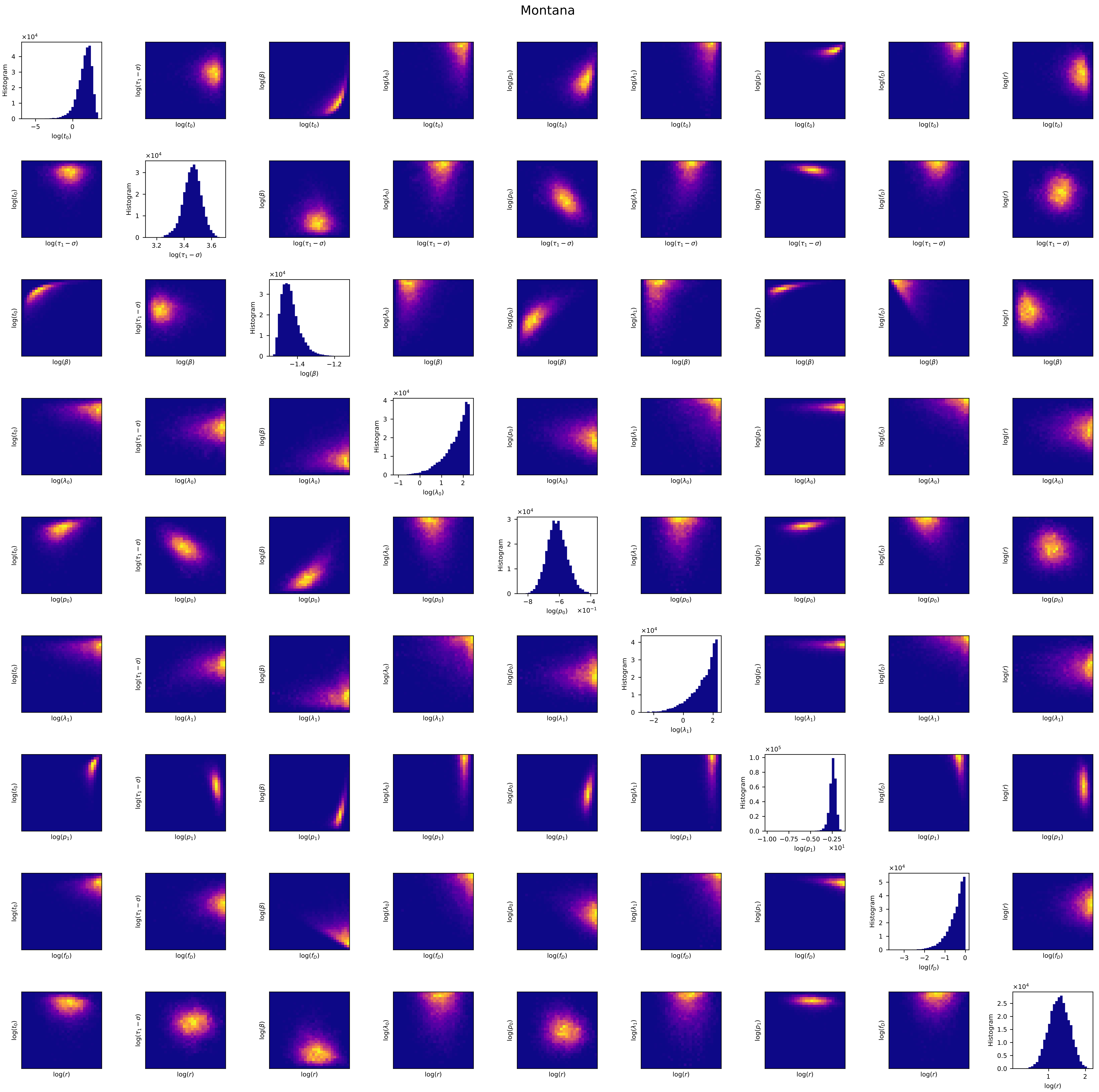
Montana
Histogram
log(t_0)
log($\tau_1 - \sigma$)
log(β)
log(λ_0)
log(p_0)
log(λ_1)
log(p_1)
log(f_D)
log(r)

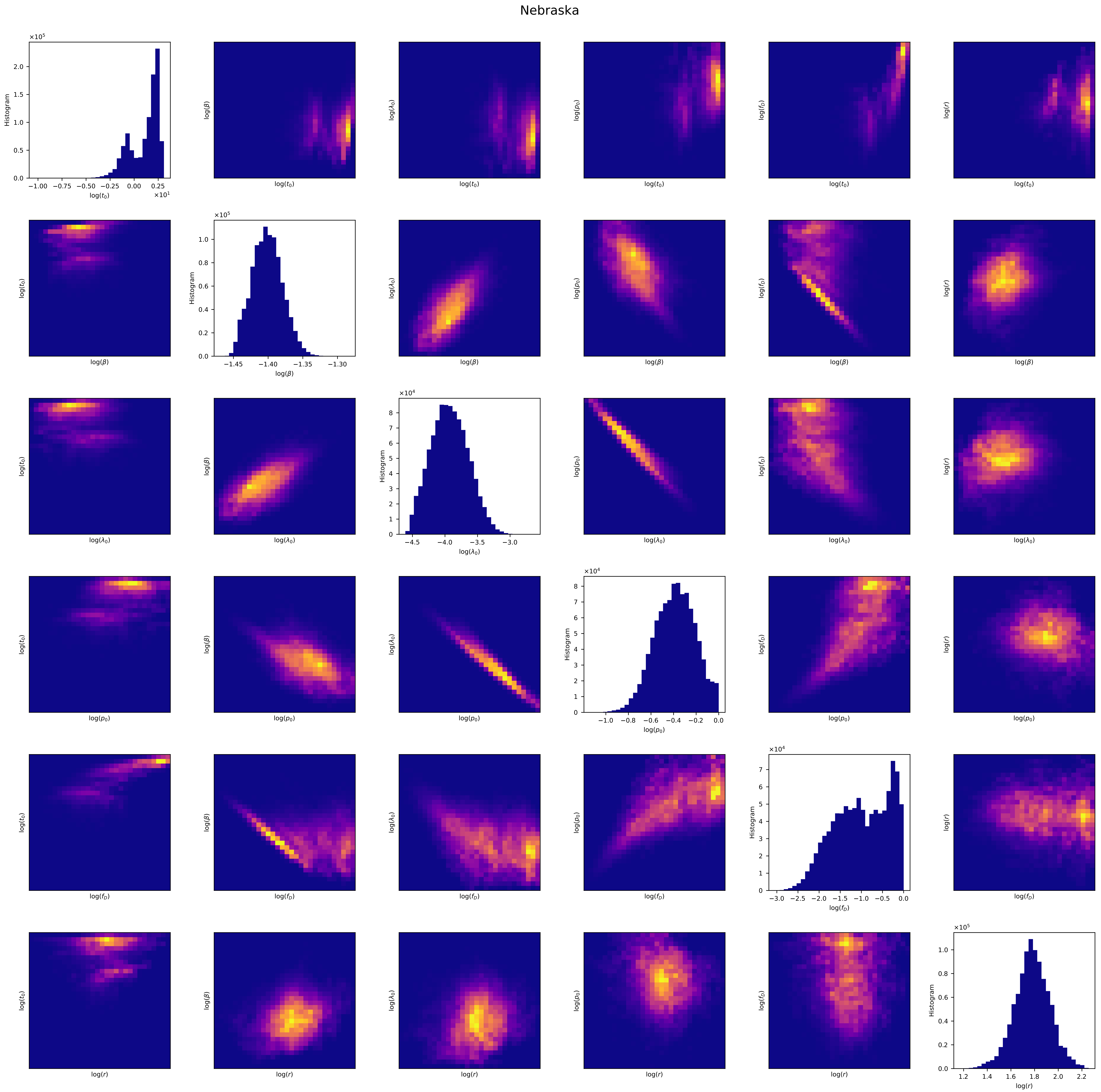
Nebraska
log(t_0)
log(β)
log(λ_0)
log(p_0)
log(f_D)
log(r)
Histogram

Nevada

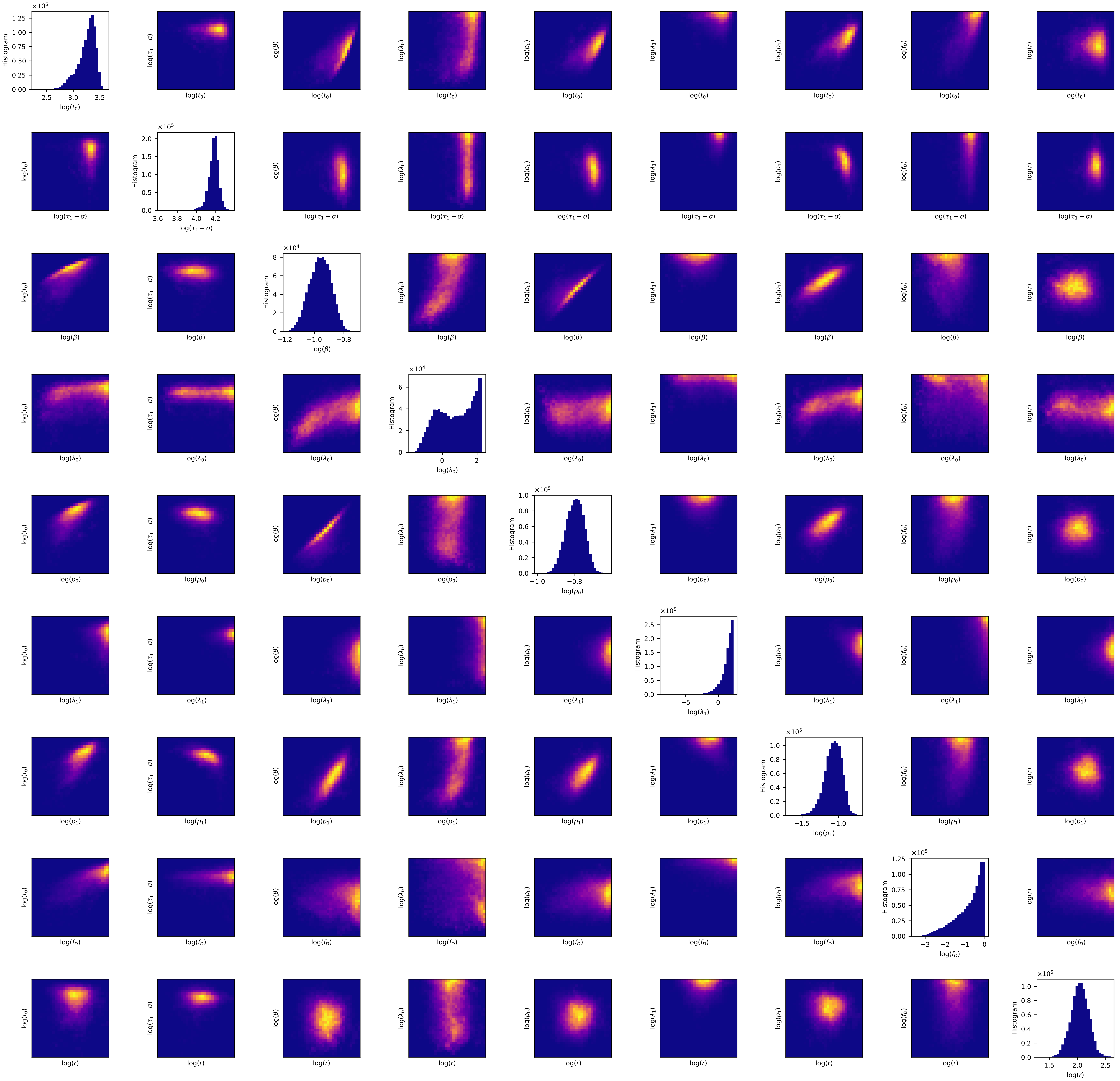

log(t_0)
log(τ_1 − σ)
log(β)
log(λ_0)
log(p_0)
log(λ_1)
log(p_1)
log(f_D)
log(r)
Histogram

New Hampshire

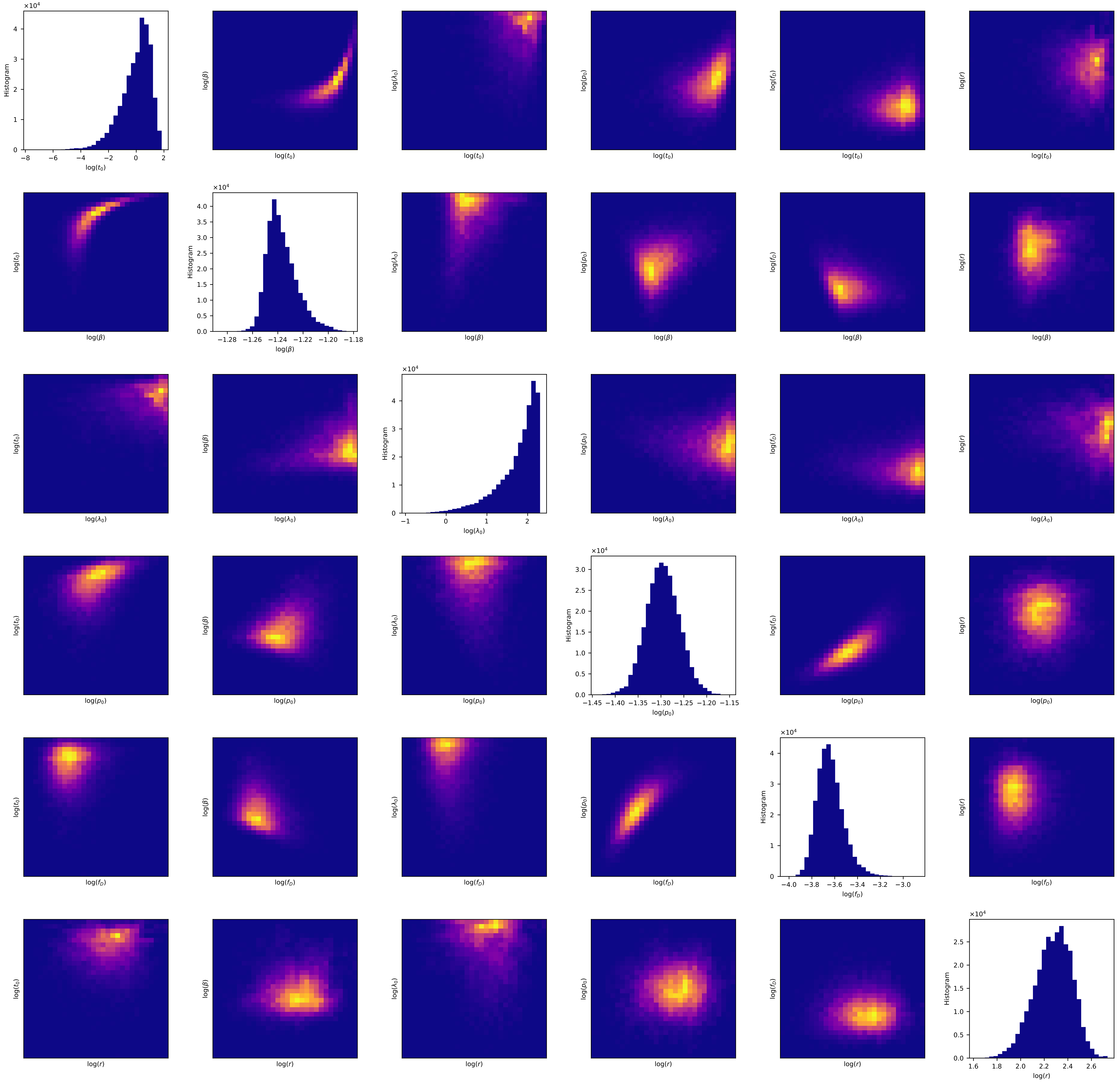

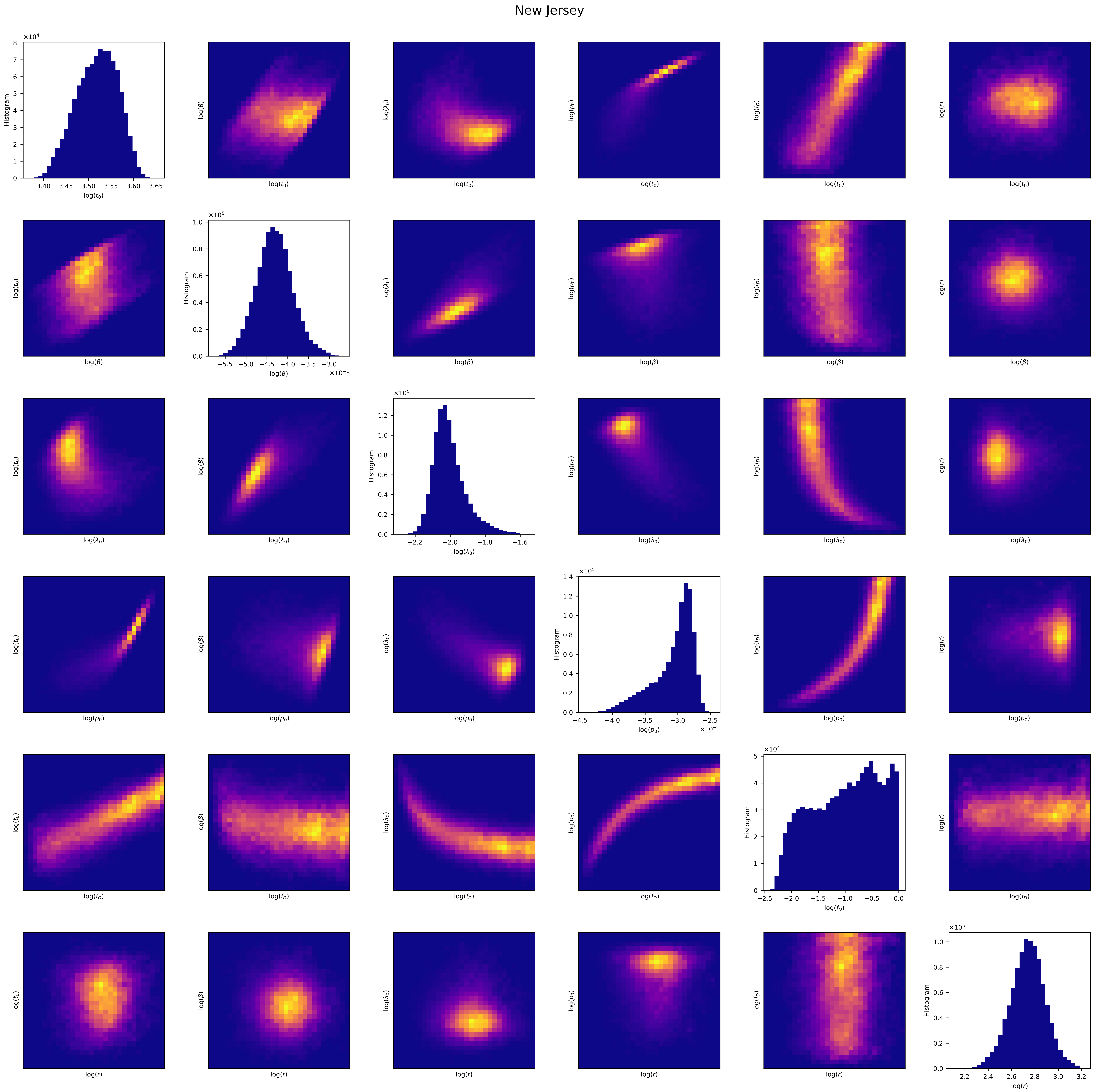
New Jersey
log(t_0)
log(β)
log(λ_0)
log(p_0)
log(f_D)
log(r)
Histogram

New Mexico

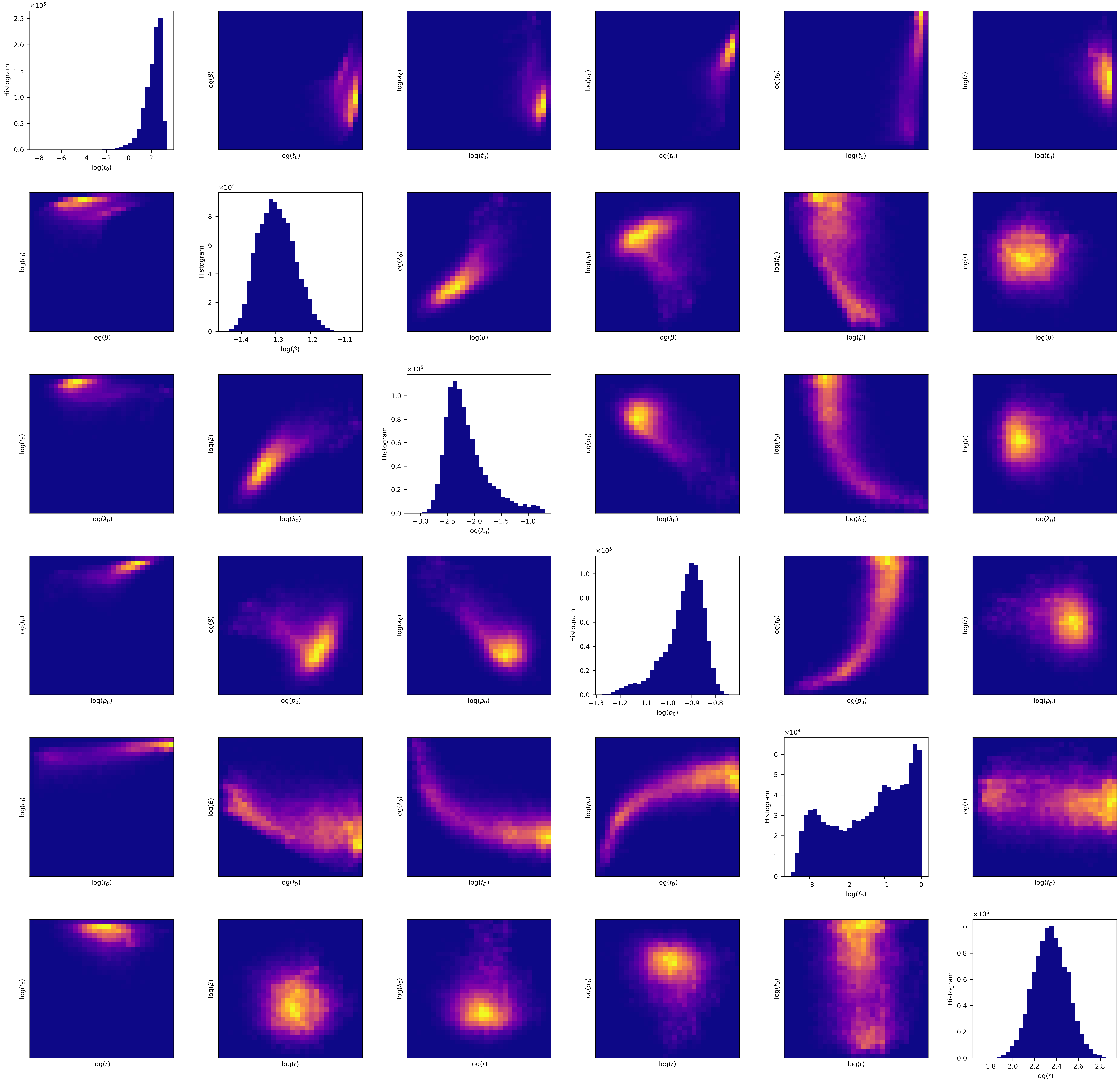

New York

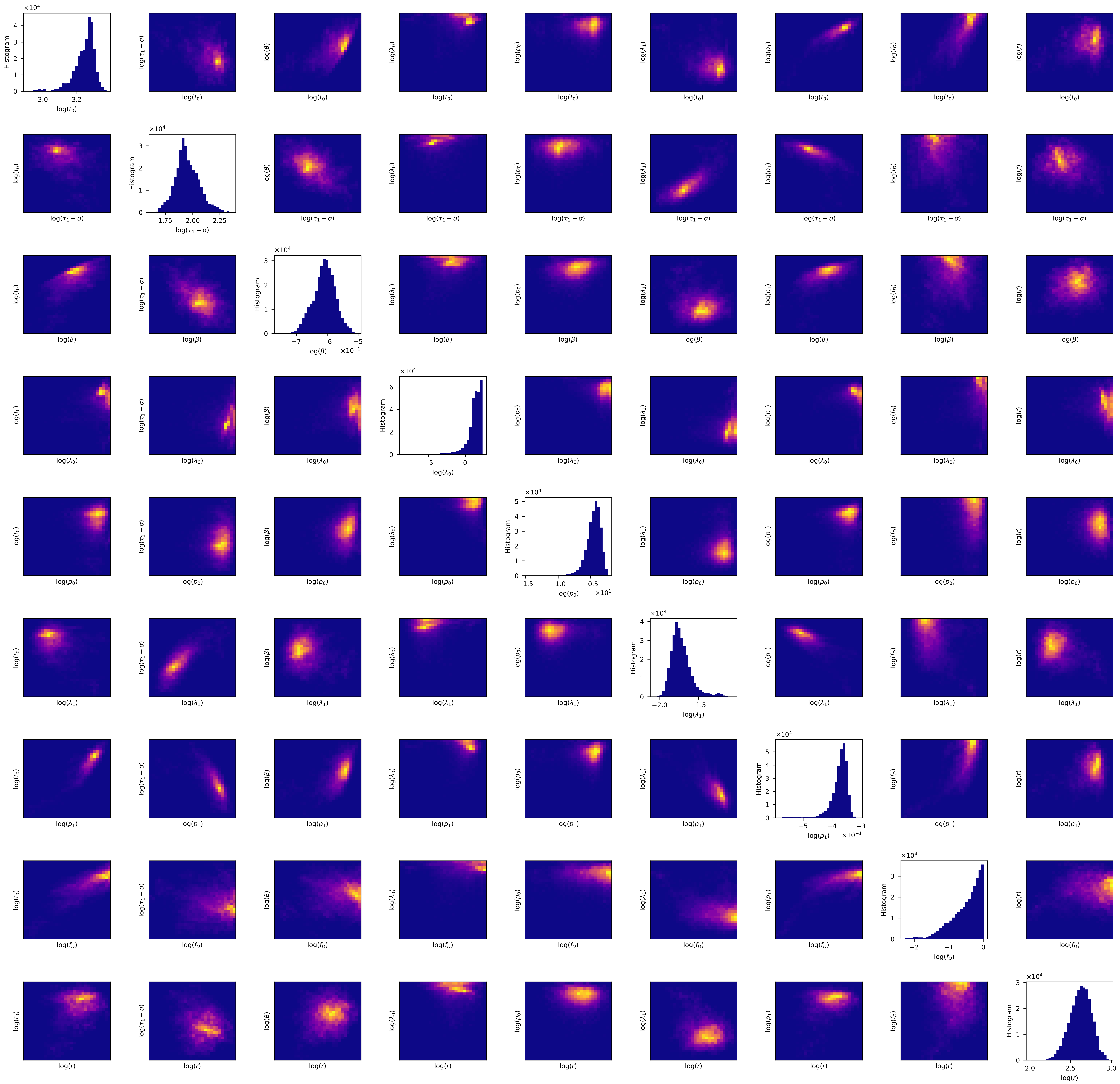

Histogram
log(t_0)
log($\tau_1 - \sigma$)
log(β)
log(λ_0)
log(p_0)
log(λ_1)
log(p_1)
log(f_D)
log(r)

North Carolina

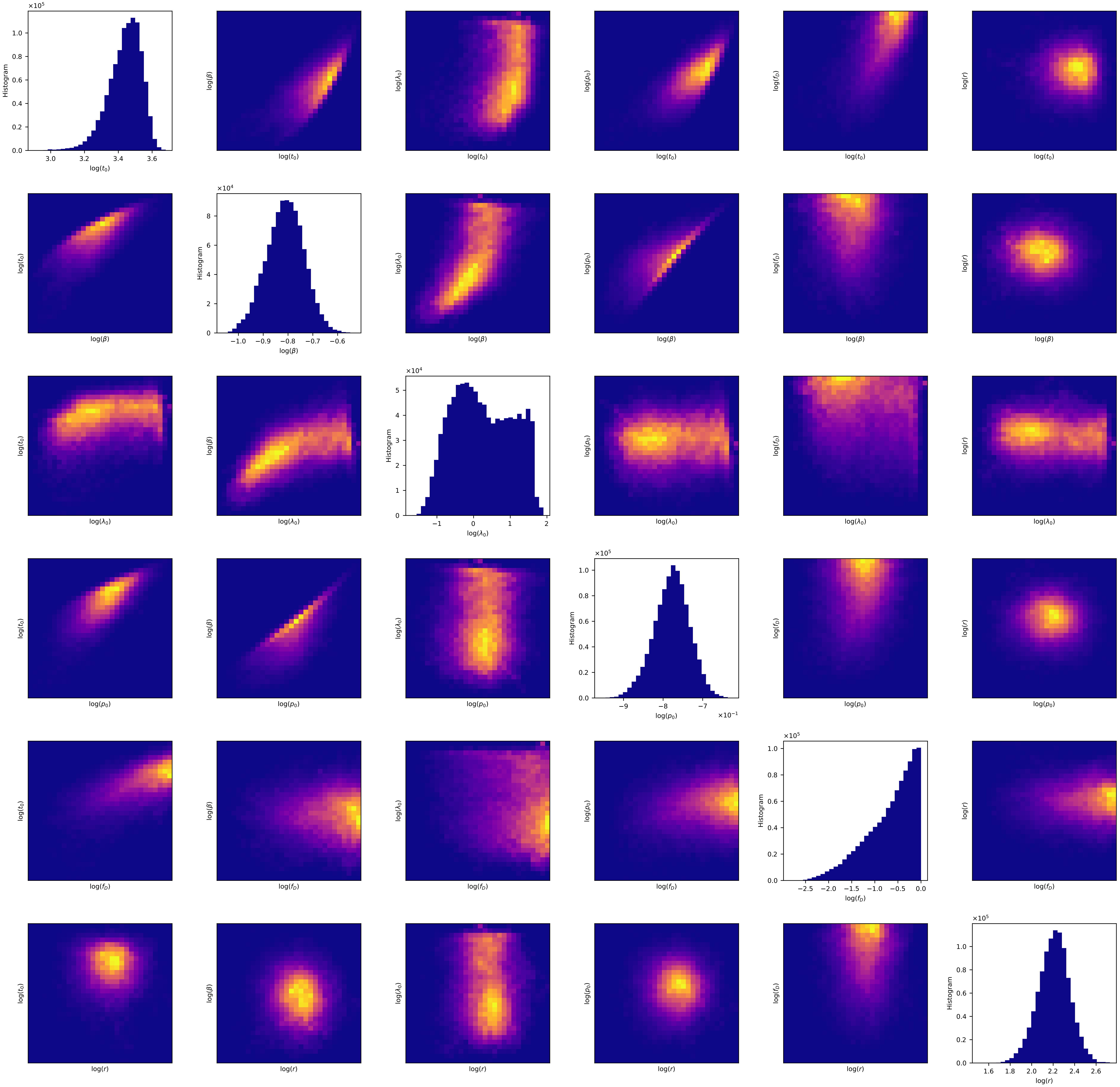

North Dakota

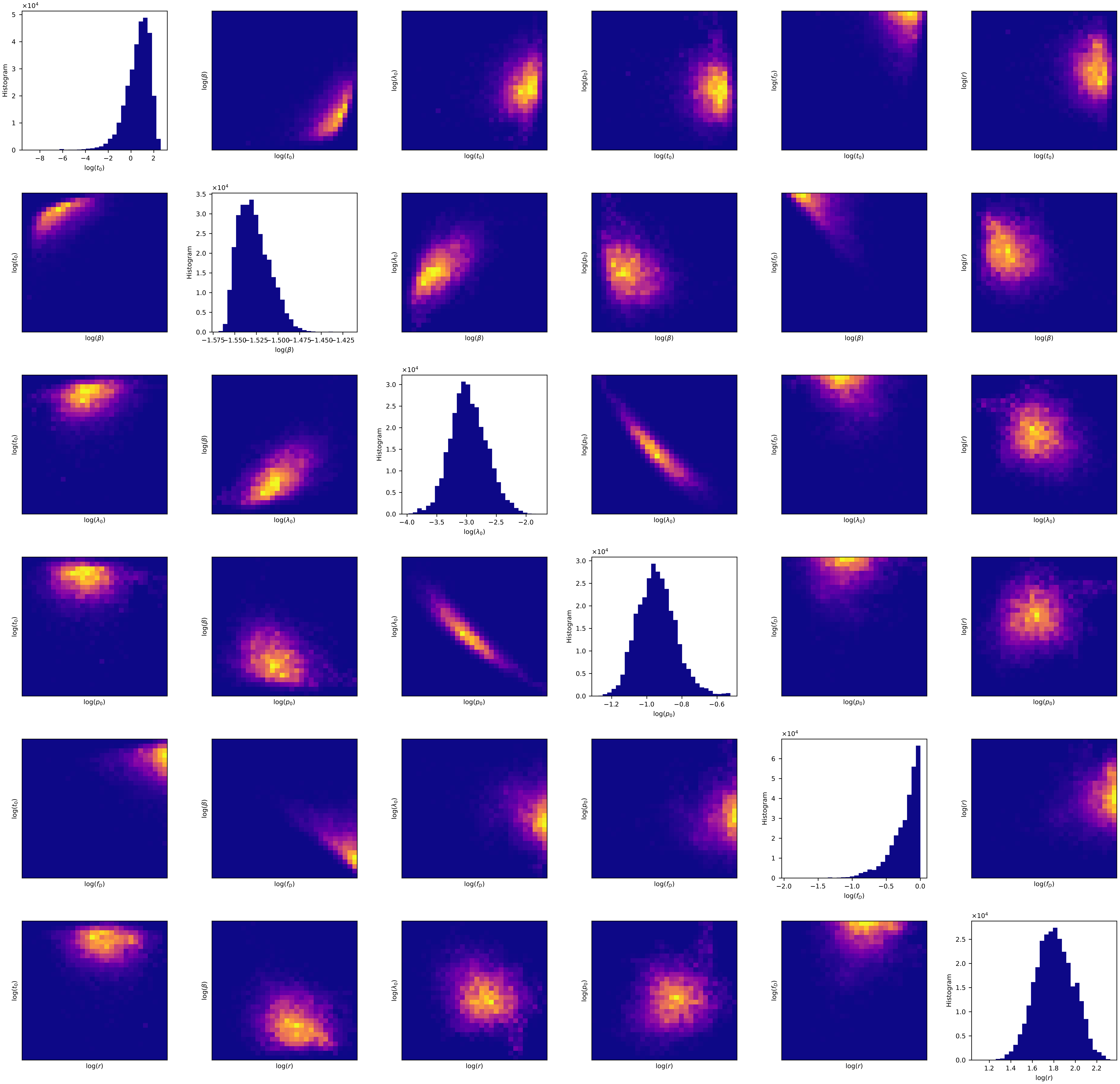

Ohio

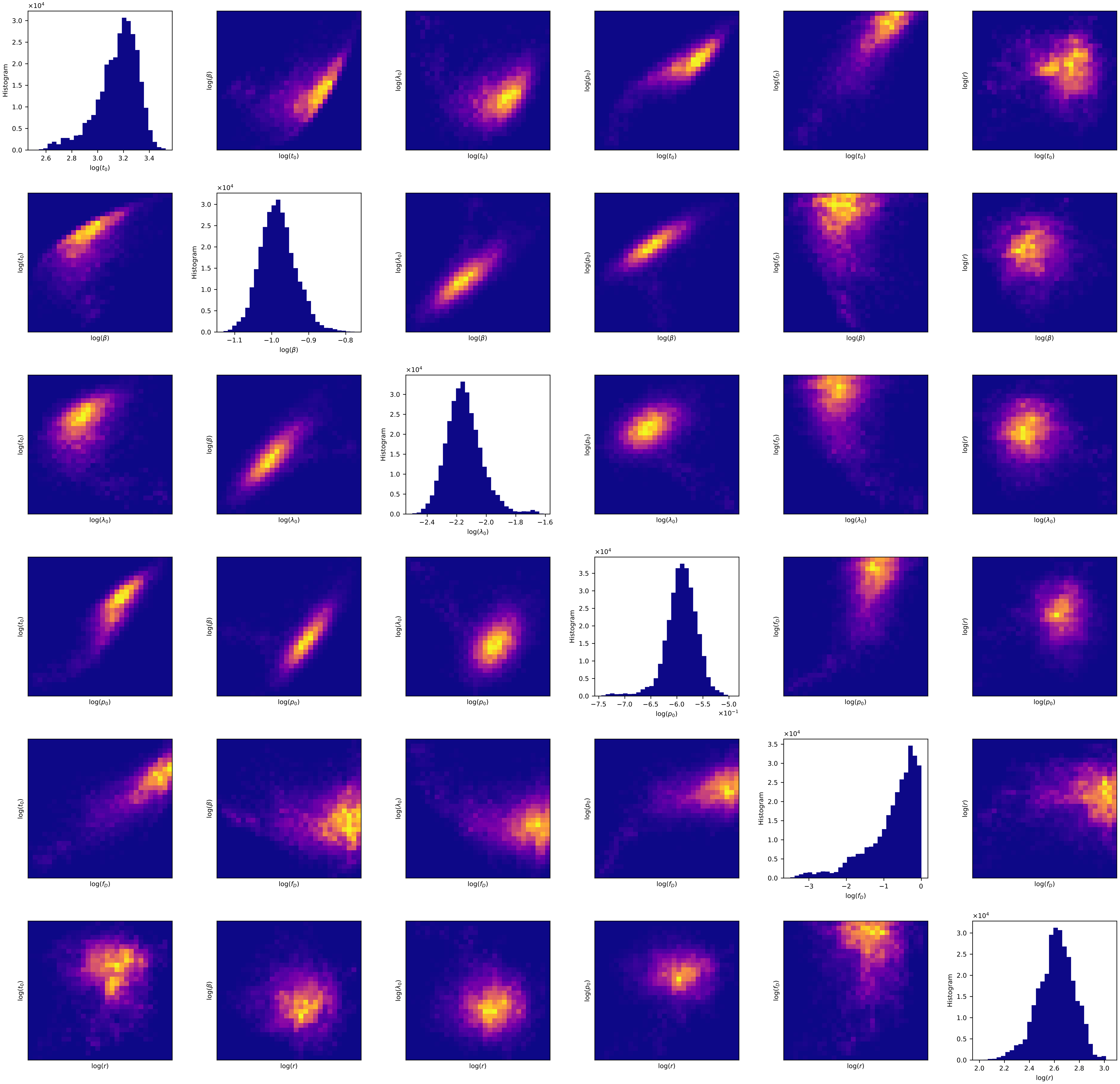

×10^4
Histogram
3.0
2.5
2.0
1.5
1.0
0.5
0.0
2.6
2.8
3.0
3.2
3.4
log(t_0)
log(β)
log(λ_0)
log(p_0)
log(f_D)
log(r)
log(t_0)
×10^4
Histogram
−1.1
−1.0
−0.9
−0.8
log(β)
×10^4
Histogram
−2.4
−2.2
−2.0
−1.8
−1.6
log(λ_0)
×10^4
Histogram
−7.5
−7.0
−6.5
−6.0
−5.5
−5.0
log(p_0)
×10^−1
×10^4
Histogram
−3
−2
−1
0
log(f_D)
×10^4
Histogram
2.0
2.2
2.4
2.6
2.8
3.0
log(r)

Oklahoma

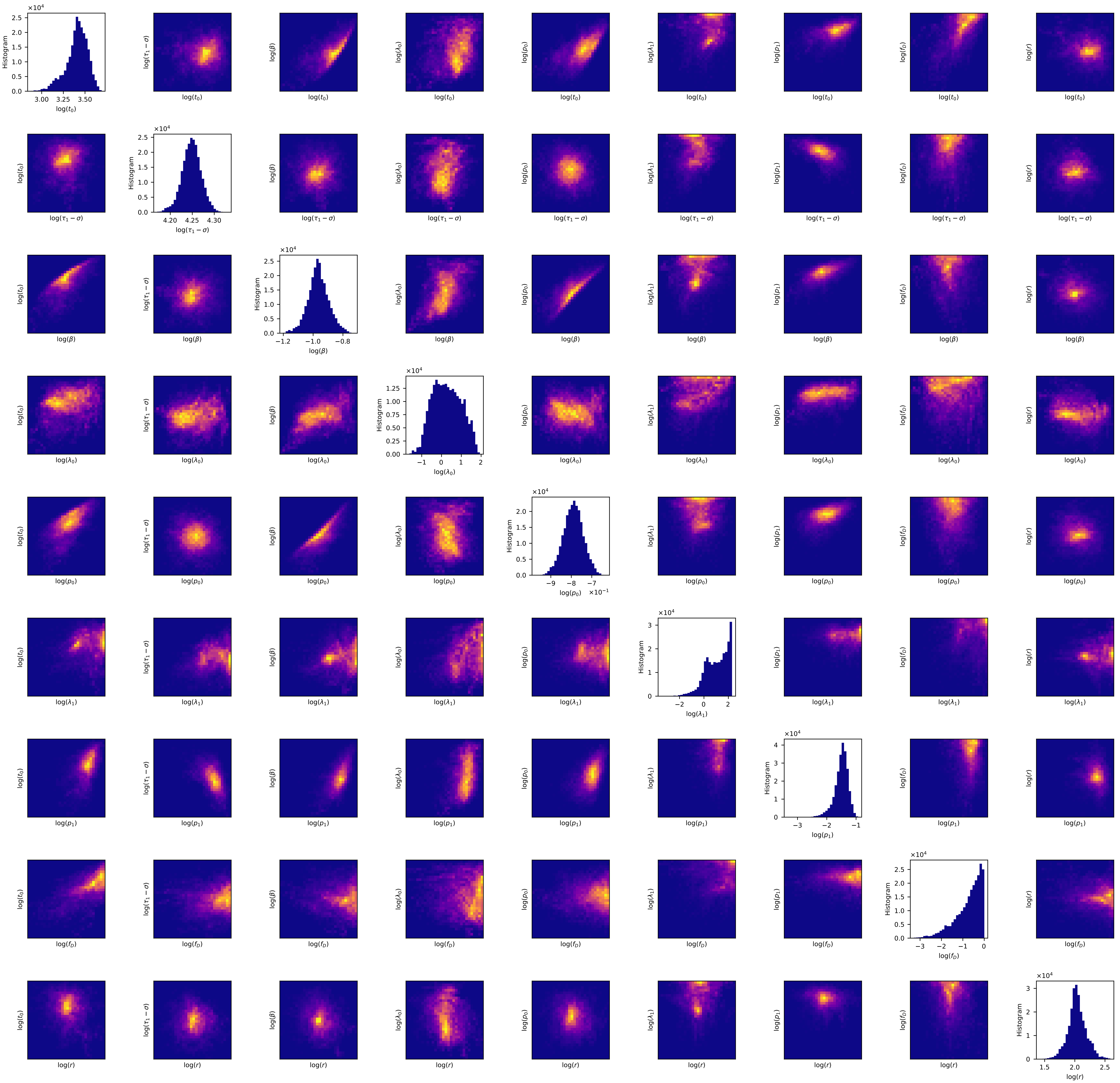

Histogram
log(t_0)
log($\tau_1 - \sigma$)
log(β)
log(λ_0)
log(p_0)
log(λ_1)
log(p_1)
log(f_D)
log(r)

Oregon

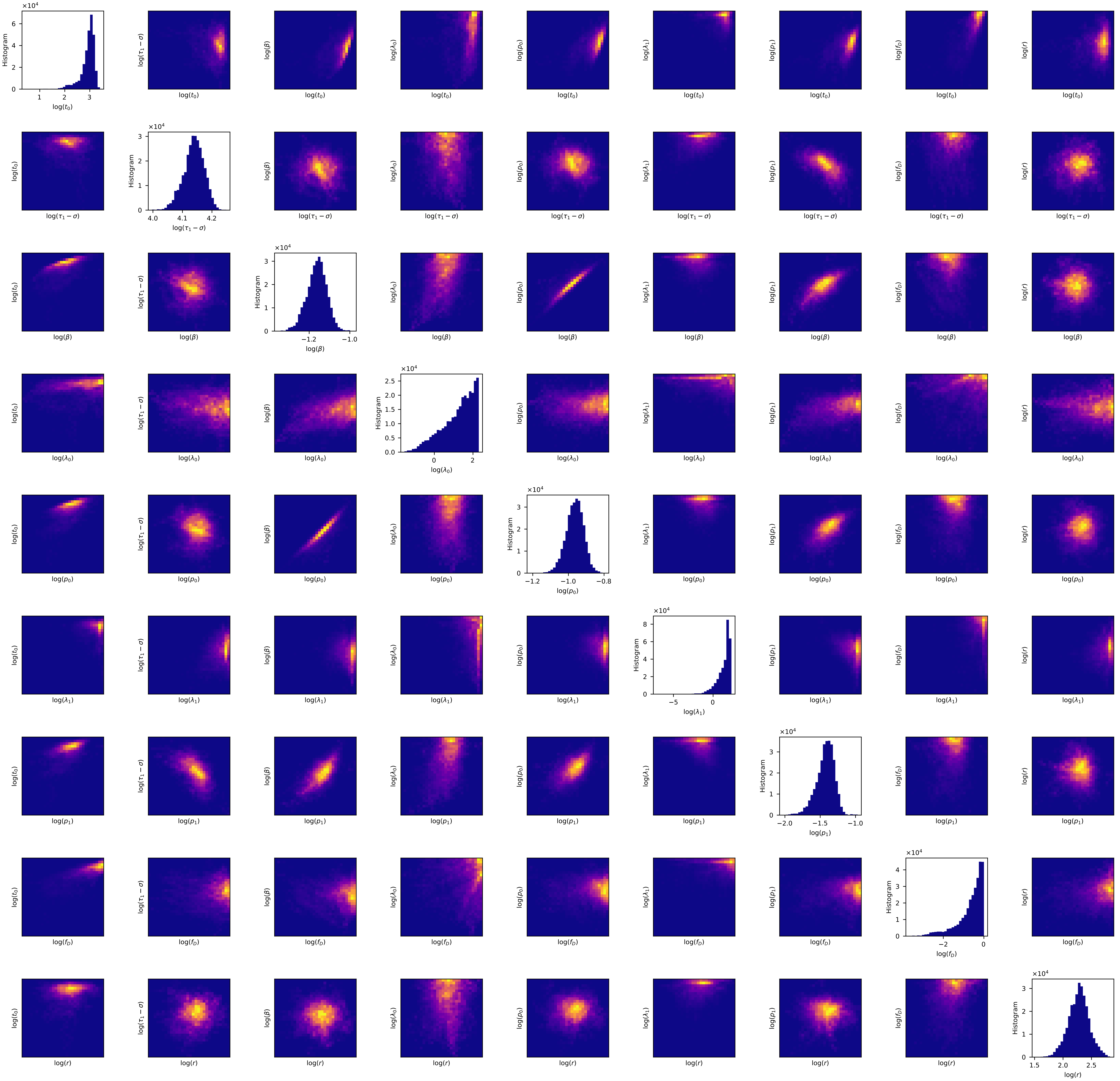

Histogram
$\times10^4$
$\log(t_0)$
$\log(\tau_1-\sigma)$
$\log(\beta)$
$\log(\lambda_0)$
$\log(p_0)$
$\log(\lambda_1)$
$\log(p_1)$
$\log(f_D)$
$\log(r)$

Pennsylvania

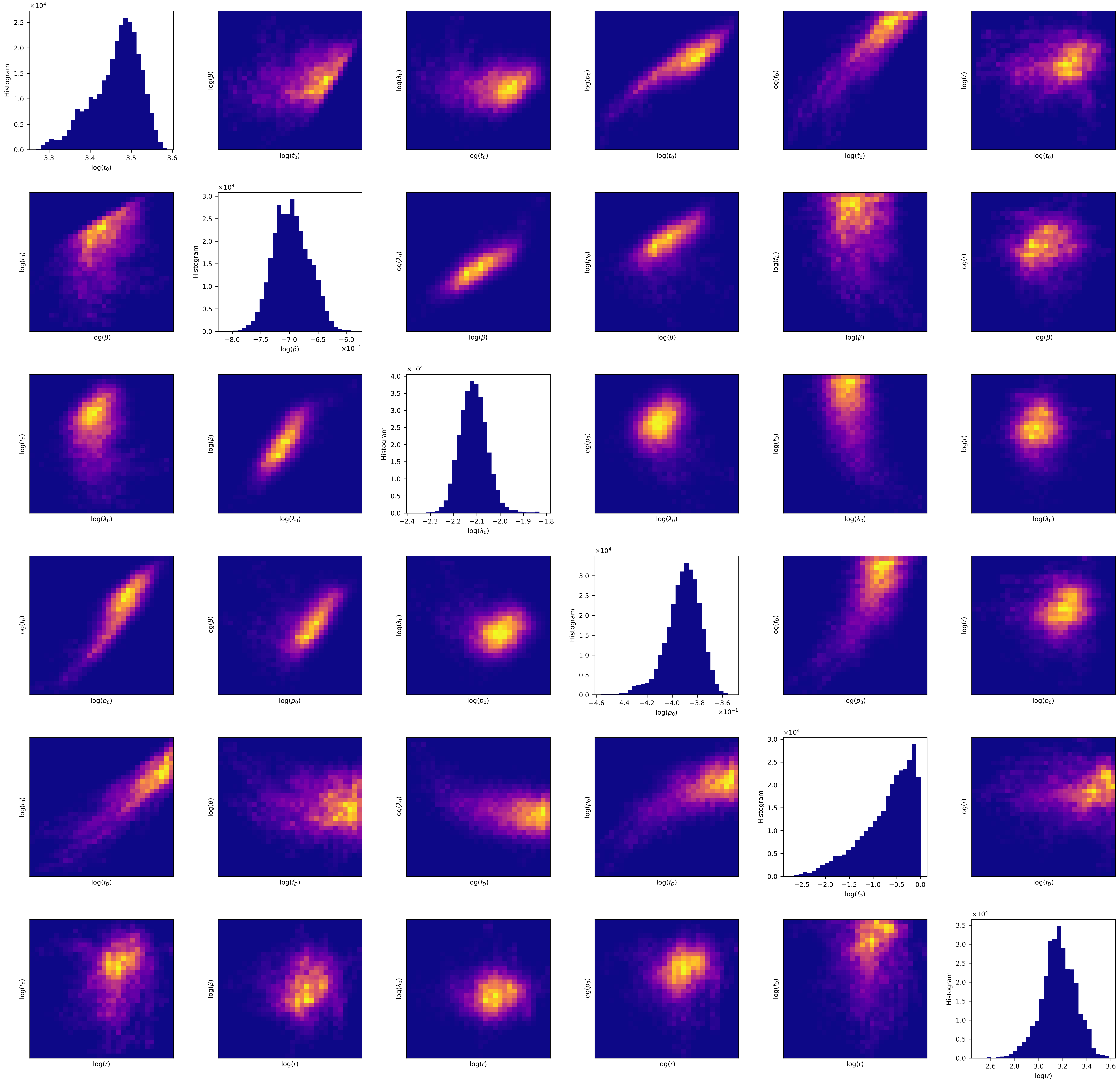

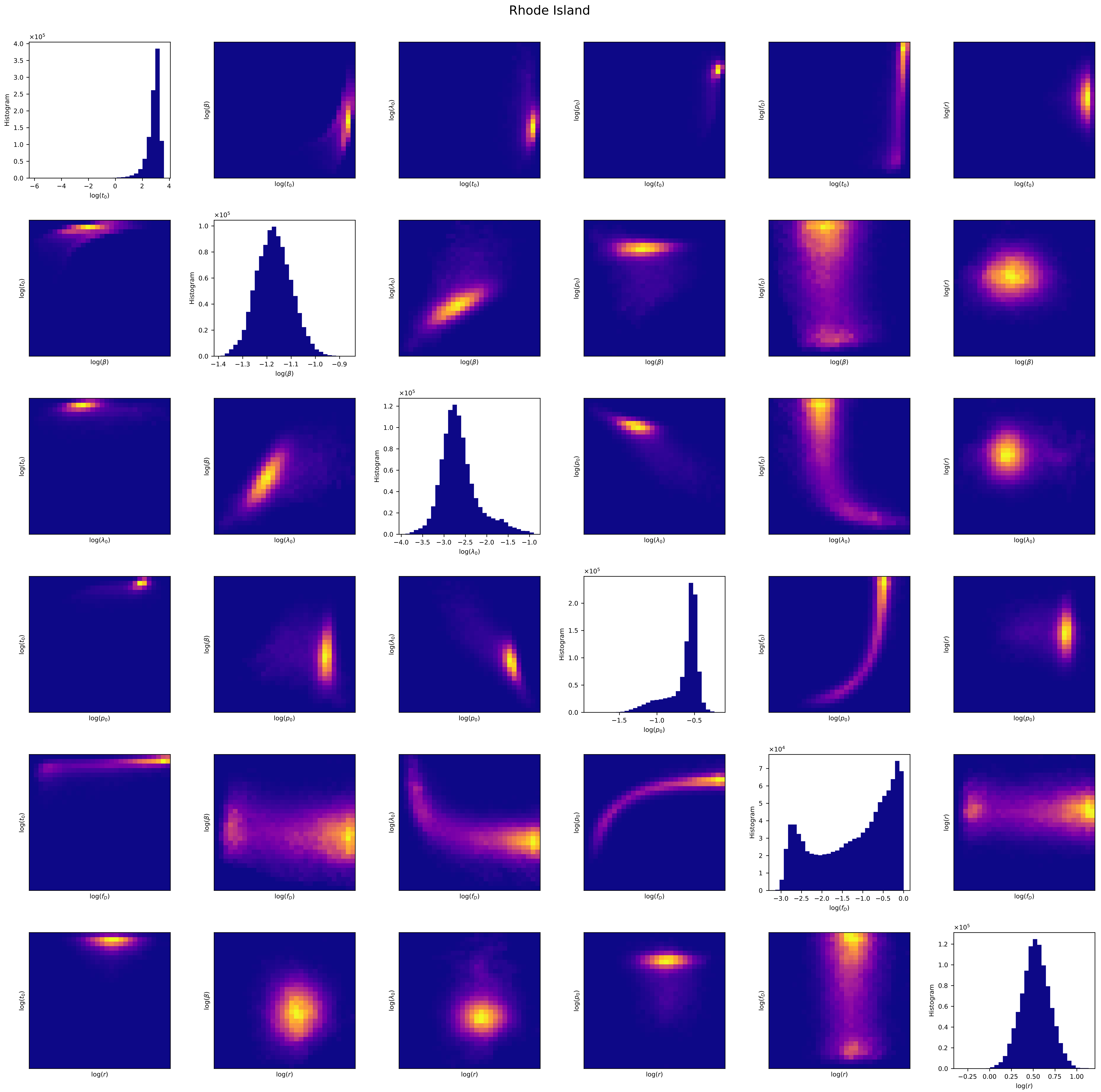

Rhode Island
Histogram
log(t_0)
log(β)
log(λ_0)
log(p_0)
log(f_D)
log(r)

South Carolina

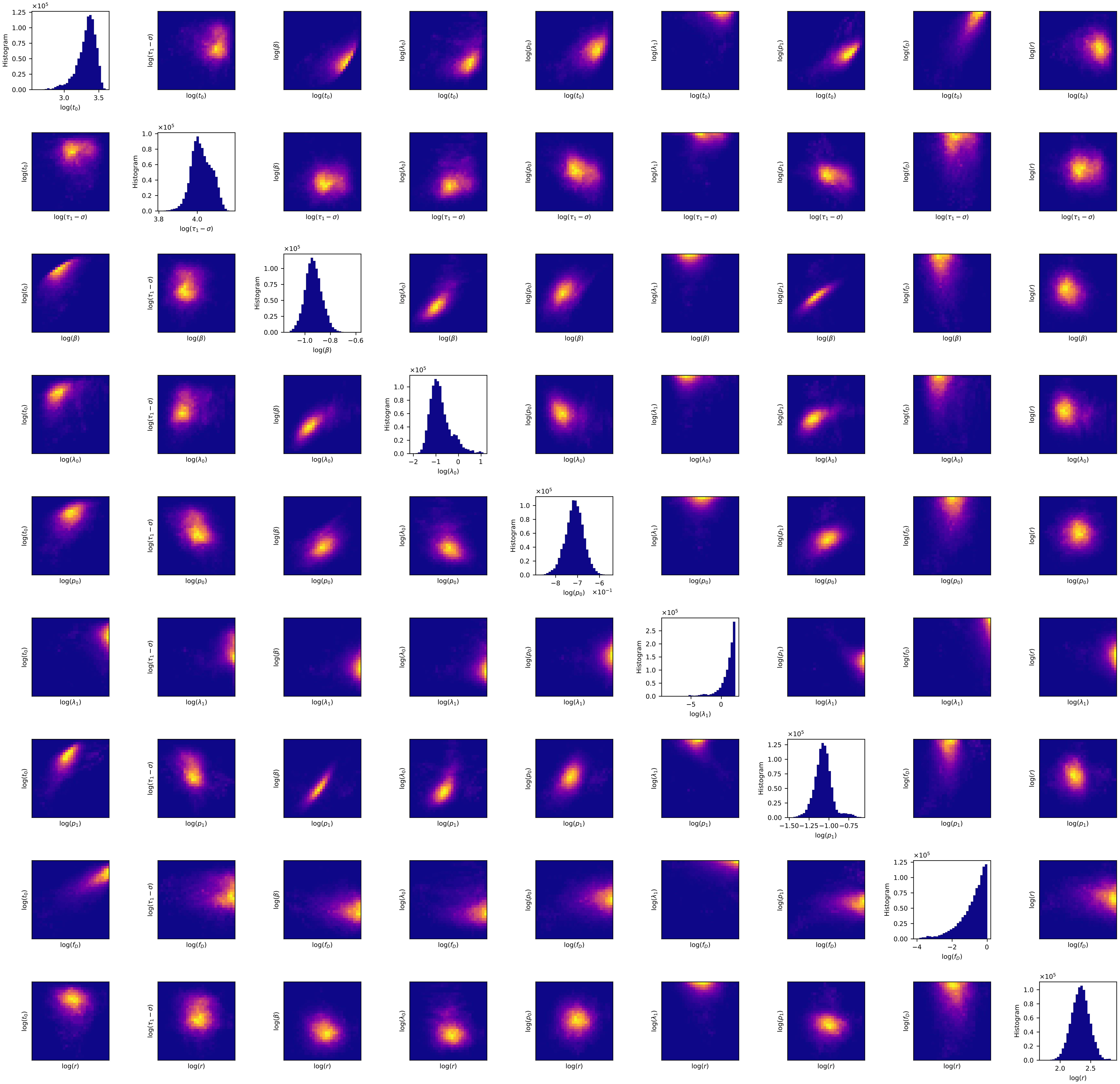

South Dakota

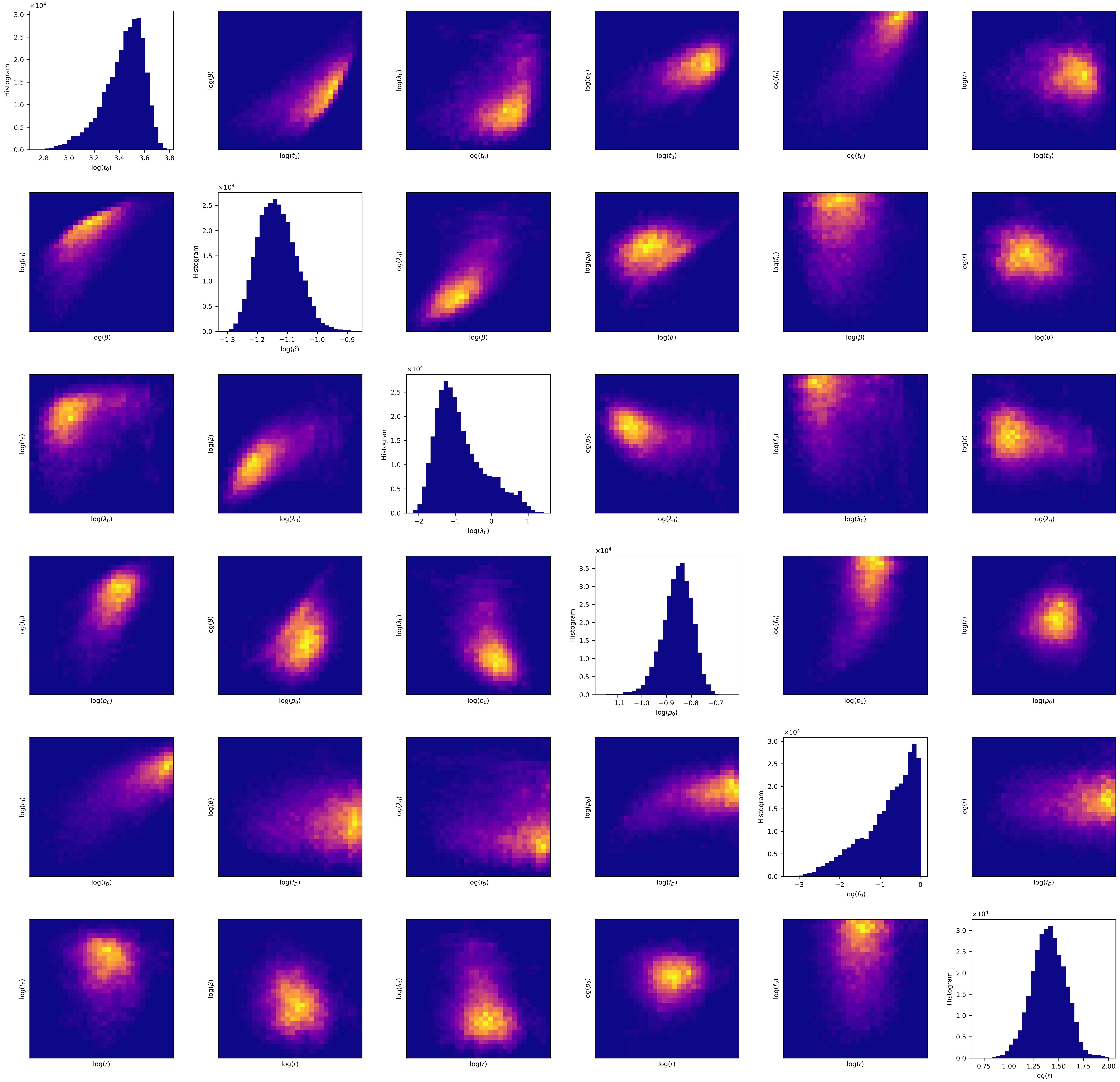

Tennessee

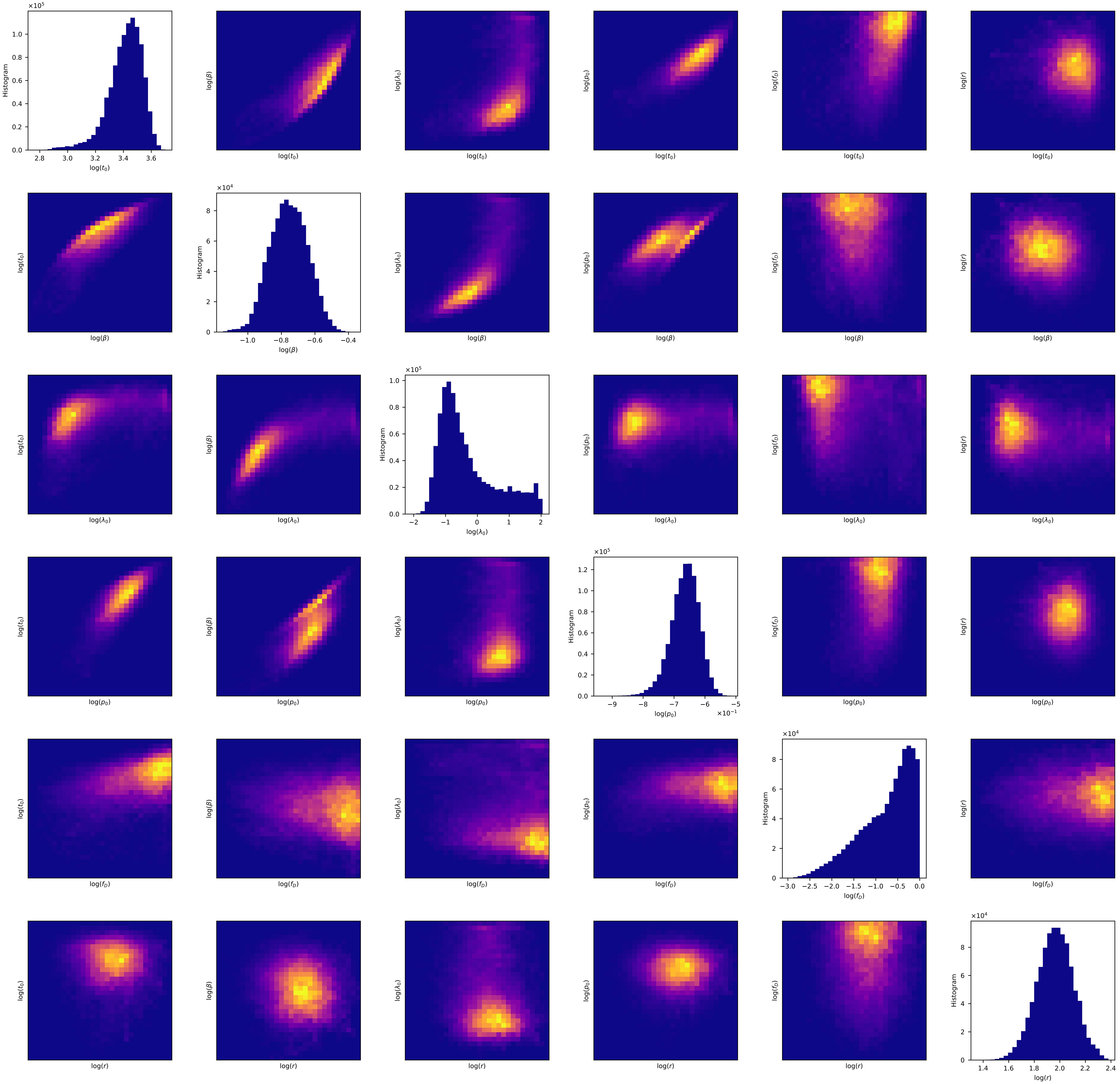

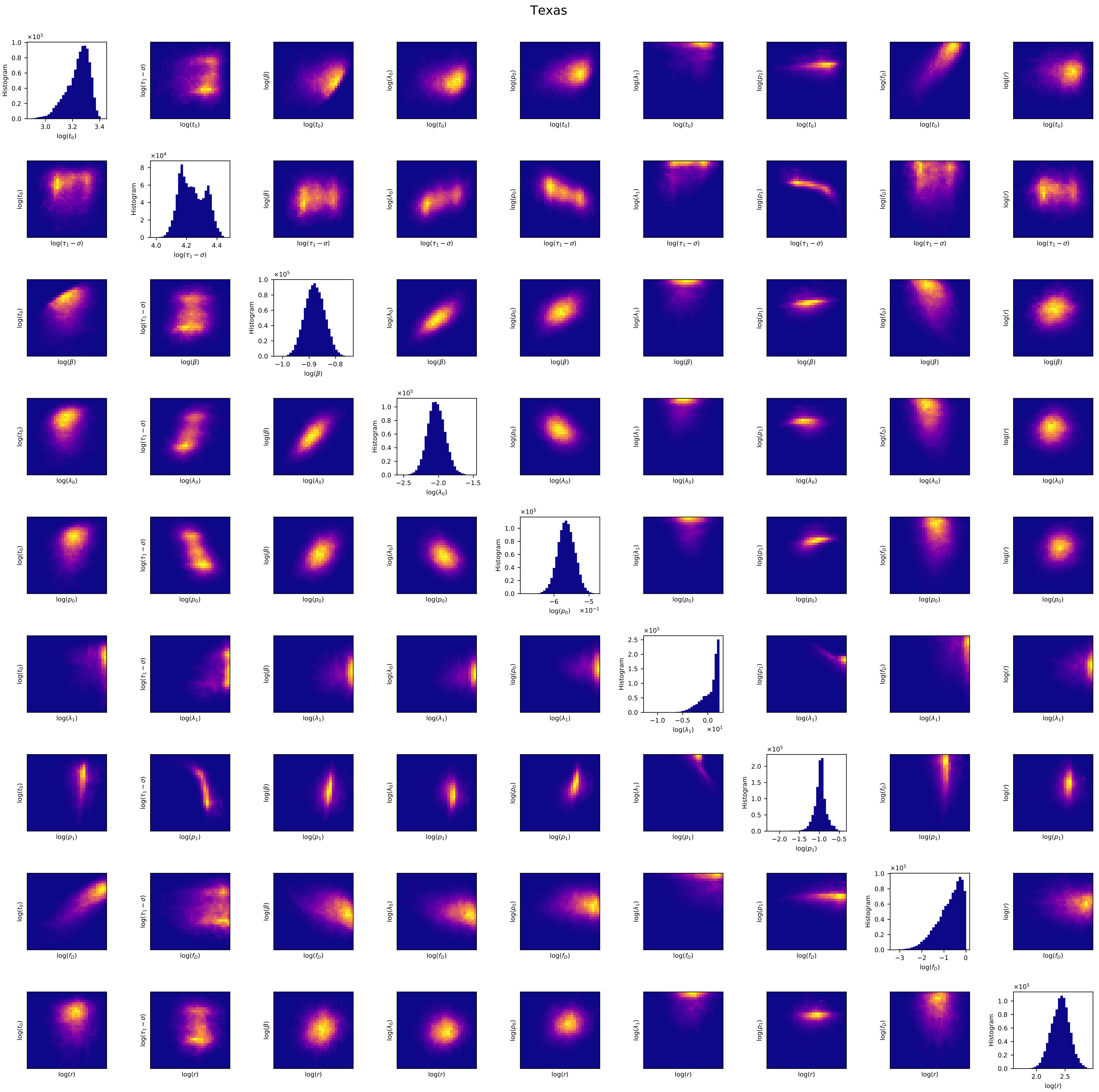
Texas
Histogram
log(t_0)
log($\tau_1 - \sigma$)
log(β)
log(λ_0)
log(p_0)
log(λ_1)
log(p_1)
log(f_D)
log(r)
$\times 10^5$
$\times 10^4$
$\times 10^{-1}$
$\times 10^1$

Utah

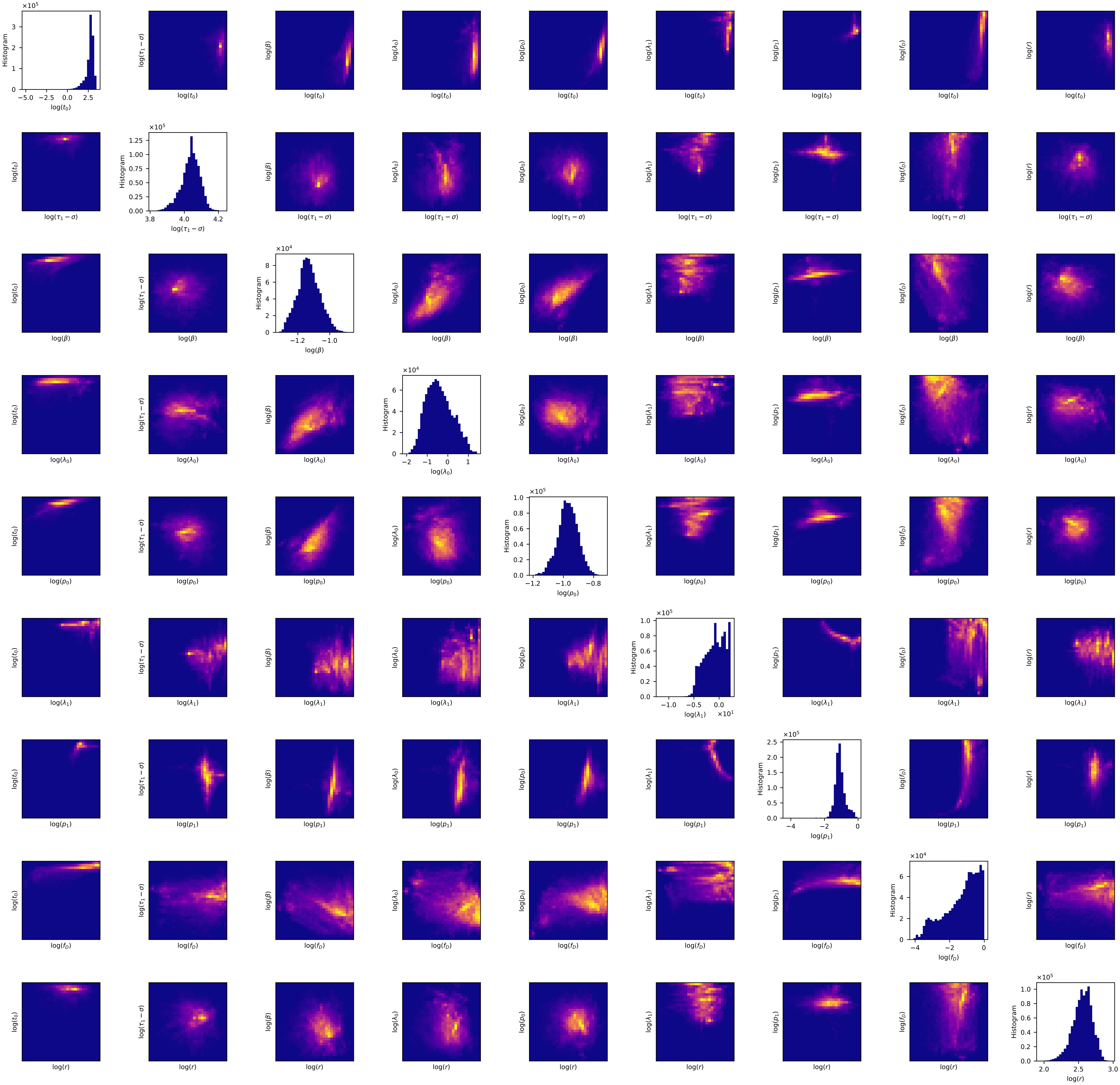

×10^5
Histogram
log(t_0)
log($\tau_1-\sigma$)
log(β)
log(λ_0)
log(p_0)
log(λ_1)
log(p_1)
log(f_D)
log(r)
−5.0 −2.5 0.0 2.5
3.8 4.0 4.2
0.00 0.25 0.50 0.75 1.00 1.25
×10^4
−1.2 −1.0
0 2 4 6 8
−2 −1 0 1
0 2 4 6
0.0 0.2 0.4 0.6 0.8 1.0
−1.2 −1.0 −0.8
−1.0 −0.5 0.0
×10^1
−4 −2 0
0.0 0.5 1.0 1.5 2.0 2.5
2.0 2.5 3.0

Vermont

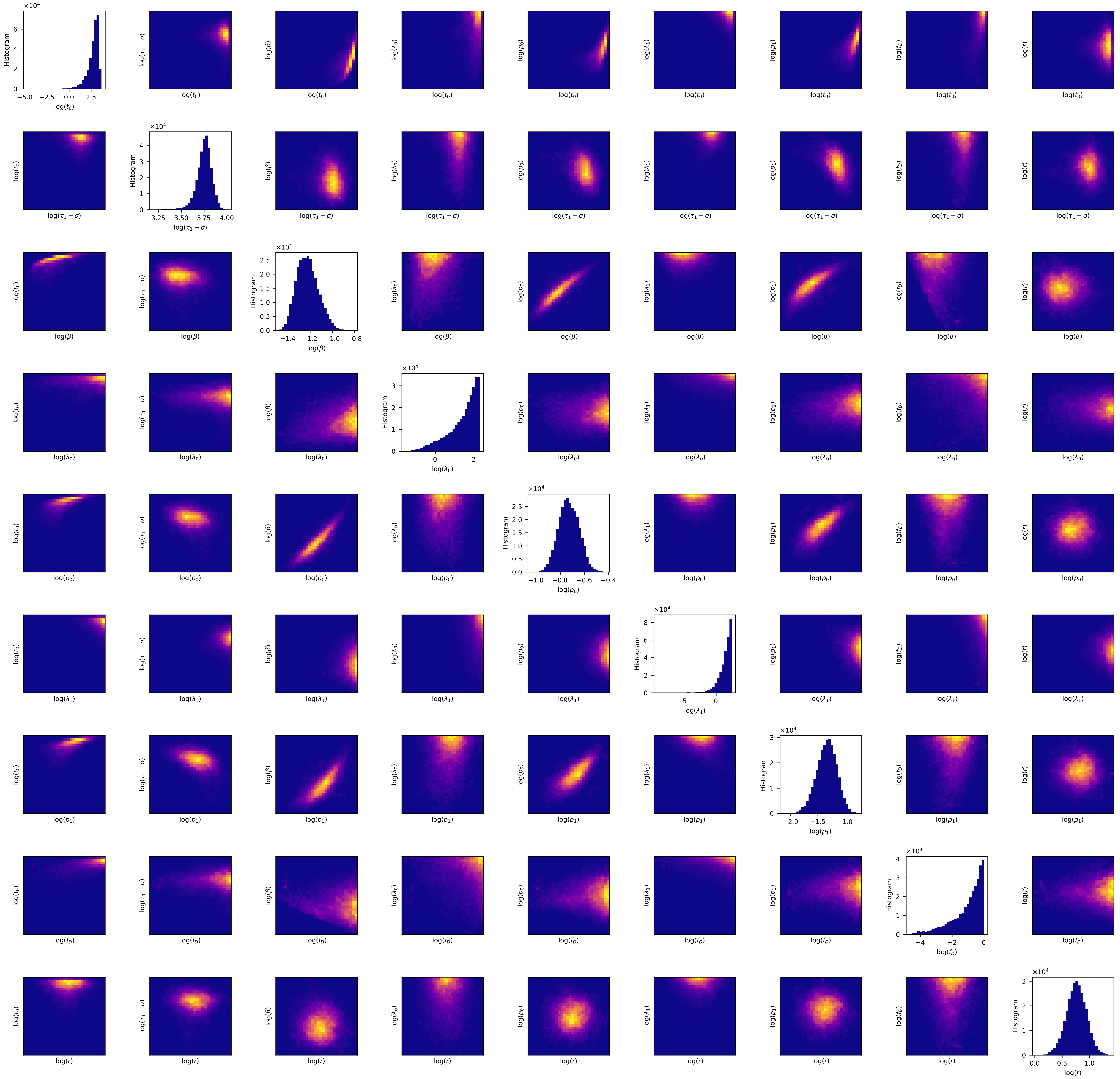

Histogram
log(t_0)
log($\tau_1 - \sigma$)
log(β)
log(λ_0)
log(p_0)
log(λ_1)
log(p_1)
log(f_D)
log(r)

Virginia

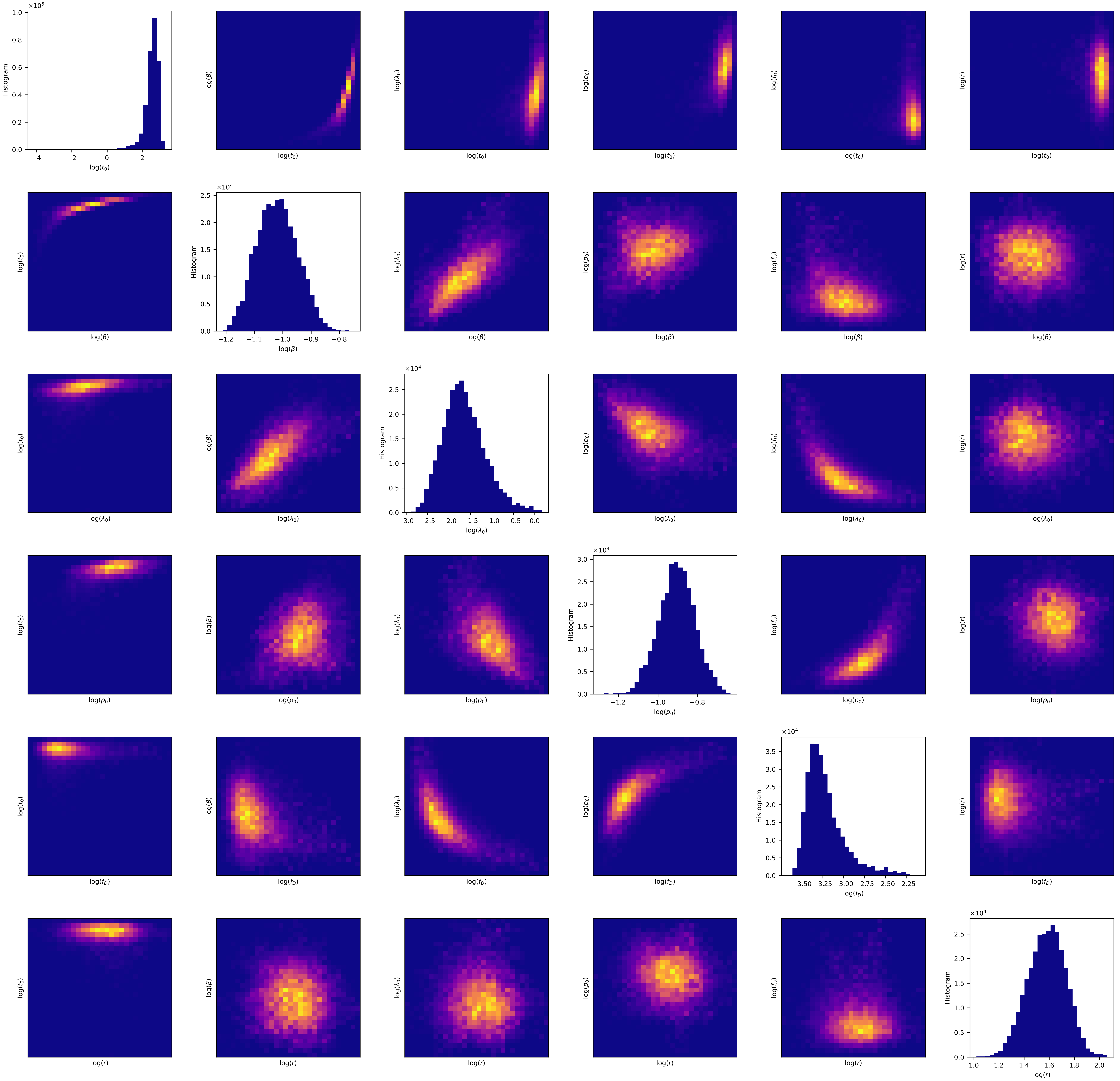

log(t_0)
log(β)
log(λ_0)
log(p_0)
log(f_D)
log(r)
Histogram
×10^5
×10^4

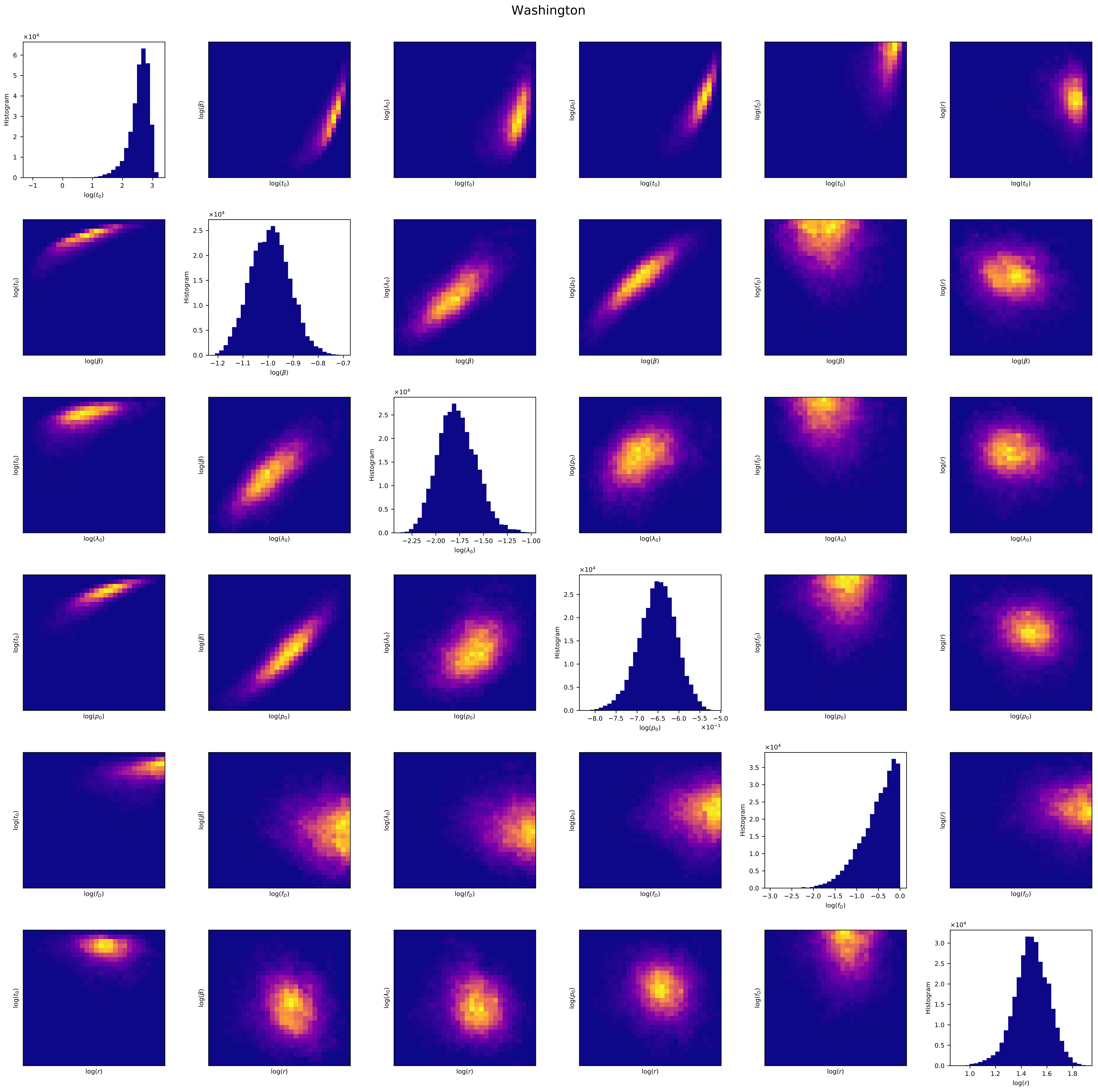
Washington
Histogram
log(t_0)
log(β)
log(λ_0)
log(p_0)
log(f_D)
log(r)
×10^4
×10^-1

West Virginia

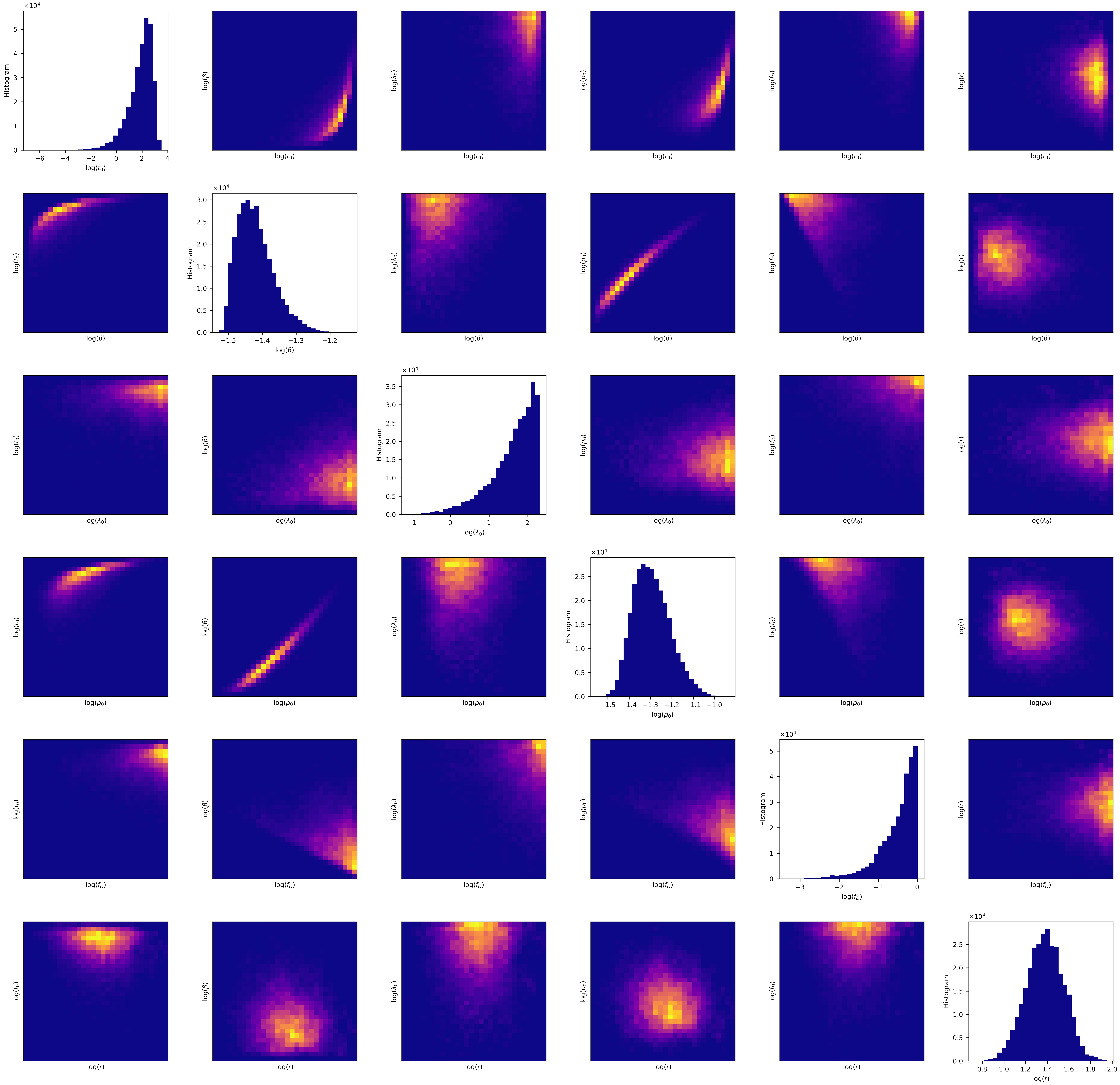

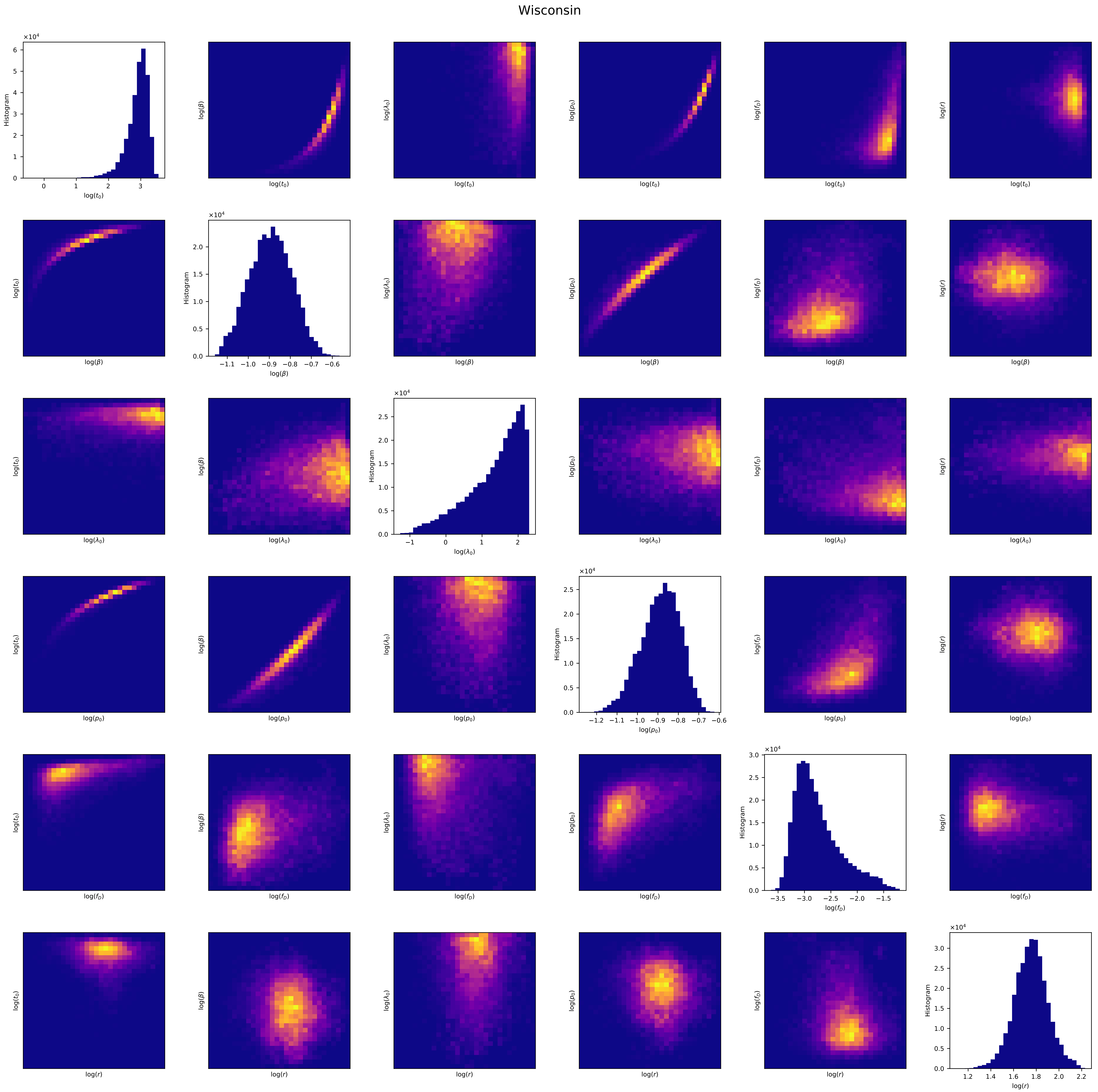
Wisconsin
Histogram
log(t_0)
log(β)
log(λ_0)
log(p_0)
log(f_D)
log(r)
×10^4

Wyoming

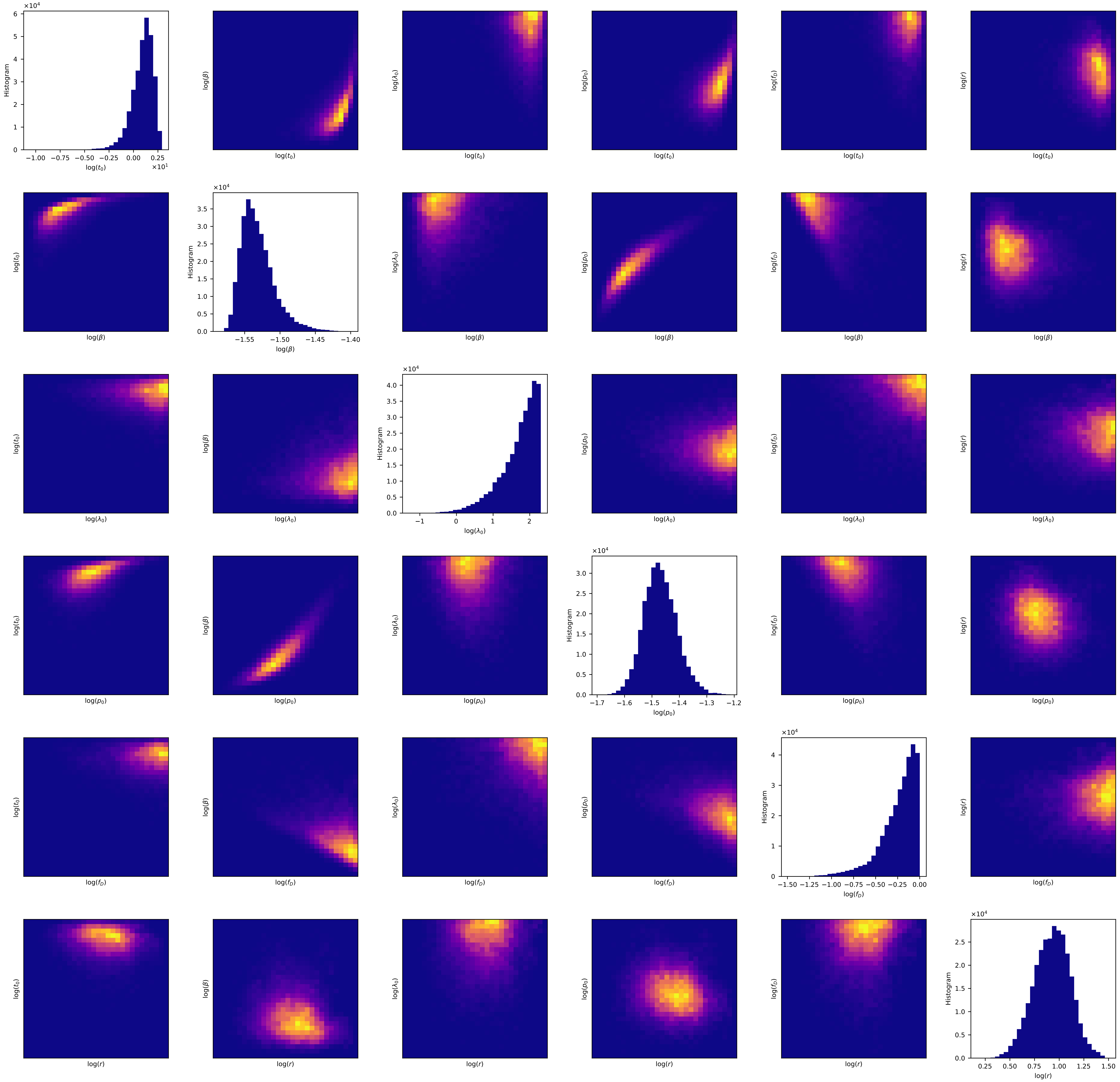

Histogram
log(t_0)
log(β)
log(λ_0)
log(p_0)
log(f_D)
log(r)
×10^4
×10^1

Figure S5. Posterior predictive checking. The time-dependent predictive posterior distribution for daily number of COVID-19 cases detected is visualized for all states except New Jersey, Wyoming, Florida, and Alaska, which are considered in Fig 1 of the main text. Inferences are based on daily reports of new COVID-19 cases from January 21 to June 21, 2020 (inclusive dates). The compartmental model [1] accounts for an initial social distancing period followed by n additional periods. We considered $n = 0, 1$ and 2 and selected the best n using the model selection procedure of Lin et al. [1]. Crosses indicate observed daily case reports. The shaded region indicates the prediction uncertainty and inferred noise in detection of new cases. The color-coded bands within the shaded region indicate the median and different credible intervals (e.g., dark purple corresponds to the median, the lightest shade of yellow corresponds to the 95% credible interval, and gradations of color between these two extremes correspond to different credible intervals as indicated in the legend). In each panel, the vertical broken line indicates the onset time of the first social-distancing period. For states with $n = 1$, there is an additional (rightmost) broken line, which indicates the onset time of the second social-distancing period. The model was used to make forecasts of new case detection for 14 days after June 21, 2020. The last prediction date was July 5, 2020.

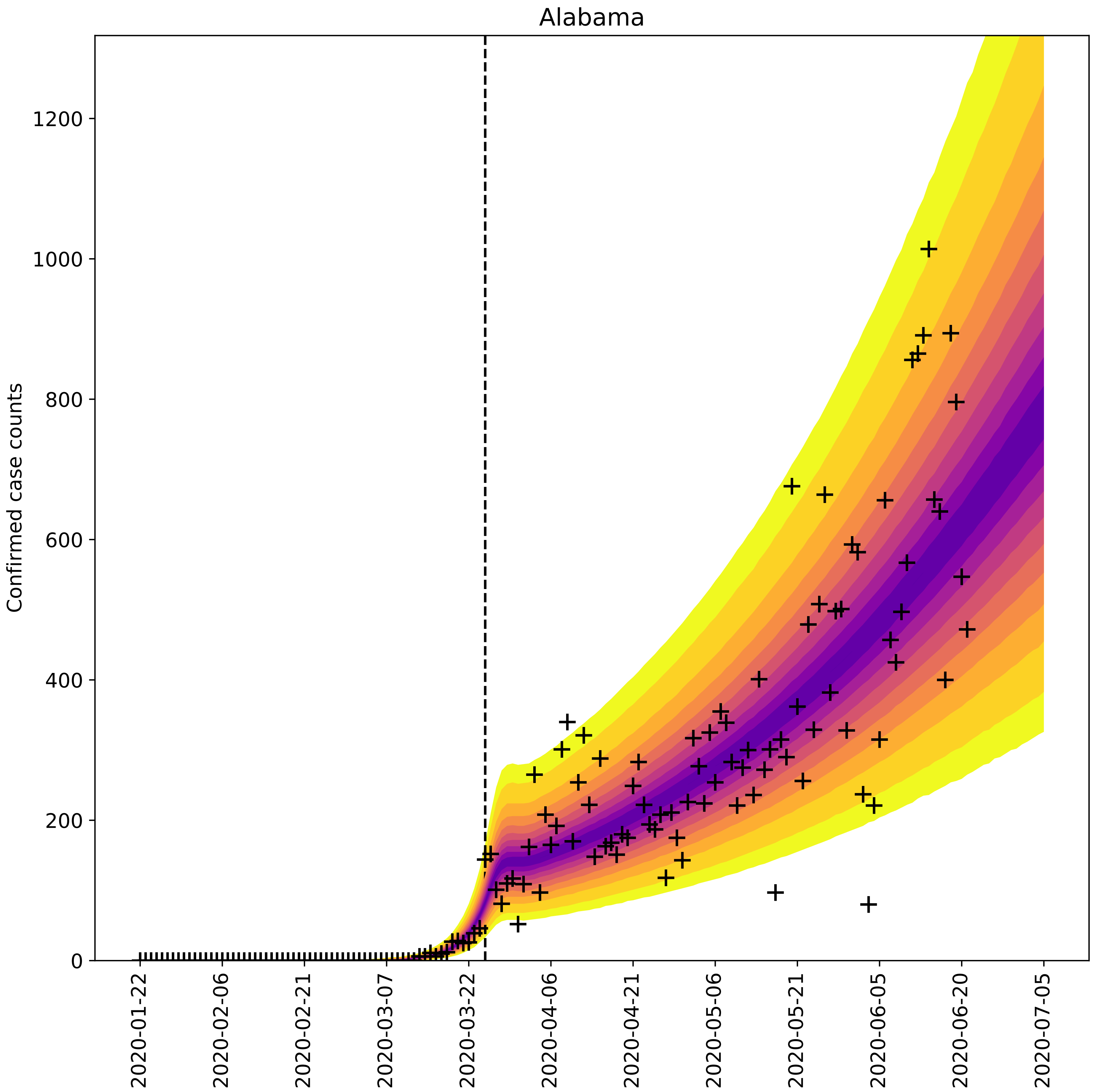

Alabama
Confirmed case counts
0
200
400
600
800
1000
1200
2020-01-22
2020-02-06
2020-02-21
2020-03-07
2020-03-22
2020-04-06
2020-04-21
2020-05-06
2020-05-21
2020-06-05
2020-06-20
2020-07-05

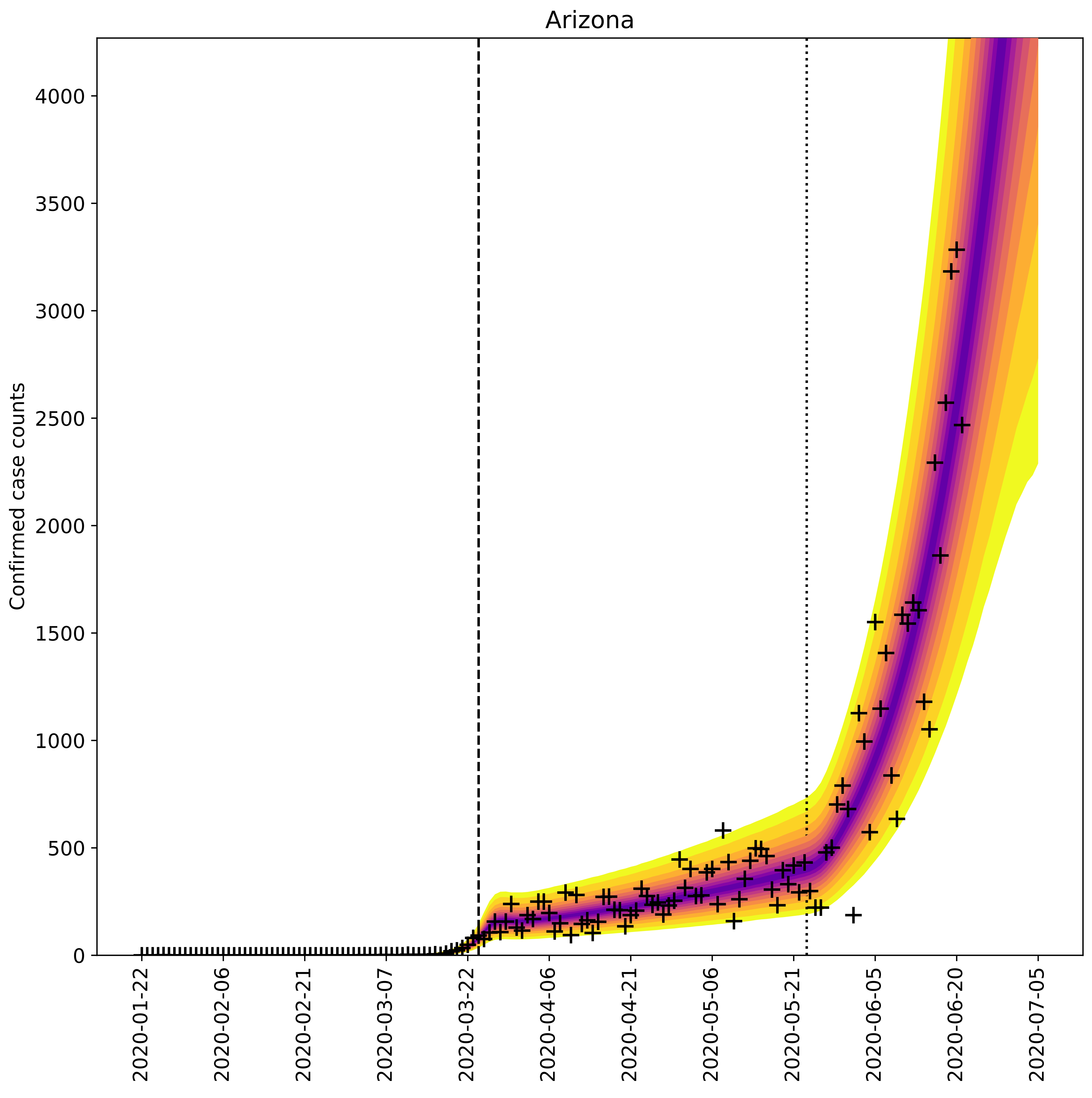

Arizona
Confirmed case counts
0
500
1000
1500
2000
2500
3000
3500
4000
2020-01-22
2020-02-06
2020-02-21
2020-03-07
2020-03-22
2020-04-06
2020-04-21
2020-05-06
2020-05-21
2020-06-05
2020-06-20
2020-07-05

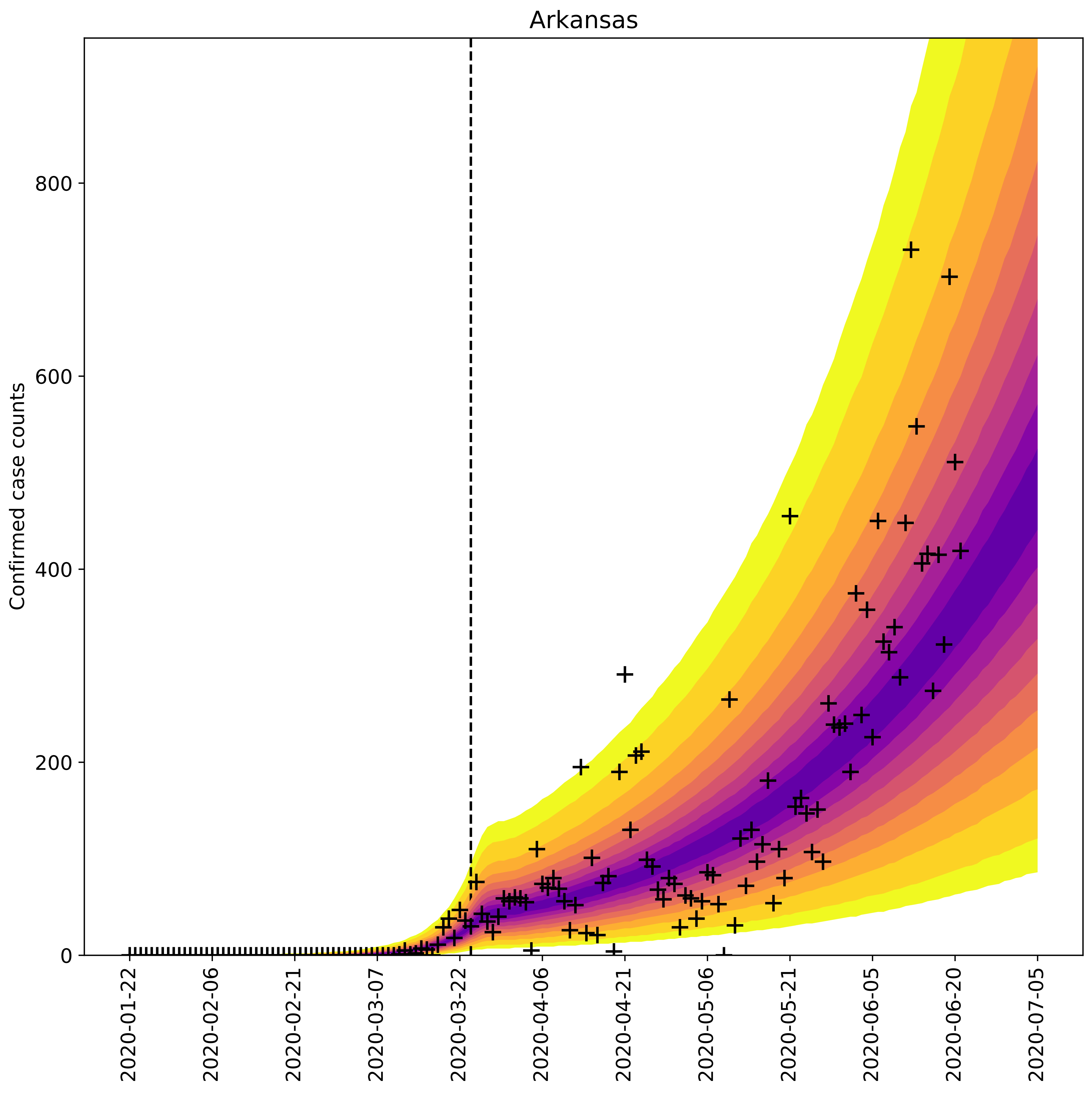

Arkansas
Confirmed case counts
0
200
400
600
800
2020-01-22
2020-02-06
2020-02-21
2020-03-07
2020-03-22
2020-04-06
2020-04-21
2020-05-06
2020-05-21
2020-06-05
2020-06-20
2020-07-05

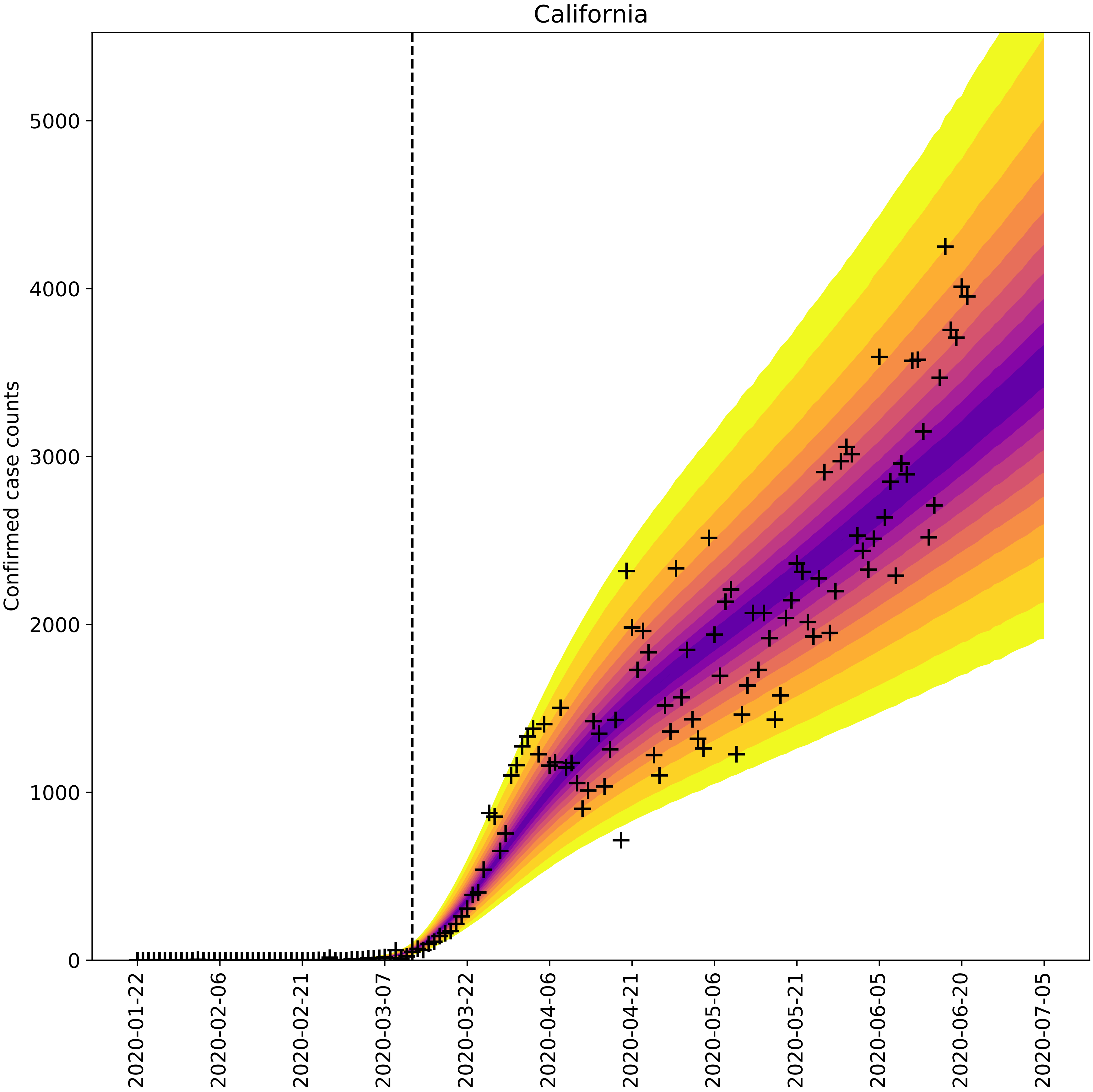
California
Confirmed case counts
5000
4000
3000
2000
1000
0
2020-01-22
2020-02-06
2020-02-21
2020-03-07
2020-03-22
2020-04-06
2020-04-21
2020-05-06
2020-05-21
2020-06-05
2020-06-20
2020-07-05

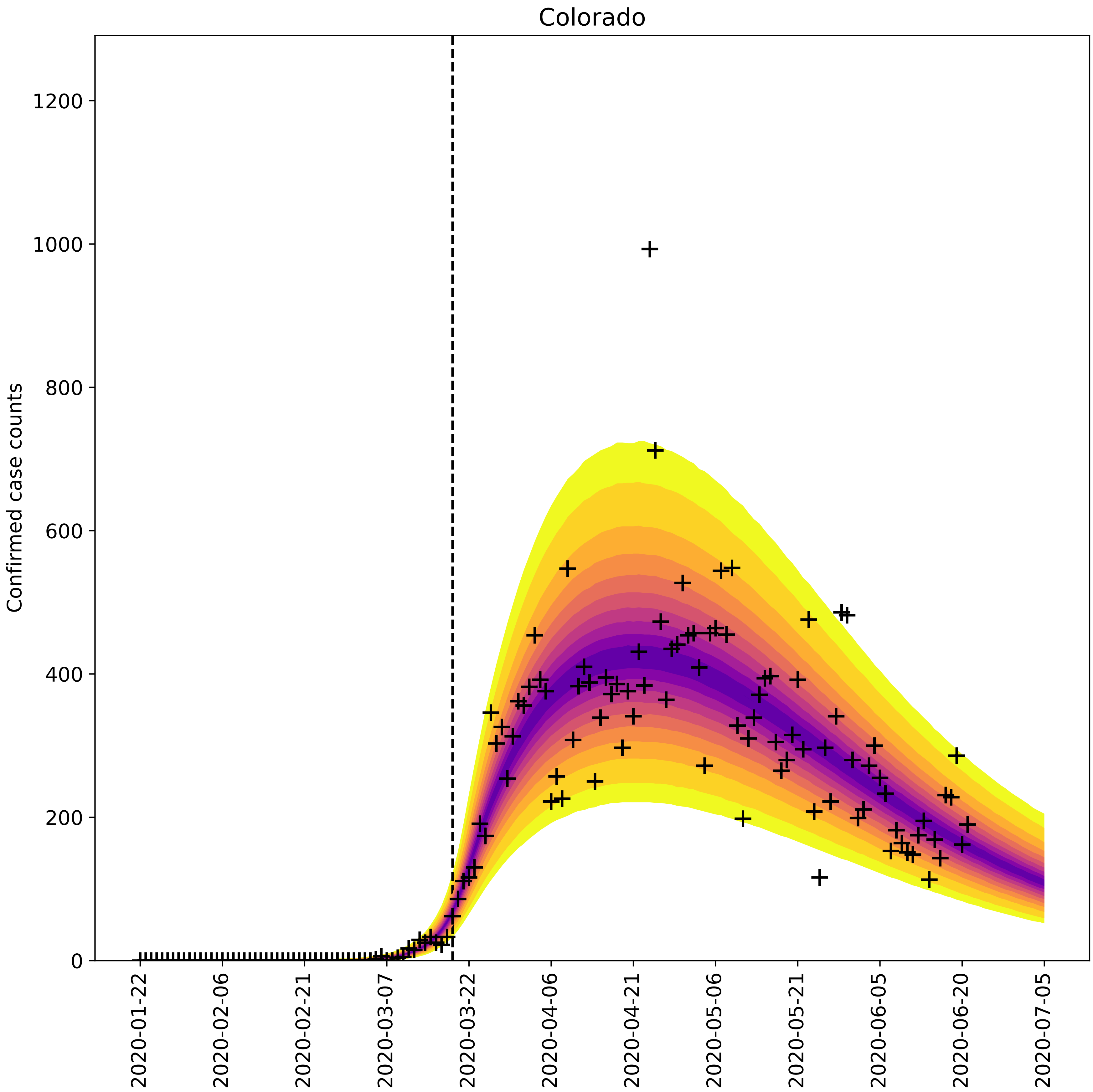
Colorado
Confirmed case counts
1200
1000
800
600
400
200
0
2020-01-22
2020-02-06
2020-02-21
2020-03-07
2020-03-22
2020-04-06
2020-04-21
2020-05-06
2020-05-21
2020-06-05
2020-06-20
2020-07-05

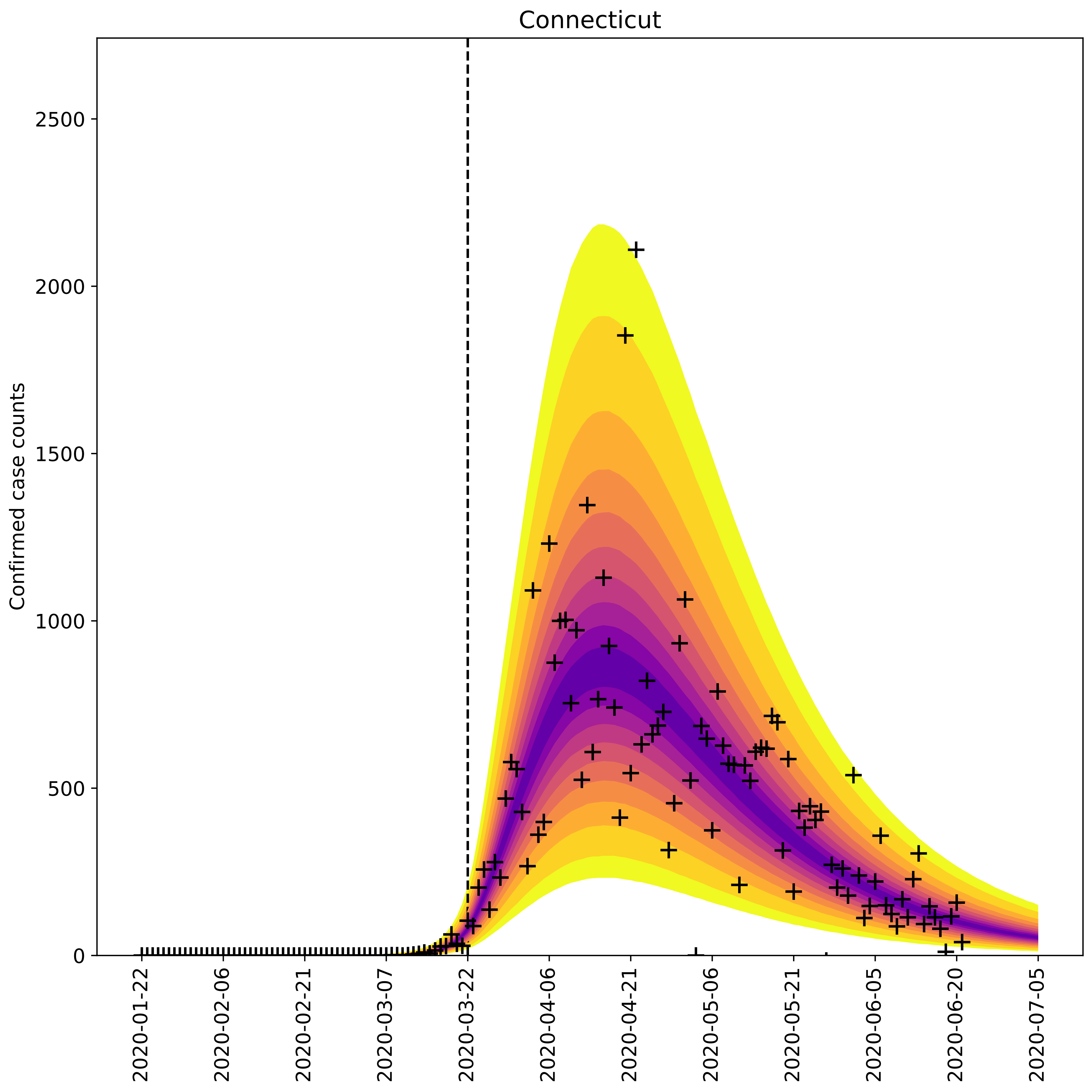
Connecticut
Confirmed case counts
0
500
1000
1500
2000
2500
2020-01-22
2020-02-06
2020-02-21
2020-03-07
2020-03-22
2020-04-06
2020-04-21
2020-05-06
2020-05-21
2020-06-05
2020-06-20
2020-07-05

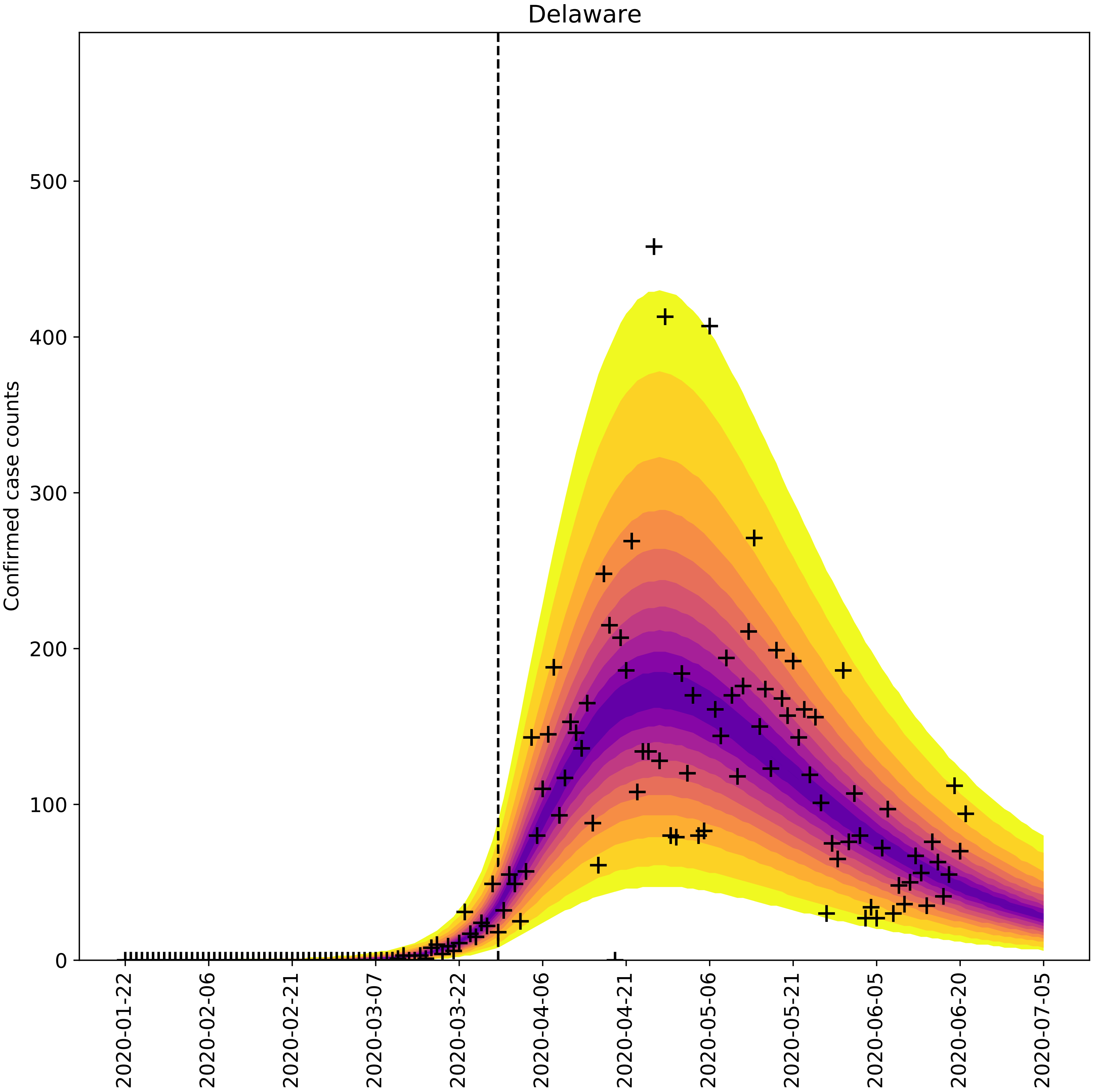
Delaware
Confirmed case counts
0
100
200
300
400
500
2020-01-22
2020-02-06
2020-02-21
2020-03-07
2020-03-22
2020-04-06
2020-04-21
2020-05-06
2020-05-21
2020-06-05
2020-06-20
2020-07-05

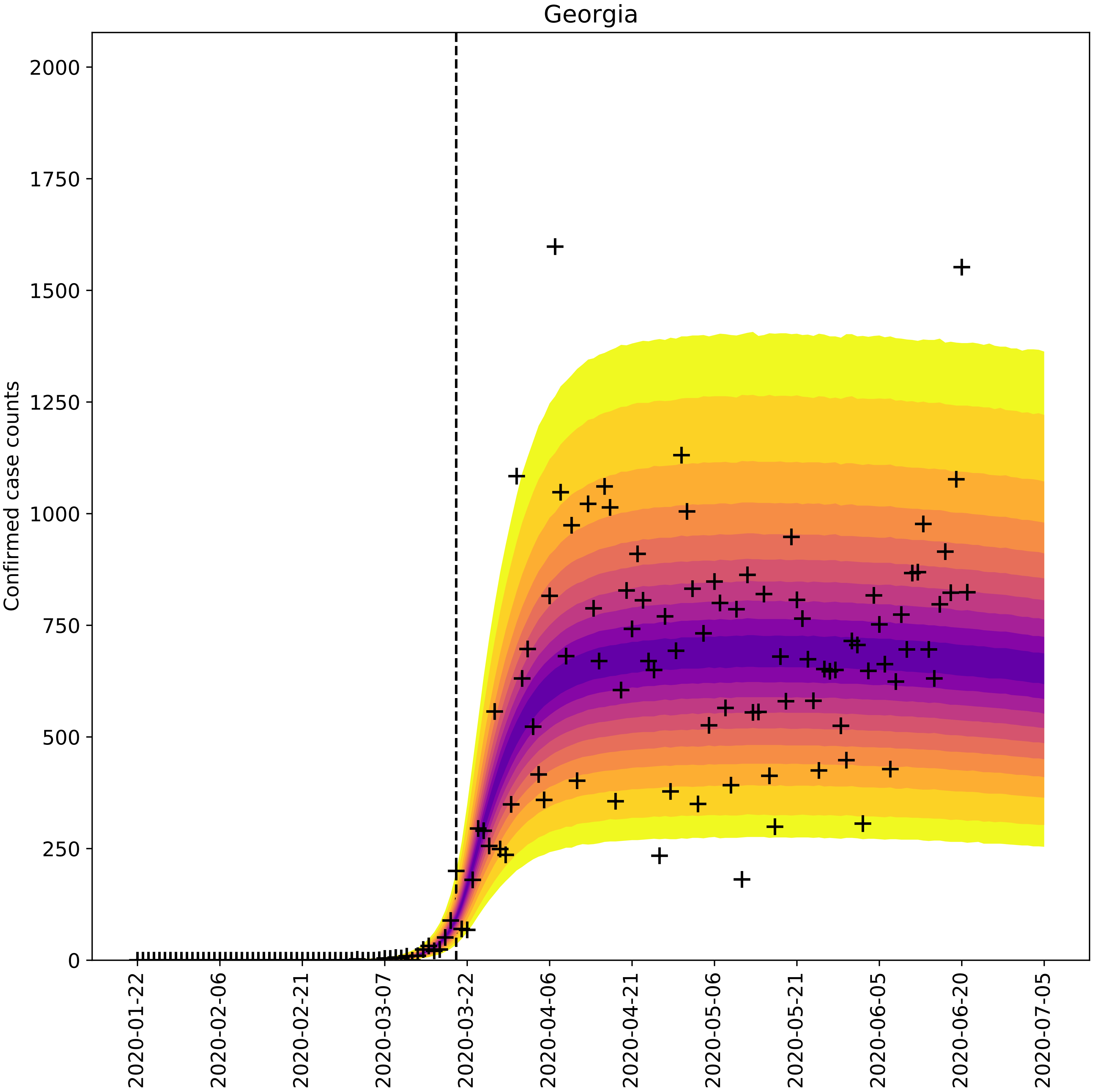
Georgia
Confirmed case counts
0
250
500
750
1000
1250
1500
1750
2000
2020-01-22
2020-02-06
2020-02-21
2020-03-07
2020-03-22
2020-04-06
2020-04-21
2020-05-06
2020-05-21
2020-06-05
2020-06-20
2020-07-05

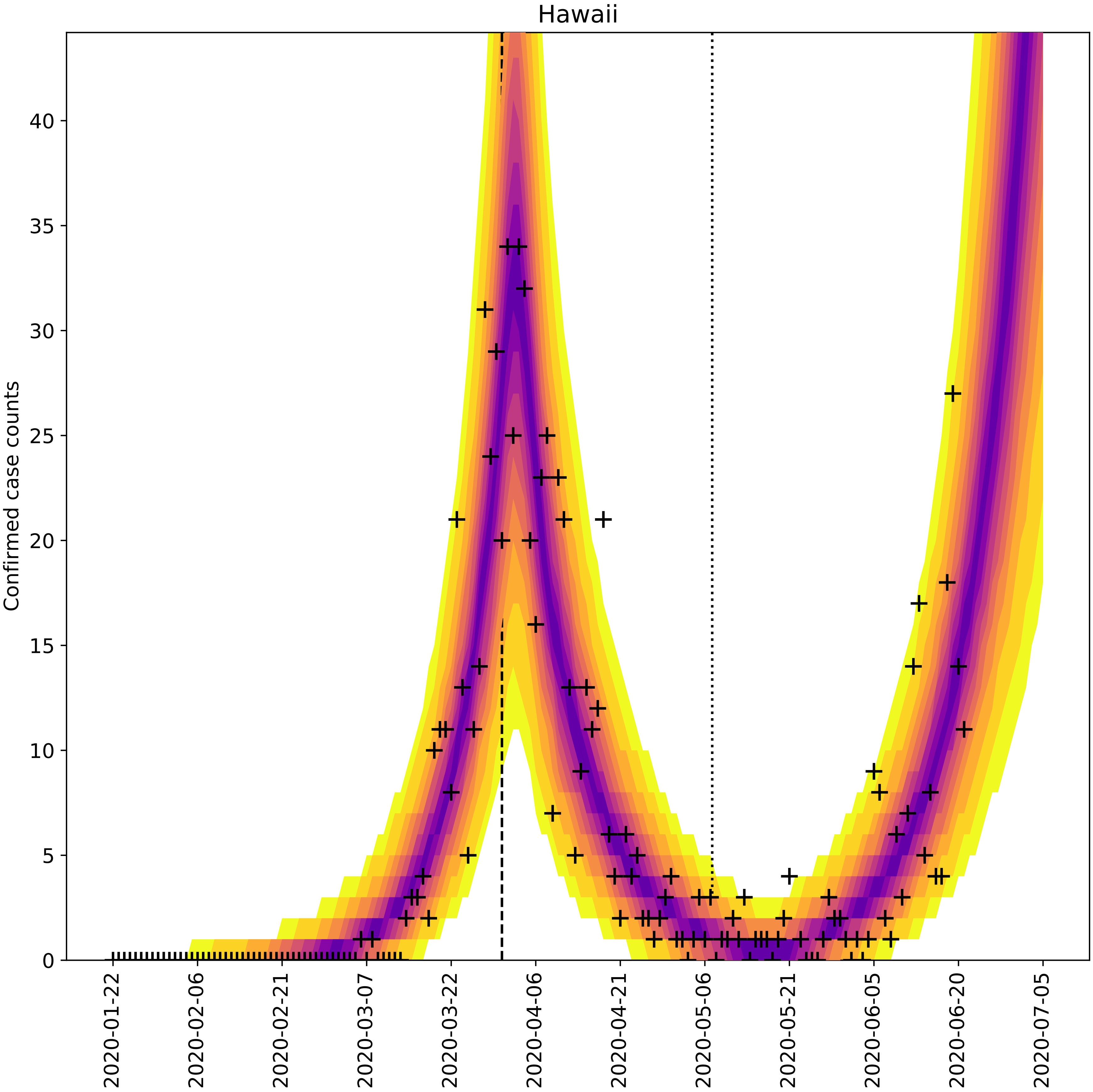
Hawaii
Confirmed case counts
0
5
10
15
20
25
30
35
40
2020-01-22
2020-02-06
2020-02-21
2020-03-07
2020-03-22
2020-04-06
2020-04-21
2020-05-06
2020-05-21
2020-06-05
2020-06-20
2020-07-05

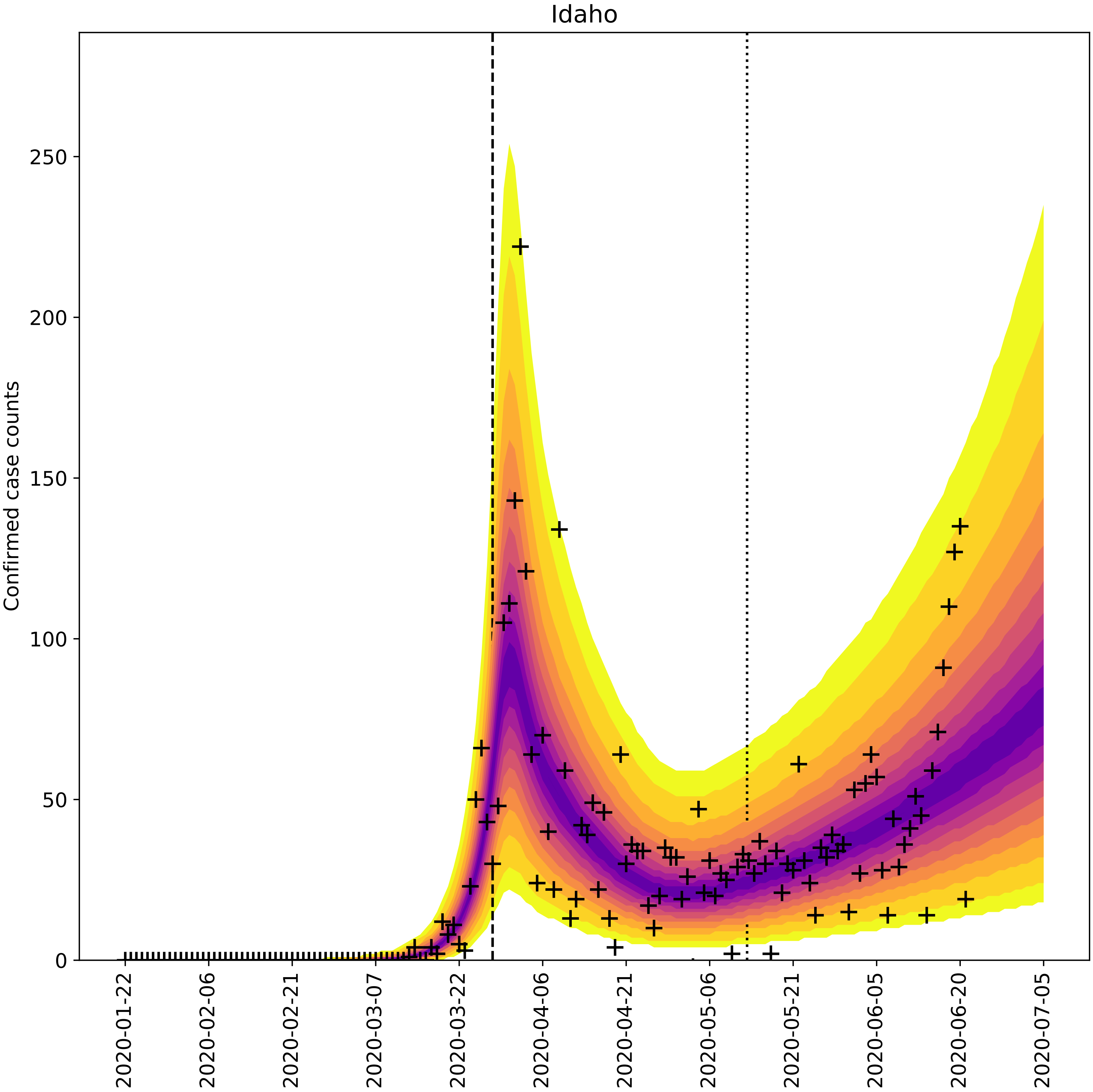

Idaho
Confirmed case counts
0
50
100
150
200
250
2020-01-22
2020-02-06
2020-02-21
2020-03-07
2020-03-22
2020-04-06
2020-04-21
2020-05-06
2020-05-21
2020-06-05
2020-06-20
2020-07-05

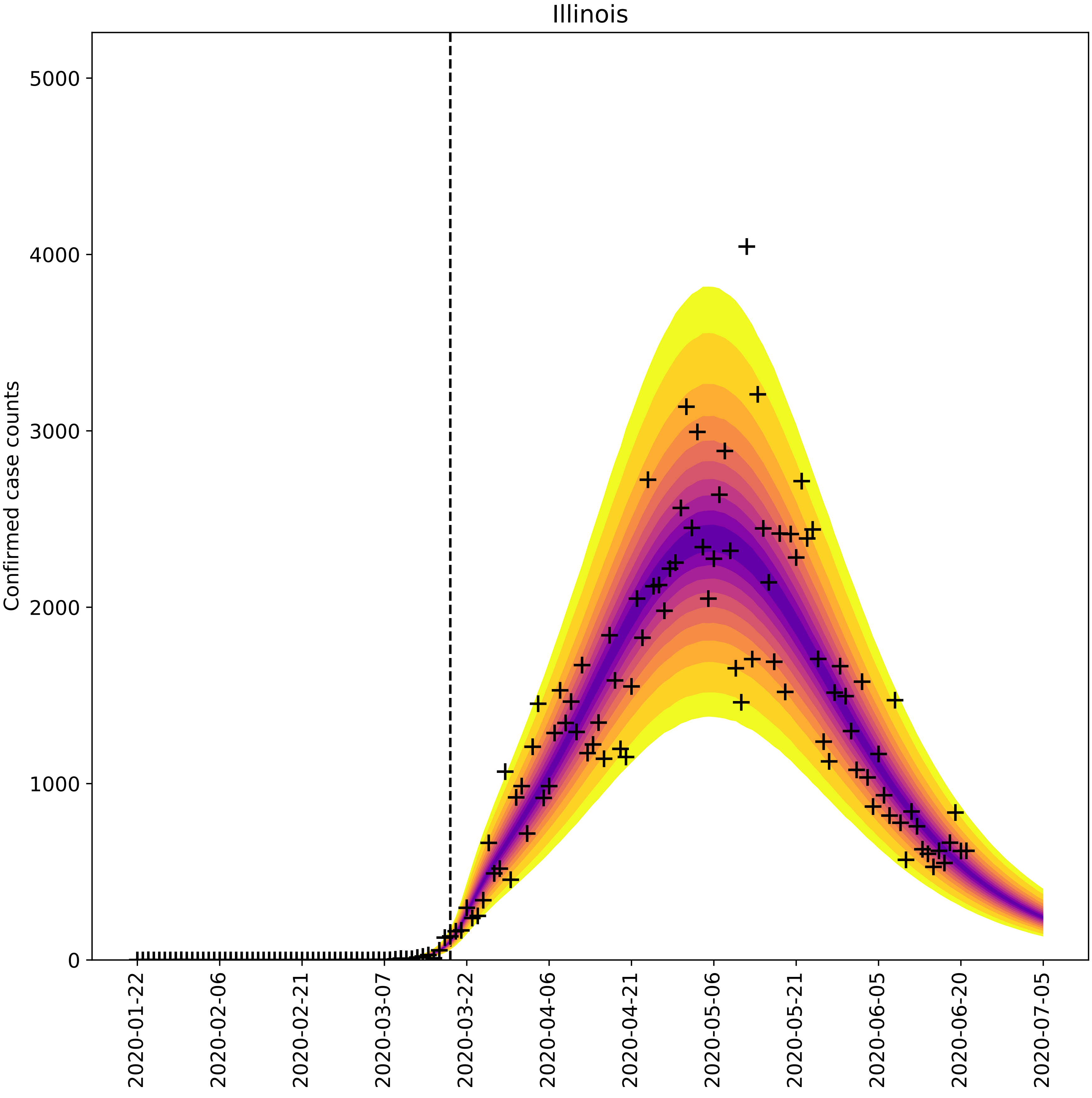

Illinois
Confirmed case counts
0
1000
2000
3000
4000
5000
2020-01-22
2020-02-06
2020-02-21
2020-03-07
2020-03-22
2020-04-06
2020-04-21
2020-05-06
2020-05-21
2020-06-05
2020-06-20
2020-07-05

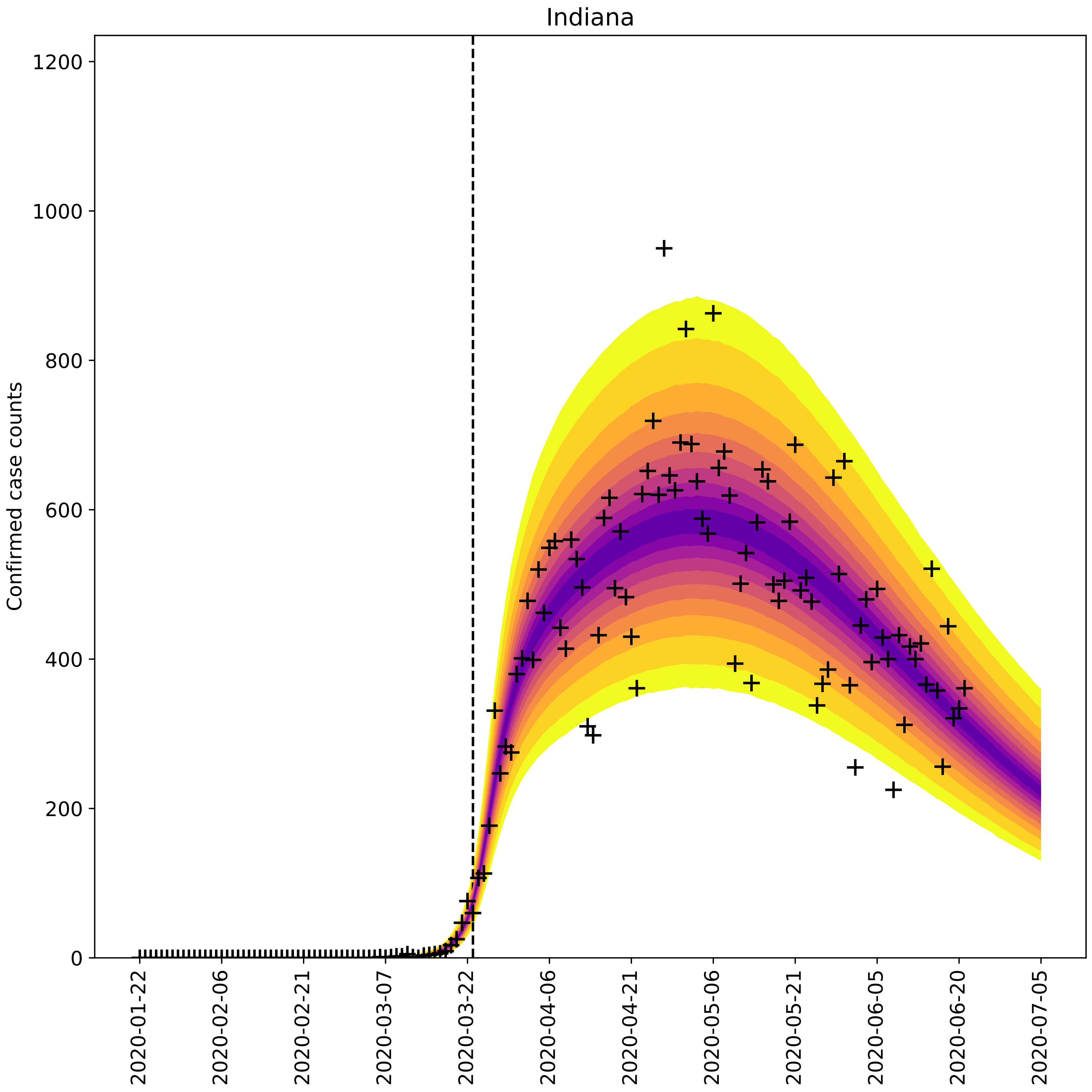

Indiana
Confirmed case counts
0
200
400
600
800
1000
1200
2020-01-22
2020-02-06
2020-02-21
2020-03-07
2020-03-22
2020-04-06
2020-04-21
2020-05-06
2020-05-21
2020-06-05
2020-06-20
2020-07-05

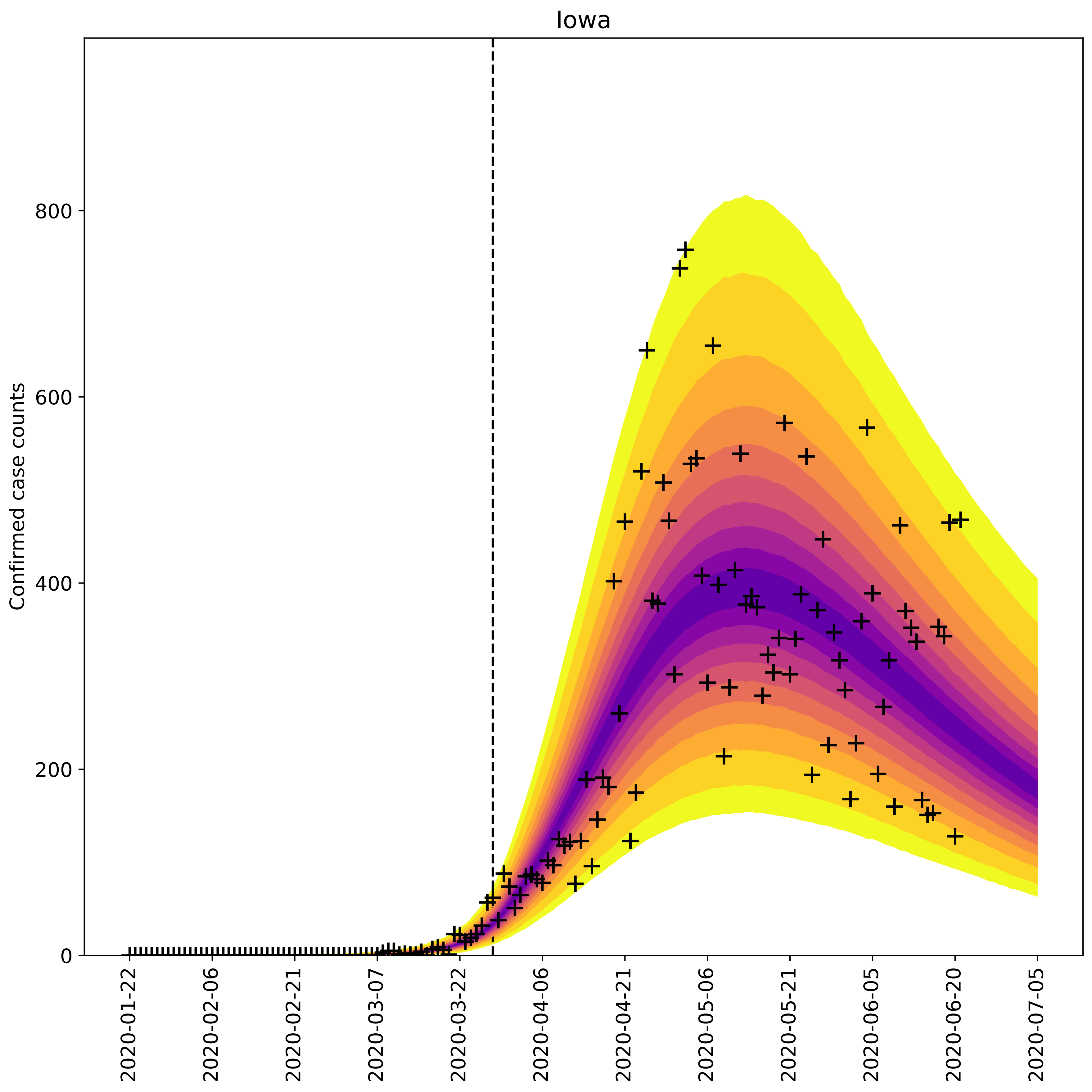

Iowa
Confirmed case counts
0
200
400
600
800
2020-01-22
2020-02-06
2020-02-21
2020-03-07
2020-03-22
2020-04-06
2020-04-21
2020-05-06
2020-05-21
2020-06-05
2020-06-20
2020-07-05

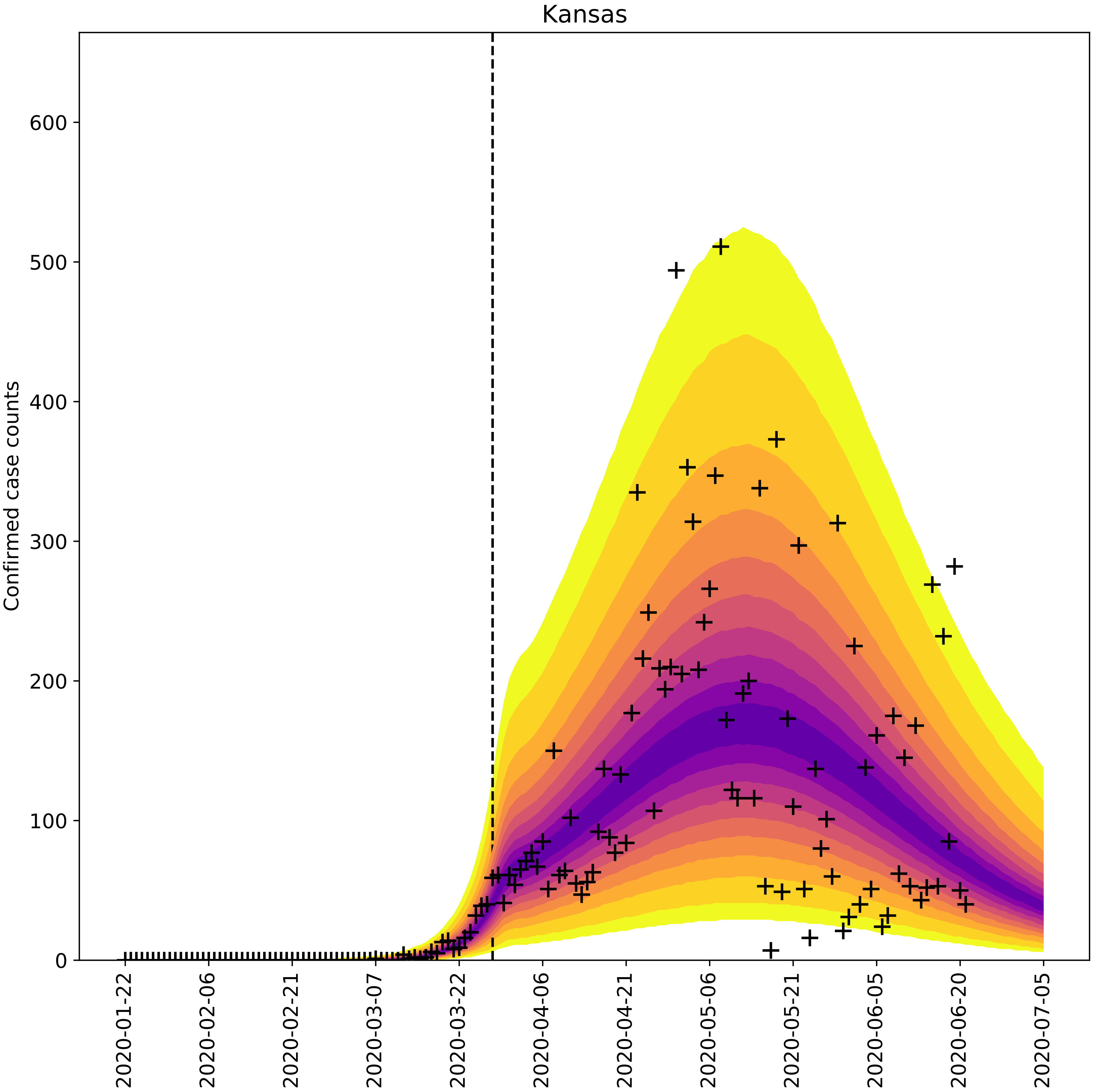
Kansas
Confirmed case counts
600
500
400
300
200
100
0
2020-01-22
2020-02-06
2020-02-21
2020-03-07
2020-03-22
2020-04-06
2020-04-21
2020-05-06
2020-05-21
2020-06-05
2020-06-20
2020-07-05

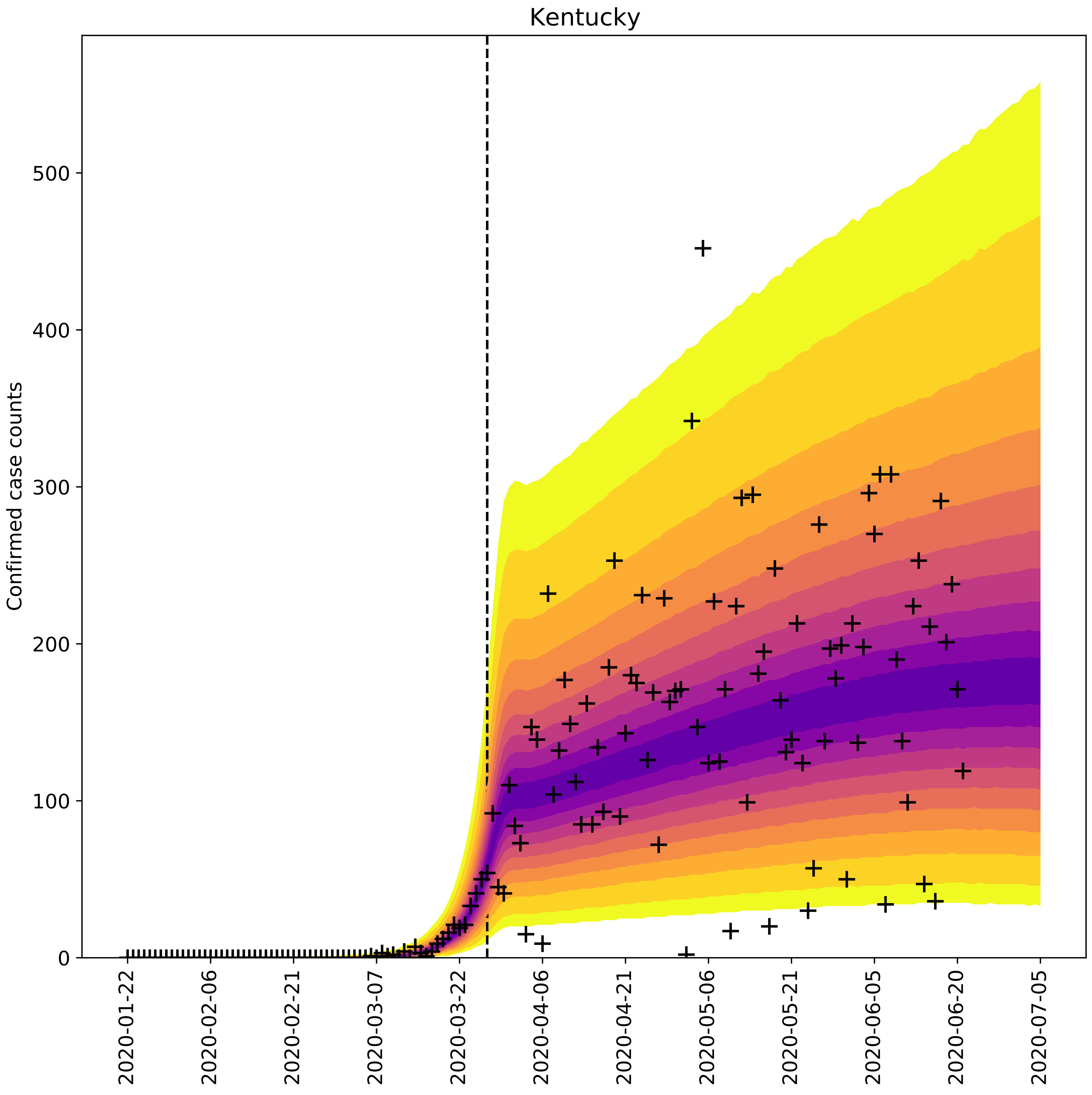

Kentucky
Confirmed case counts
0
100
200
300
400
500
2020-01-22
2020-02-06
2020-02-21
2020-03-07
2020-03-22
2020-04-06
2020-04-21
2020-05-06
2020-05-21
2020-06-05
2020-06-20
2020-07-05

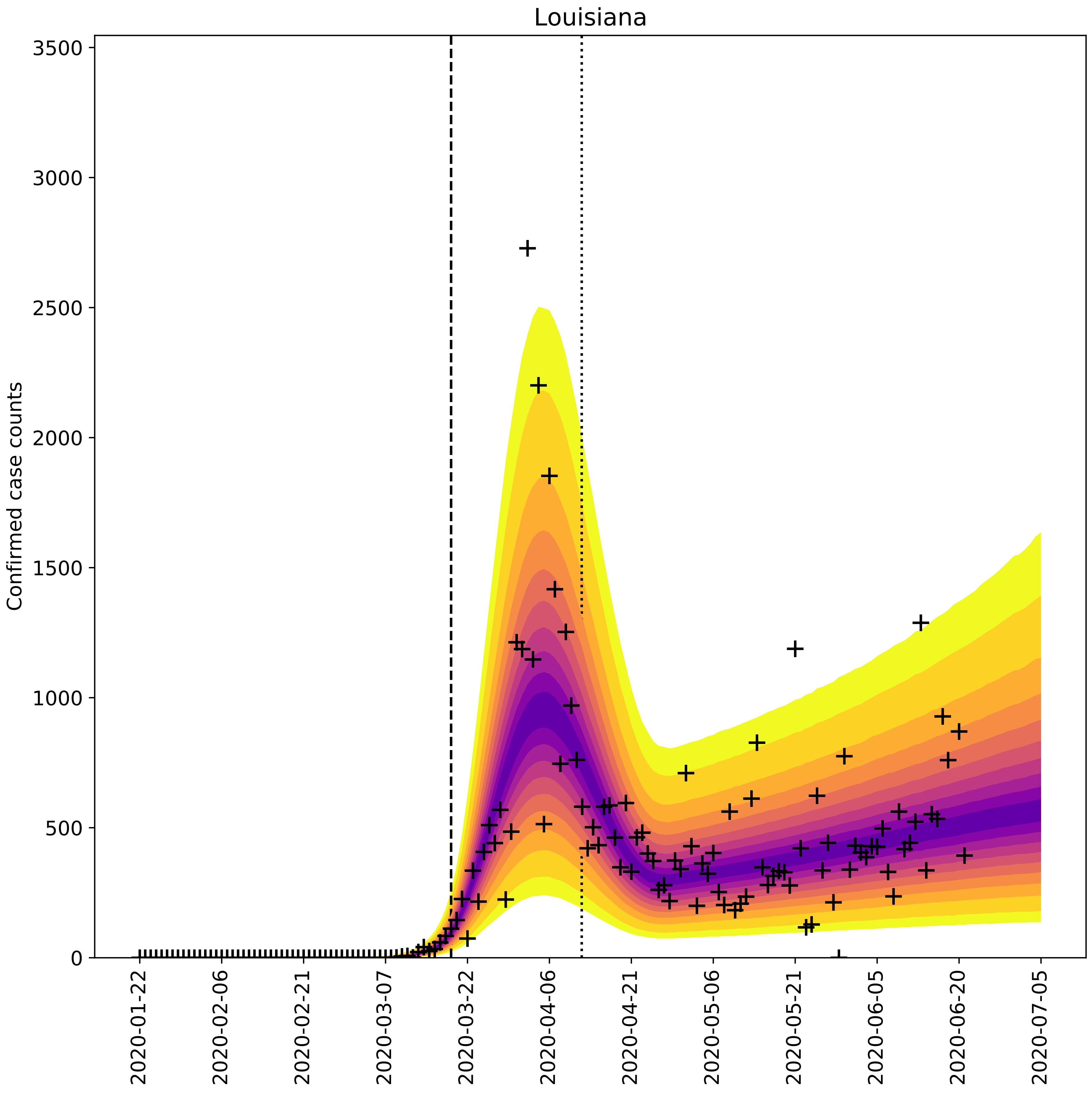
Louisiana
Confirmed case counts
0
500
1000
1500
2000
2500
3000
3500
2020-01-22
2020-02-06
2020-02-21
2020-03-07
2020-03-22
2020-04-06
2020-04-21
2020-05-06
2020-05-21
2020-06-05
2020-06-20
2020-07-05

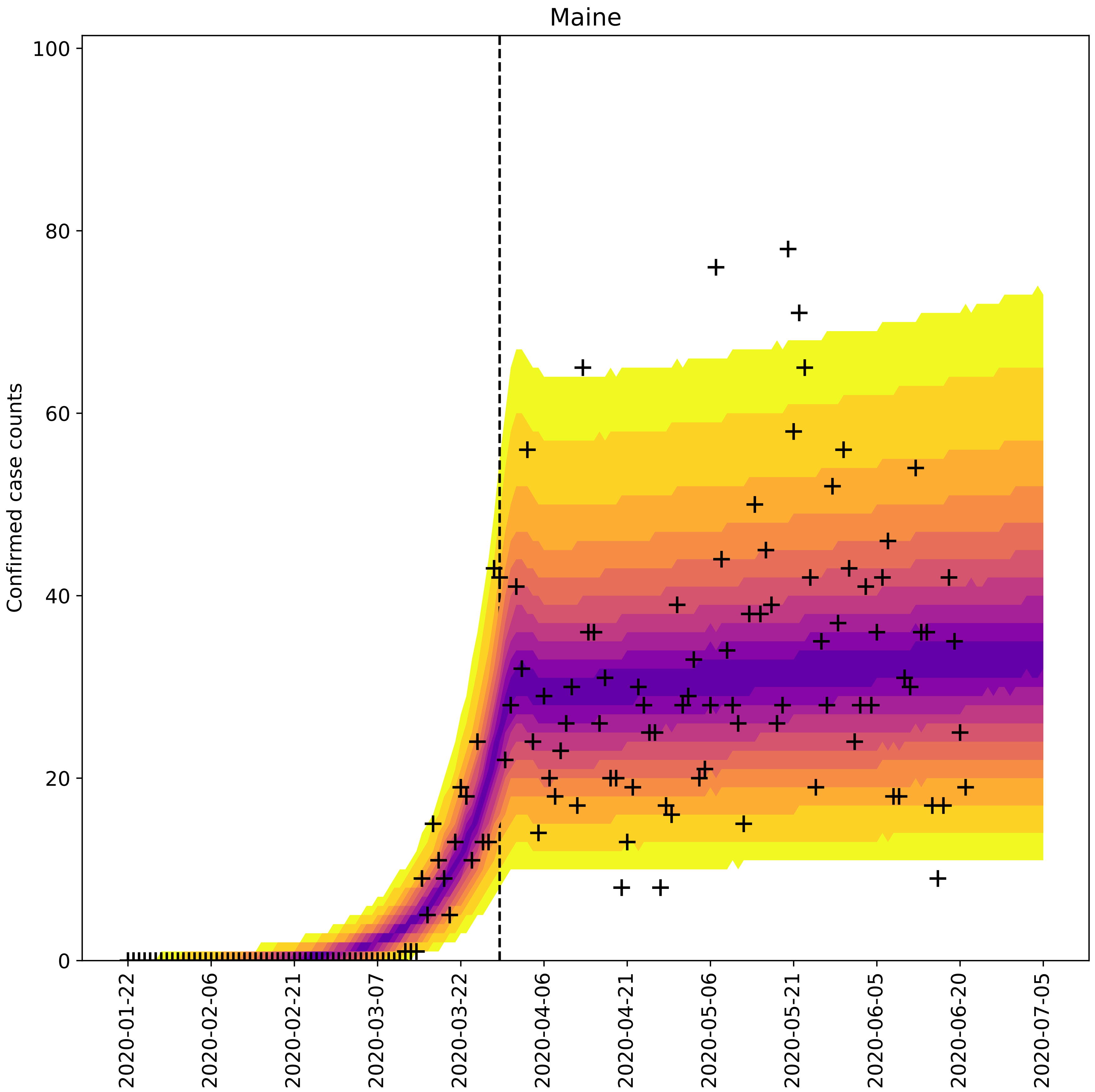

Maine
Confirmed case counts
0
20
40
60
80
100
2020-01-22
2020-02-06
2020-02-21
2020-03-07
2020-03-22
2020-04-06
2020-04-21
2020-05-06
2020-05-21
2020-06-05
2020-06-20
2020-07-05

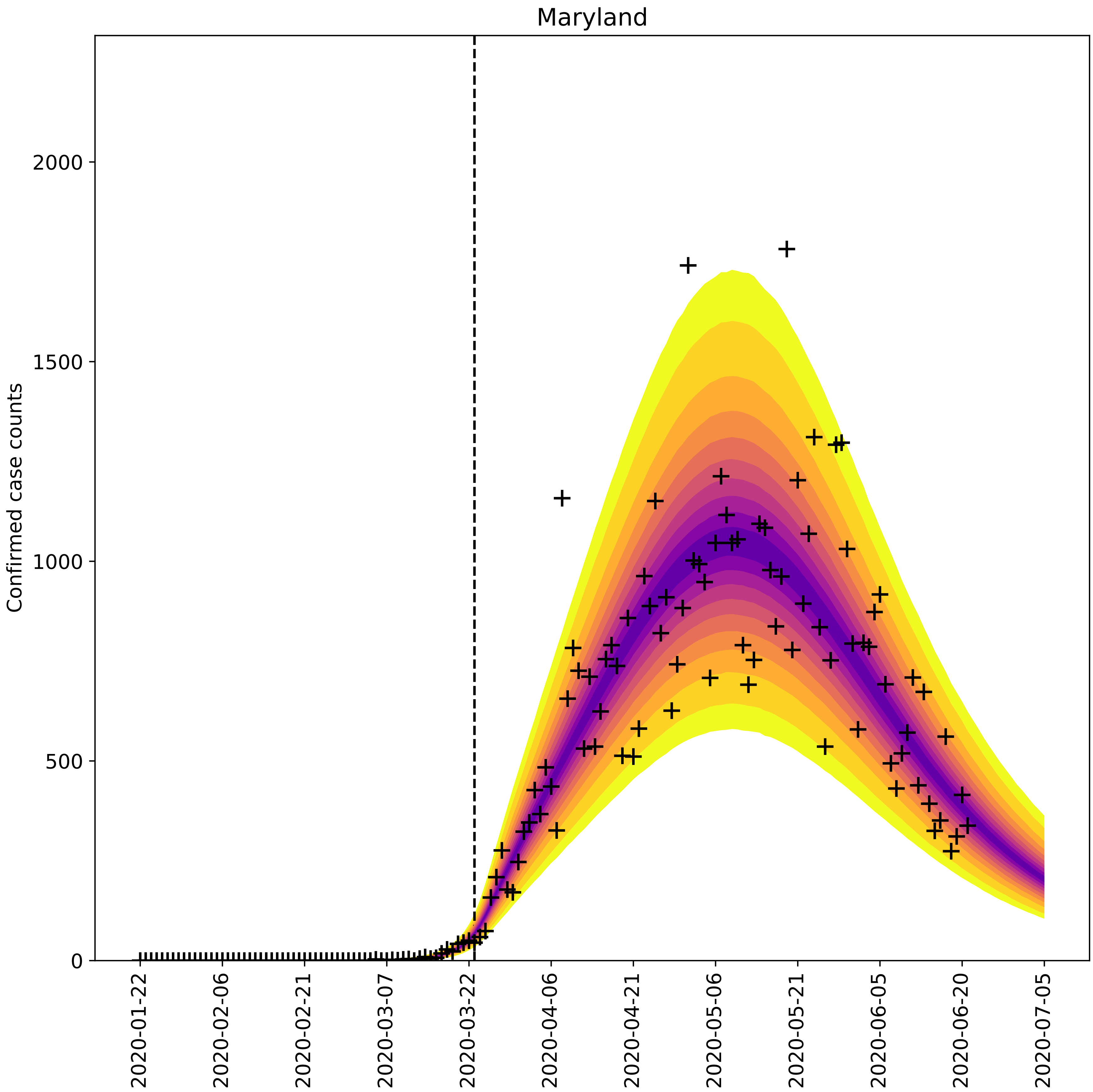

Maryland
Confirmed case counts
0
500
1000
1500
2000
2020-01-22
2020-02-06
2020-02-21
2020-03-07
2020-03-22
2020-04-06
2020-04-21
2020-05-06
2020-05-21
2020-06-05
2020-06-20
2020-07-05

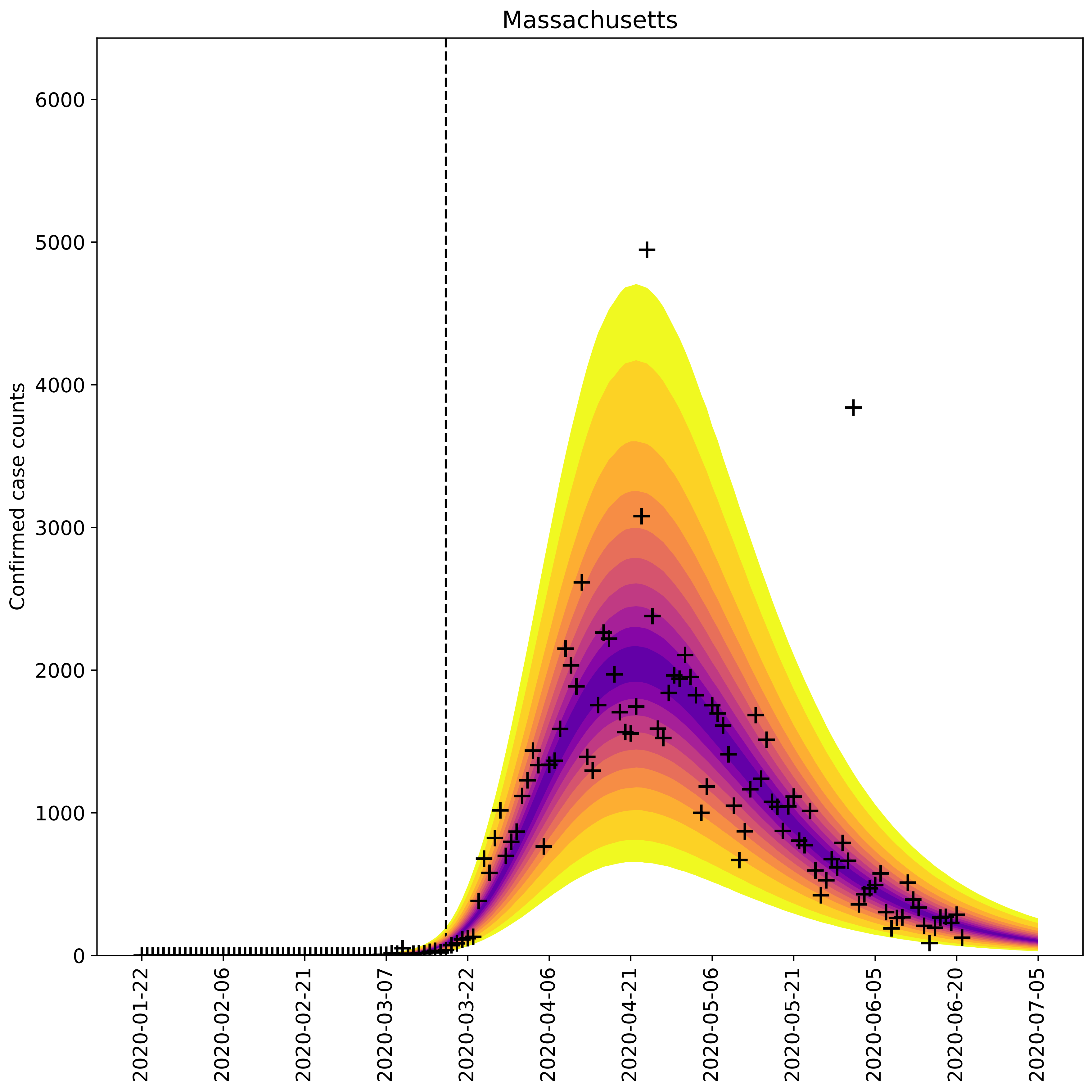
Massachusetts
Confirmed case counts
0
1000
2000
3000
4000
5000
6000
2020-01-22
2020-02-06
2020-02-21
2020-03-07
2020-03-22
2020-04-06
2020-04-21
2020-05-06
2020-05-21
2020-06-05
2020-06-20
2020-07-05

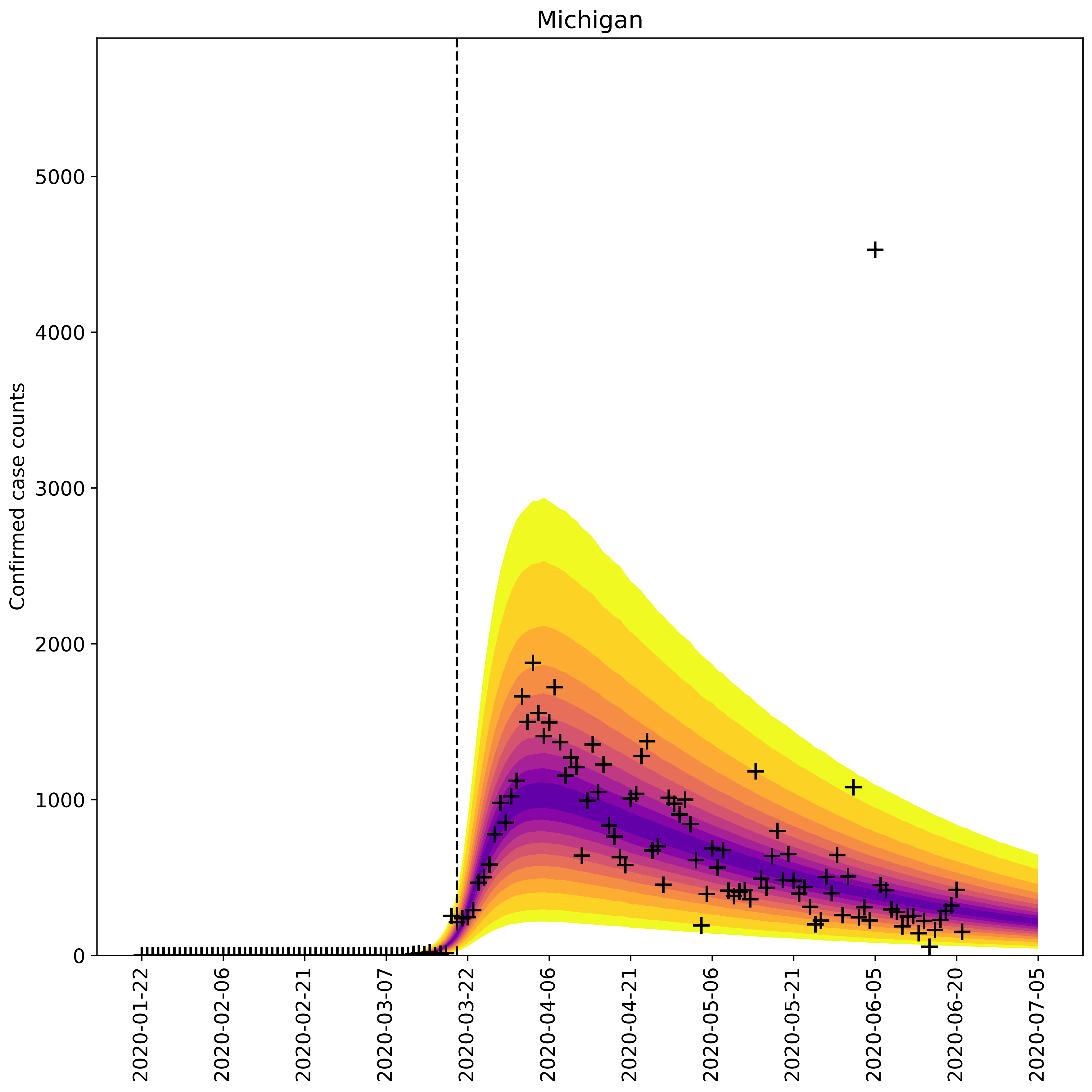

Michigan
Confirmed case counts
0
1000
2000
3000
4000
5000
2020-01-22
2020-02-06
2020-02-21
2020-03-07
2020-03-22
2020-04-06
2020-04-21
2020-05-06
2020-05-21
2020-06-05
2020-06-20
2020-07-05

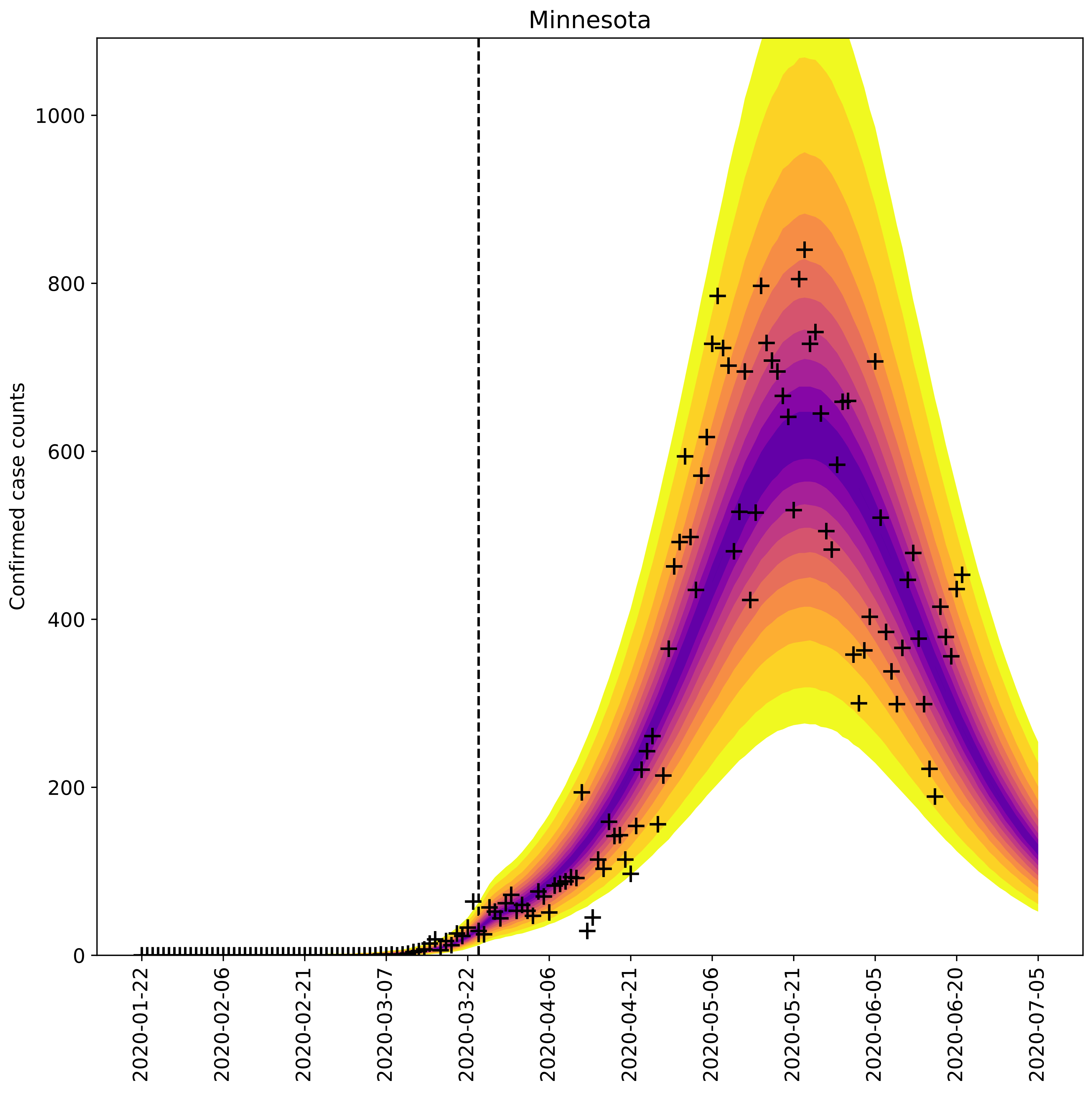
Minnesota
Confirmed case counts
0
200
400
600
800
1000
2020-01-22
2020-02-06
2020-02-21
2020-03-07
2020-03-22
2020-04-06
2020-04-21
2020-05-06
2020-05-21
2020-06-05
2020-06-20
2020-07-05

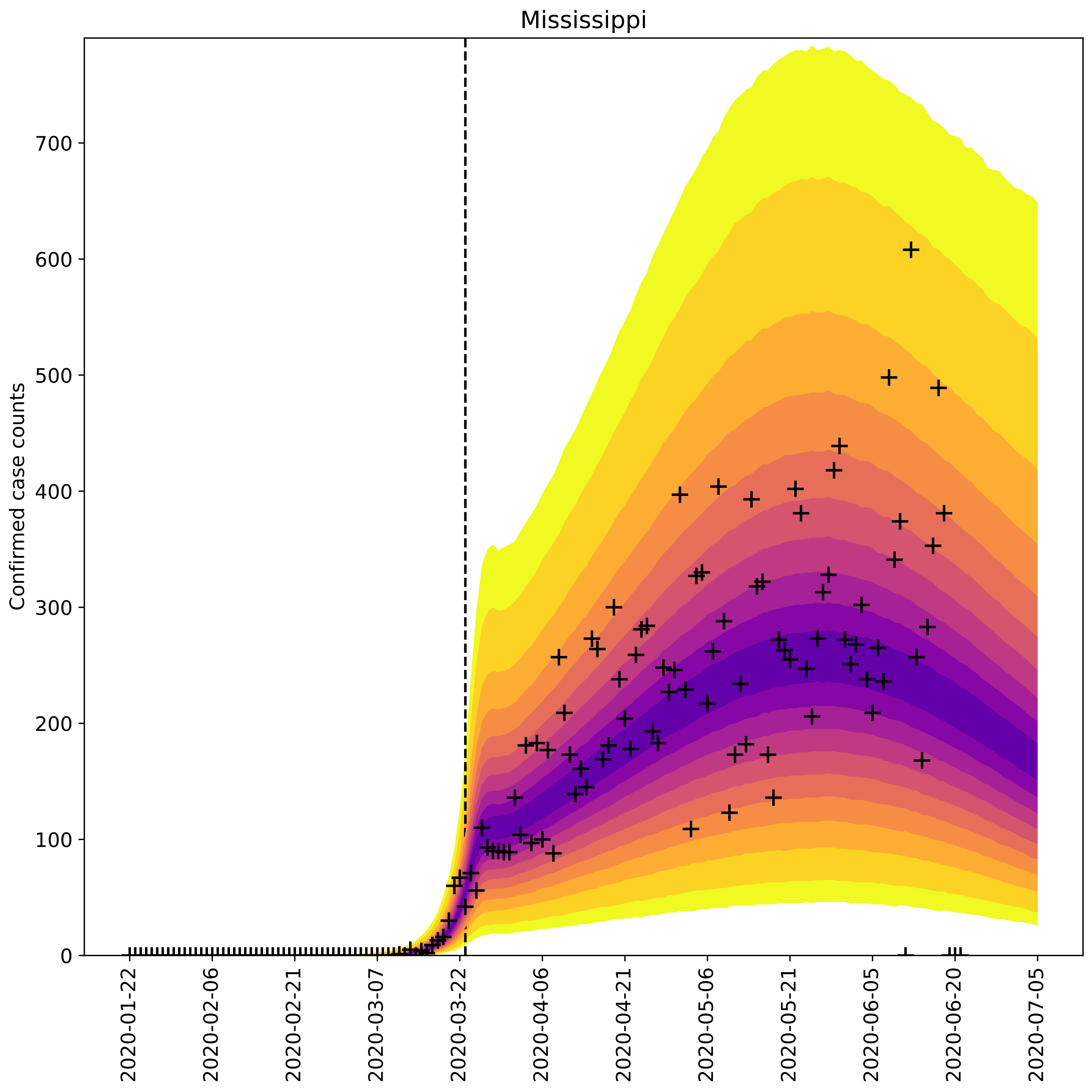
Mississippi
Confirmed case counts
0
100
200
300
400
500
600
700
2020-01-22
2020-02-06
2020-02-21
2020-03-07
2020-03-22
2020-04-06
2020-04-21
2020-05-06
2020-05-21
2020-06-05
2020-06-20
2020-07-05

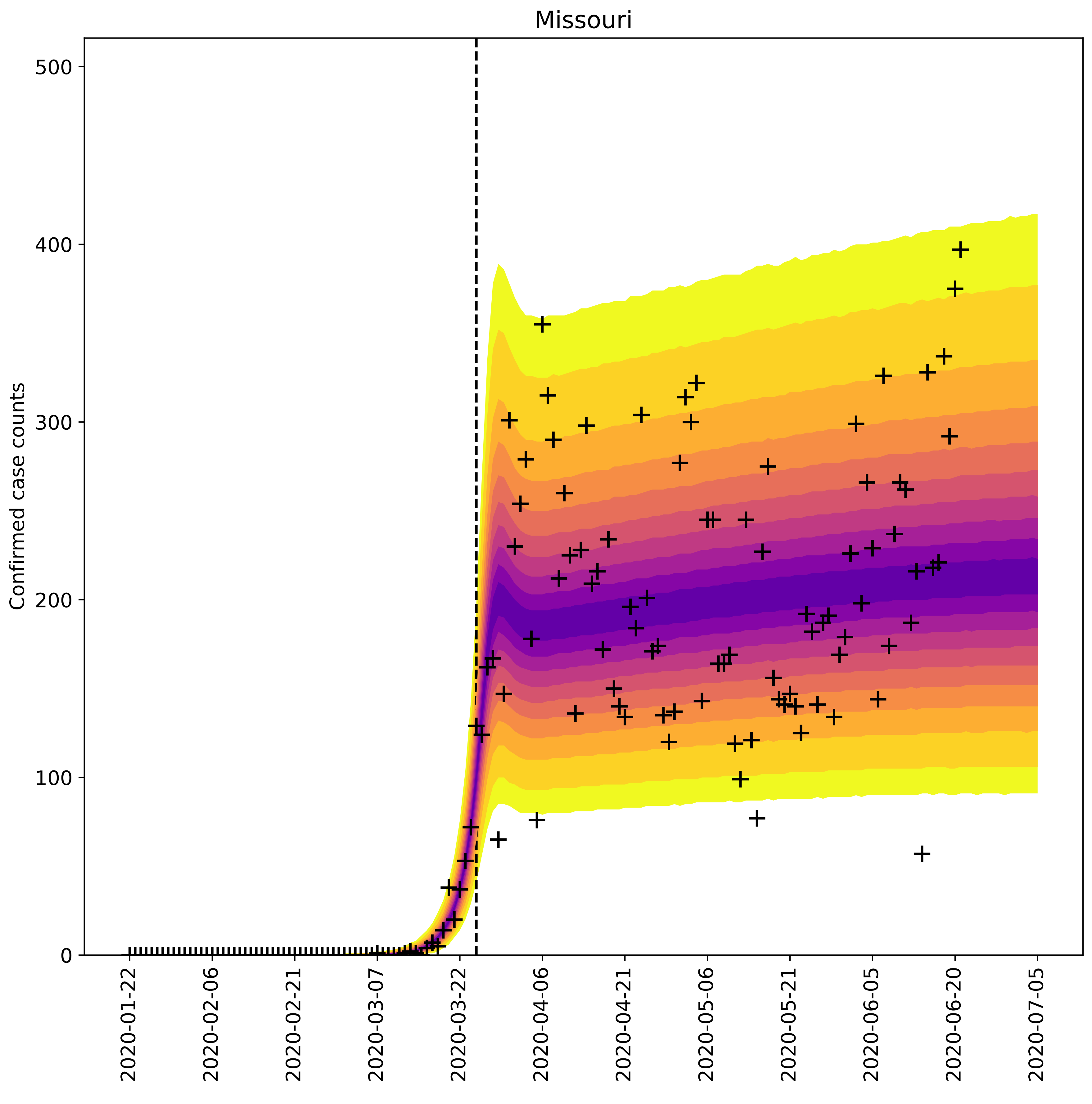

Missouri
Confirmed case counts
0
100
200
300
400
500
2020-01-22
2020-02-06
2020-02-21
2020-03-07
2020-03-22
2020-04-06
2020-04-21
2020-05-06
2020-05-21
2020-06-05
2020-06-20
2020-07-05

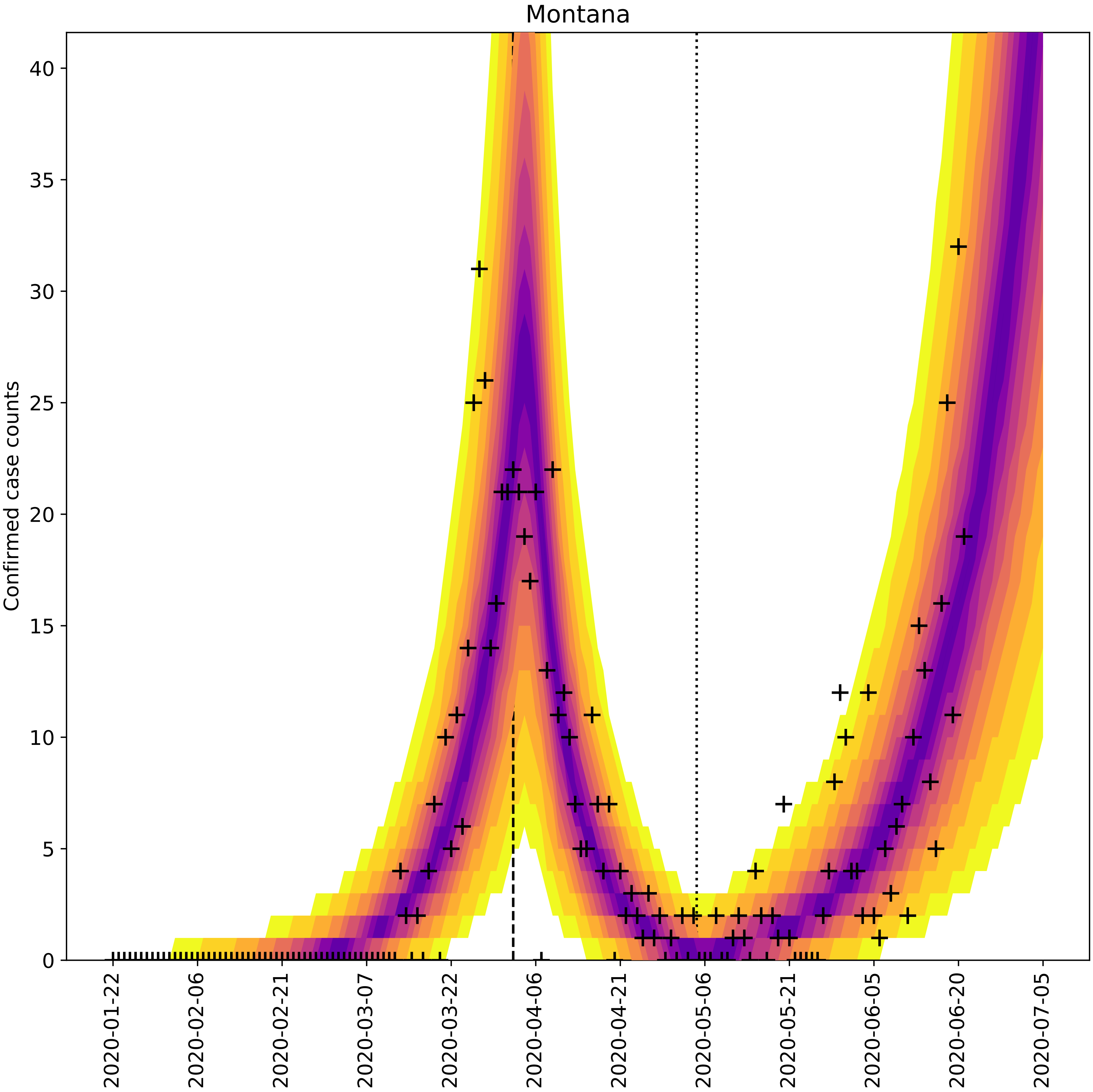

Montana
Confirmed case counts
0
5
10
15
20
25
30
35
40
2020-01-22
2020-02-06
2020-02-21
2020-03-07
2020-03-22
2020-04-06
2020-04-21
2020-05-06
2020-05-21
2020-06-05
2020-06-20
2020-07-05

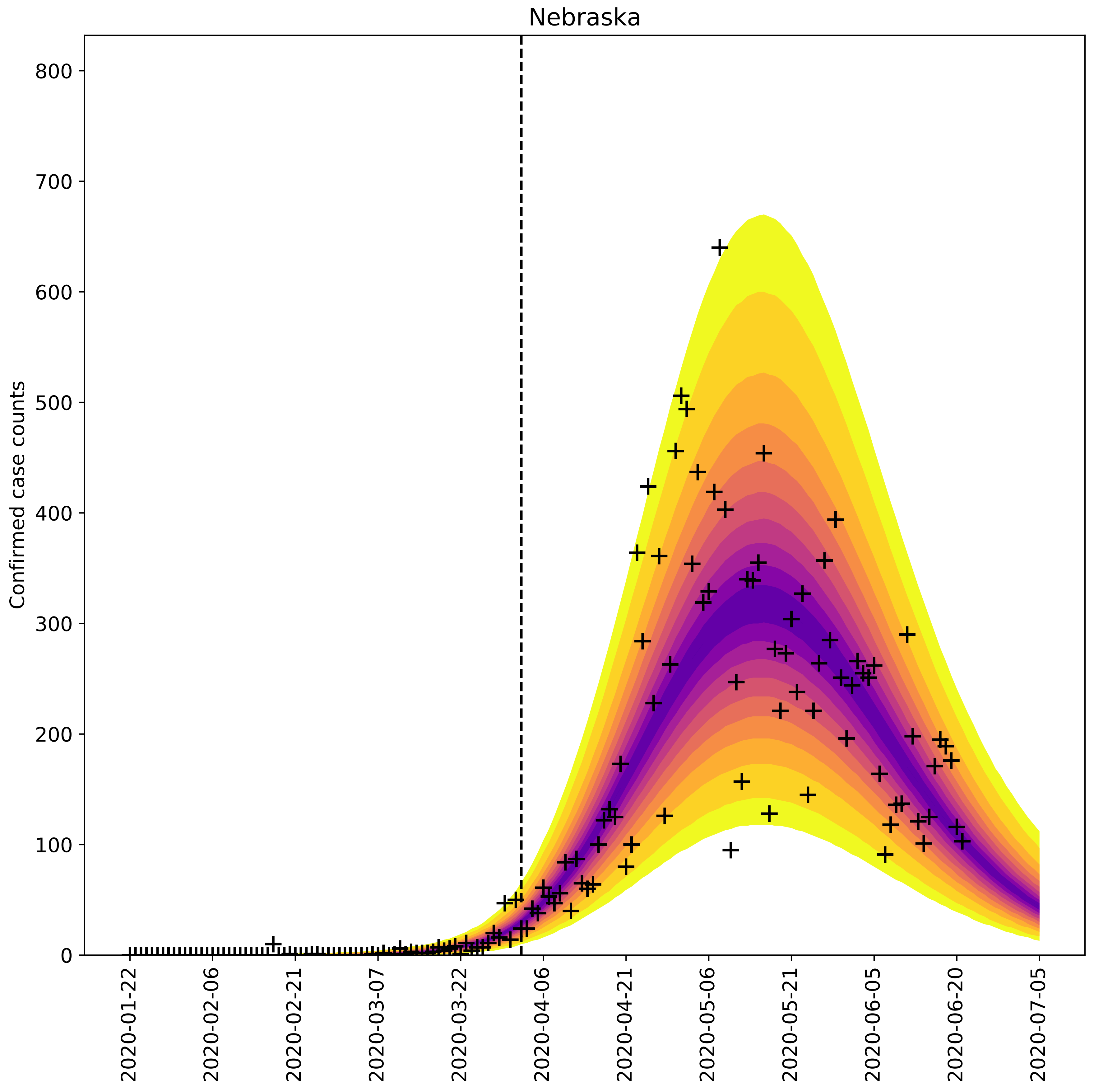

Nebraska
Confirmed case counts
0
100
200
300
400
500
600
700
800
2020-01-22
2020-02-06
2020-02-21
2020-03-07
2020-03-22
2020-04-06
2020-04-21
2020-05-06
2020-05-21
2020-06-05
2020-06-20
2020-07-05

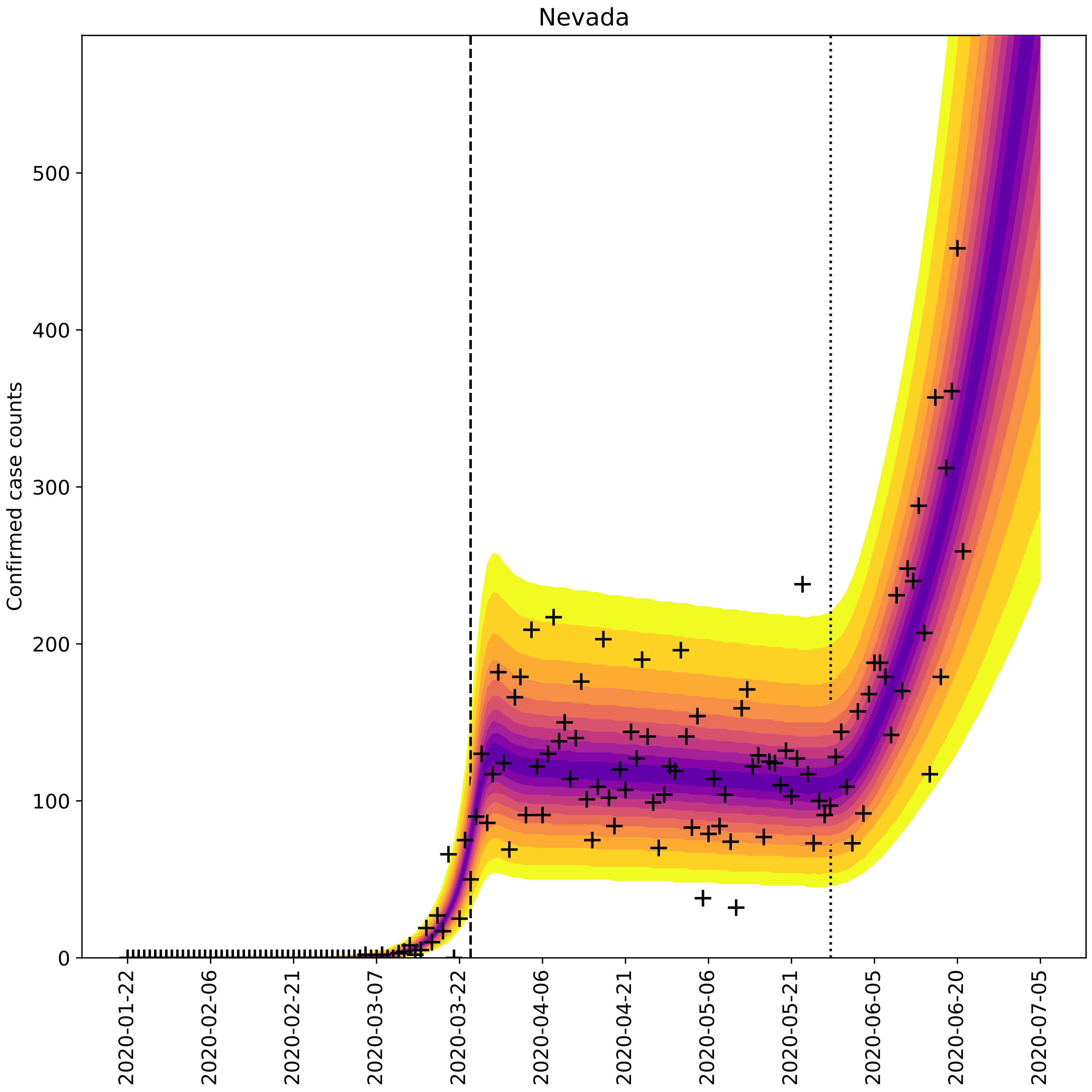

Nevada
Confirmed case counts
0
100
200
300
400
500
2020-01-22
2020-02-06
2020-02-21
2020-03-07
2020-03-22
2020-04-06
2020-04-21
2020-05-06
2020-05-21
2020-06-05
2020-06-20
2020-07-05

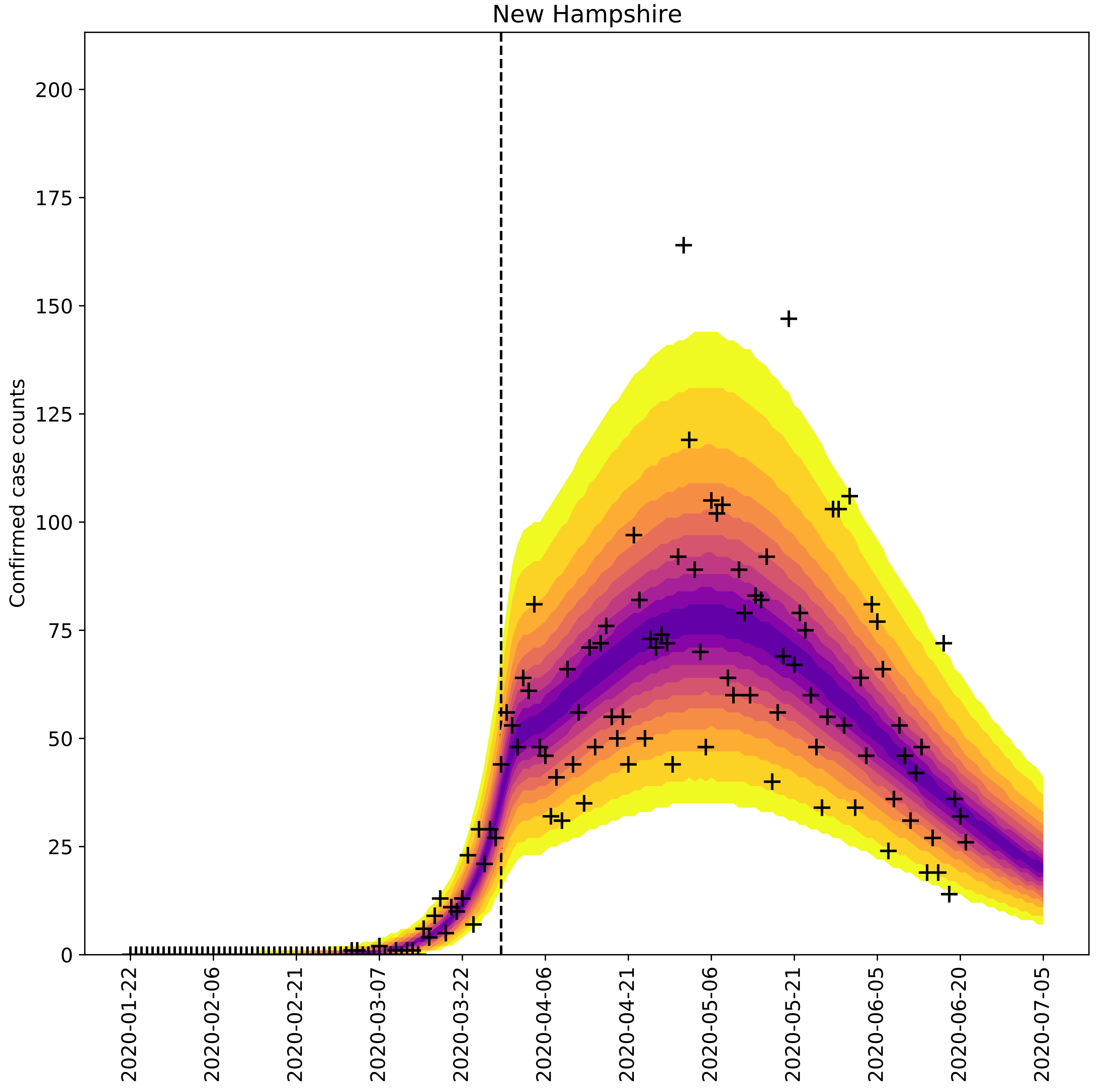
New Hampshire
Confirmed case counts
0
25
50
75
100
125
150
175
200
2020-01-22
2020-02-06
2020-02-21
2020-03-07
2020-03-22
2020-04-06
2020-04-21
2020-05-06
2020-05-21
2020-06-05
2020-06-20
2020-07-05

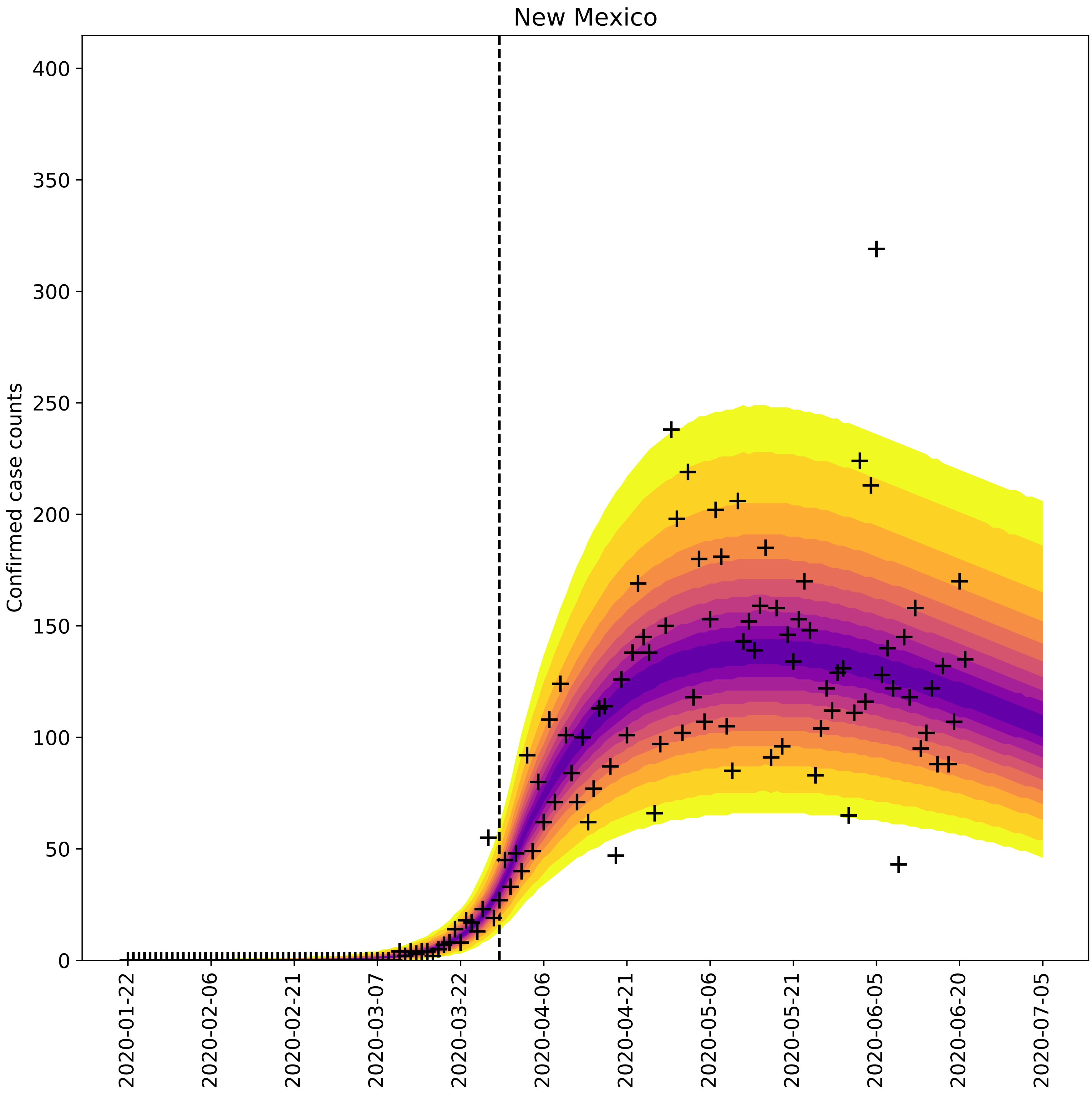

New Mexico
Confirmed case counts
0
50
100
150
200
250
300
350
400
2020-01-22
2020-02-06
2020-02-21
2020-03-07
2020-03-22
2020-04-06
2020-04-21
2020-05-06
2020-05-21
2020-06-05
2020-06-20
2020-07-05

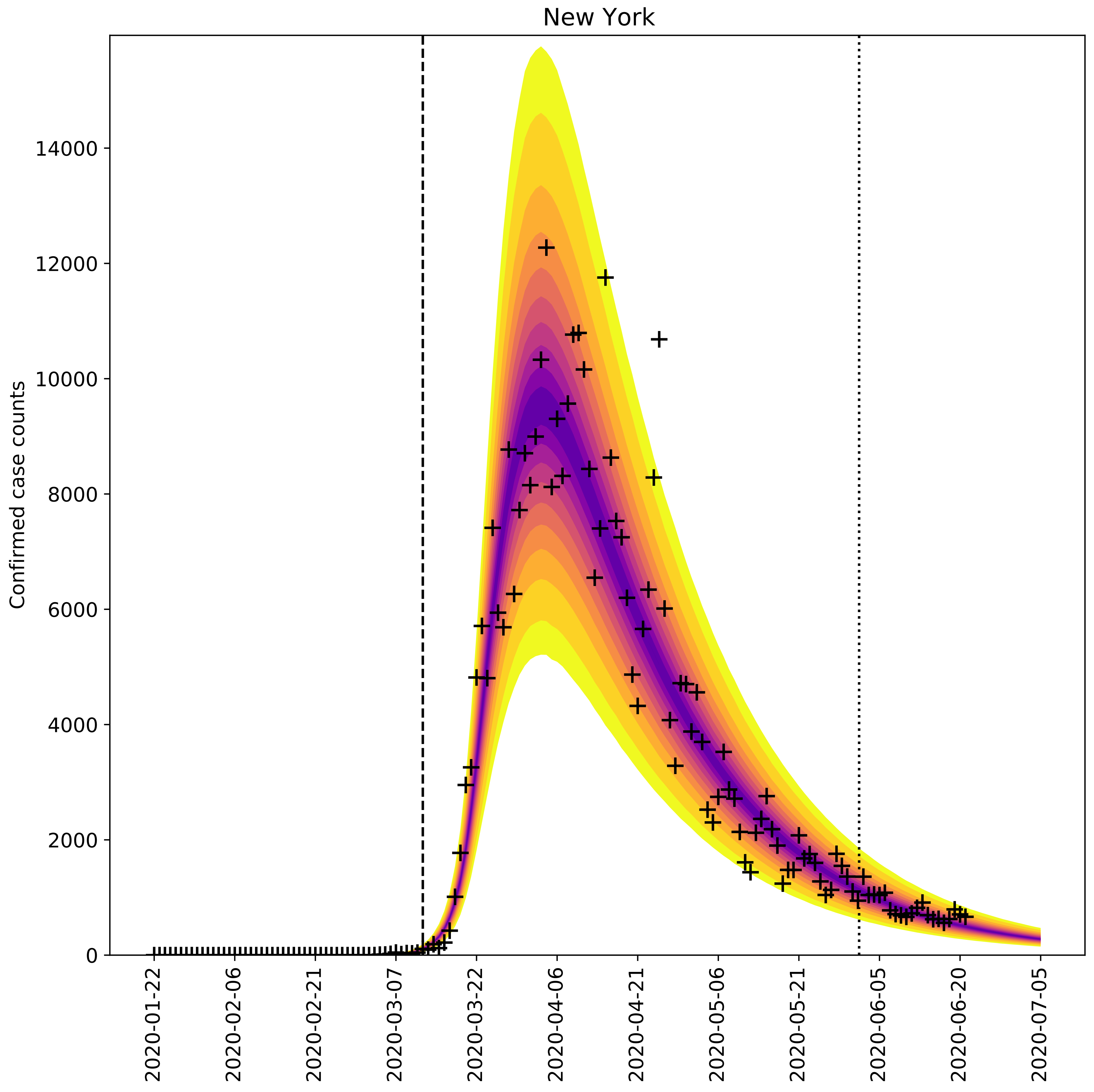
New York
Confirmed case counts
0
2000
4000
6000
8000
10000
12000
14000
2020-01-22
2020-02-06
2020-02-21
2020-03-07
2020-03-22
2020-04-06
2020-04-21
2020-05-06
2020-05-21
2020-06-05
2020-06-20
2020-07-05

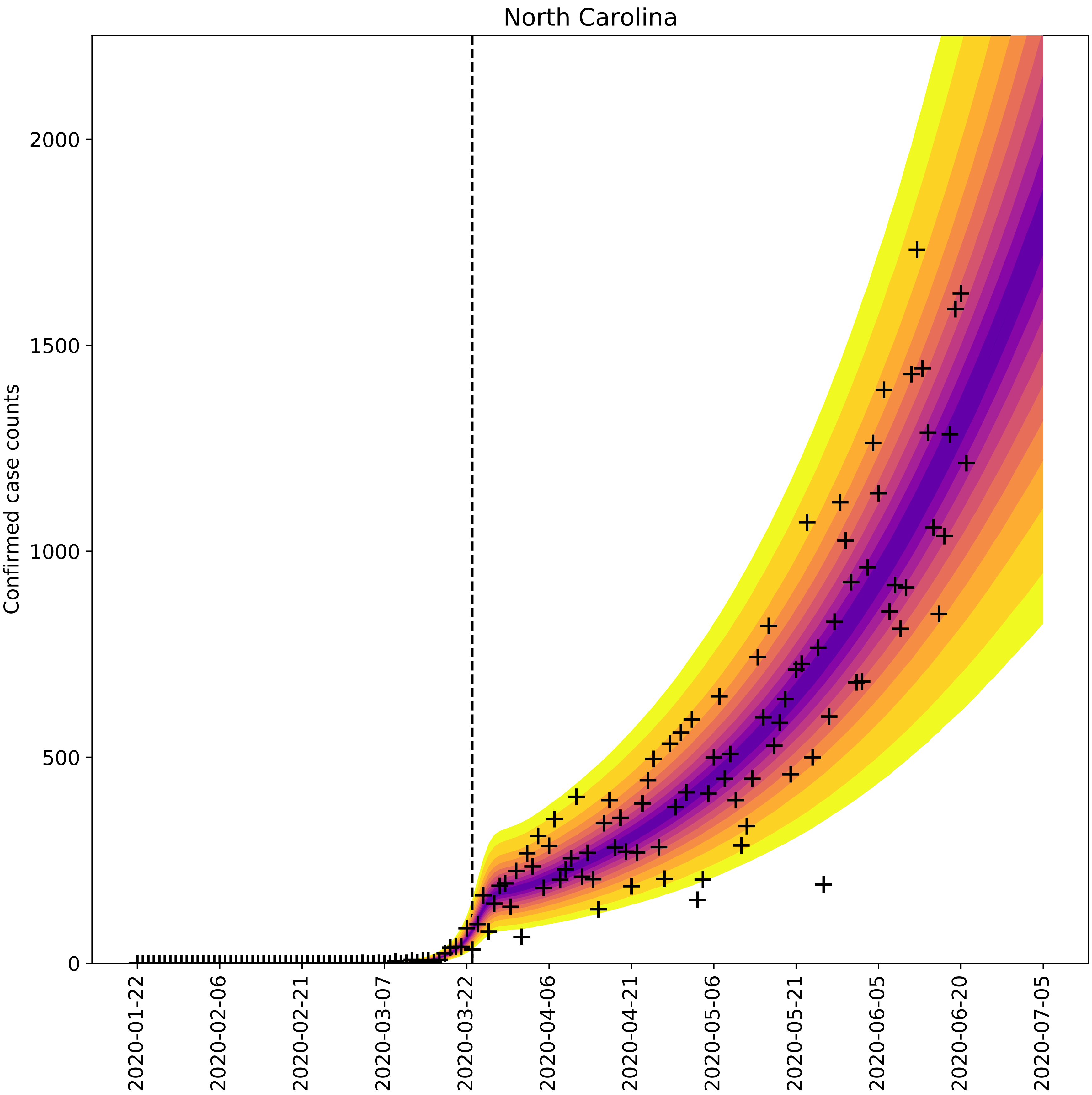

North Carolina
Confirmed case counts
0
500
1000
1500
2000
2020-01-22
2020-02-06
2020-02-21
2020-03-07
2020-03-22
2020-04-06
2020-04-21
2020-05-06
2020-05-21
2020-06-05
2020-06-20
2020-07-05

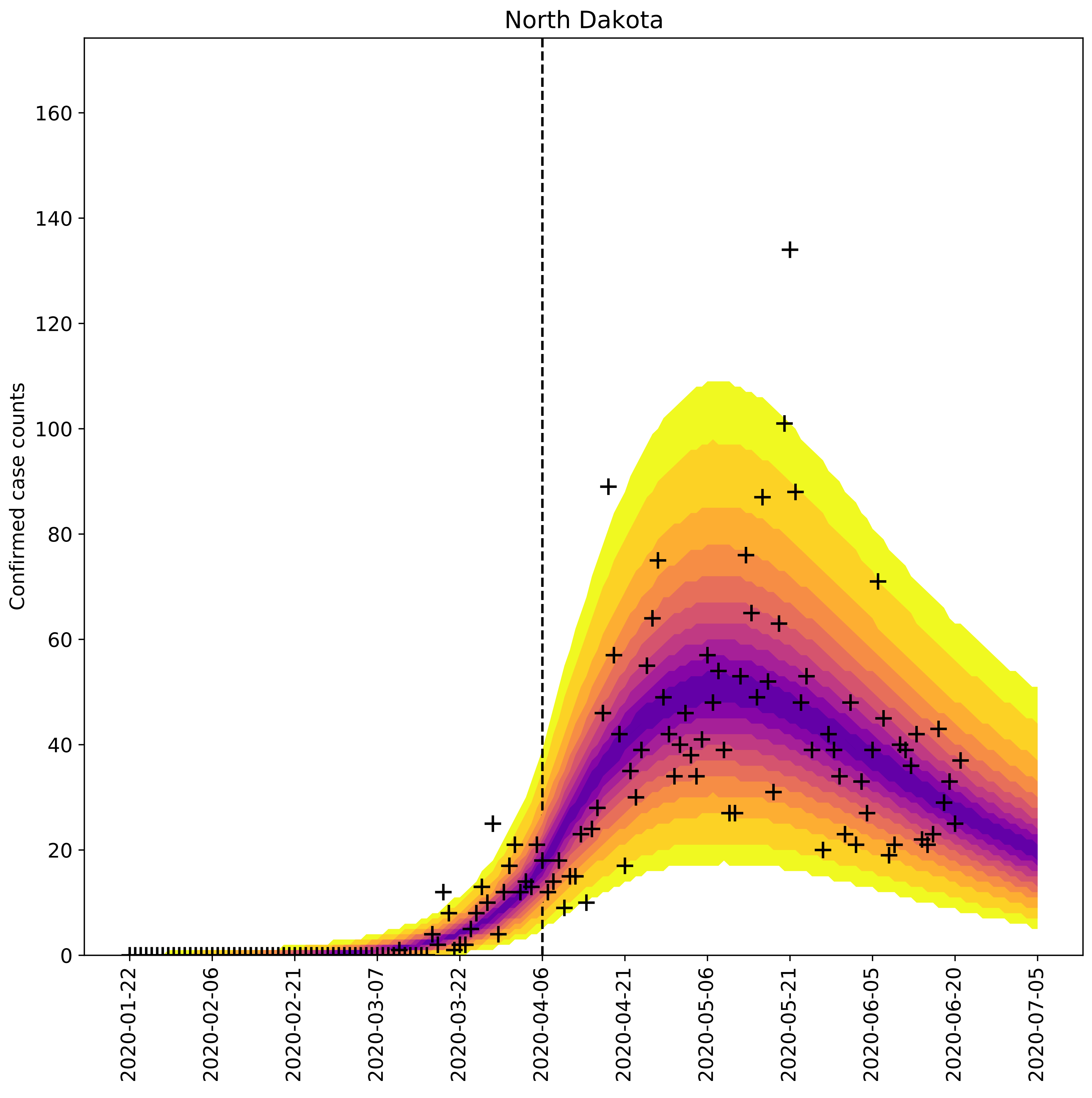

North Dakota
Confirmed case counts
0
20
40
60
80
100
120
140
160
2020-01-22
2020-02-06
2020-02-21
2020-03-07
2020-03-22
2020-04-06
2020-04-21
2020-05-06
2020-05-21
2020-06-05
2020-06-20
2020-07-05

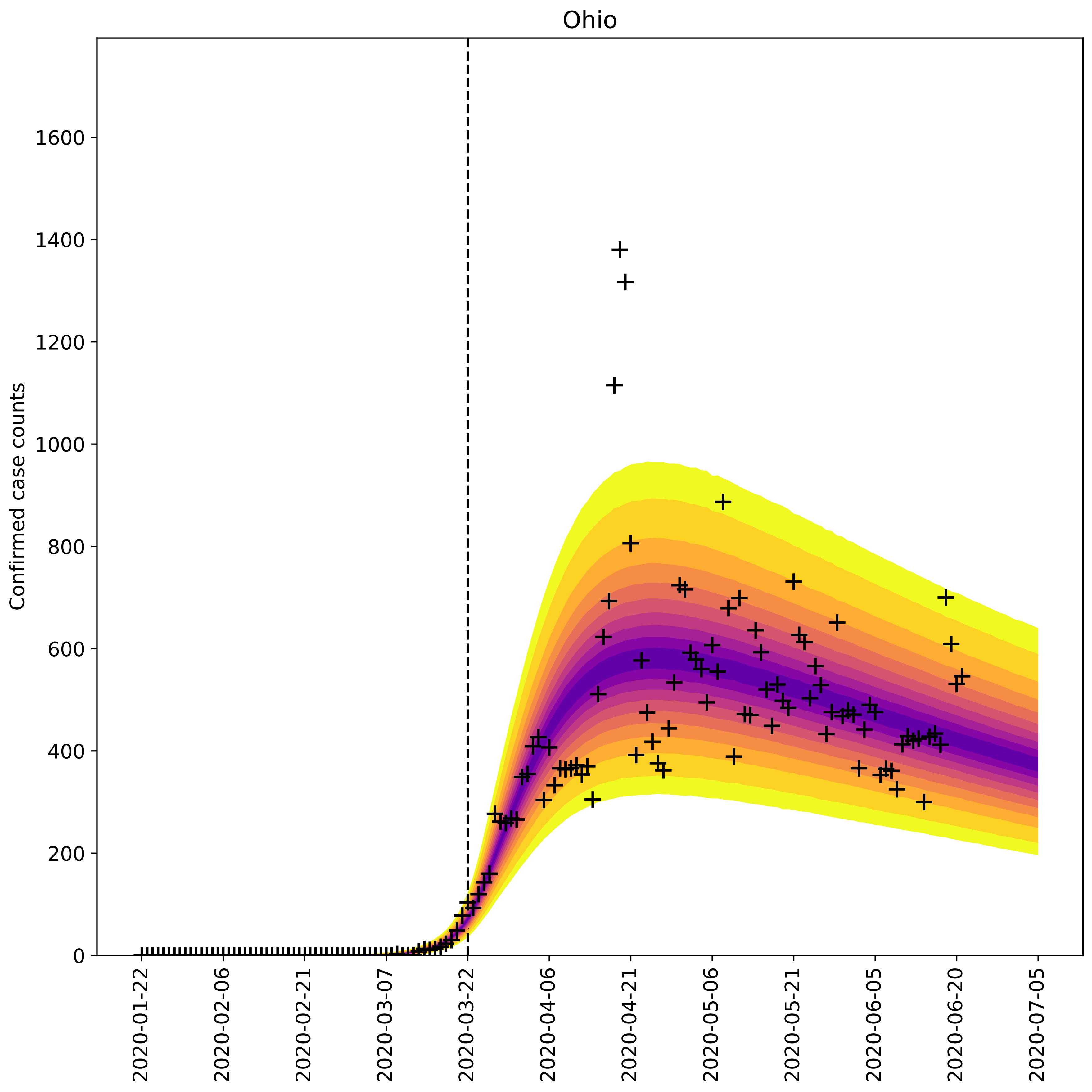

Ohio
Confirmed case counts
0
200
400
600
800
1000
1200
1400
1600
2020-01-22
2020-02-06
2020-02-21
2020-03-07
2020-03-22
2020-04-06
2020-04-21
2020-05-06
2020-05-21
2020-06-05
2020-06-20
2020-07-05

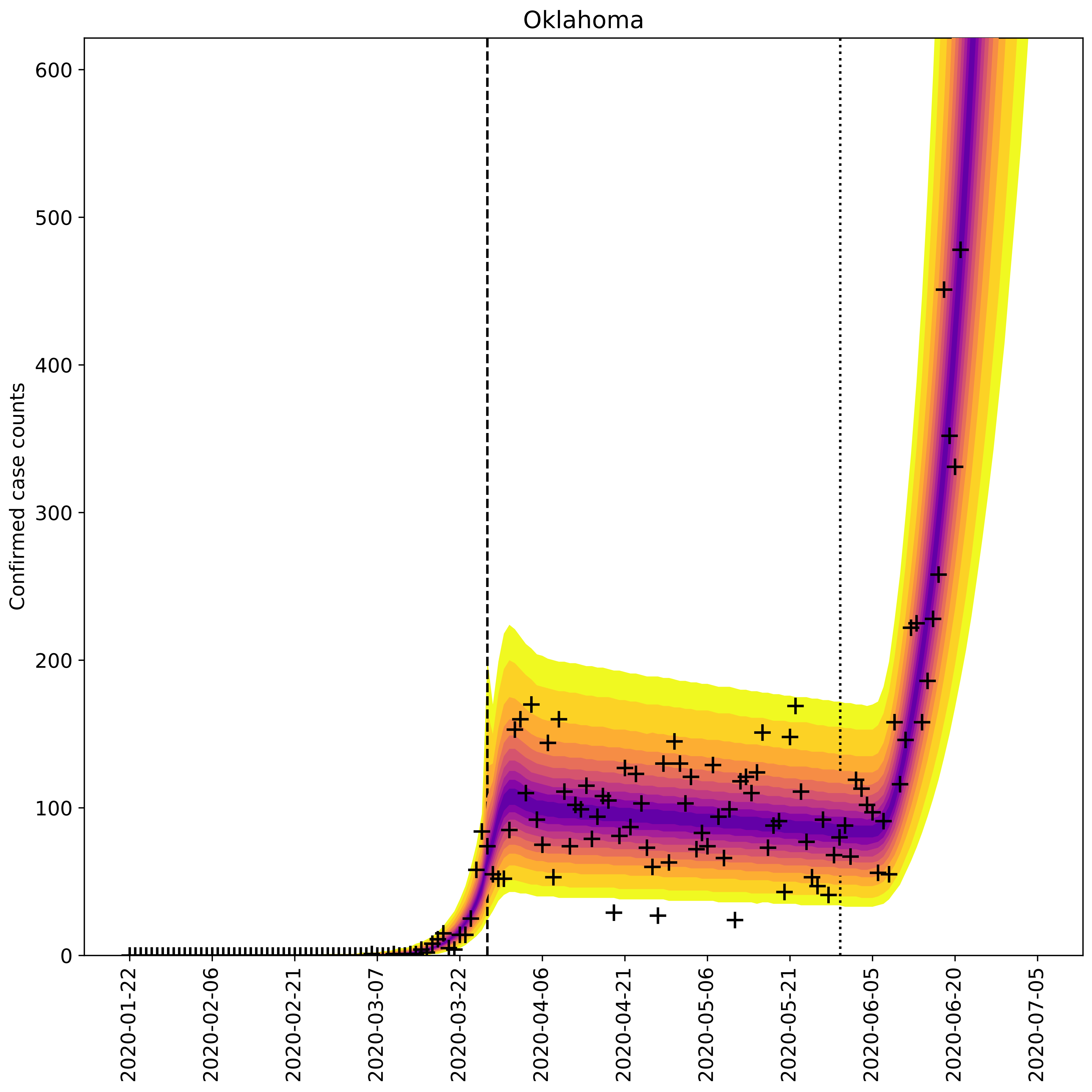
Oklahoma
Confirmed case counts
0
100
200
300
400
500
600
2020-01-22
2020-02-06
2020-02-21
2020-03-07
2020-03-22
2020-04-06
2020-04-21
2020-05-06
2020-05-21
2020-06-05
2020-06-20
2020-07-05

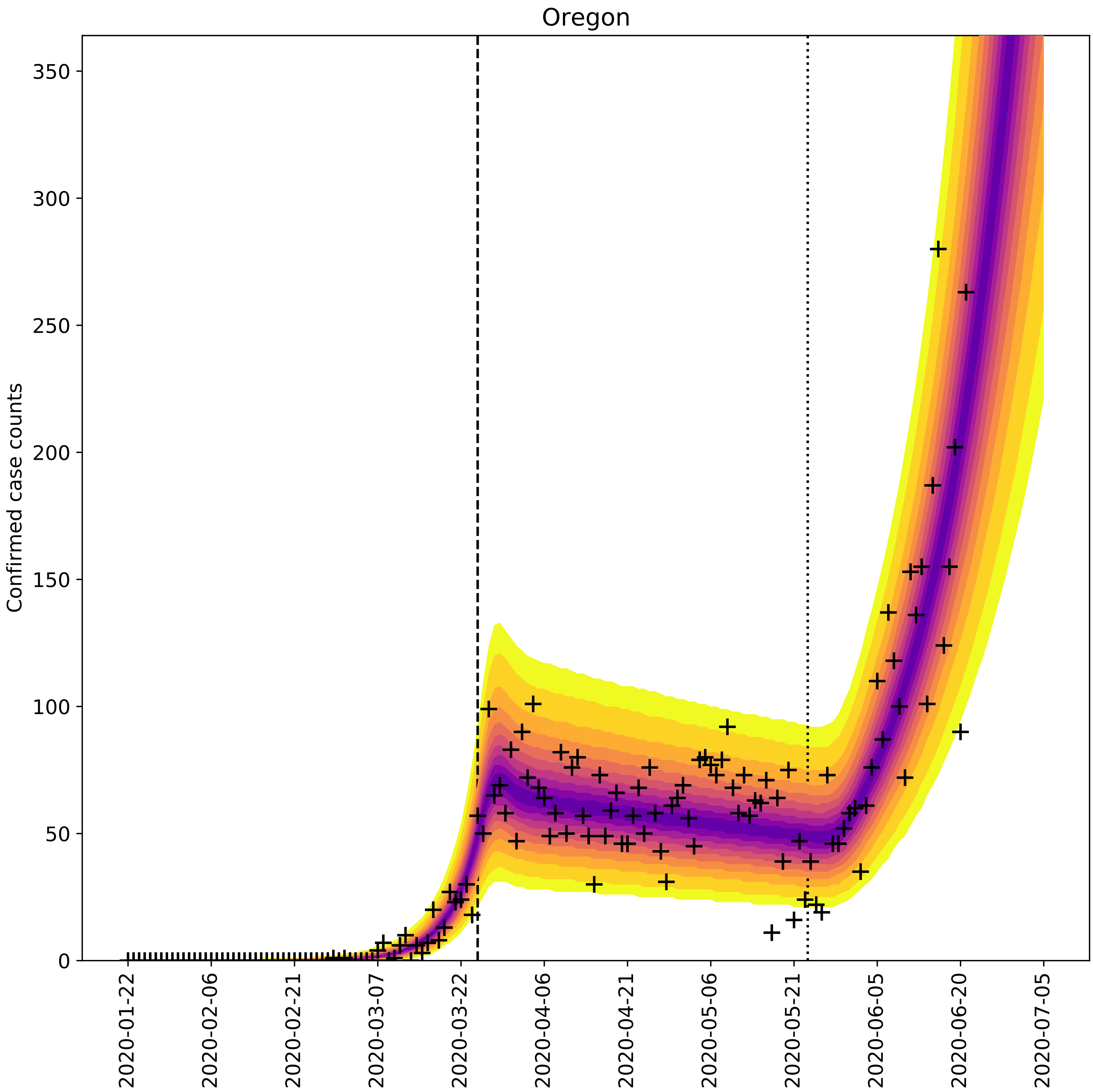
Oregon
Confirmed case counts
0
50
100
150
200
250
300
350
2020-01-22
2020-02-06
2020-02-21
2020-03-07
2020-03-22
2020-04-06
2020-04-21
2020-05-06
2020-05-21
2020-06-05
2020-06-20
2020-07-05

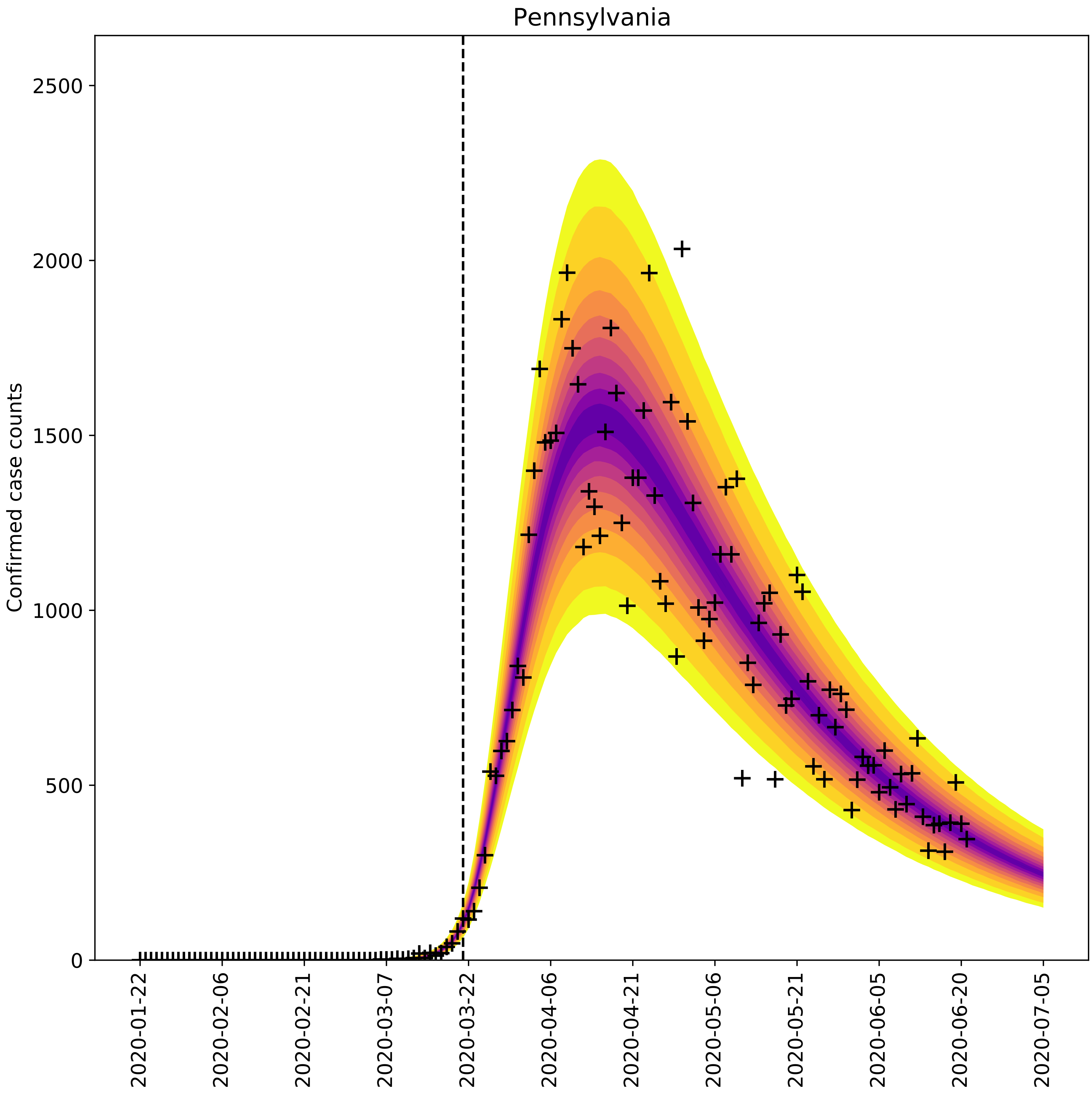

Pennsylvania
Confirmed case counts
0
500
1000
1500
2000
2500
2020-01-22
2020-02-06
2020-02-21
2020-03-07
2020-03-22
2020-04-06
2020-04-21
2020-05-06
2020-05-21
2020-06-05
2020-06-20
2020-07-05

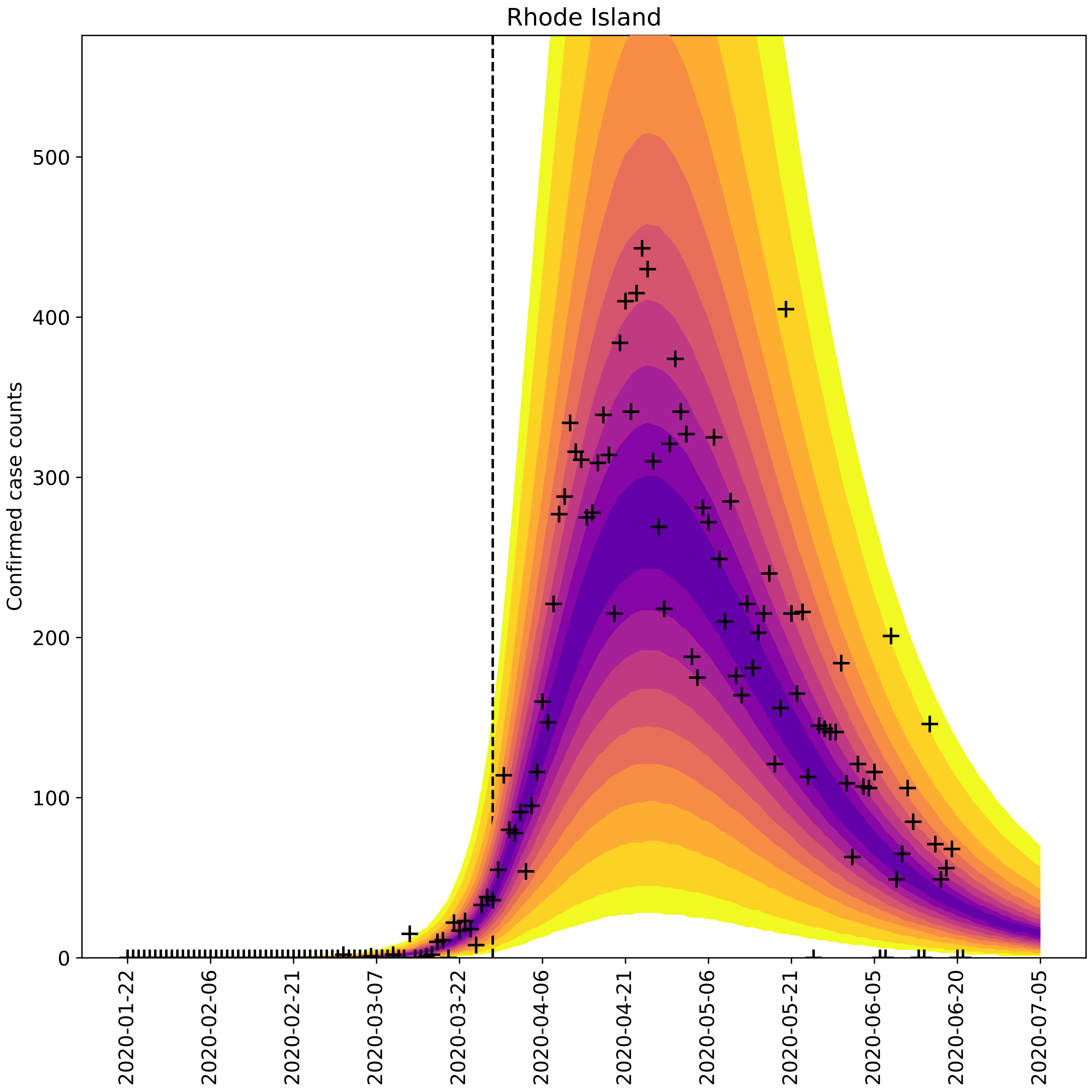

Rhode Island
Confirmed case counts
0
100
200
300
400
500
2020-01-22
2020-02-06
2020-02-21
2020-03-07
2020-03-22
2020-04-06
2020-04-21
2020-05-06
2020-05-21
2020-06-05
2020-06-20
2020-07-05

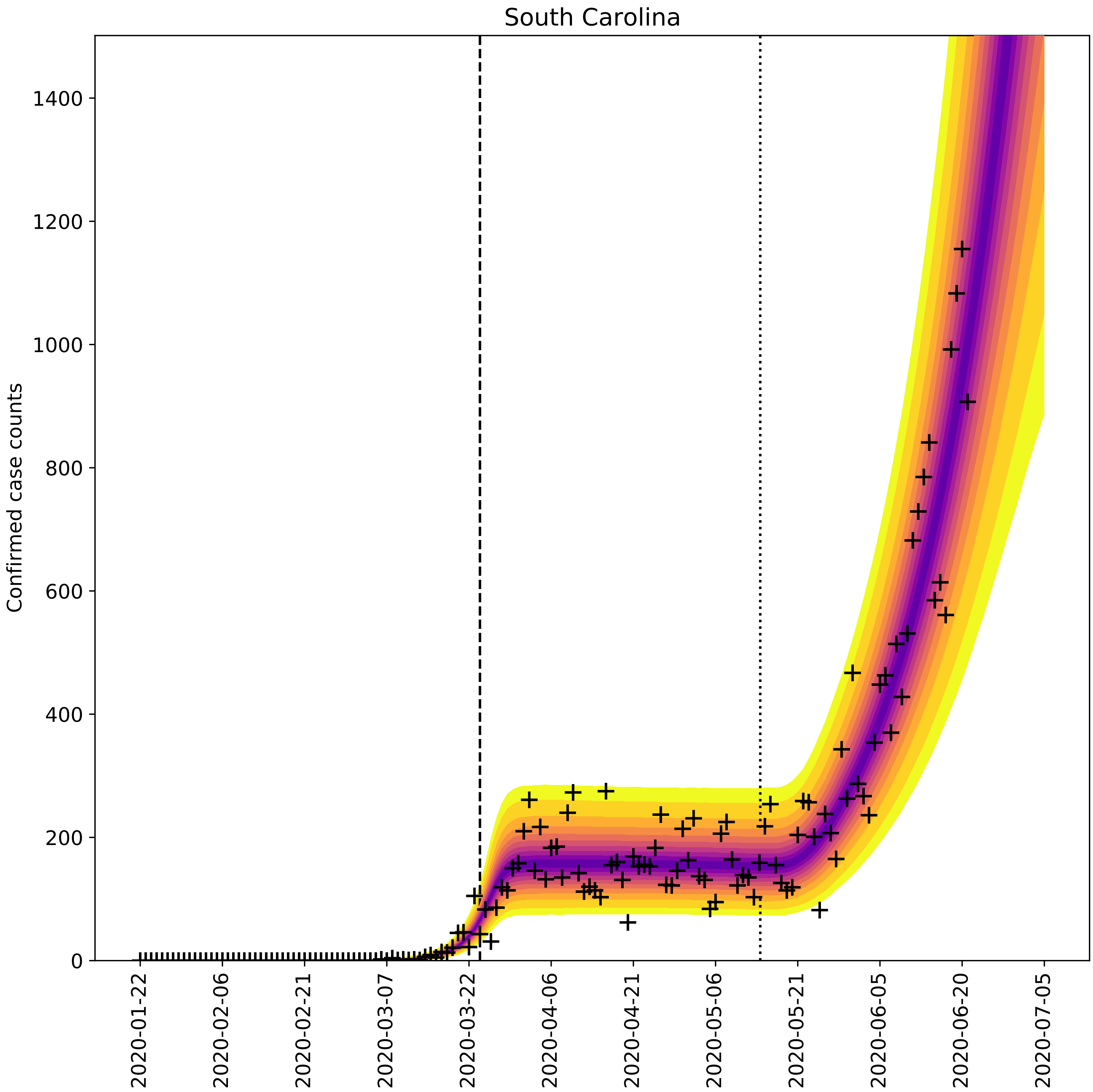
South Carolina
Confirmed case counts
0
200
400
600
800
1000
1200
1400
2020-01-22
2020-02-06
2020-02-21
2020-03-07
2020-03-22
2020-04-06
2020-04-21
2020-05-06
2020-05-21
2020-06-05
2020-06-20
2020-07-05

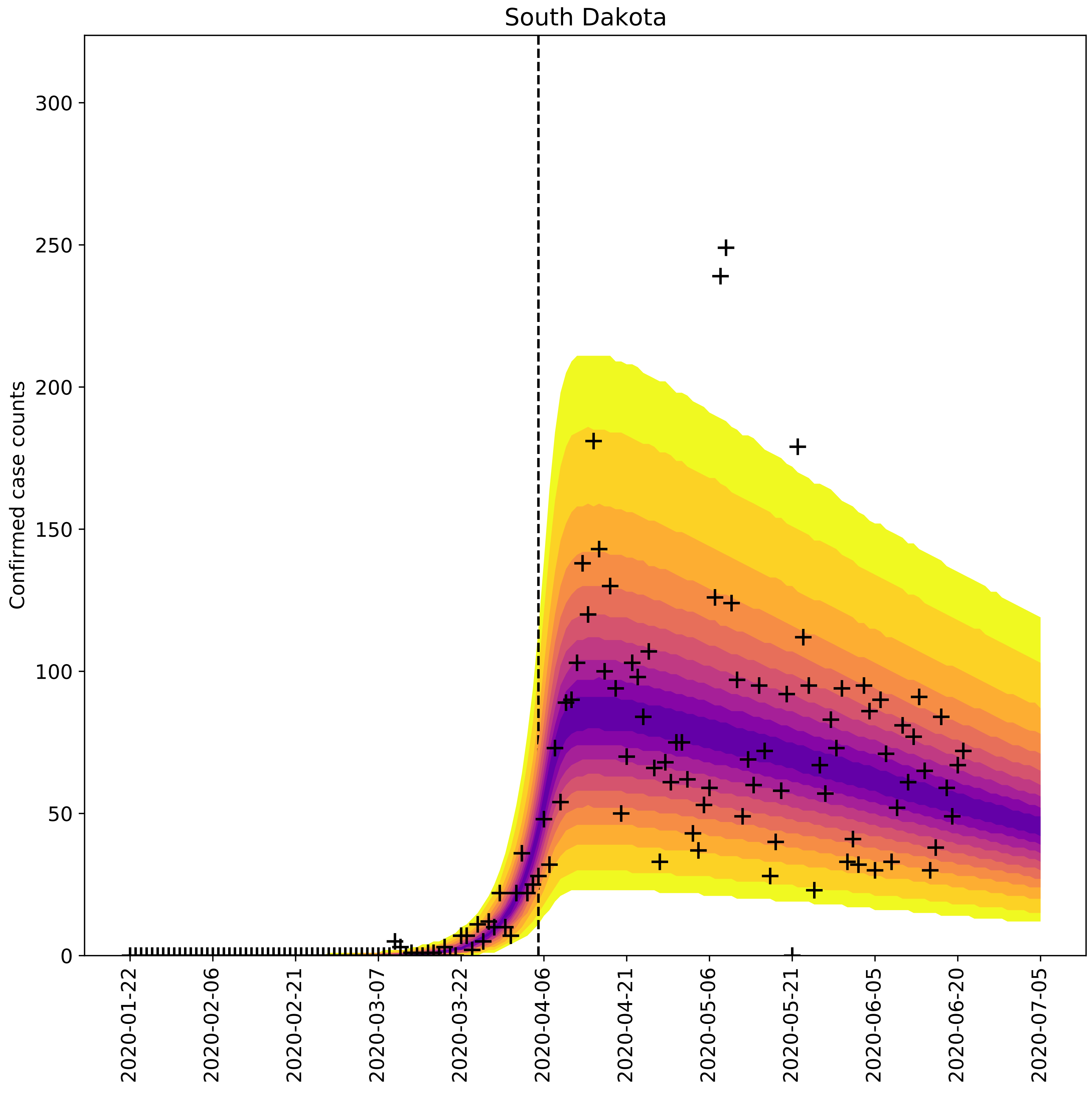

South Dakota
Confirmed case counts
0
50
100
150
200
250
300
2020-01-22
2020-02-06
2020-02-21
2020-03-07
2020-03-22
2020-04-06
2020-04-21
2020-05-06
2020-05-21
2020-06-05
2020-06-20
2020-07-05

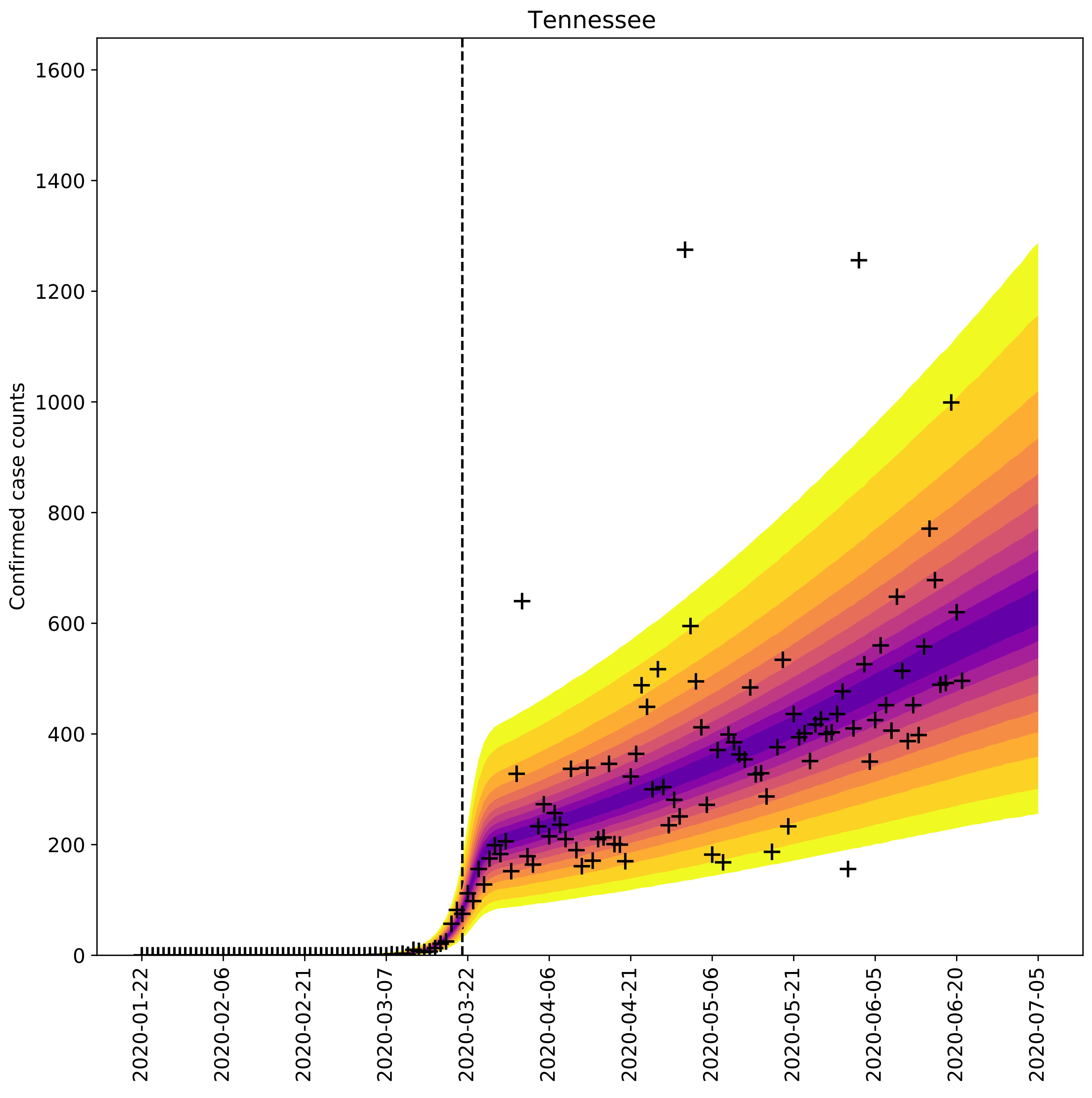

Tennessee
Confirmed case counts
0
200
400
600
800
1000
1200
1400
1600
2020-01-22
2020-02-06
2020-02-21
2020-03-07
2020-03-22
2020-04-06
2020-04-21
2020-05-06
2020-05-21
2020-06-05
2020-06-20
2020-07-05

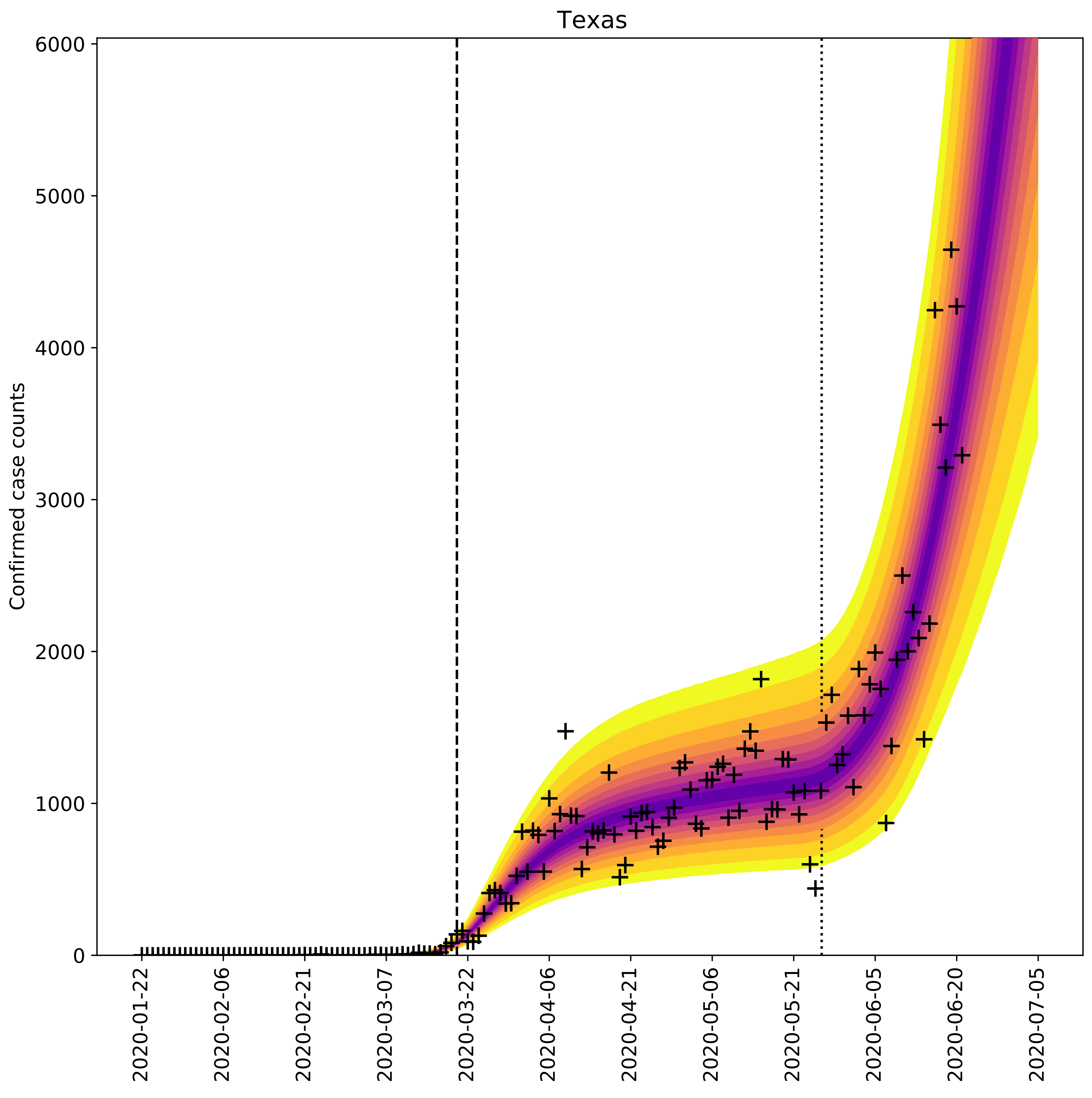
Texas
Confirmed case counts
6000
5000
4000
3000
2000
1000
0
2020-01-22
2020-02-06
2020-02-21
2020-03-07
2020-03-22
2020-04-06
2020-04-21
2020-05-06
2020-05-21
2020-06-05
2020-06-20
2020-07-05

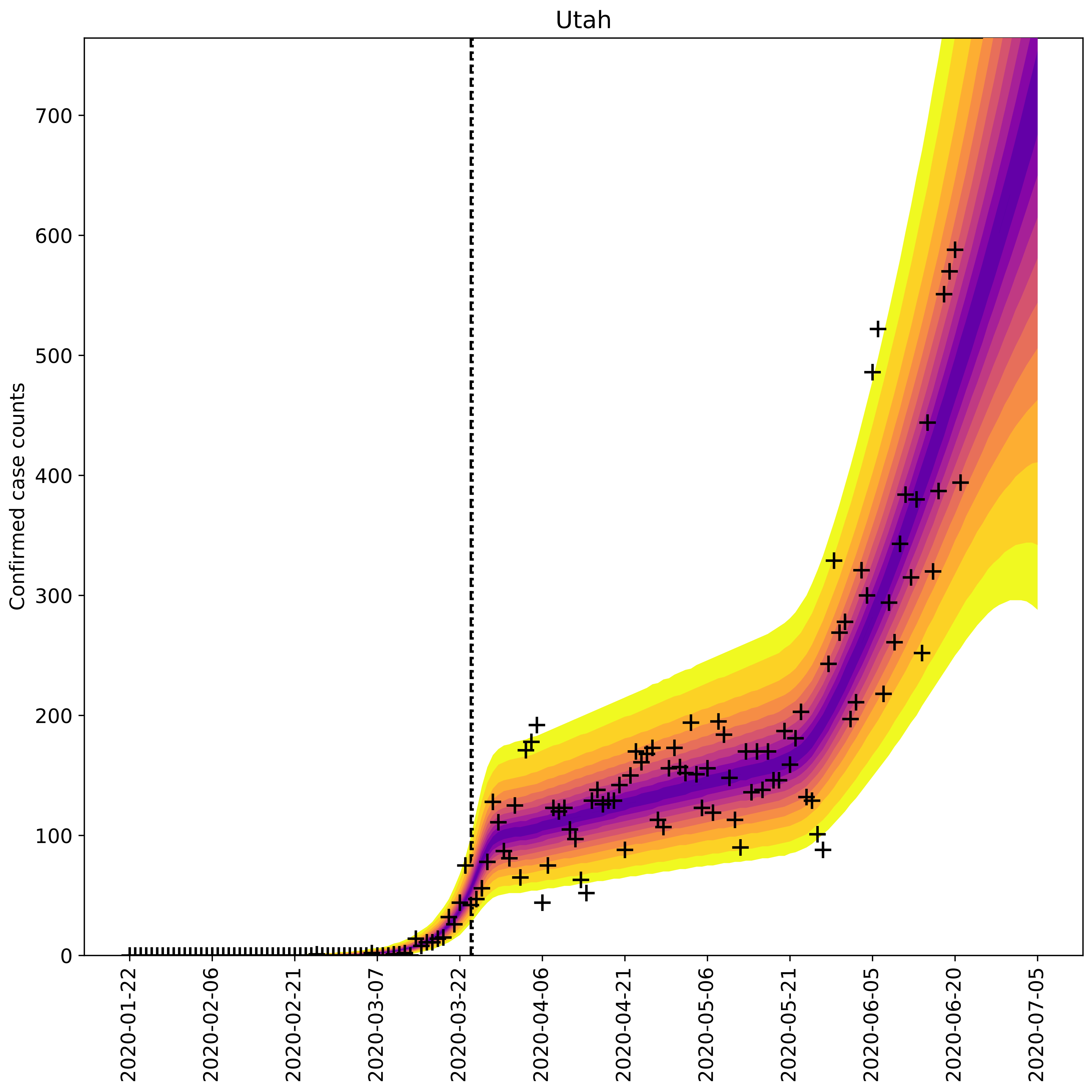

Utah
Confirmed case counts
0
100
200
300
400
500
600
700
2020-01-22
2020-02-06
2020-02-21
2020-03-07
2020-03-22
2020-04-06
2020-04-21
2020-05-06
2020-05-21
2020-06-05
2020-06-20
2020-07-05

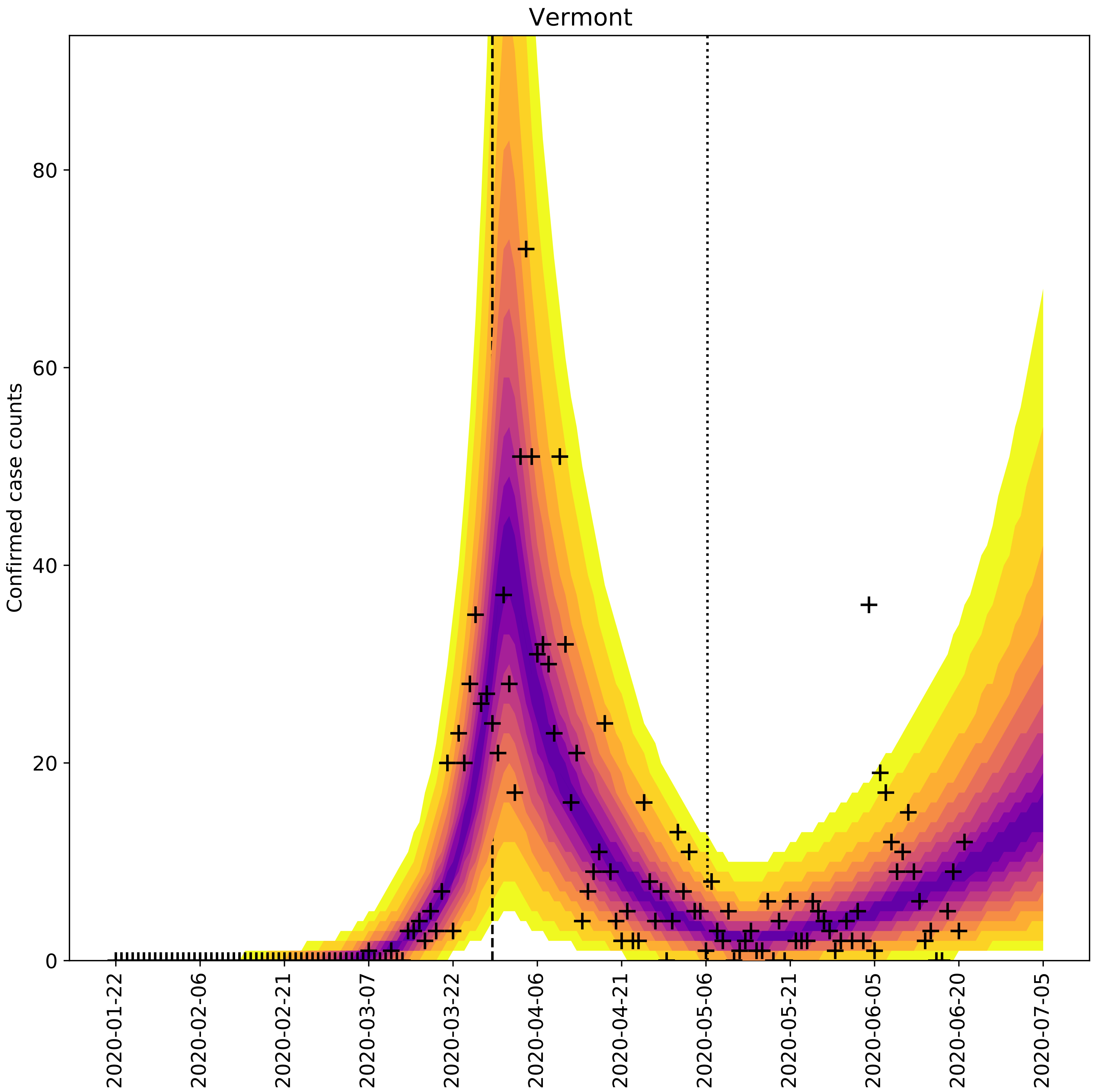

Vermont
Confirmed case counts
0
20
40
60
80
2020-01-22
2020-02-06
2020-02-21
2020-03-07
2020-03-22
2020-04-06
2020-04-21
2020-05-06
2020-05-21
2020-06-05
2020-06-20
2020-07-05

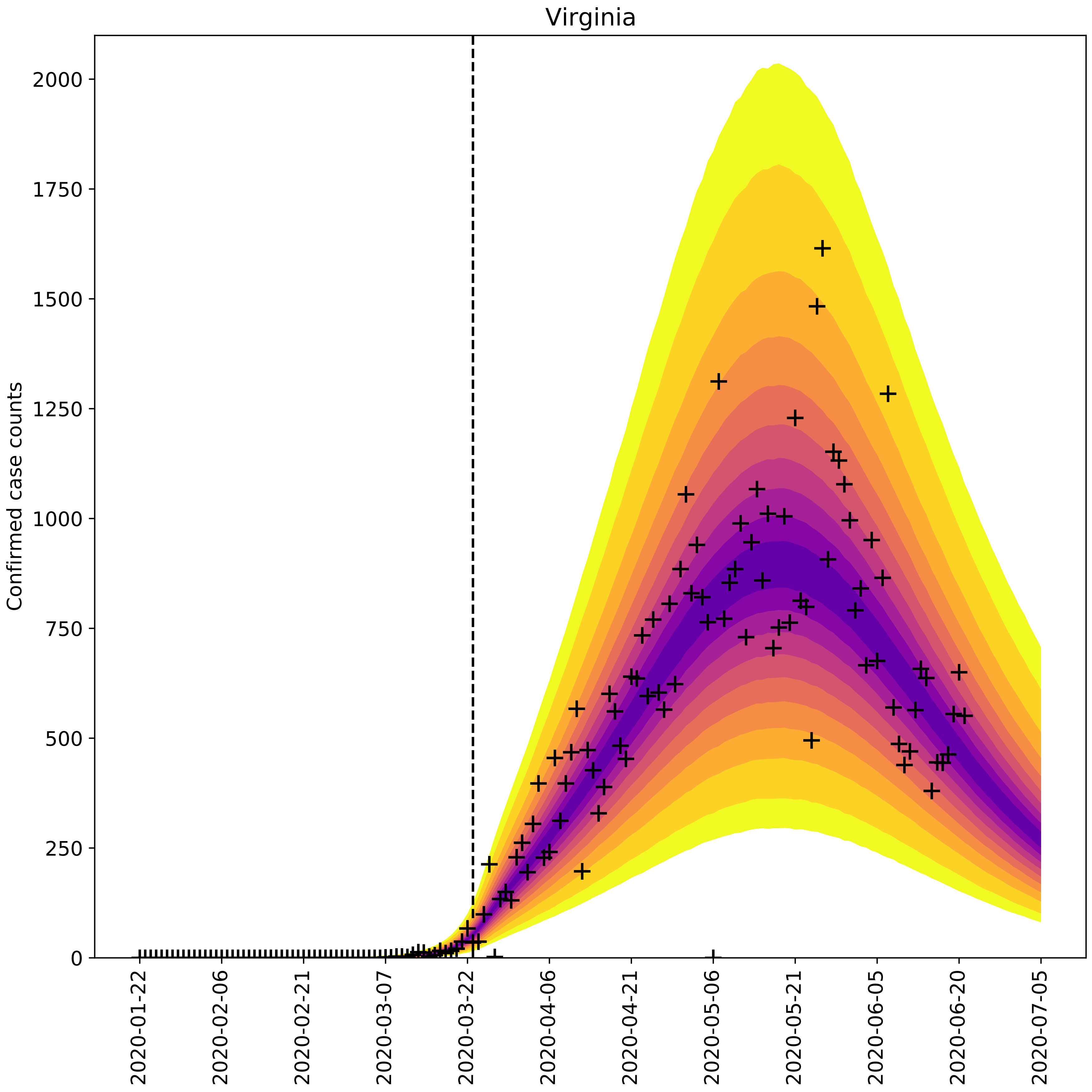

Virginia
Confirmed case counts
0
250
500
750
1000
1250
1500
1750
2000
2020-01-22
2020-02-06
2020-02-21
2020-03-07
2020-03-22
2020-04-06
2020-04-21
2020-05-06
2020-05-21
2020-06-05
2020-06-20
2020-07-05

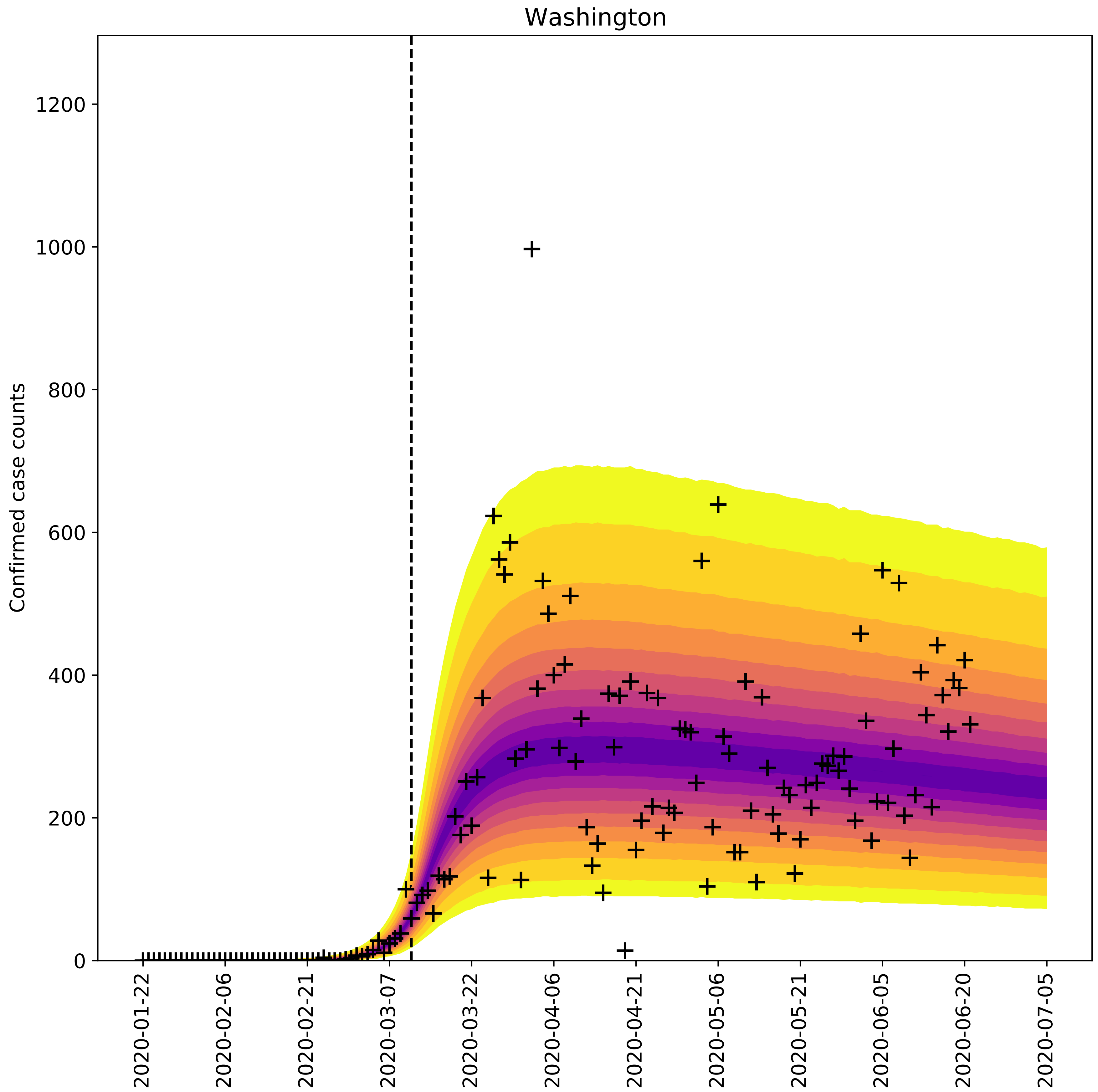

Washington
Confirmed case counts
0
200
400
600
800
1000
1200
2020-01-22
2020-02-06
2020-02-21
2020-03-07
2020-03-22
2020-04-06
2020-04-21
2020-05-06
2020-05-21
2020-06-05
2020-06-20
2020-07-05

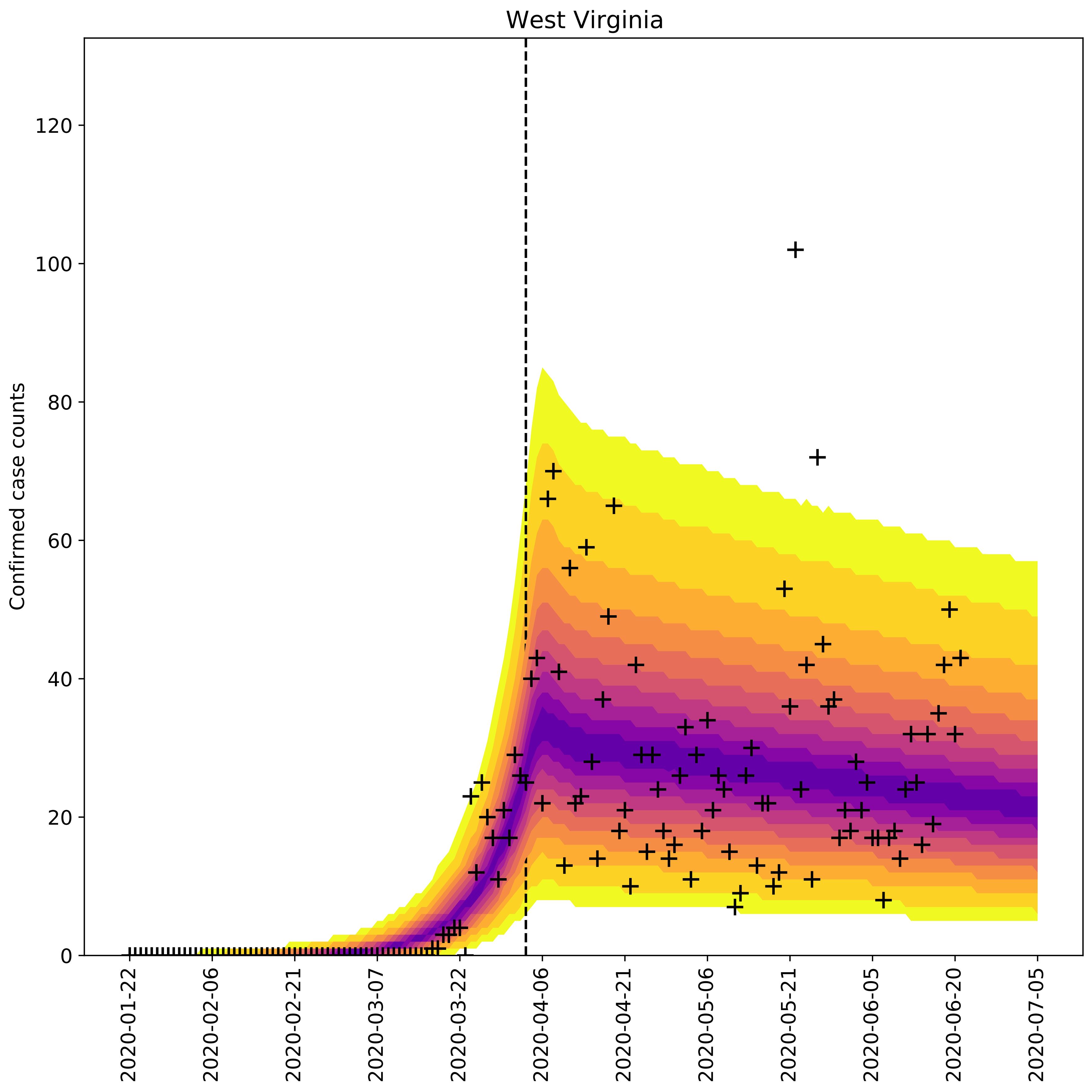

West Virginia
Confirmed case counts
0
20
40
60
80
100
120
2020-01-22
2020-02-06
2020-02-21
2020-03-07
2020-03-22
2020-04-06
2020-04-21
2020-05-06
2020-05-21
2020-06-05
2020-06-20
2020-07-05

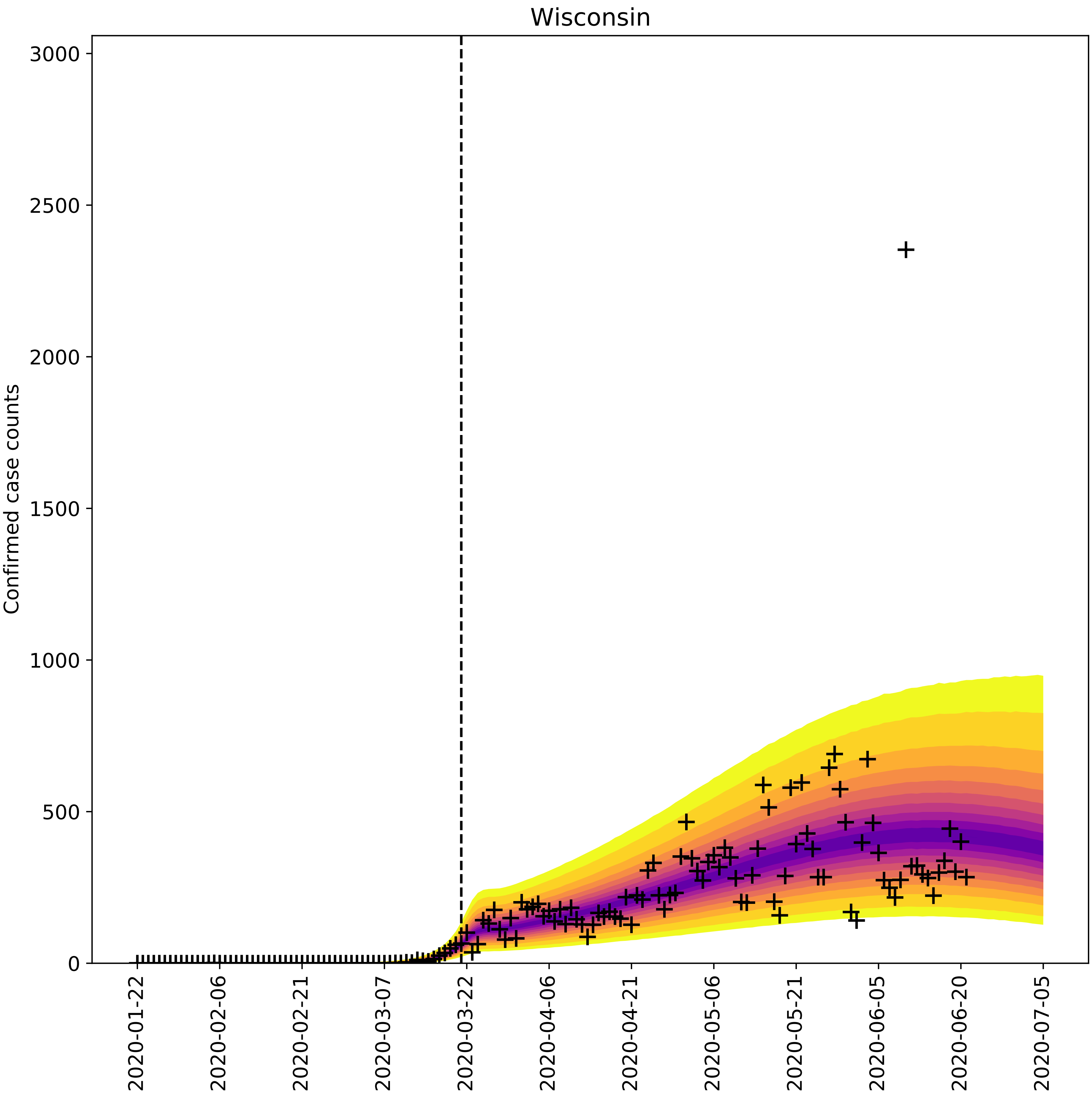

Wisconsin
Confirmed case counts
0
500
1000
1500
2000
2500
3000
2020-01-22
2020-02-06
2020-02-21
2020-03-07
2020-03-22
2020-04-06
2020-04-21
2020-05-06
2020-05-21
2020-06-05
2020-06-20
2020-07-05

Figure S6. Consistency of model-derived λ estimates with empirical growth rates during initial exponential increase in disease incidence in 46 states of the US (i.e., excluding New Jersey, Wyoming, Florida, and Alaska; see Fig 3 in the main text). In each panel, the initial slope of the solid curve corresponds to λ (calculated as described in Materials and Methods), the crosses indicate empirical cumulative case counts, and the broken line is the model prediction based on MAP estimates for adjustable parameters. The solid curve is derived from the reduced model (Eqs. 1-8 in the SI). This curve shows cumulative case counts had there not been any interventions to limit disease transmission. As can be seen, the initial slopes of the solid and broken curves are comparable. It should be noted that, in contrast with Fig S5, the y-axis here indicates cumulative (vs. daily) number of cases on a logarithmic (vs. linear) scale.

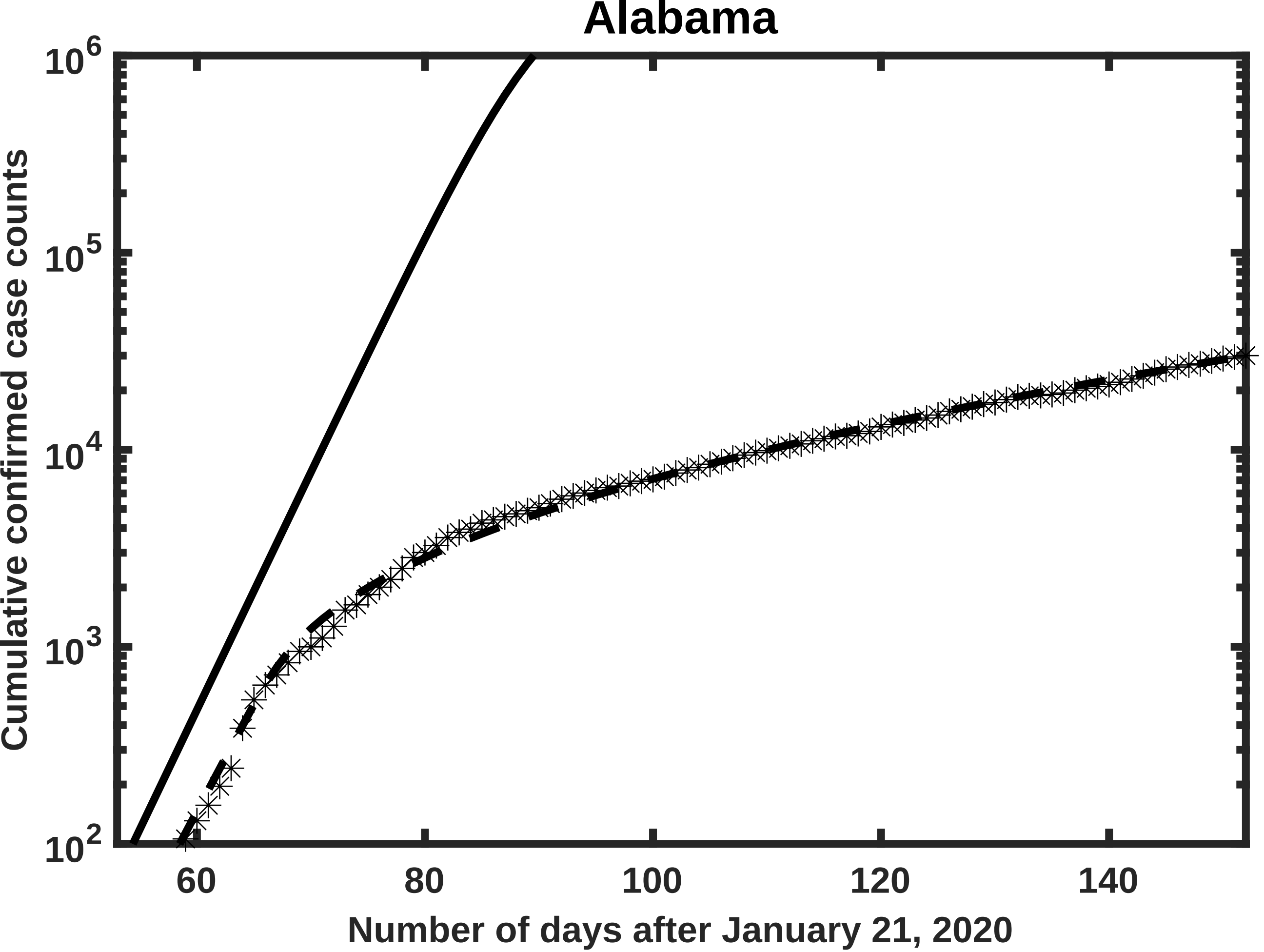

Alabama
Cumulative confirmed case counts
Number of days after January 21, 2020
10^6
10^5
10^4
10^3
10^2
60
80
100
120
140

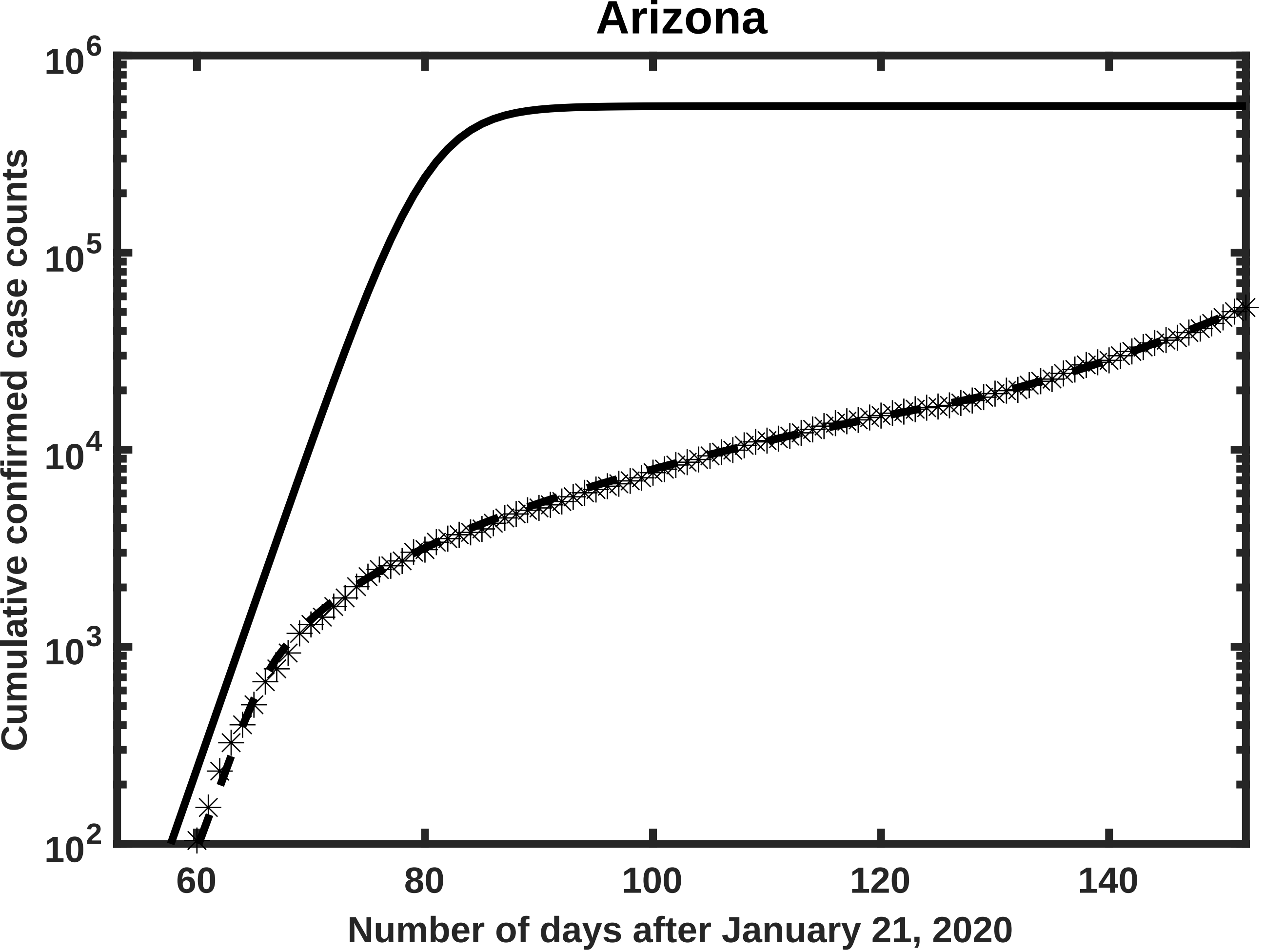

Arizona
Cumulative confirmed case counts
10^6
10^5
10^4
10^3
10^2
60
80
100
120
140
Number of days after January 21, 2020

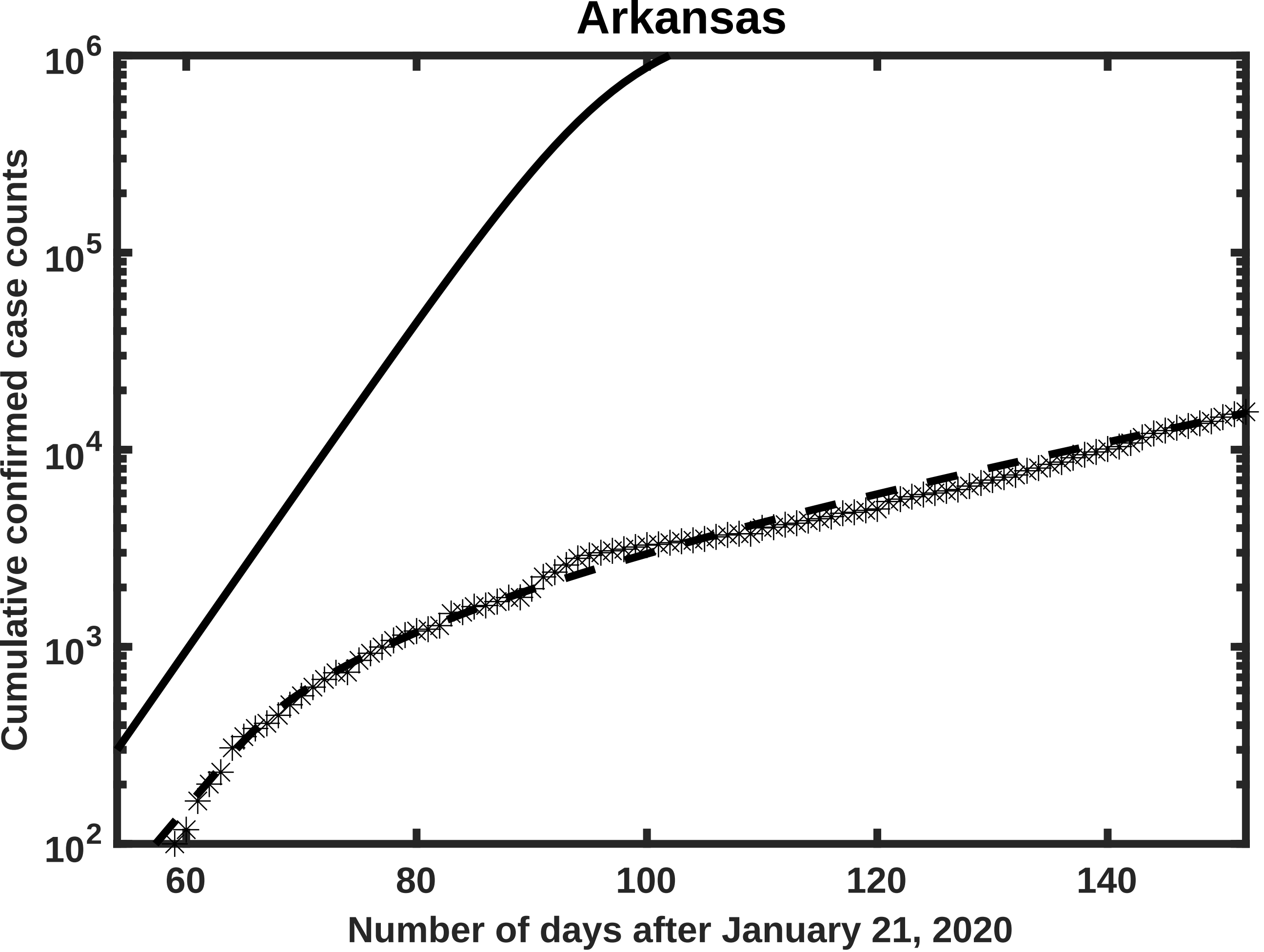

Arkansas
10^6
10^5
10^4
10^3
10^2
60
80
100
120
140
Cumulative confirmed case counts
Number of days after January 21, 2020

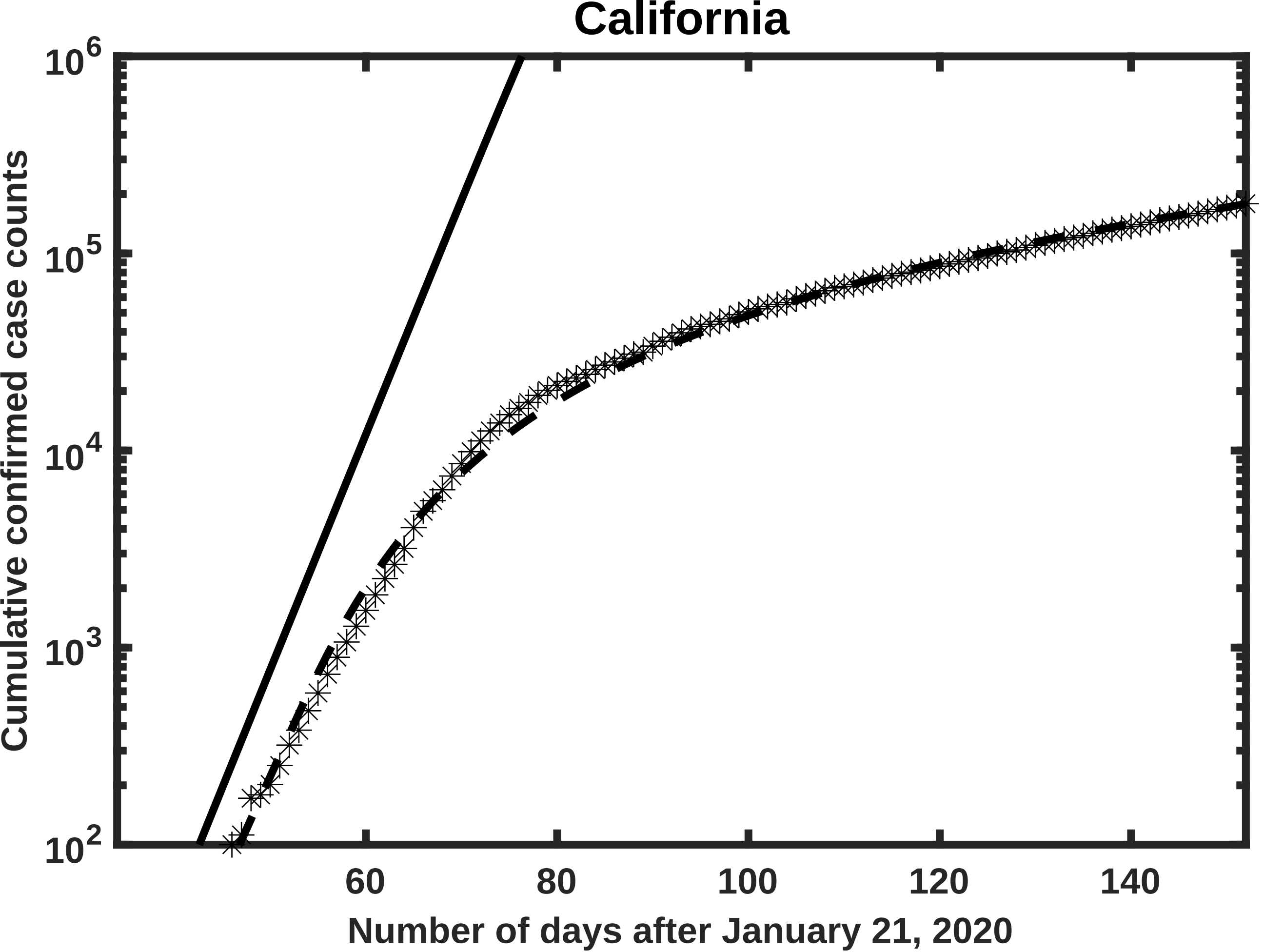
California
Cumulative confirmed case counts
10^6
10^5
10^4
10^3
10^2
60
80
100
120
140
Number of days after January 21, 2020

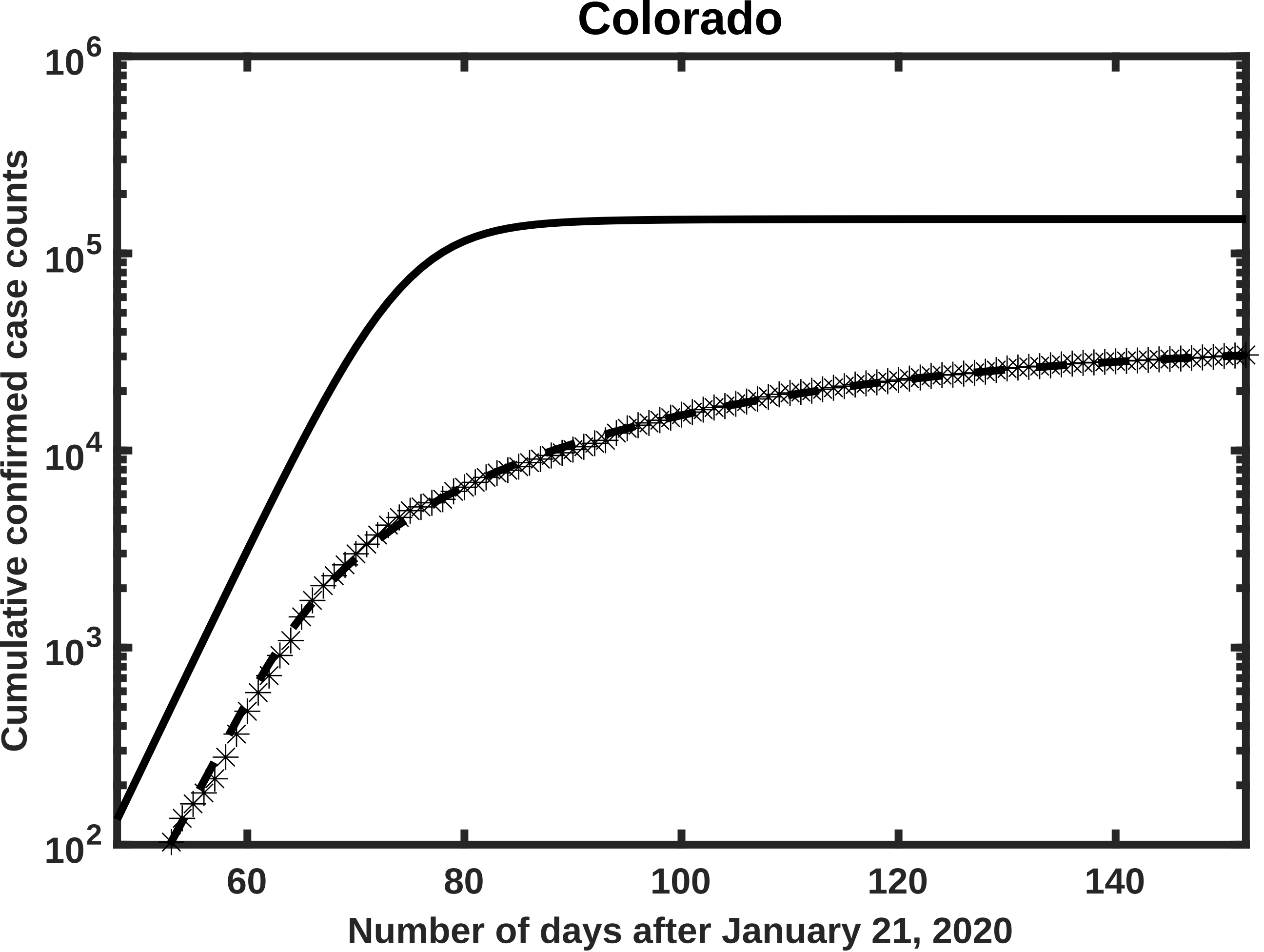
Colorado
Cumulative confirmed case counts
10^6
10^5
10^4
10^3
10^2
60
80
100
120
140
Number of days after January 21, 2020

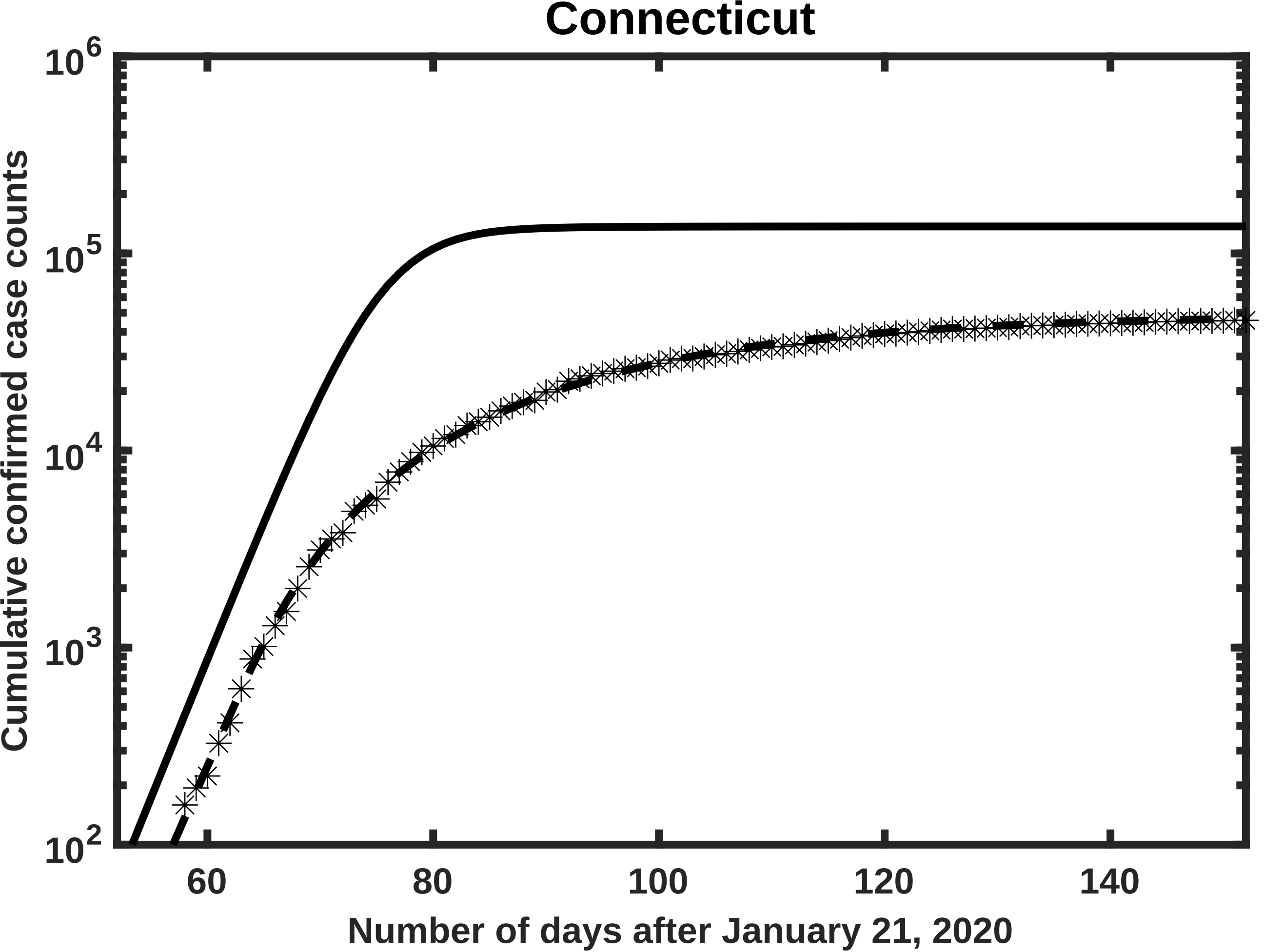
Connecticut
Cumulative confirmed case counts
10^6
10^5
10^4
10^3
10^2
60
80
100
120
140
Number of days after January 21, 2020

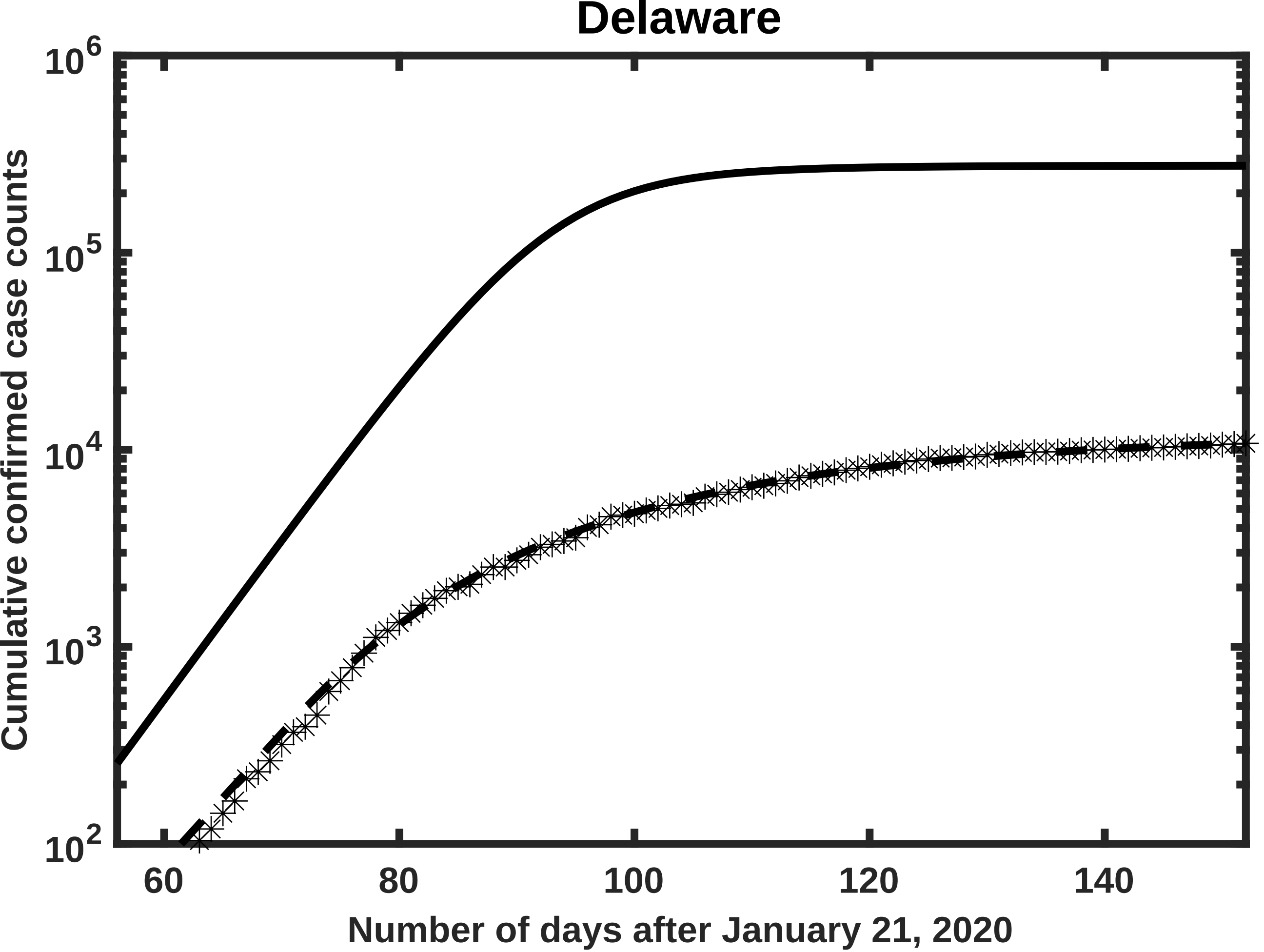

Delaware
10^6
10^5
10^4
10^3
10^2
Cumulative confirmed case counts
60
80
100
120
140
Number of days after January 21, 2020

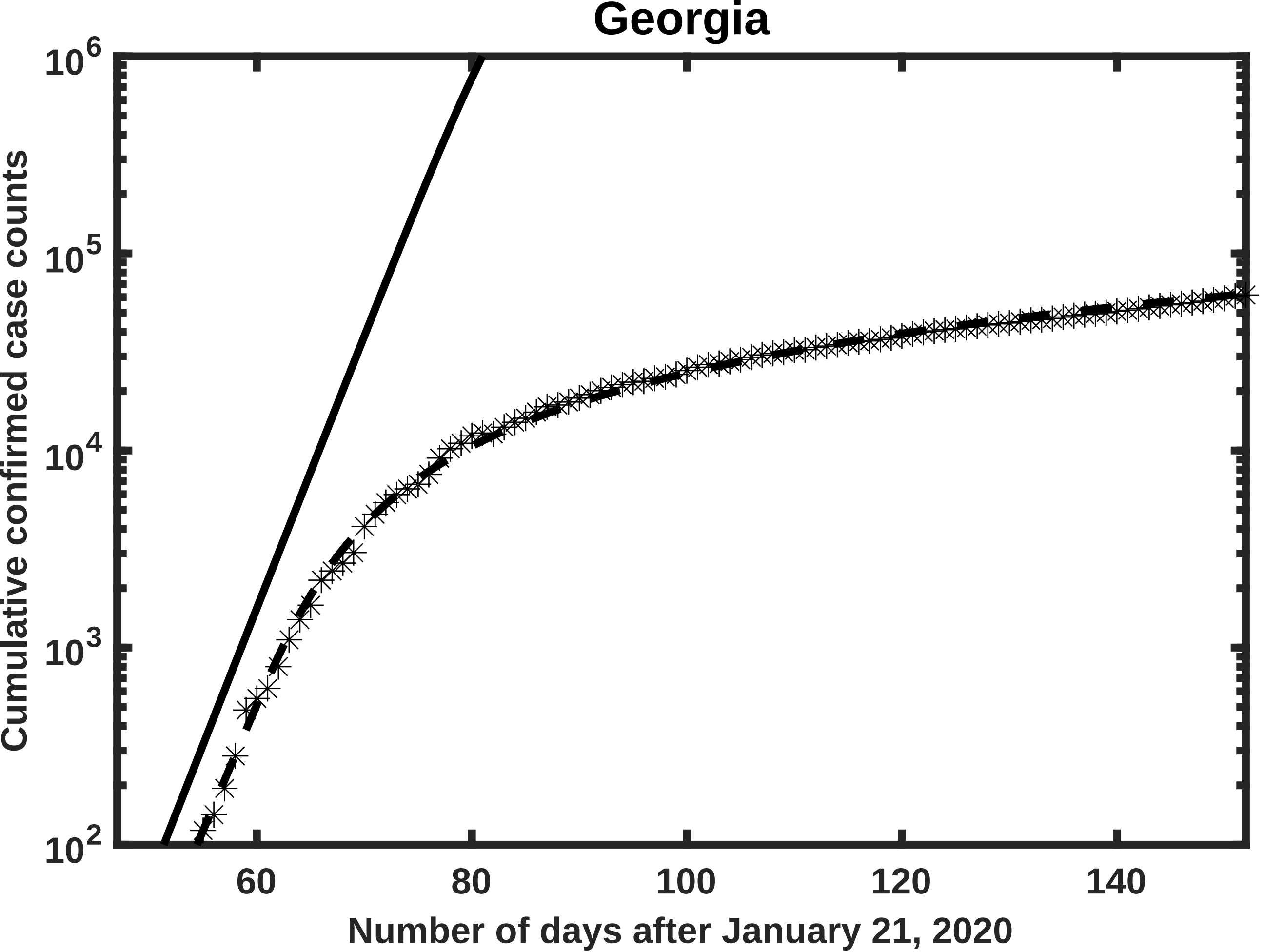
Georgia
Cumulative confirmed case counts
10^6
10^5
10^4
10^3
10^2
60
80
100
120
140
Number of days after January 21, 2020

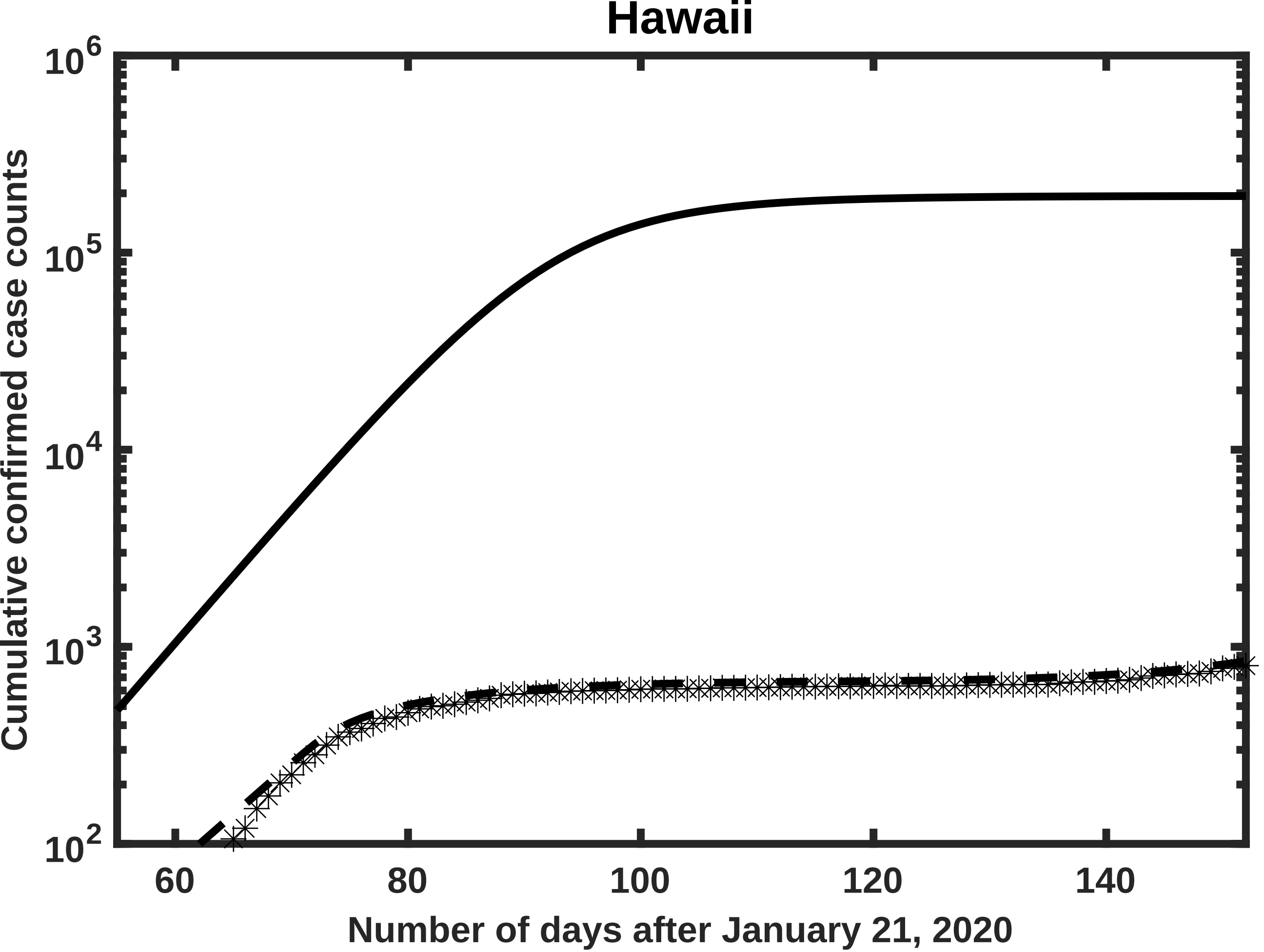

Hawaii
Cumulative confirmed case counts
10^6
10^5
10^4
10^3
10^2
60
80
100
120
140
Number of days after January 21, 2020

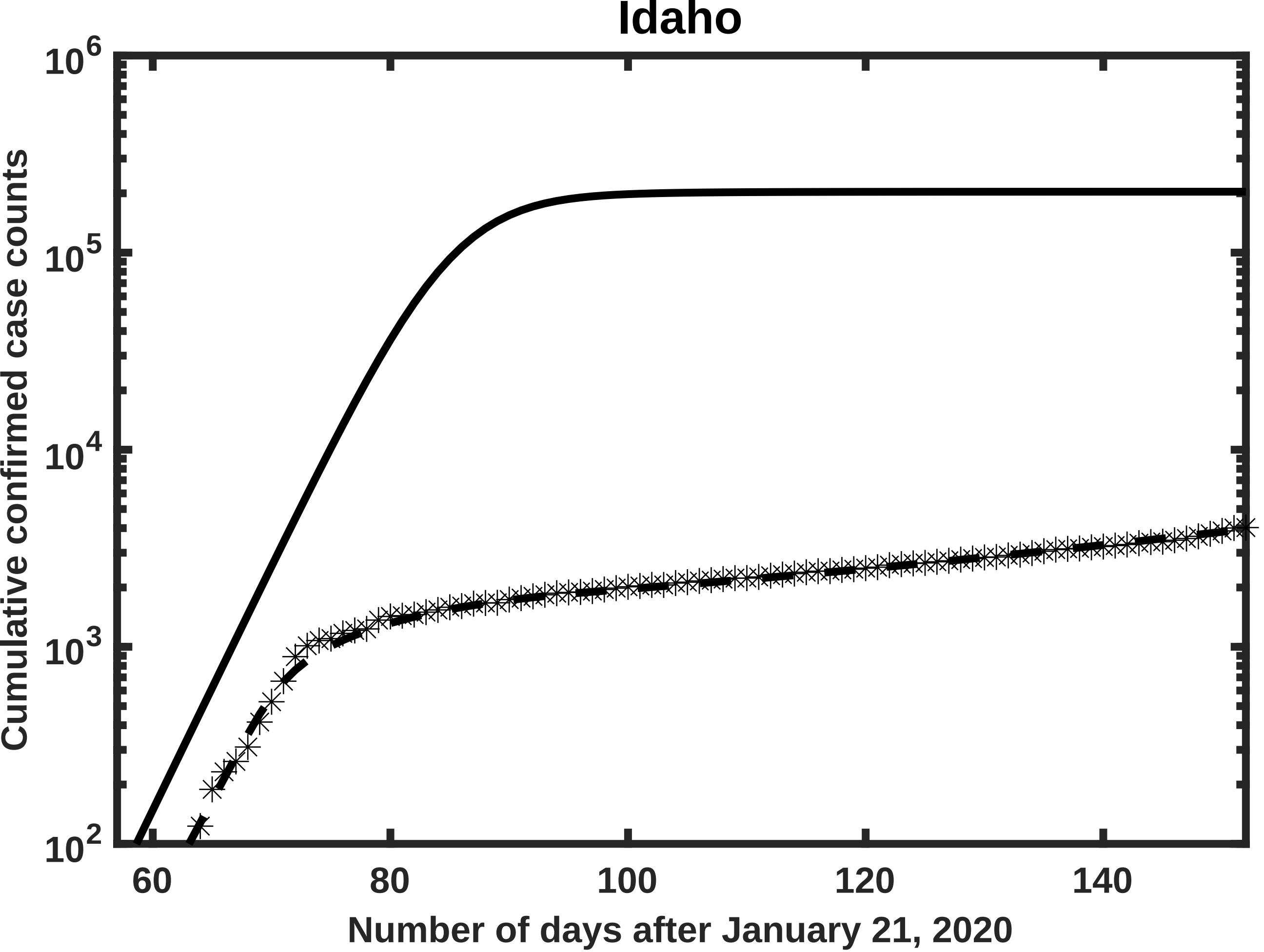
Idaho
Cumulative confirmed case counts
10^6
10^5
10^4
10^3
10^2
60
80
100
120
140
Number of days after January 21, 2020

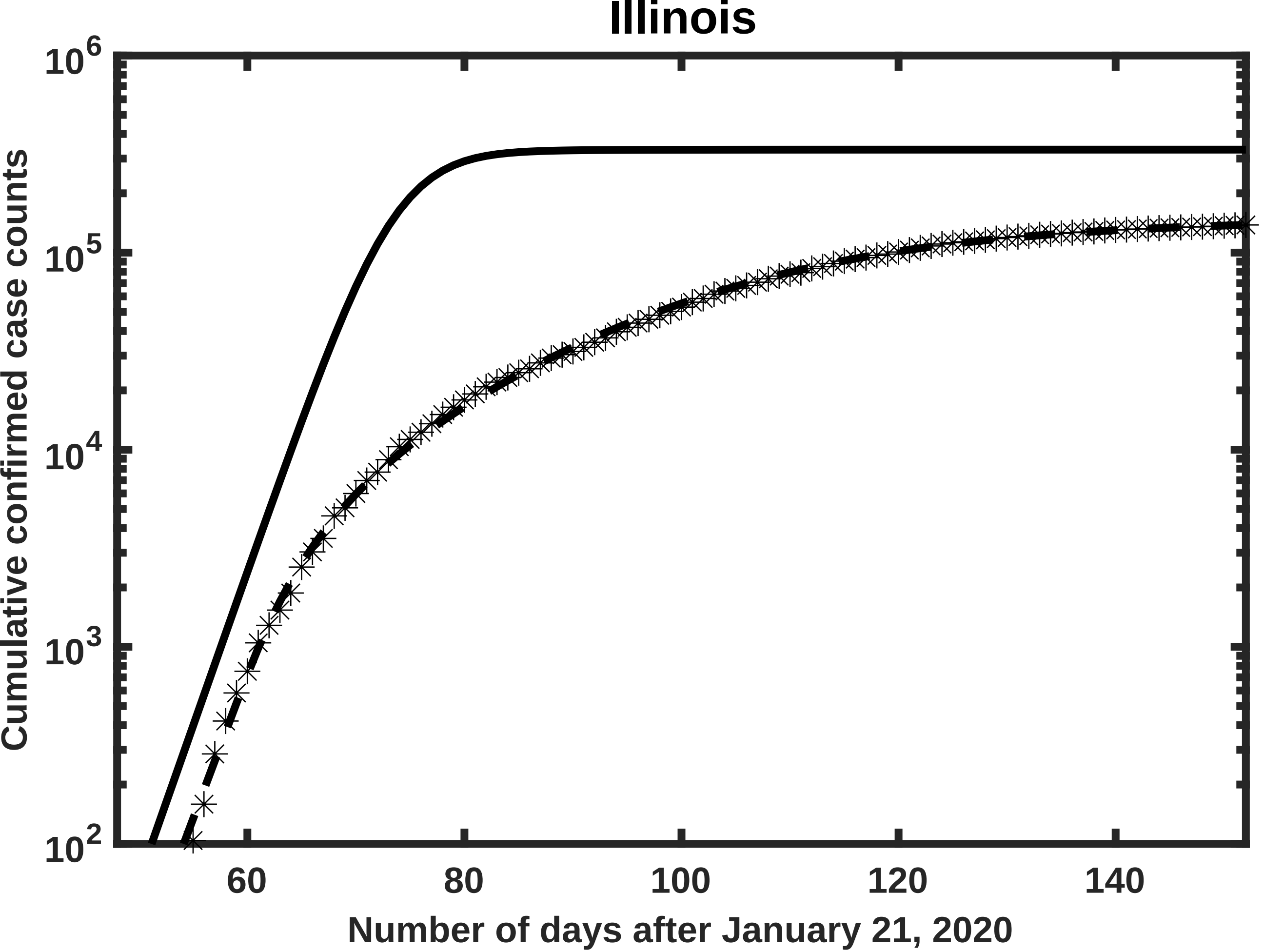
Illinois
10^6
10^5
10^4
10^3
10^2
Cumulative confirmed case counts
60
80
100
120
140
Number of days after January 21, 2020

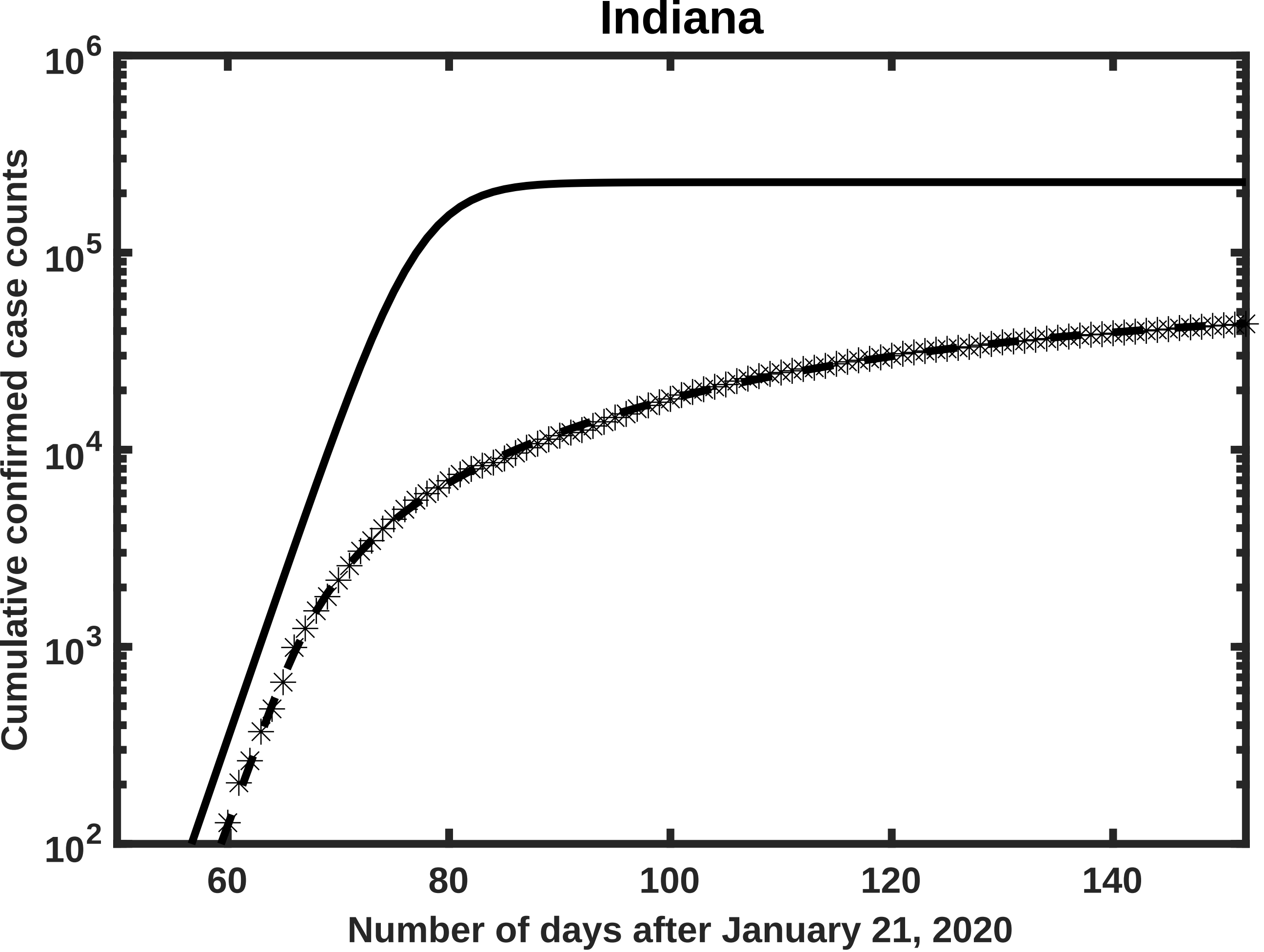
Indiana
10^6
10^5
10^4
10^3
10^2
60
80
100
120
140
Number of days after January 21, 2020
Cumulative confirmed case counts

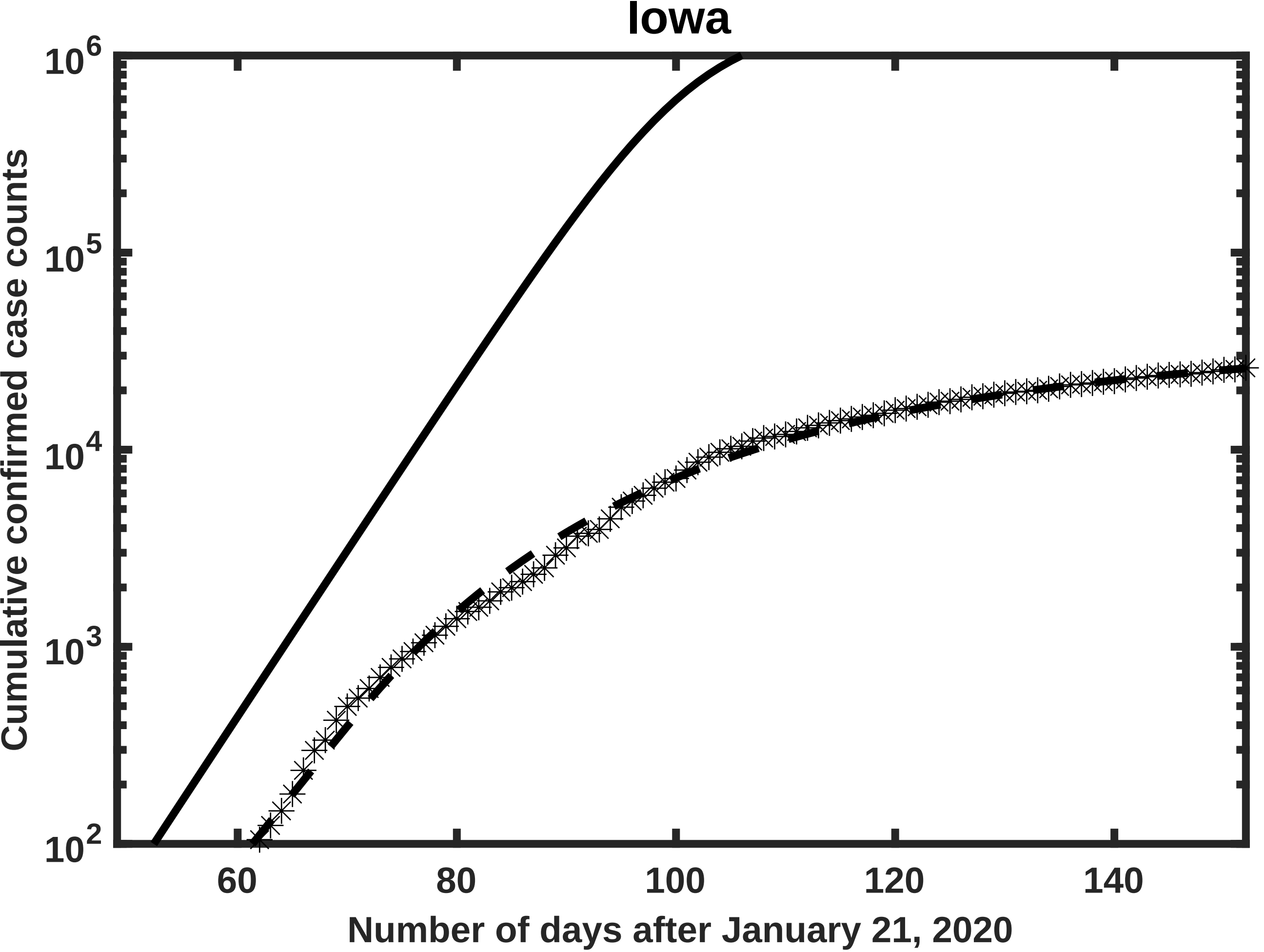
Iowa
Cumulative confirmed case counts
10^6
10^5
10^4
10^3
10^2
60
80
100
120
140
Number of days after January 21, 2020

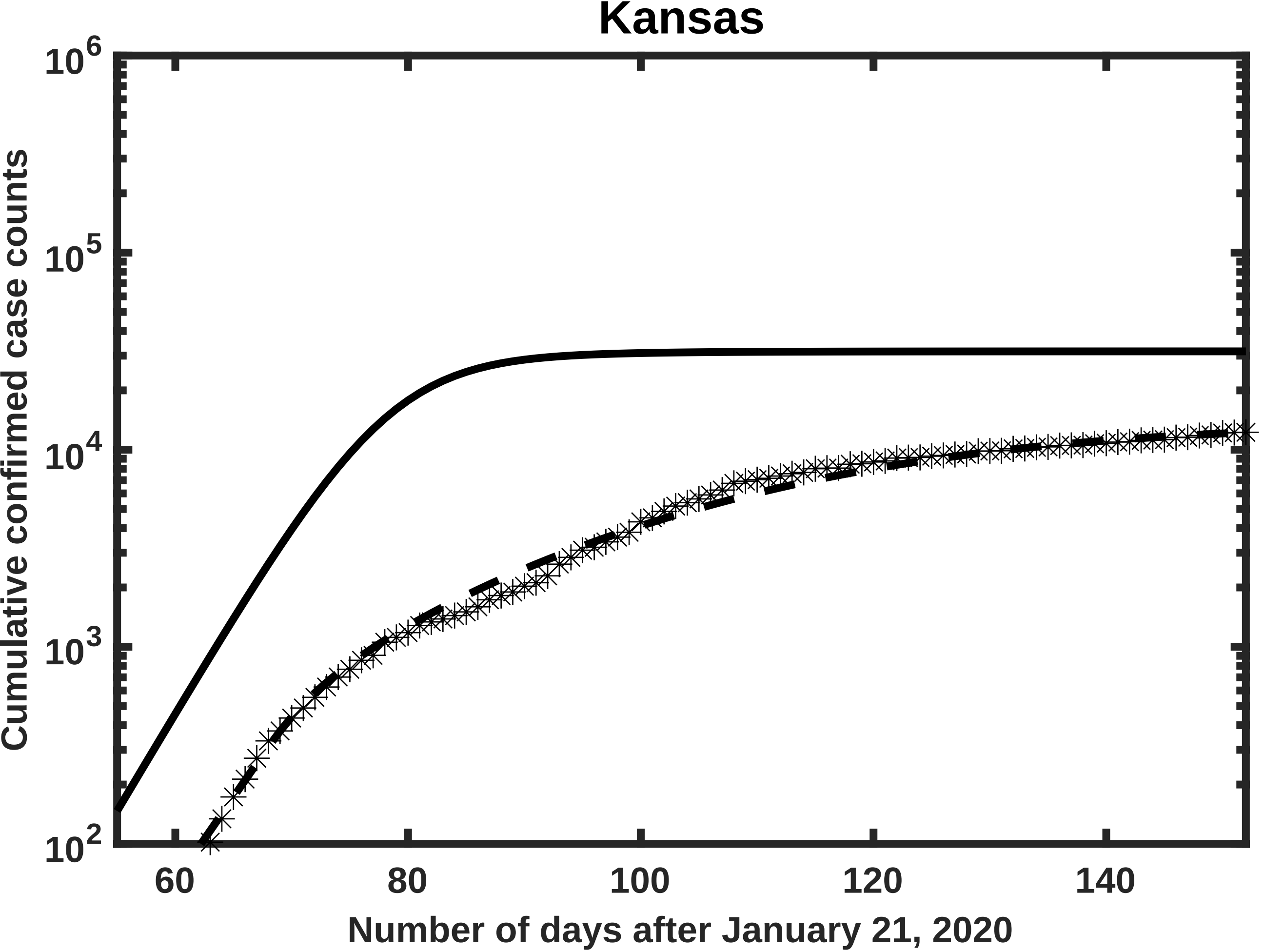
Kansas
10^6
10^5
10^4
10^3
10^2
Cumulative confirmed case counts
60
80
100
120
140
Number of days after January 21, 2020

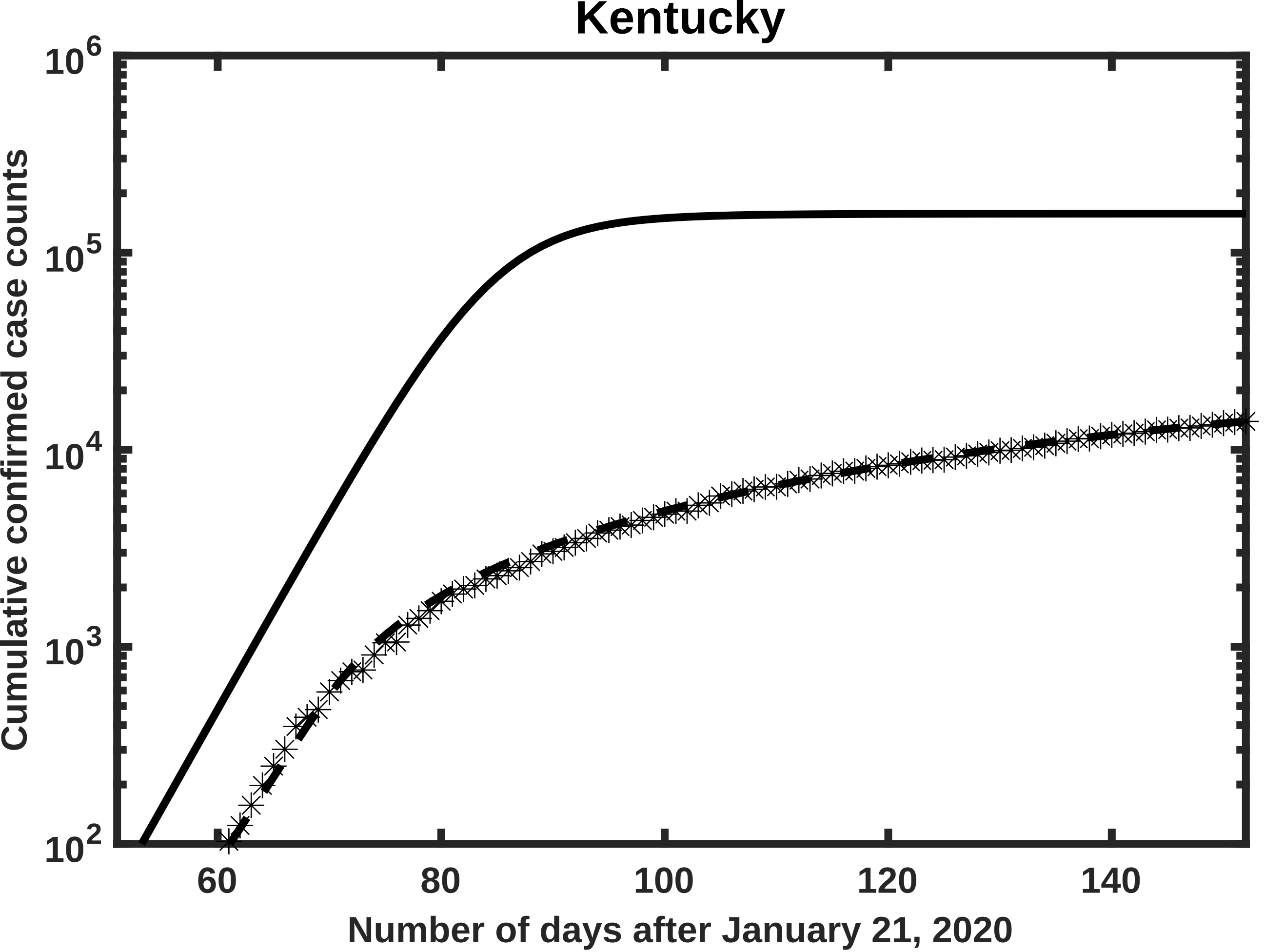
Kentucky
10^6
10^5
10^4
10^3
10^2
Cumulative confirmed case counts
60
80
100
120
140
Number of days after January 21, 2020

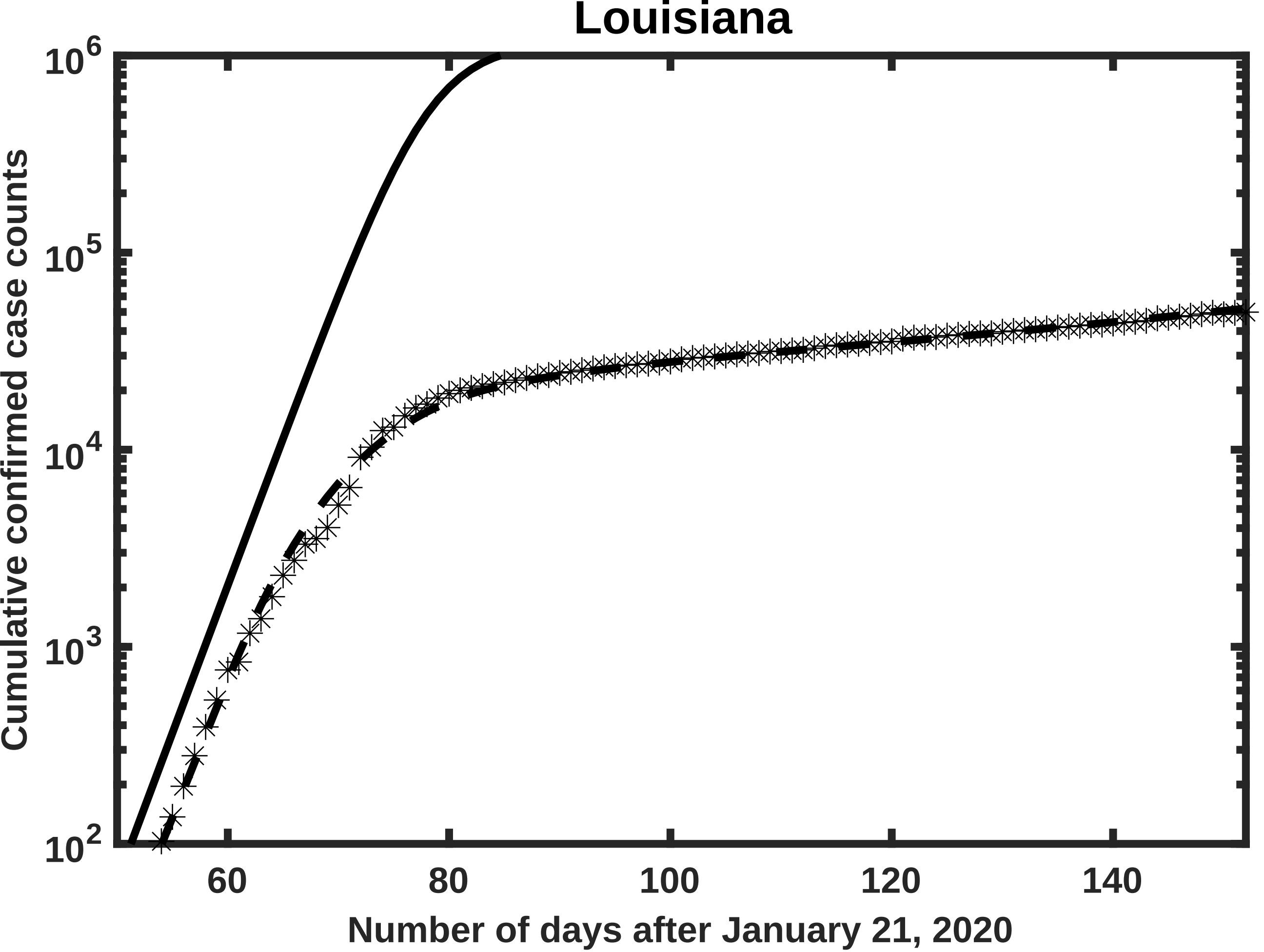
Louisiana
Cumulative confirmed case counts
10^6
10^5
10^4
10^3
10^2
60
80
100
120
140
Number of days after January 21, 2020

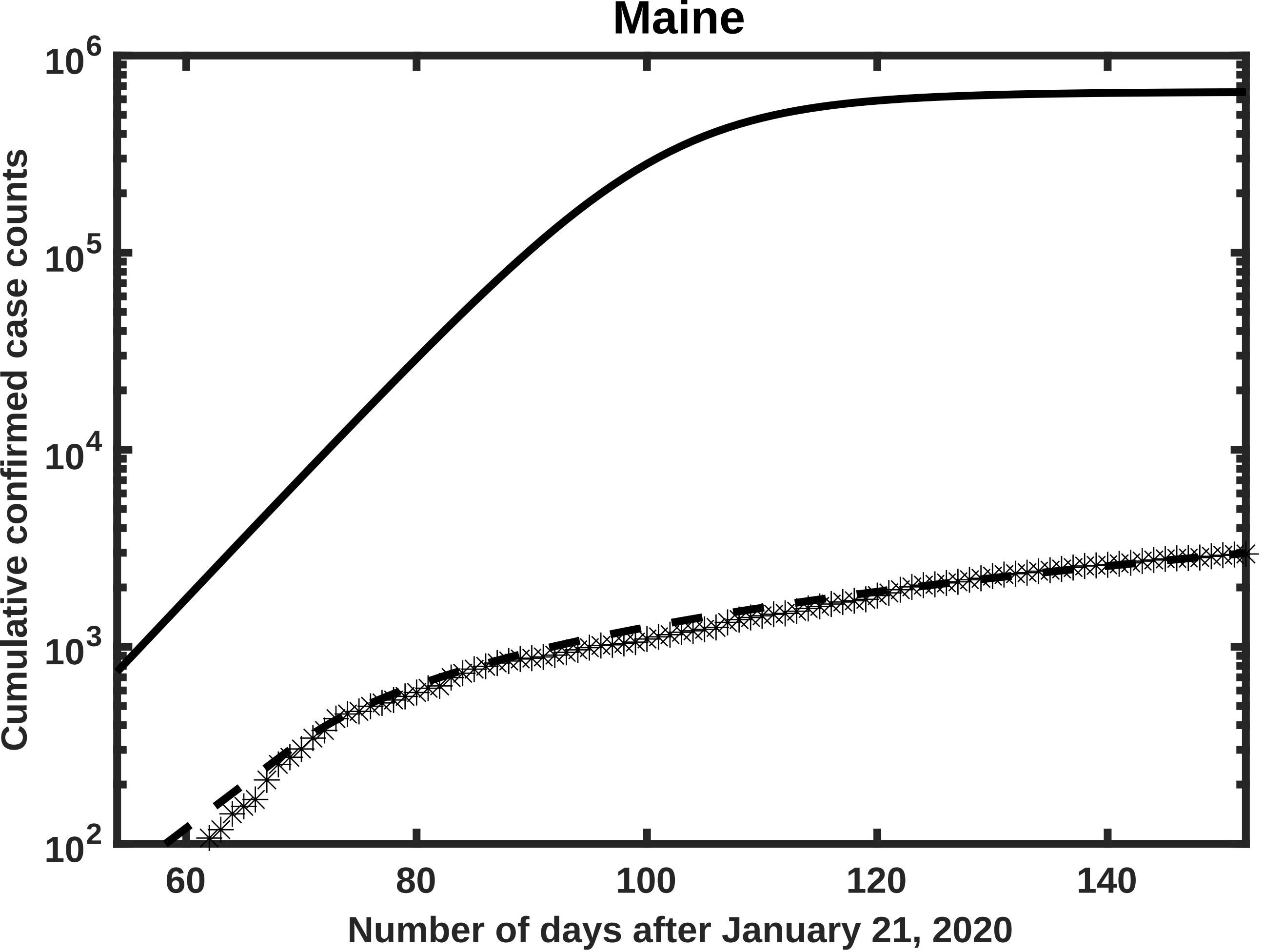

Maine
10^6
10^5
10^4
10^3
10^2
Cumulative confirmed case counts
60
80
100
120
140
Number of days after January 21, 2020

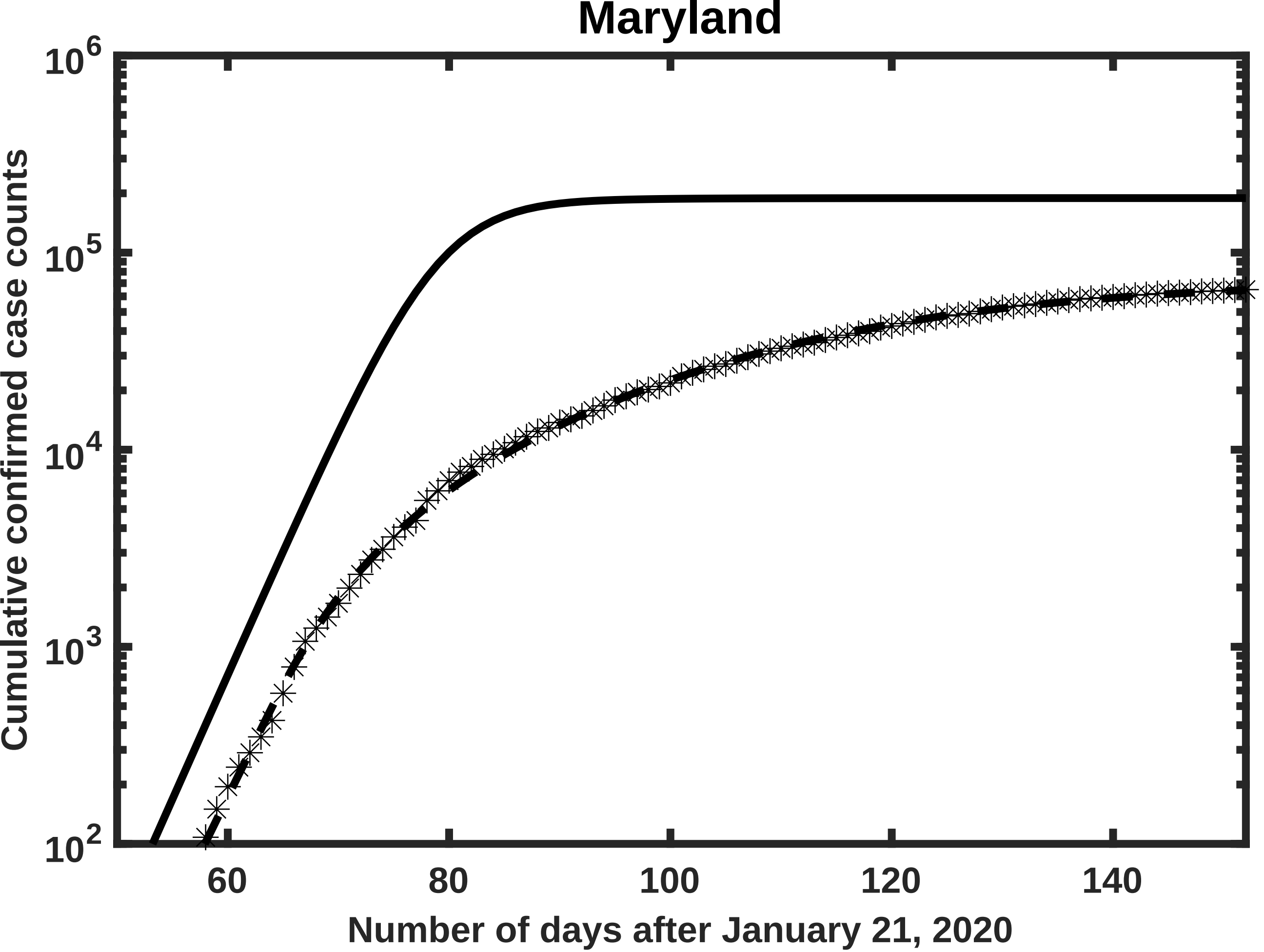
Maryland
Cumulative confirmed case counts
10^6
10^5
10^4
10^3
10^2
60
80
100
120
140
Number of days after January 21, 2020

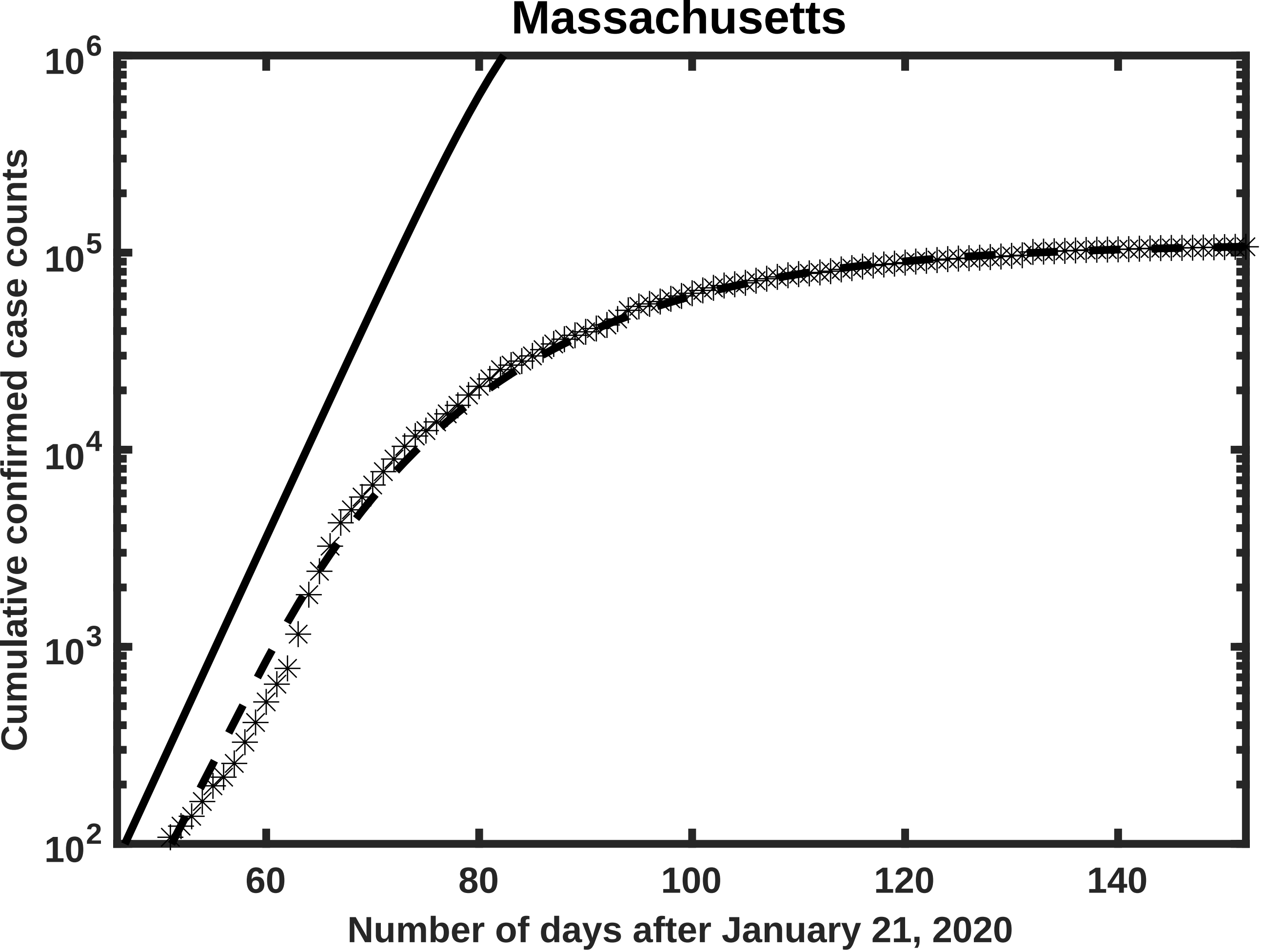

Massachusetts
Cumulative confirmed case counts
10^2
10^3
10^4
10^5
10^6
60
80
100
120
140
Number of days after January 21, 2020

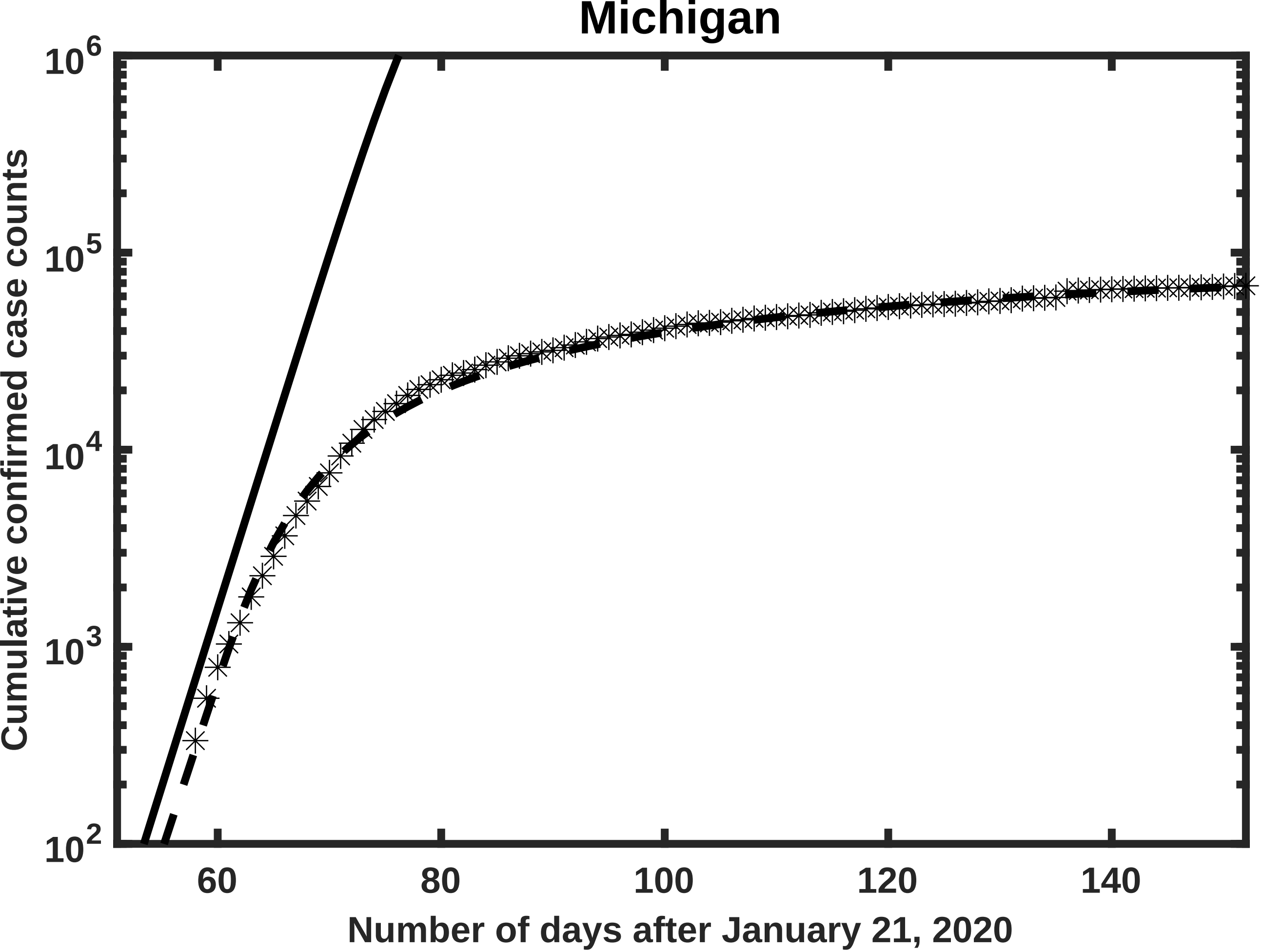

Michigan
Cumulative confirmed case counts
10^6
10^5
10^4
10^3
10^2
60
80
100
120
140
Number of days after January 21, 2020

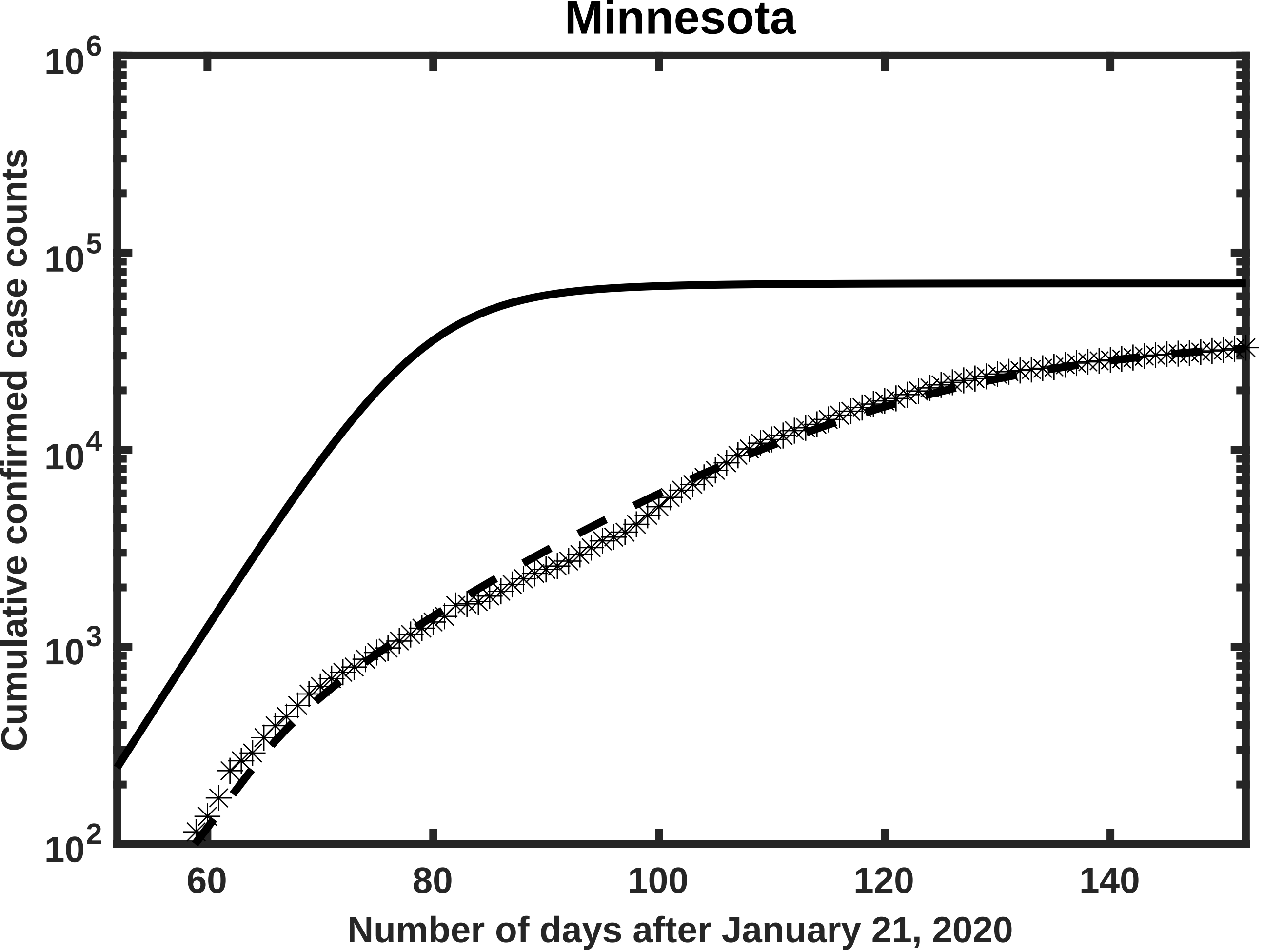

Minnesota
Cumulative confirmed case counts
Number of days after January 21, 2020
10^6
10^5
10^4
10^3
10^2
60
80
100
120
140

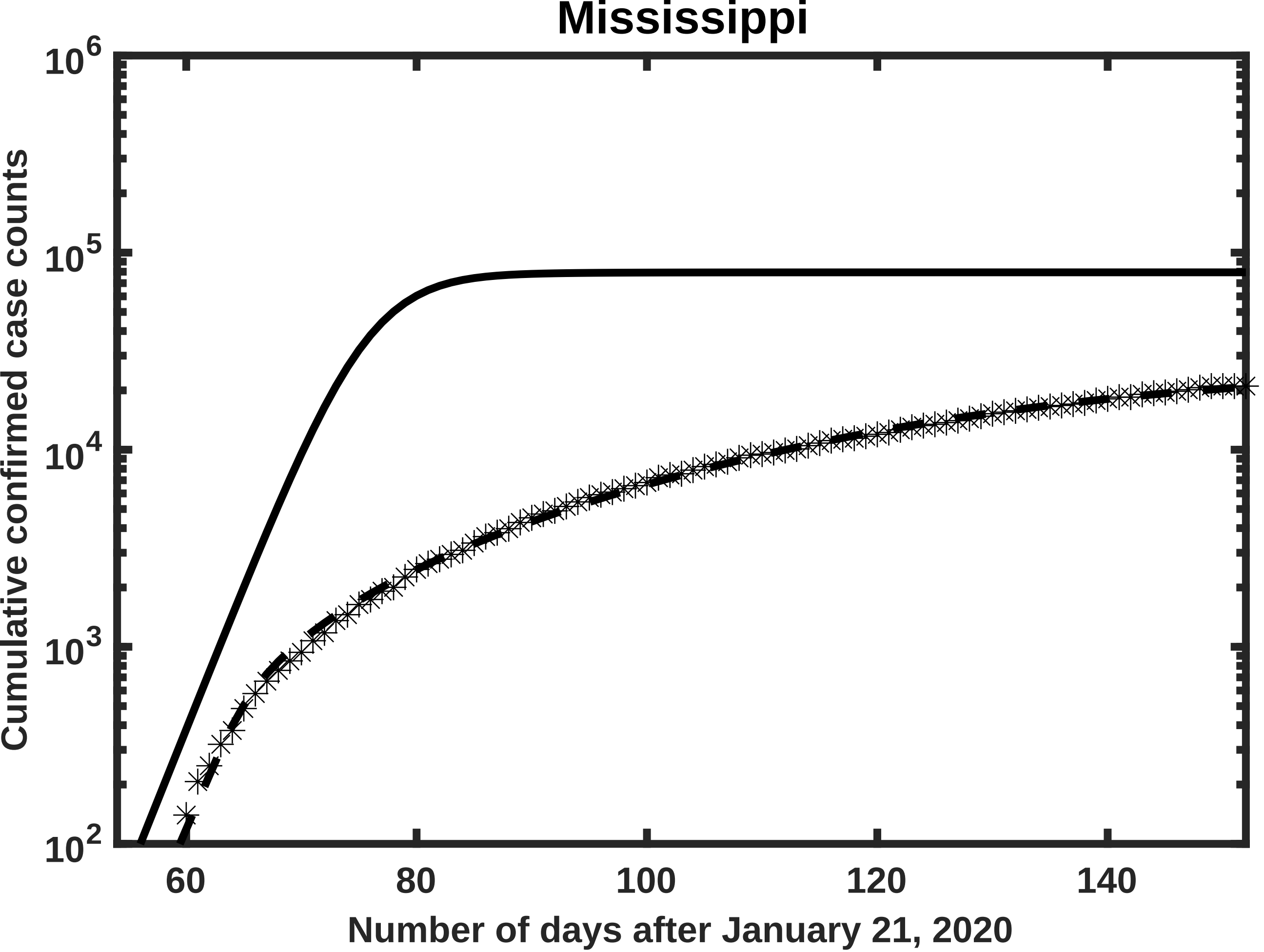
Mississippi
10^6
10^5
10^4
10^3
10^2
Cumulative confirmed case counts
60
80
100
120
140
Number of days after January 21, 2020

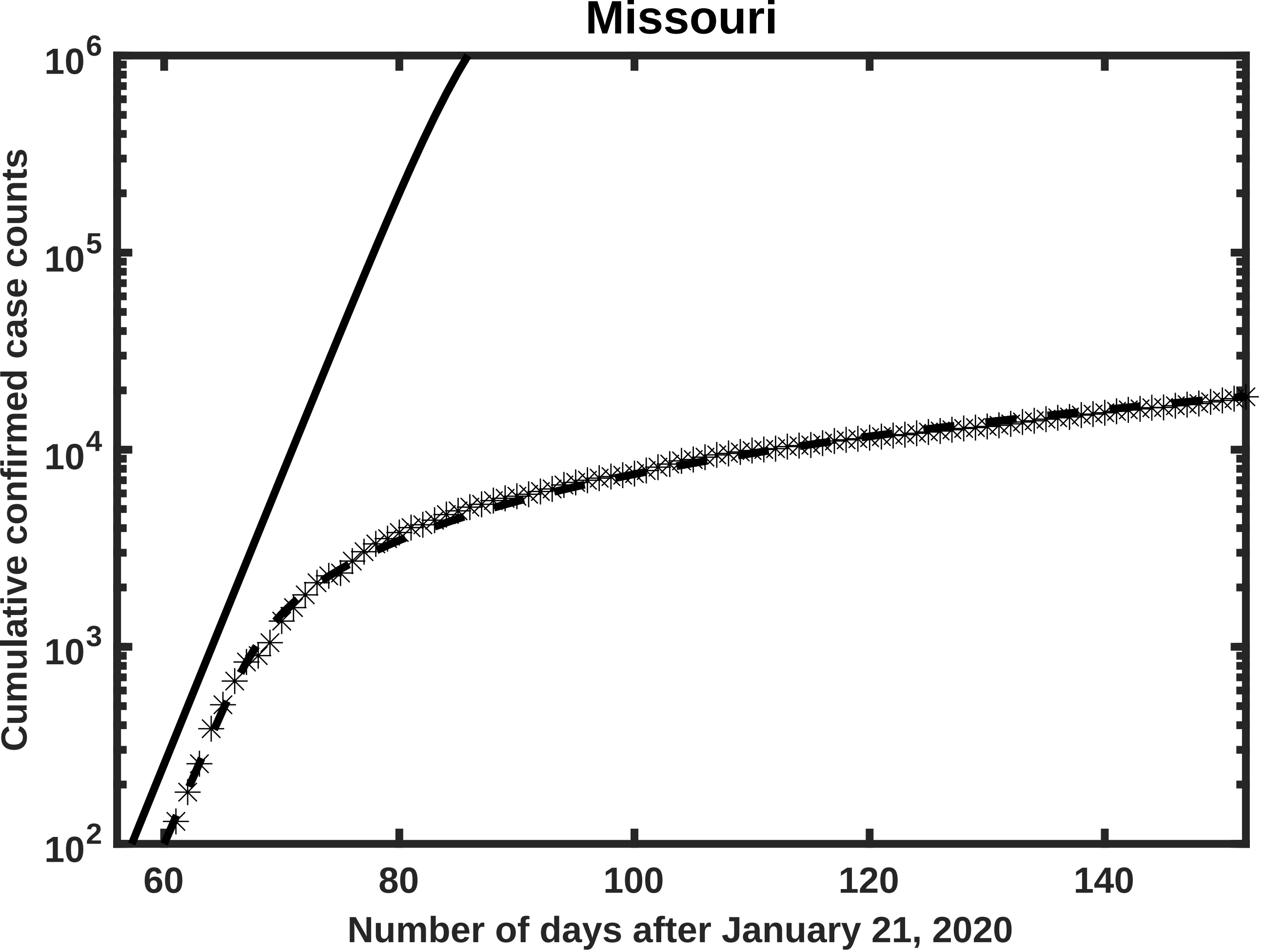
Missouri
10^6
10^5
10^4
10^3
10^2
Cumulative confirmed case counts
60
80
100
120
140
Number of days after January 21, 2020

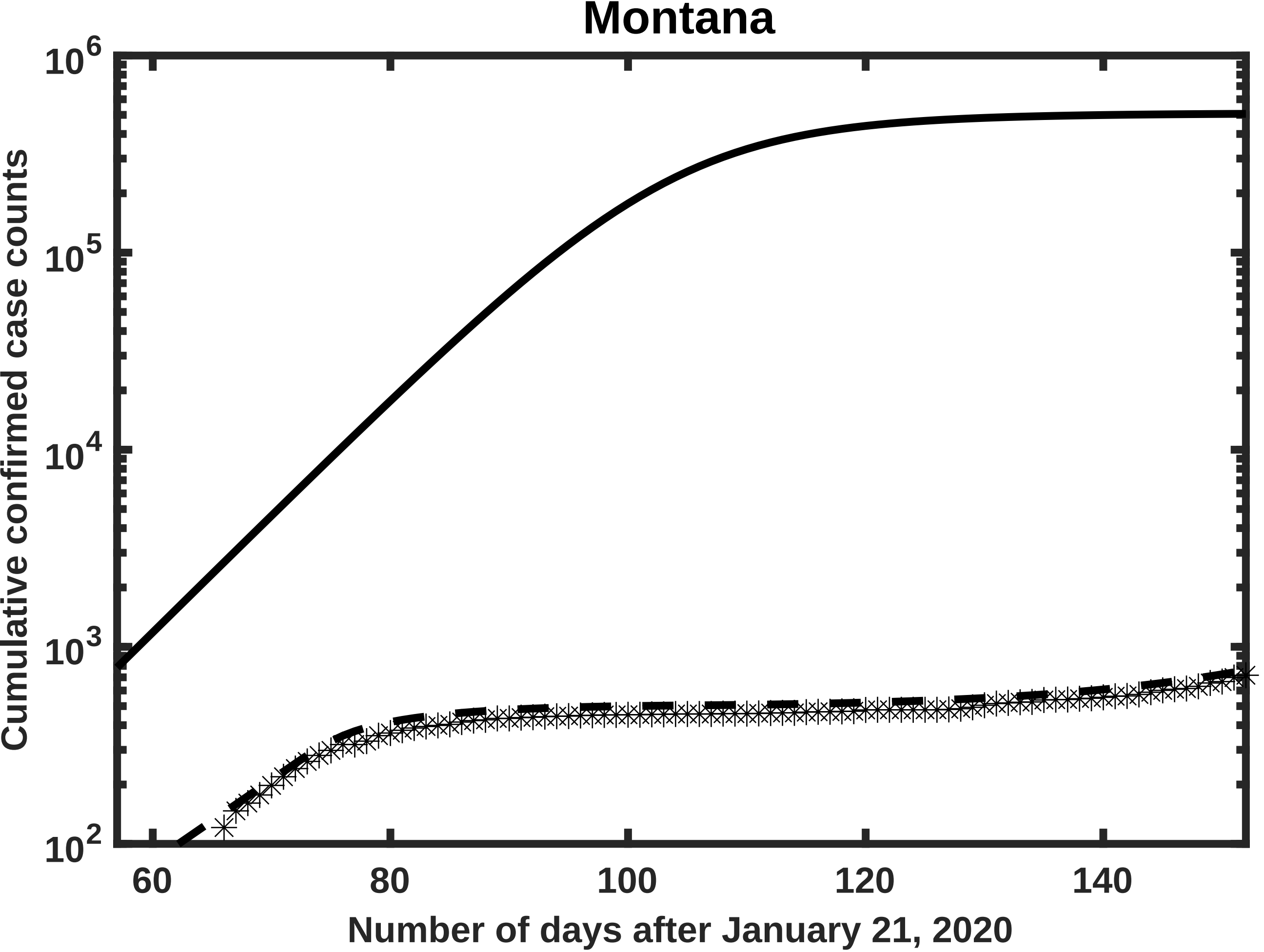
Montana
Cumulative confirmed case counts
10^6
10^5
10^4
10^3
10^2
60
80
100
120
140
Number of days after January 21, 2020

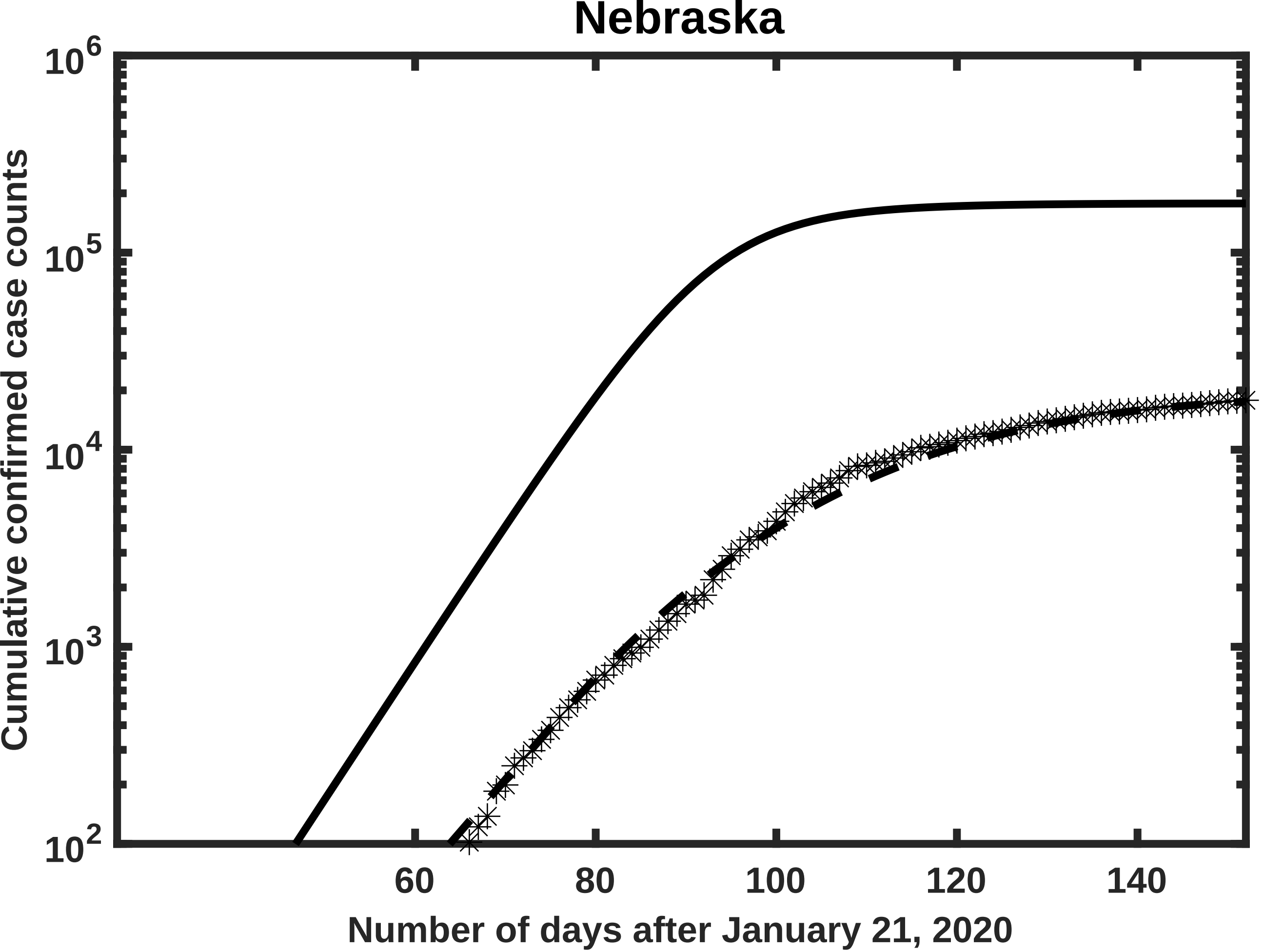

Nebraska
10^6
10^5
10^4
10^3
10^2
60
80
100
120
140
Cumulative confirmed case counts
Number of days after January 21, 2020

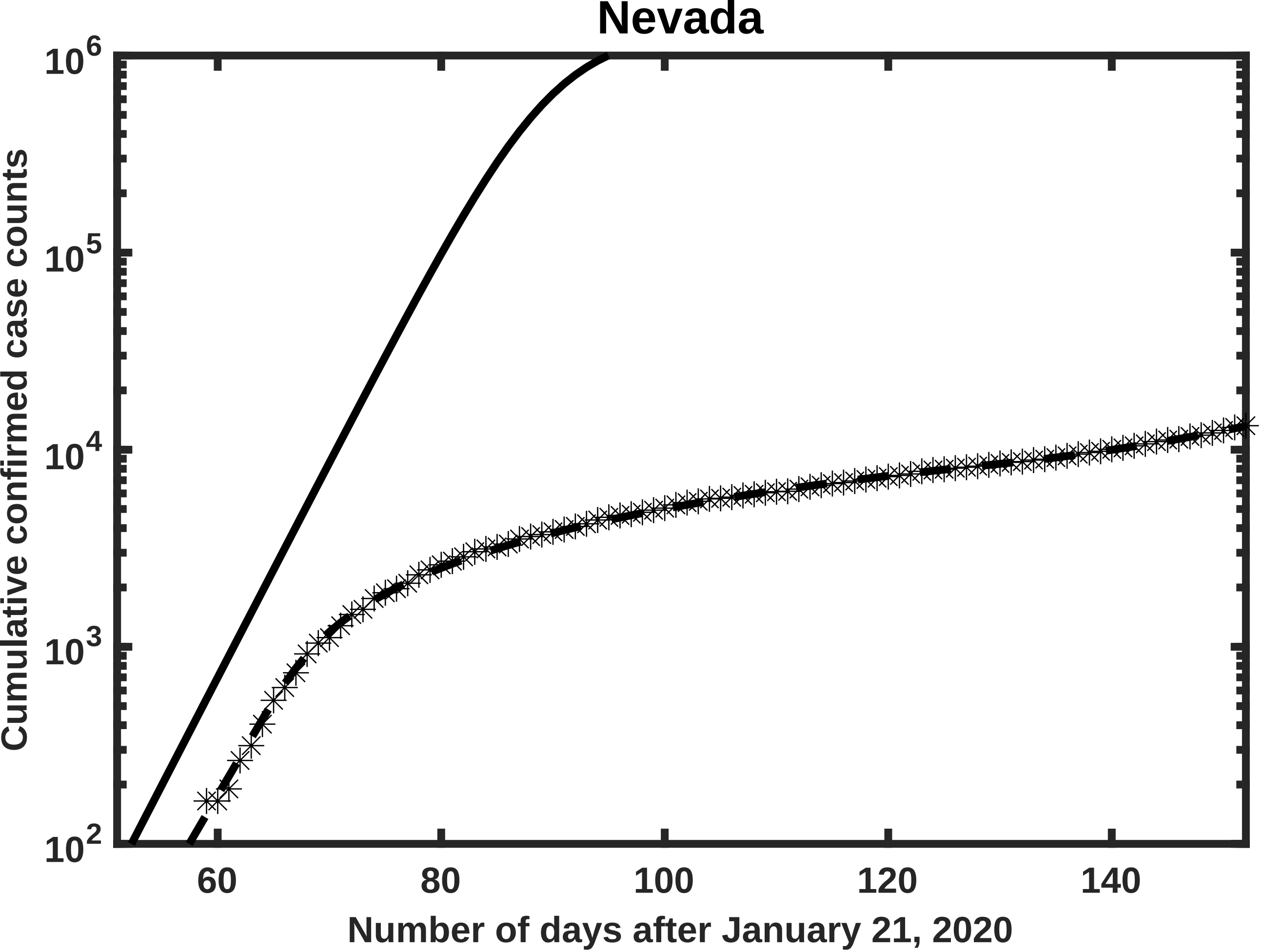

Nevada
10^6
10^5
10^4
10^3
10^2
Cumulative confirmed case counts
60
80
100
120
140
Number of days after January 21, 2020

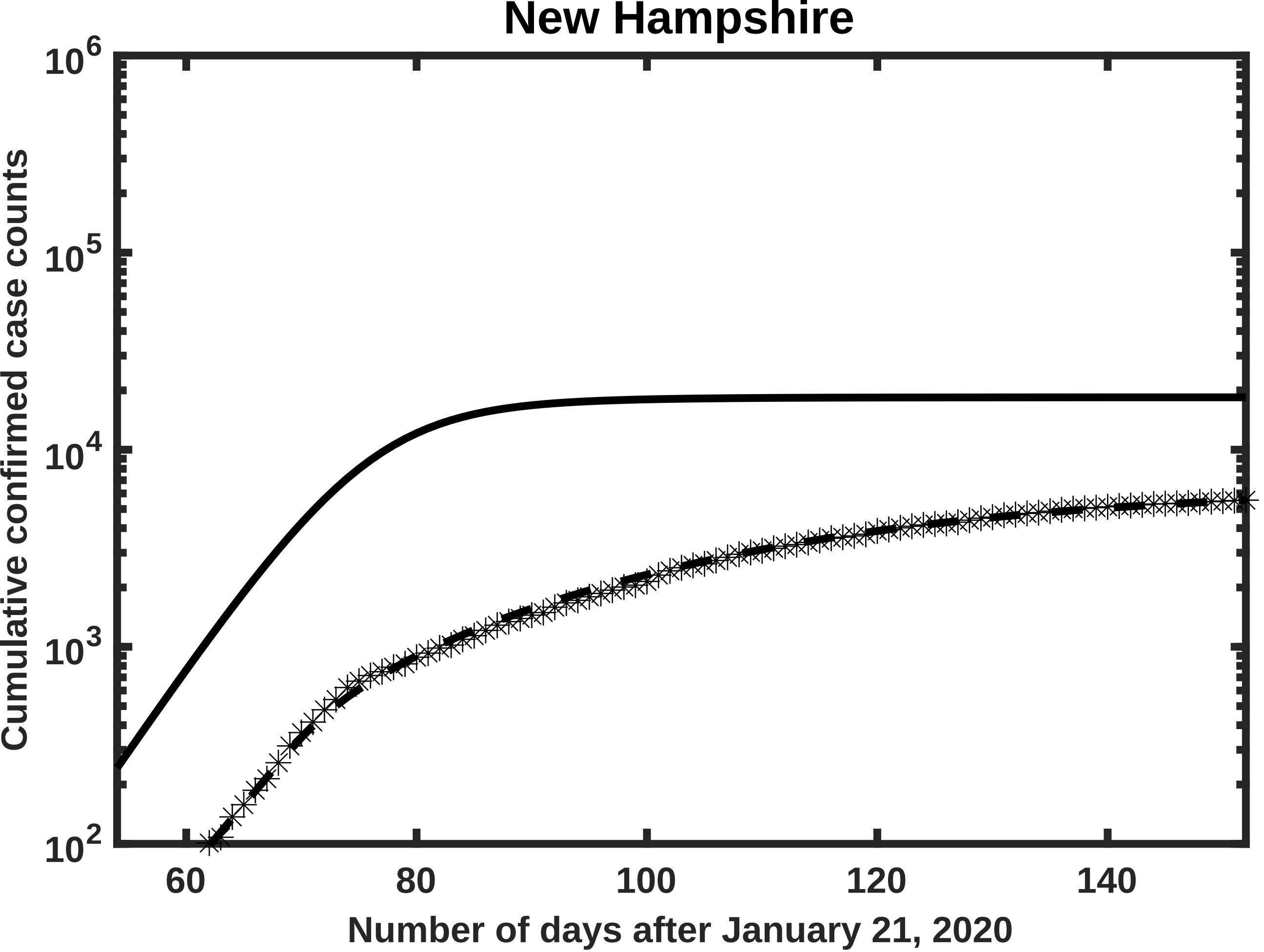

New Hampshire
Cumulative confirmed case counts
10^6
10^5
10^4
10^3
10^2
60
80
100
120
140
Number of days after January 21, 2020

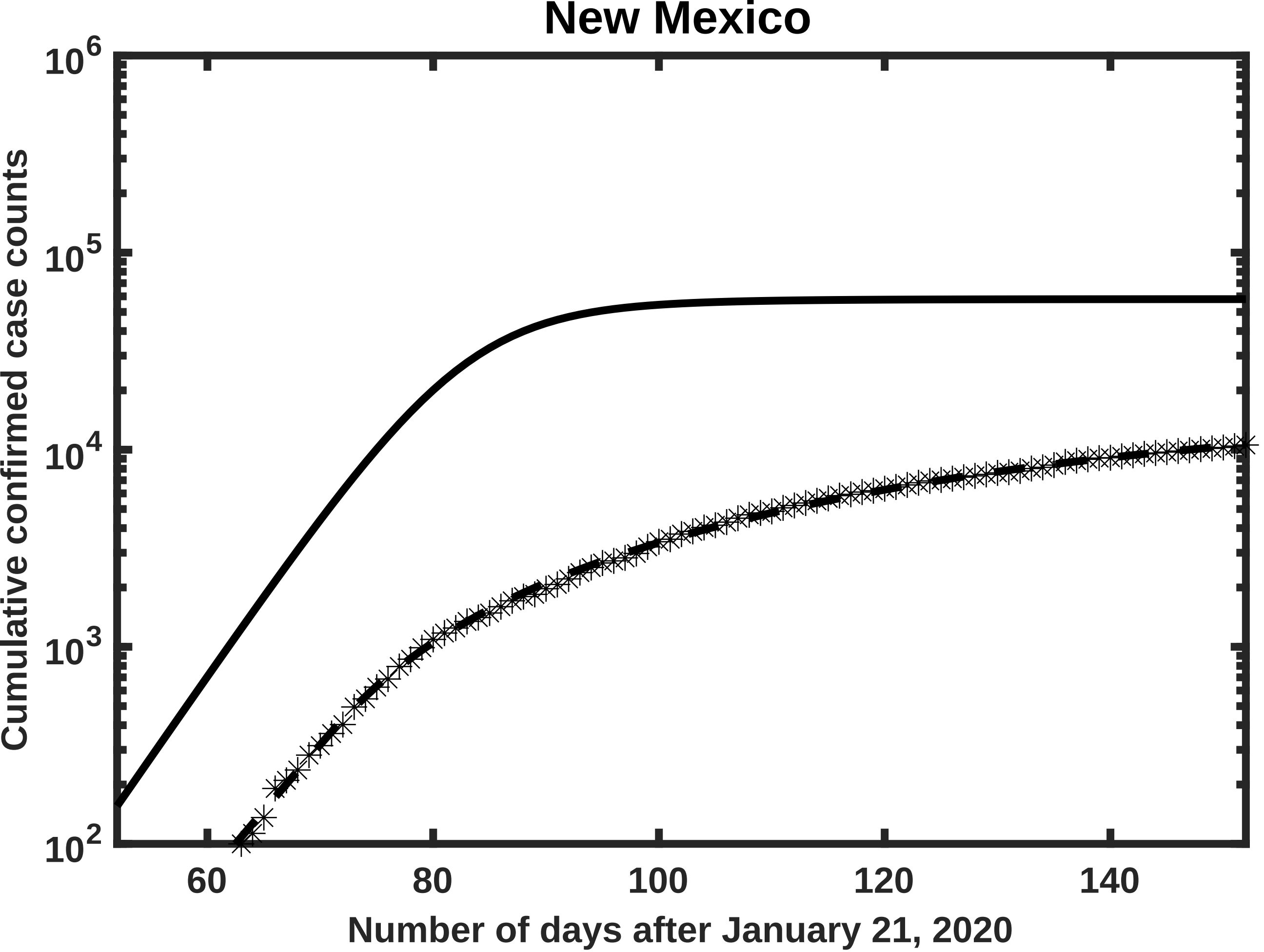
New Mexico
Cumulative confirmed case counts
10^2
10^3
10^4
10^5
10^6
60
80
100
120
140
Number of days after January 21, 2020

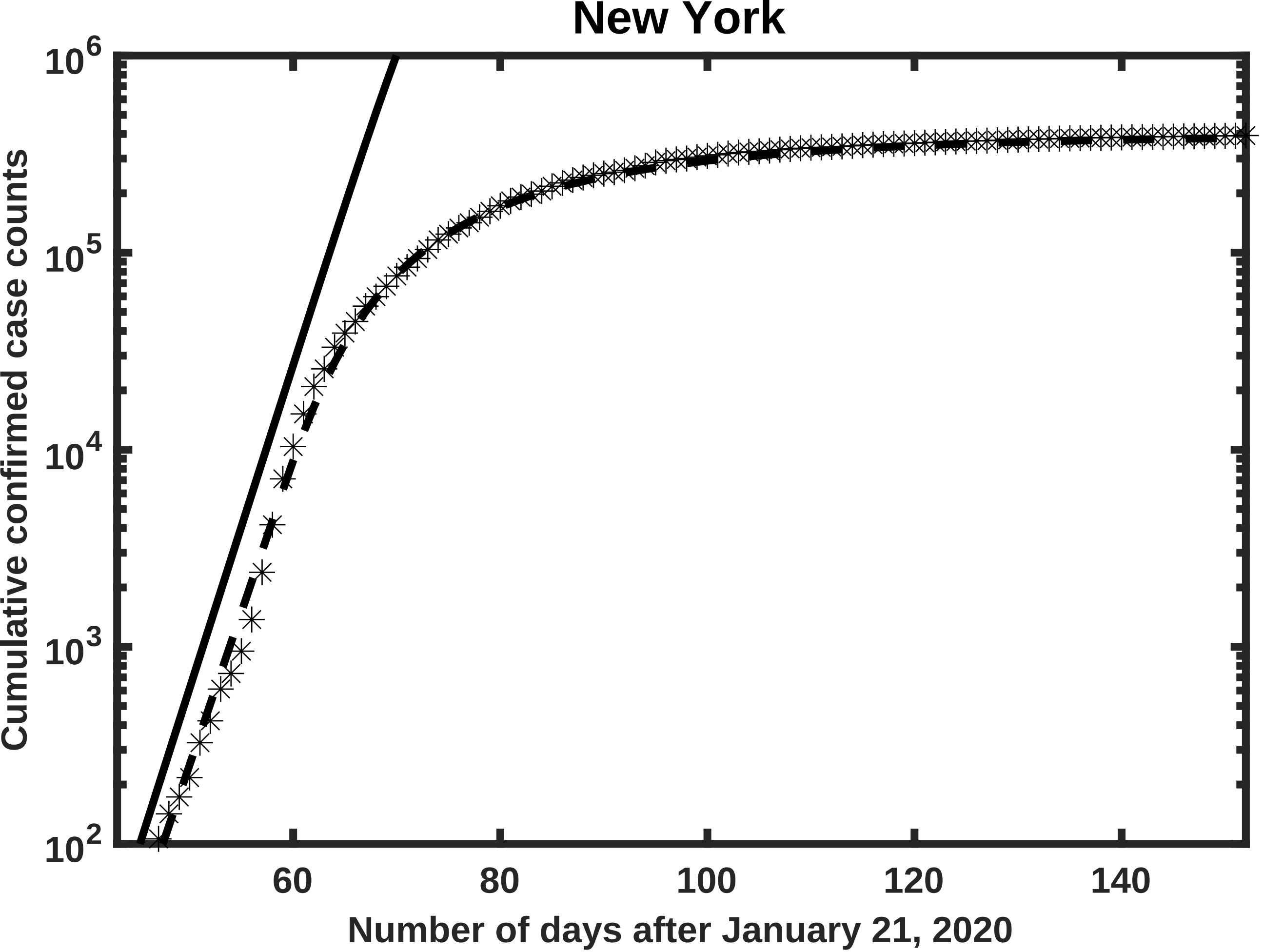
New York
Cumulative confirmed case counts
10^2
10^3
10^4
10^5
10^6
60
80
100
120
140
Number of days after January 21, 2020

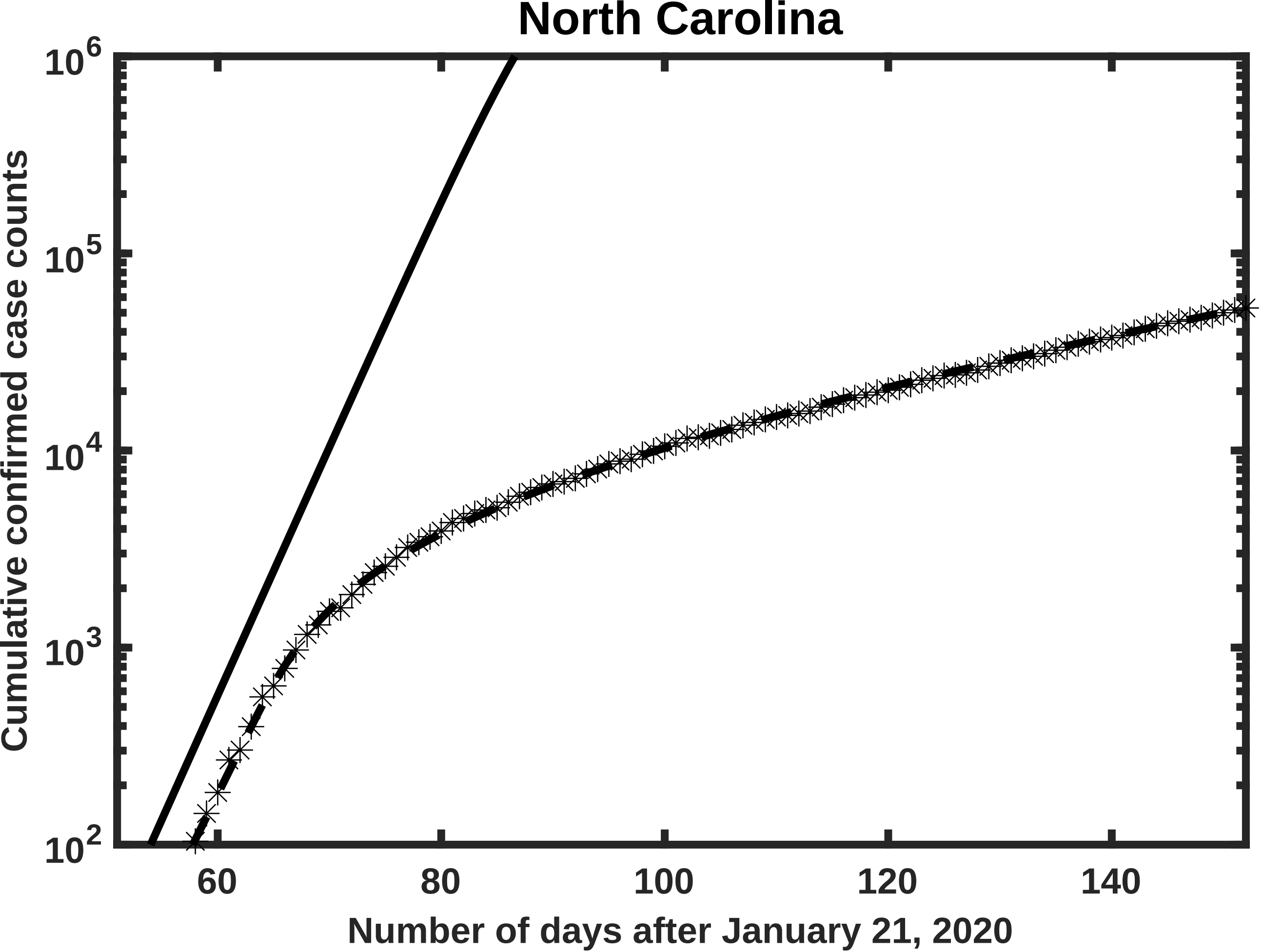

North Carolina
Cumulative confirmed case counts
10^6
10^5
10^4
10^3
10^2
60
80
100
120
140
Number of days after January 21, 2020

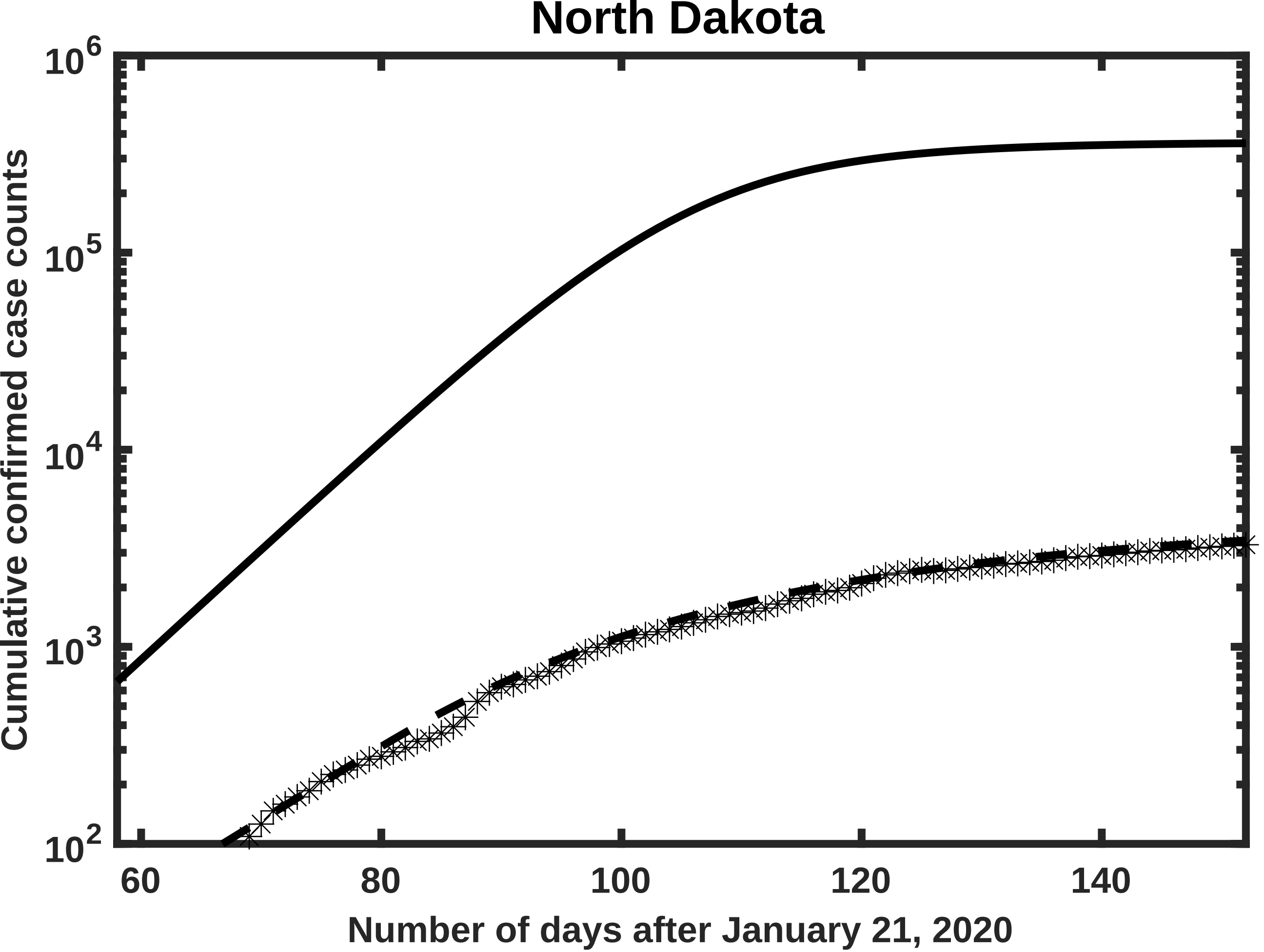

North Dakota
Cumulative confirmed case counts
10^6
10^5
10^4
10^3
10^2
60
80
100
120
140
Number of days after January 21, 2020

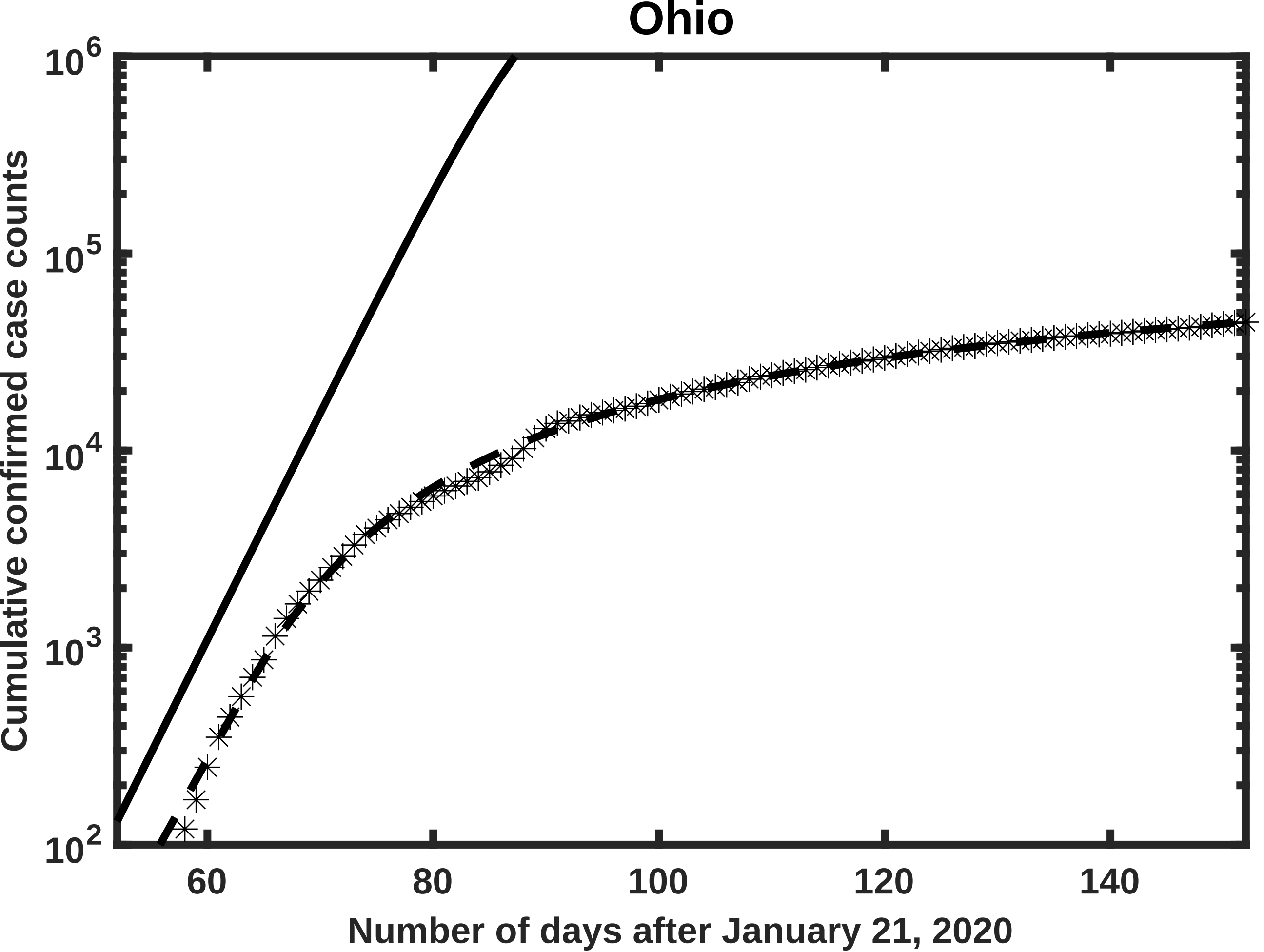
Ohio
Cumulative confirmed case counts
Number of days after January 21, 2020
10^2
10^3
10^4
10^5
10^6
60
80
100
120
140

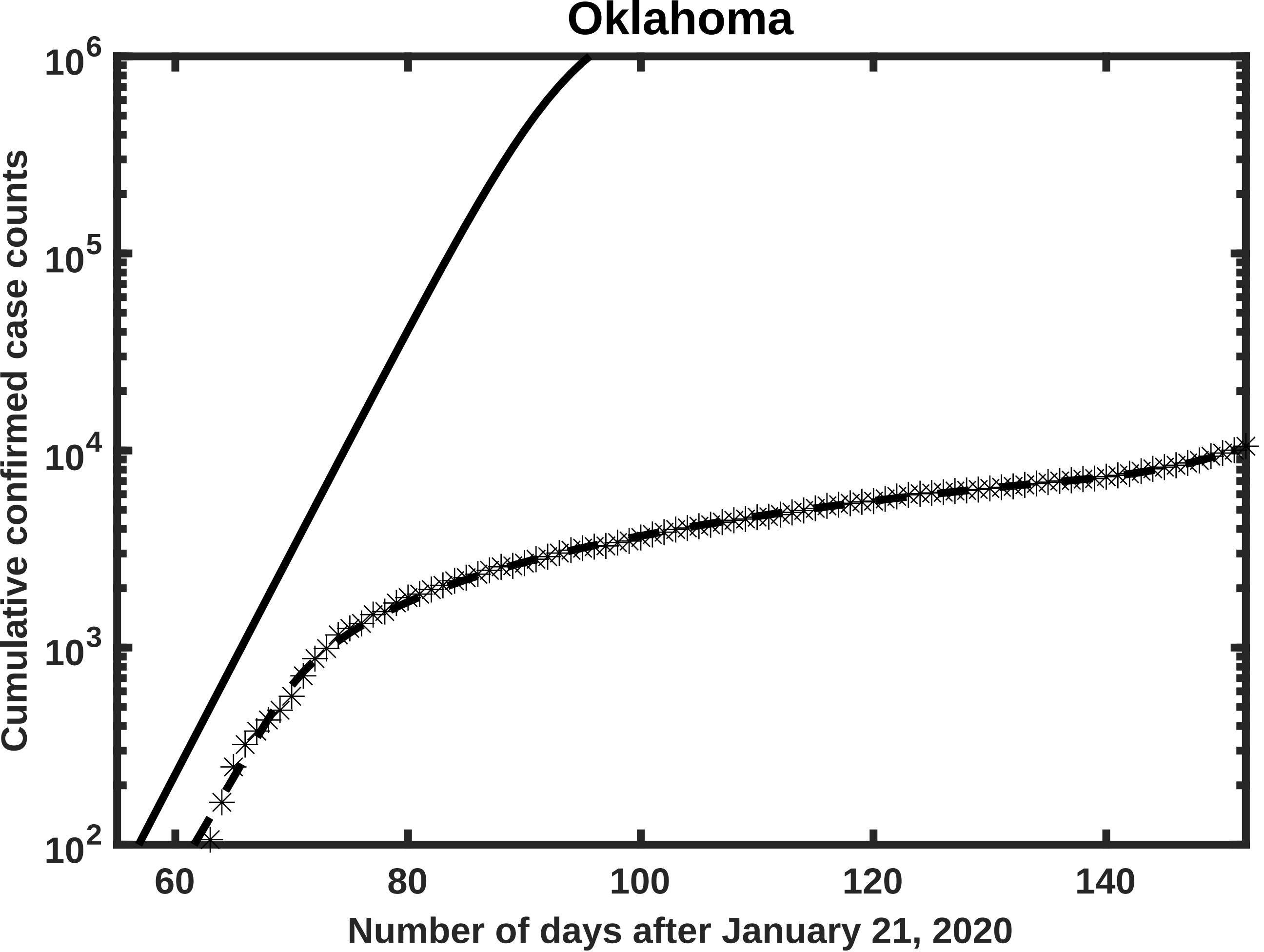
Oklahoma
10^6
10^5
10^4
10^3
10^2
Cumulative confirmed case counts
60
80
100
120
140
Number of days after January 21, 2020

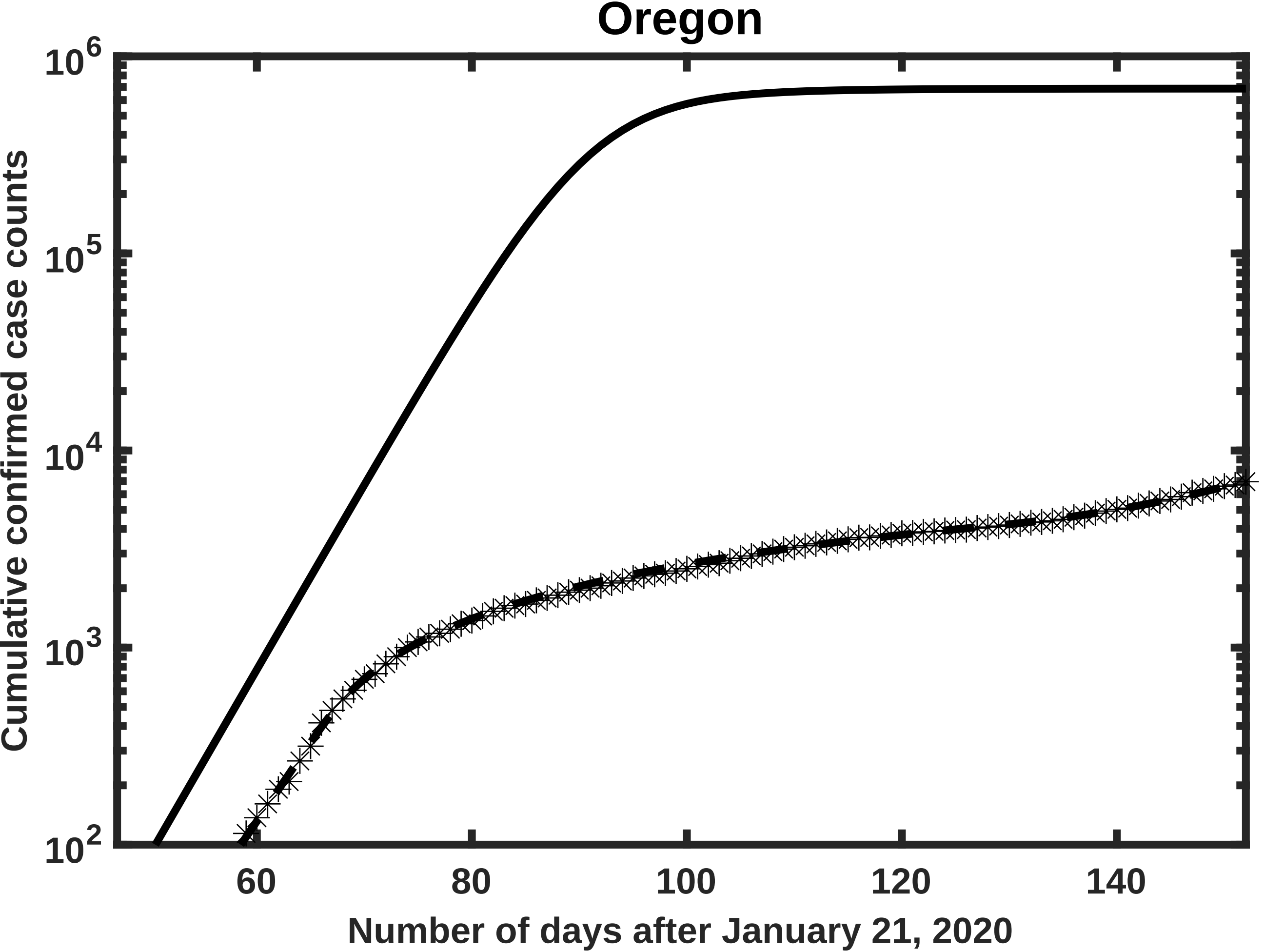
Oregon
Cumulative confirmed case counts
Number of days after January 21, 2020
10^6
10^5
10^4
10^3
10^2
60
80
100
120
140

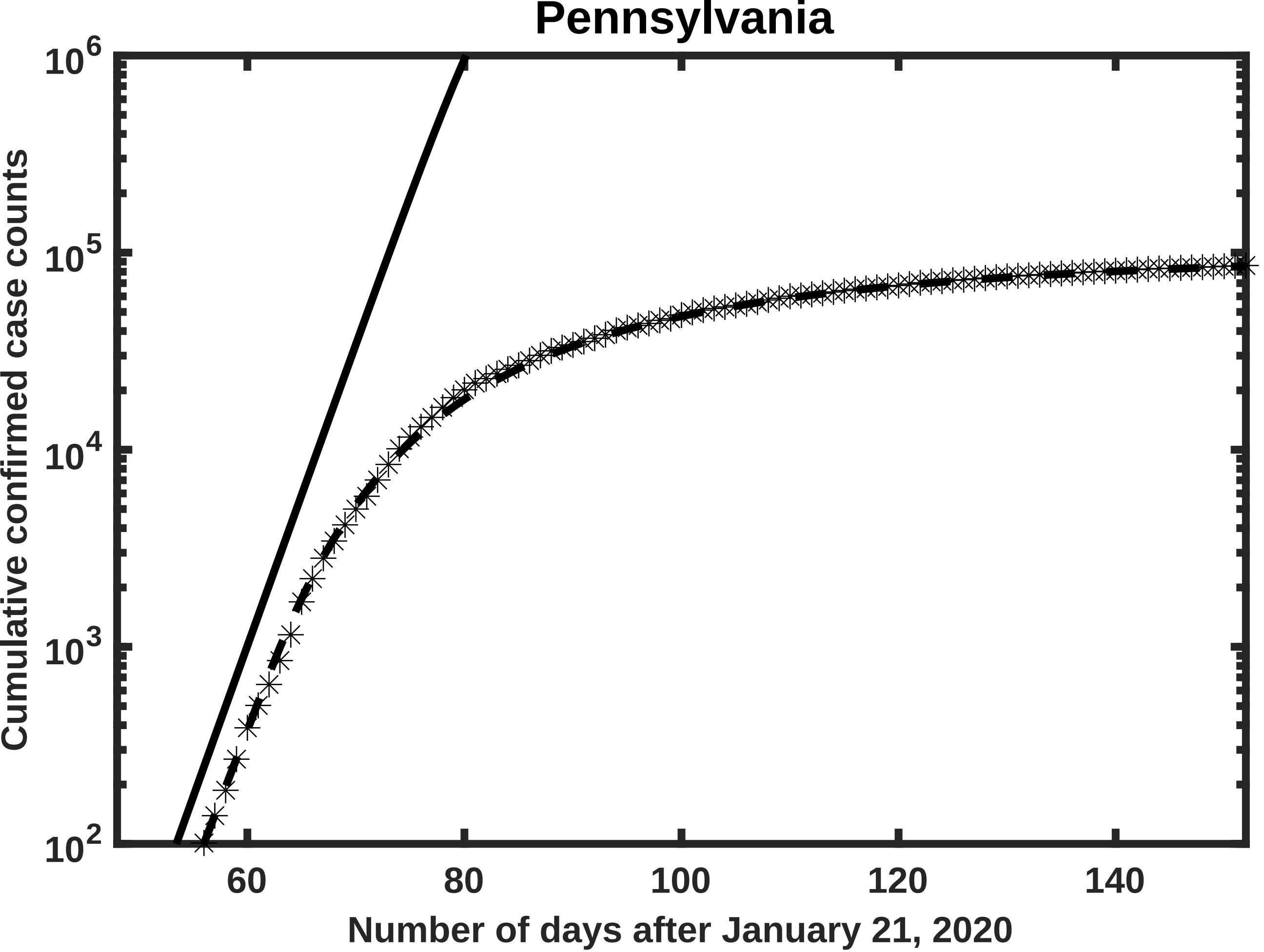

Pennsylvania
10^6
10^5
10^4
10^3
10^2
Cumulative confirmed case counts
60
80
100
120
140
Number of days after January 21, 2020

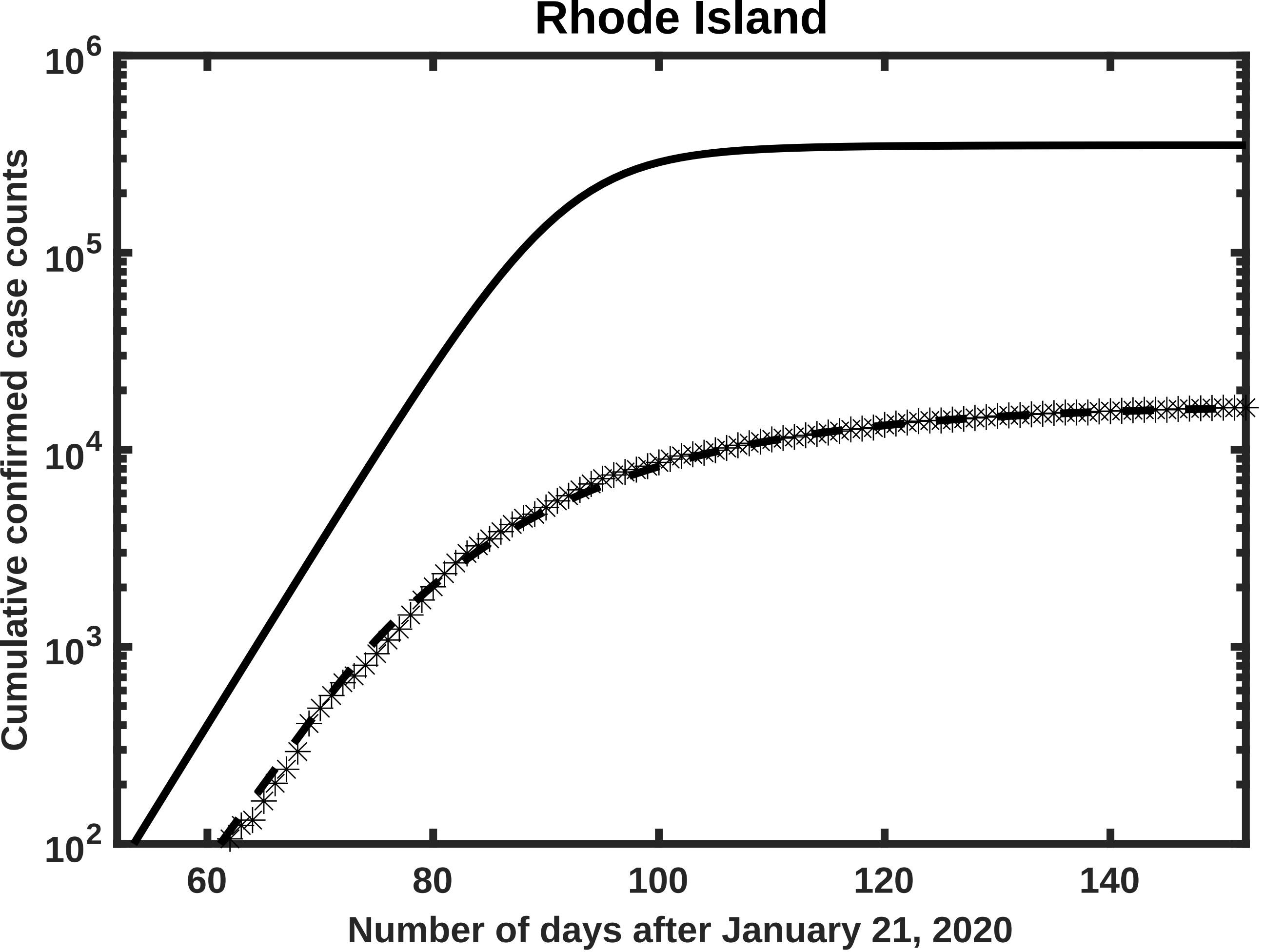
Rhode Island
Cumulative confirmed case counts
10^6
10^5
10^4
10^3
10^2
60
80
100
120
140
Number of days after January 21, 2020

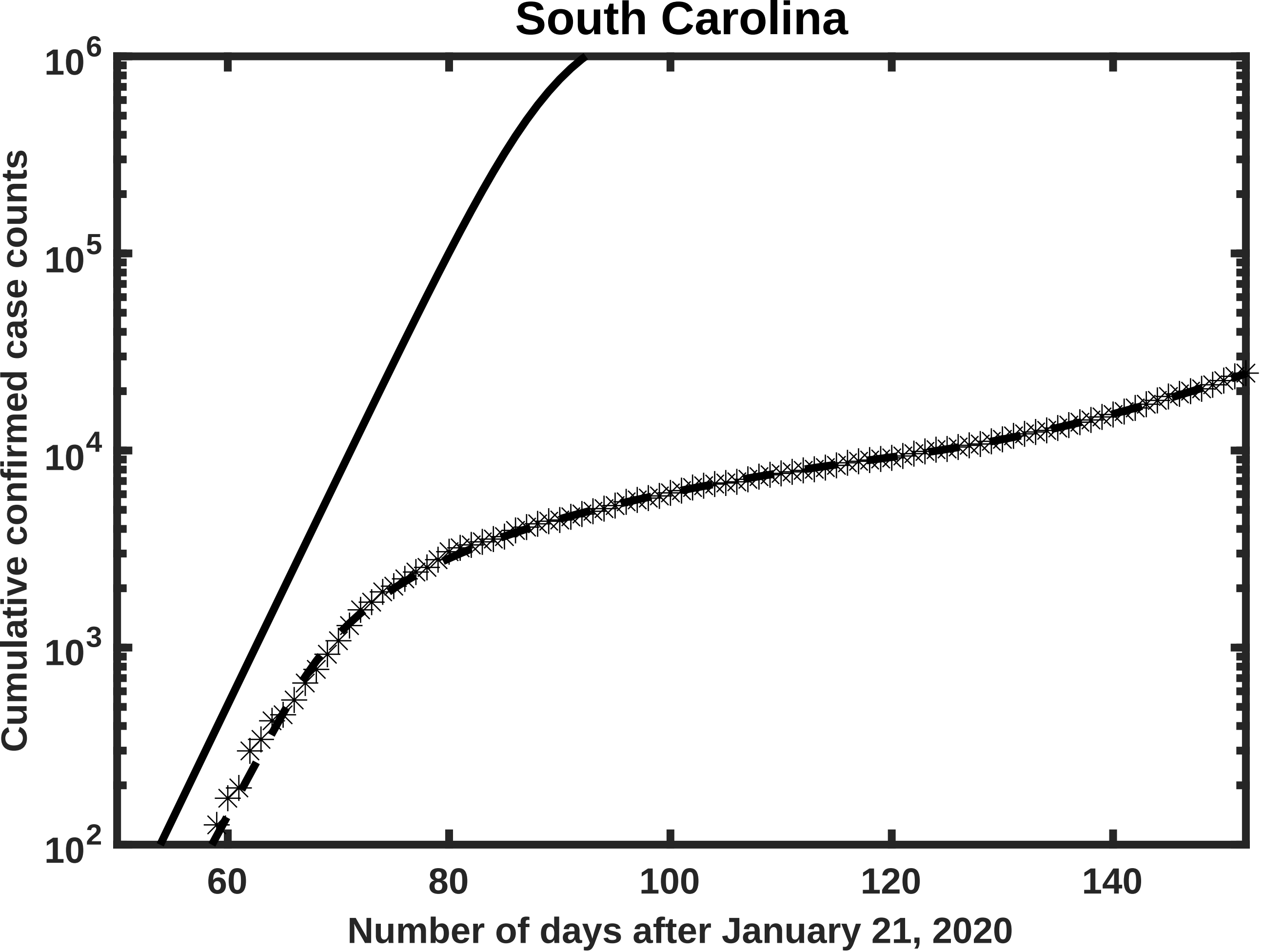

South Carolina
Cumulative confirmed case counts
10^6
10^5
10^4
10^3
10^2
60
80
100
120
140
Number of days after January 21, 2020

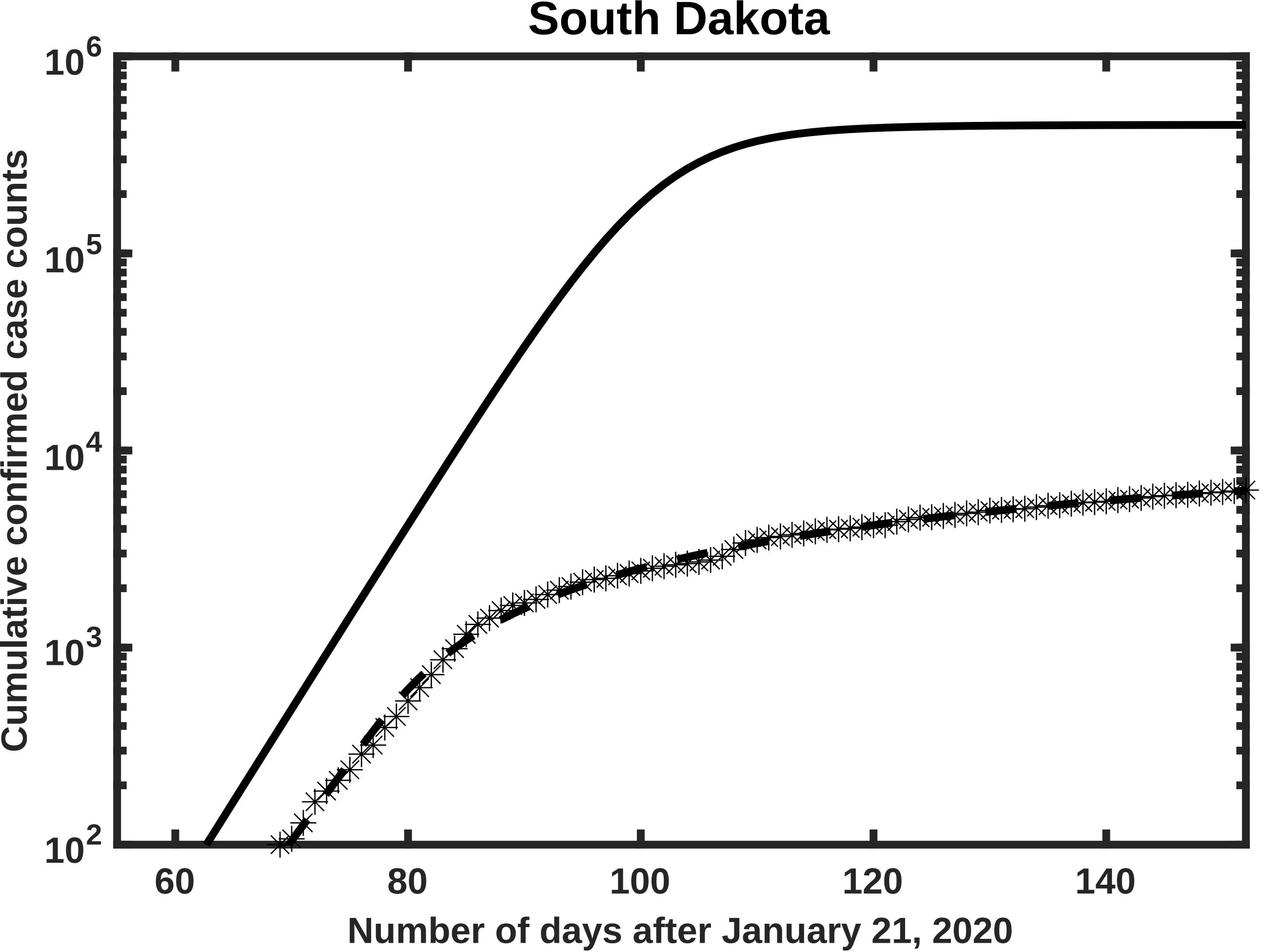

South Dakota
10^6
10^5
10^4
10^3
10^2
60
80
100
120
140
Cumulative confirmed case counts
Number of days after January 21, 2020

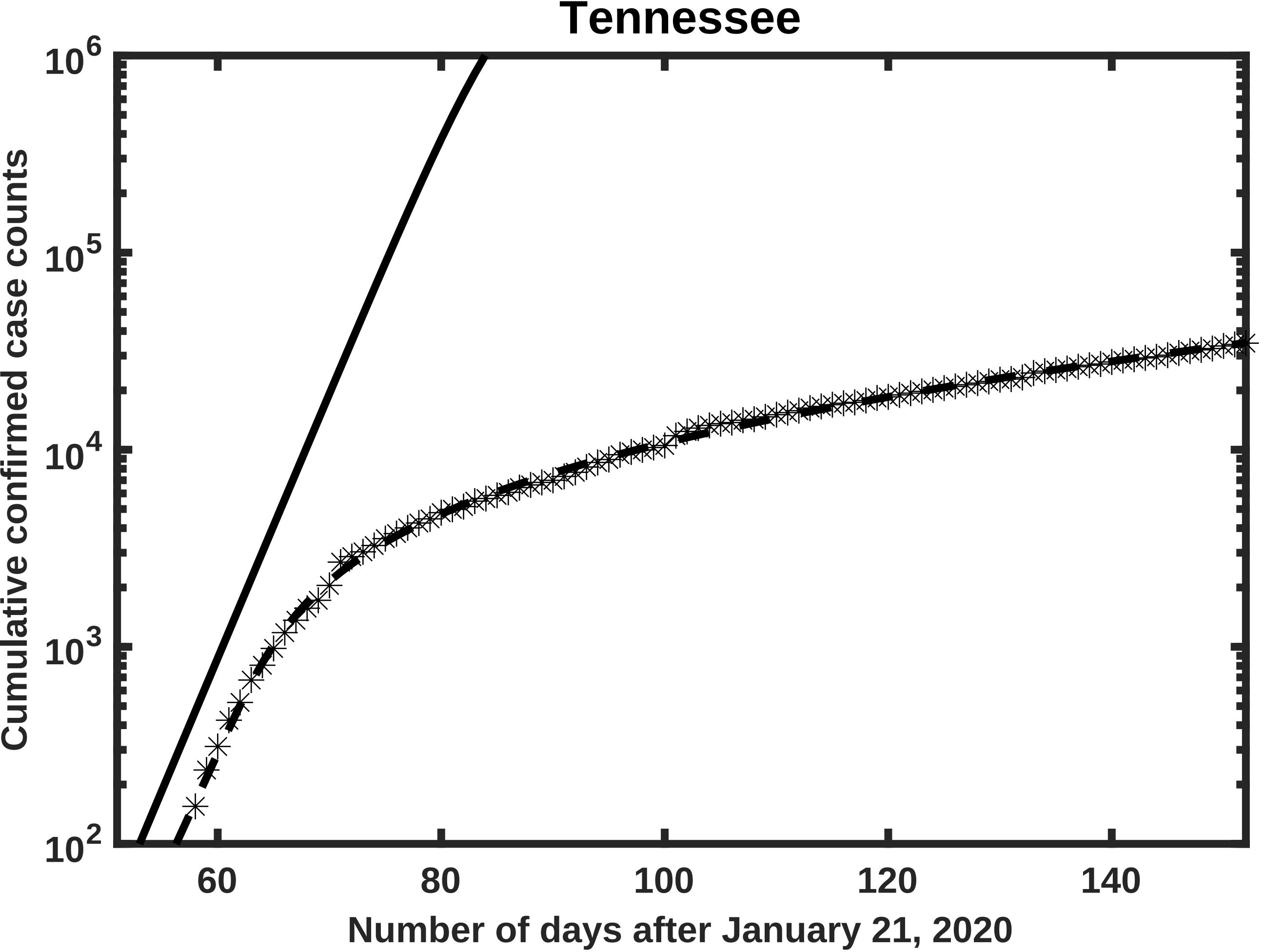

Tennessee
Cumulative confirmed case counts
Number of days after January 21, 2020
10^6
10^5
10^4
10^3
10^2
60
80
100
120
140

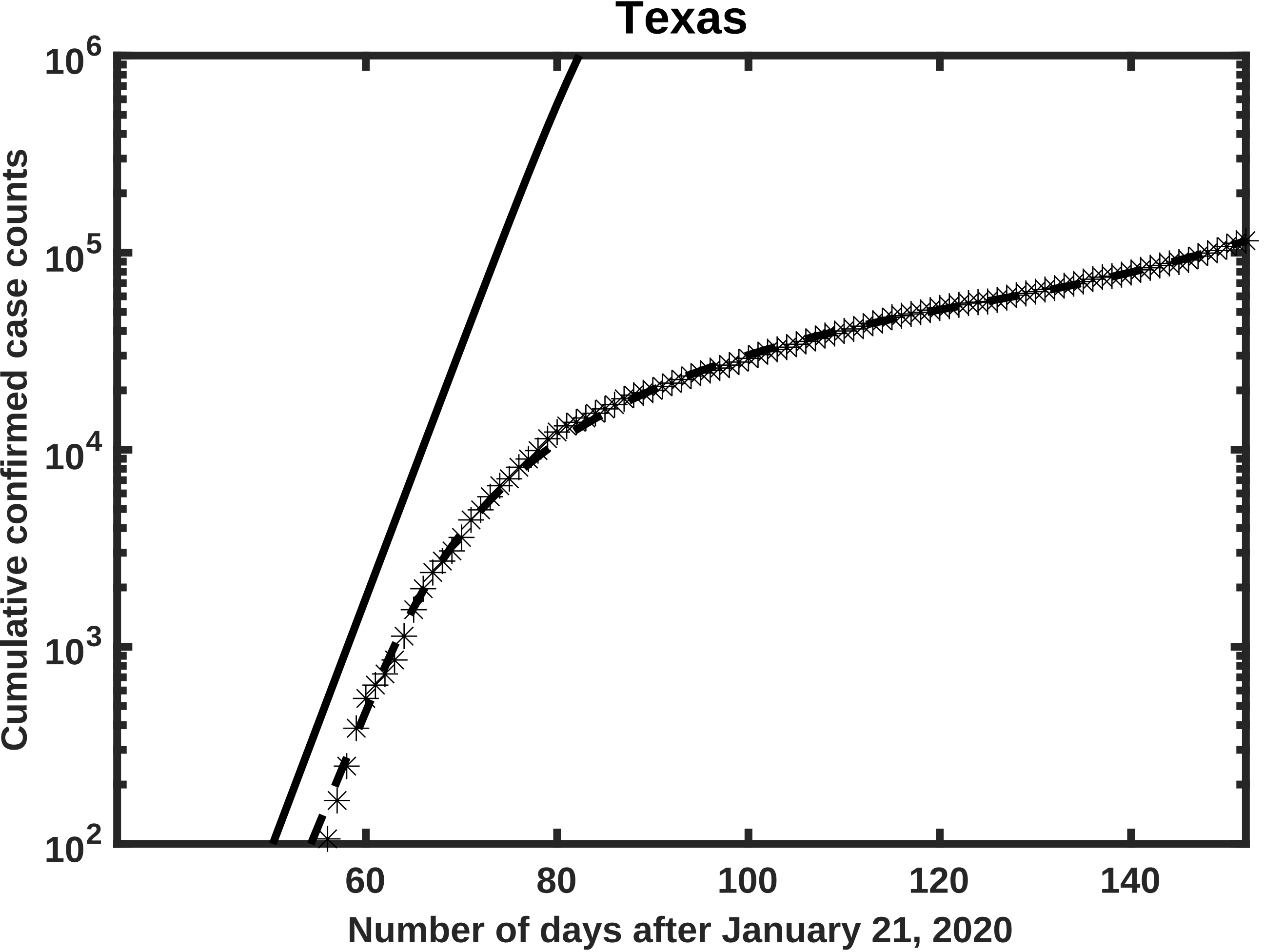
Texas
10^6
10^5
10^4
10^3
10^2
60
80
100
120
140
Cumulative confirmed case counts
Number of days after January 21, 2020

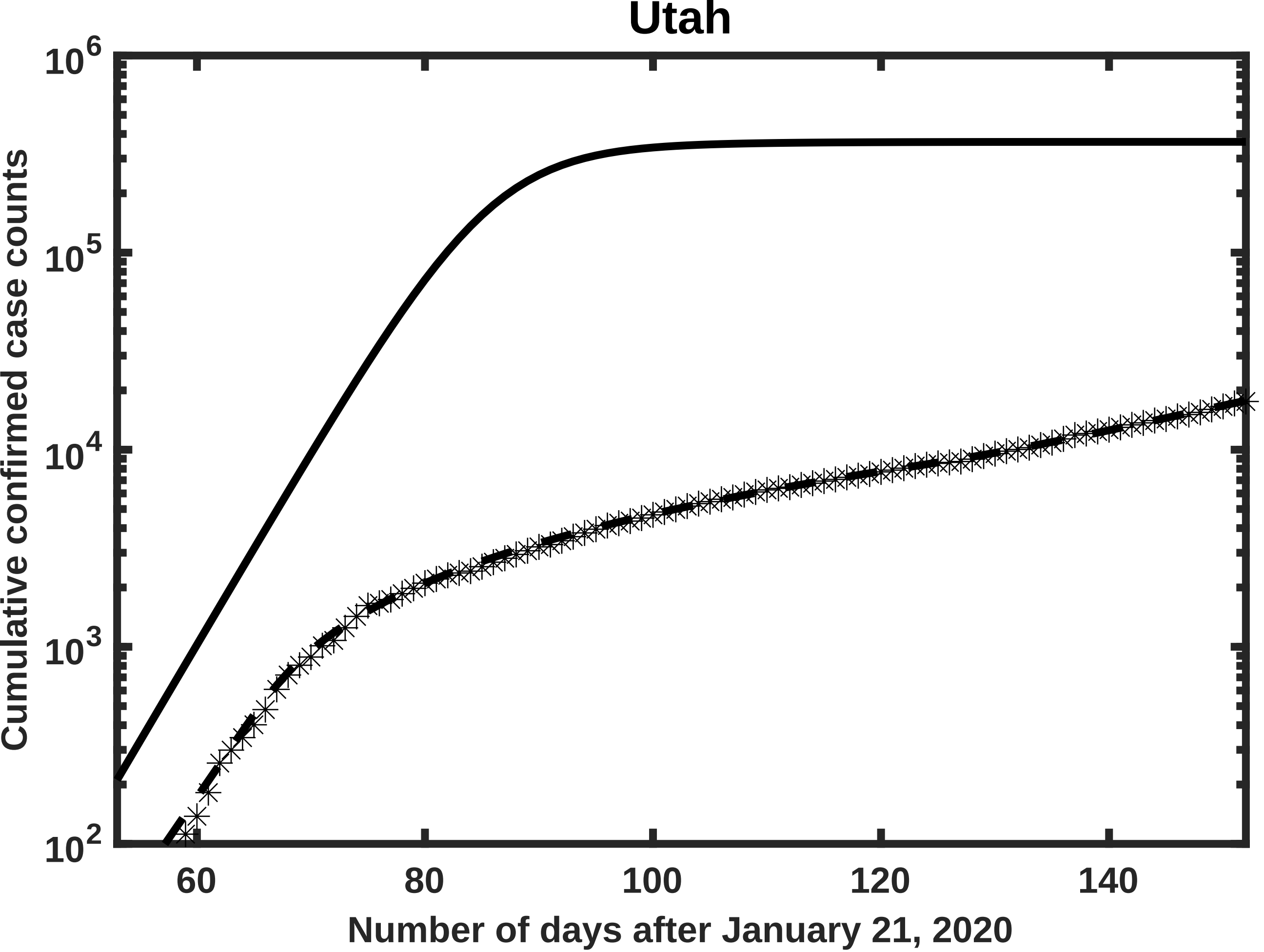
Utah
Cumulative confirmed case counts
10^6
10^5
10^4
10^3
10^2
60
80
100
120
140
Number of days after January 21, 2020

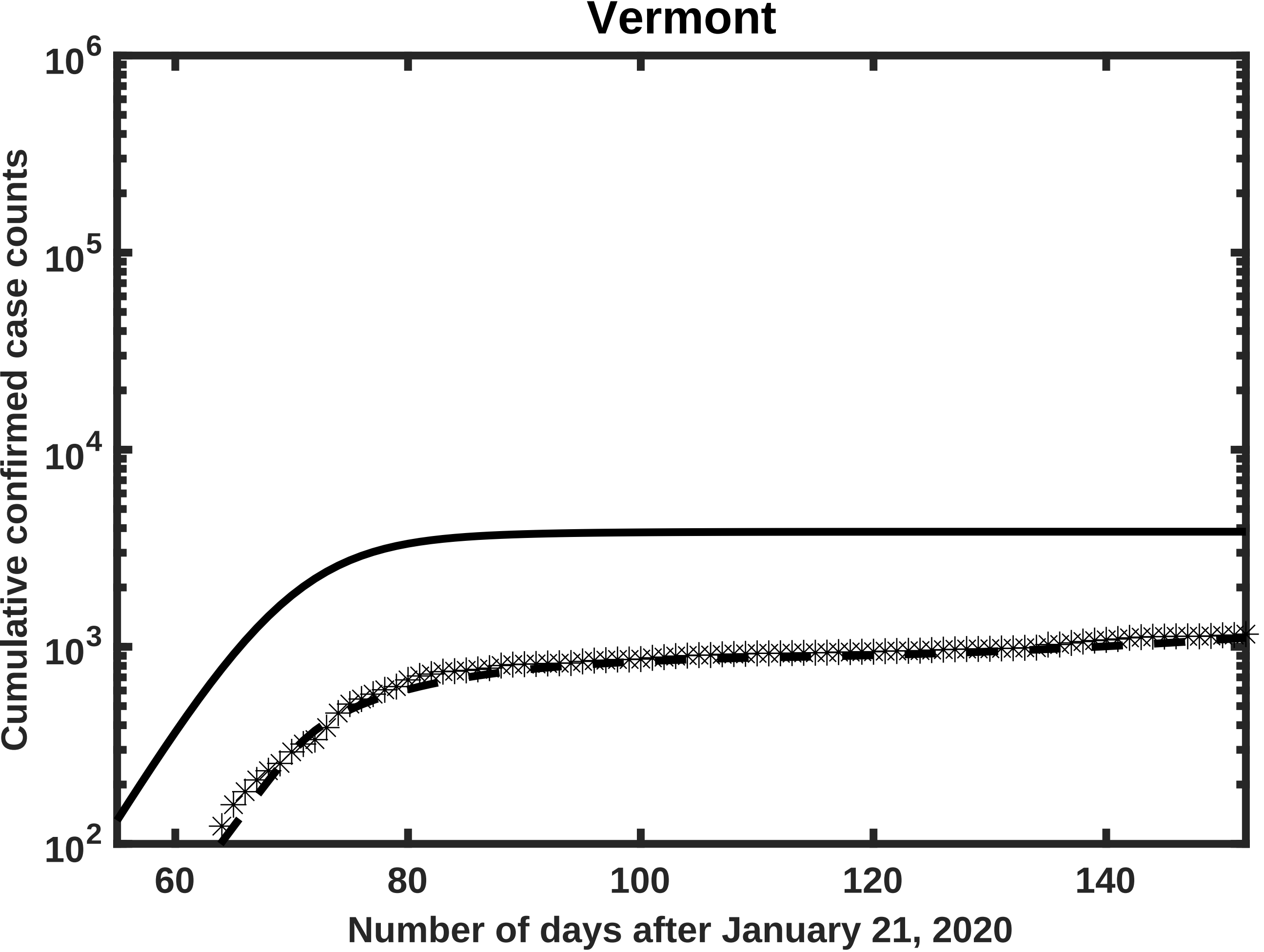
Vermont
10^6
10^5
10^4
10^3
10^2
Cumulative confirmed case counts
60
80
100
120
140
Number of days after January 21, 2020

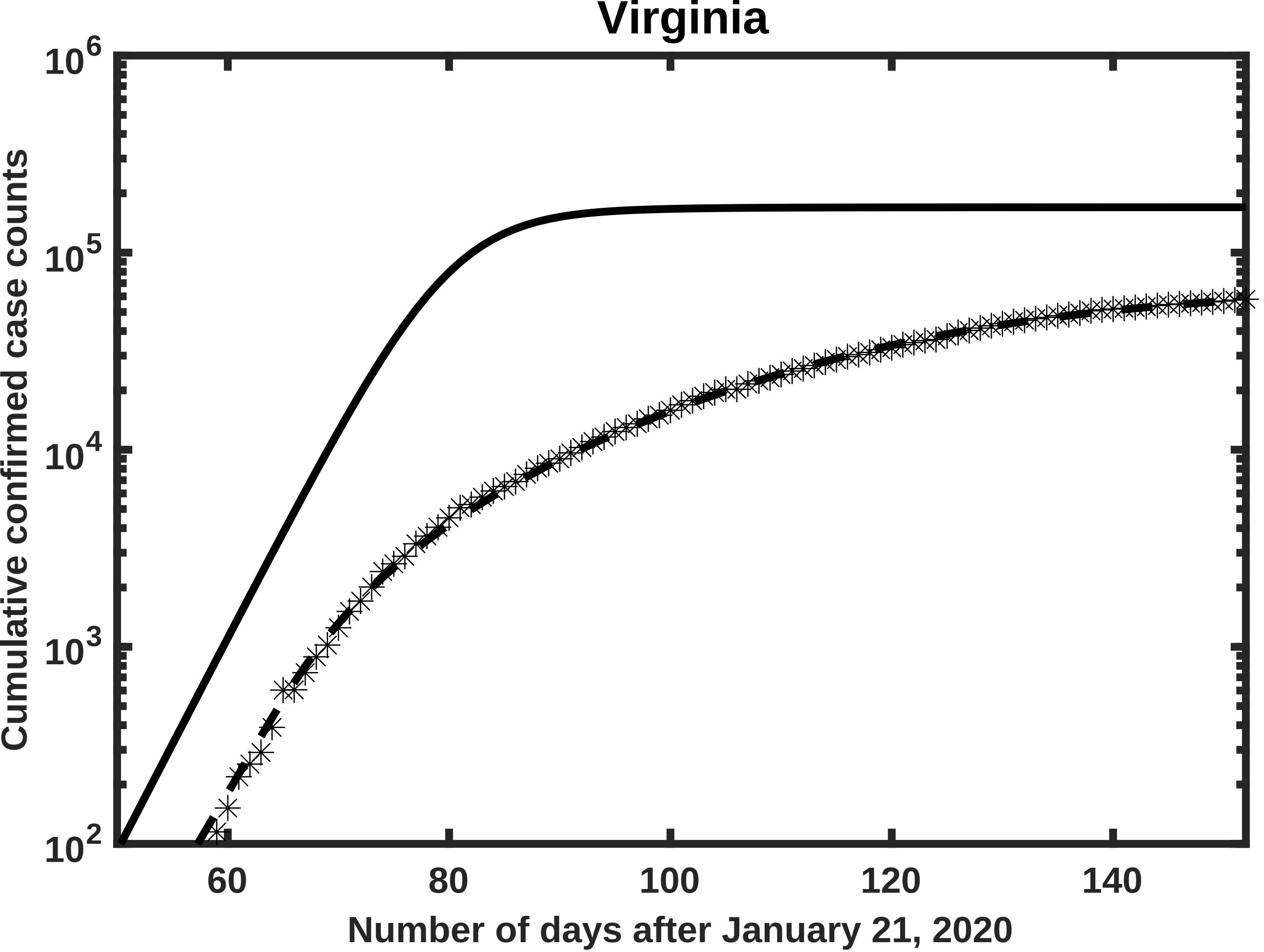
Virginia
Cumulative confirmed case counts
10^6
10^5
10^4
10^3
10^2
60
80
100
120
140
Number of days after January 21, 2020

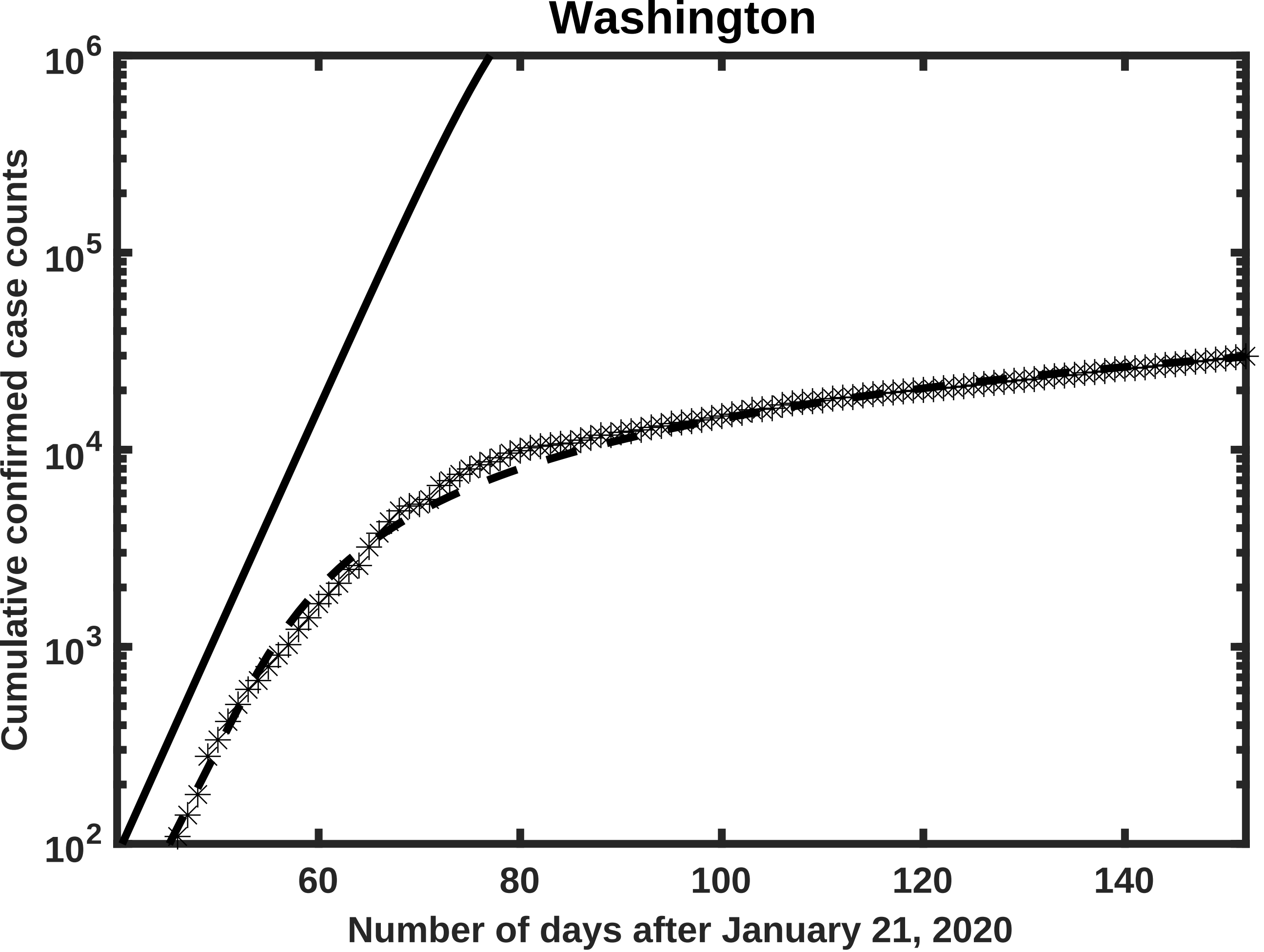
Washington
Cumulative confirmed case counts
10^6
10^5
10^4
10^3
10^2
60
80
100
120
140
Number of days after January 21, 2020

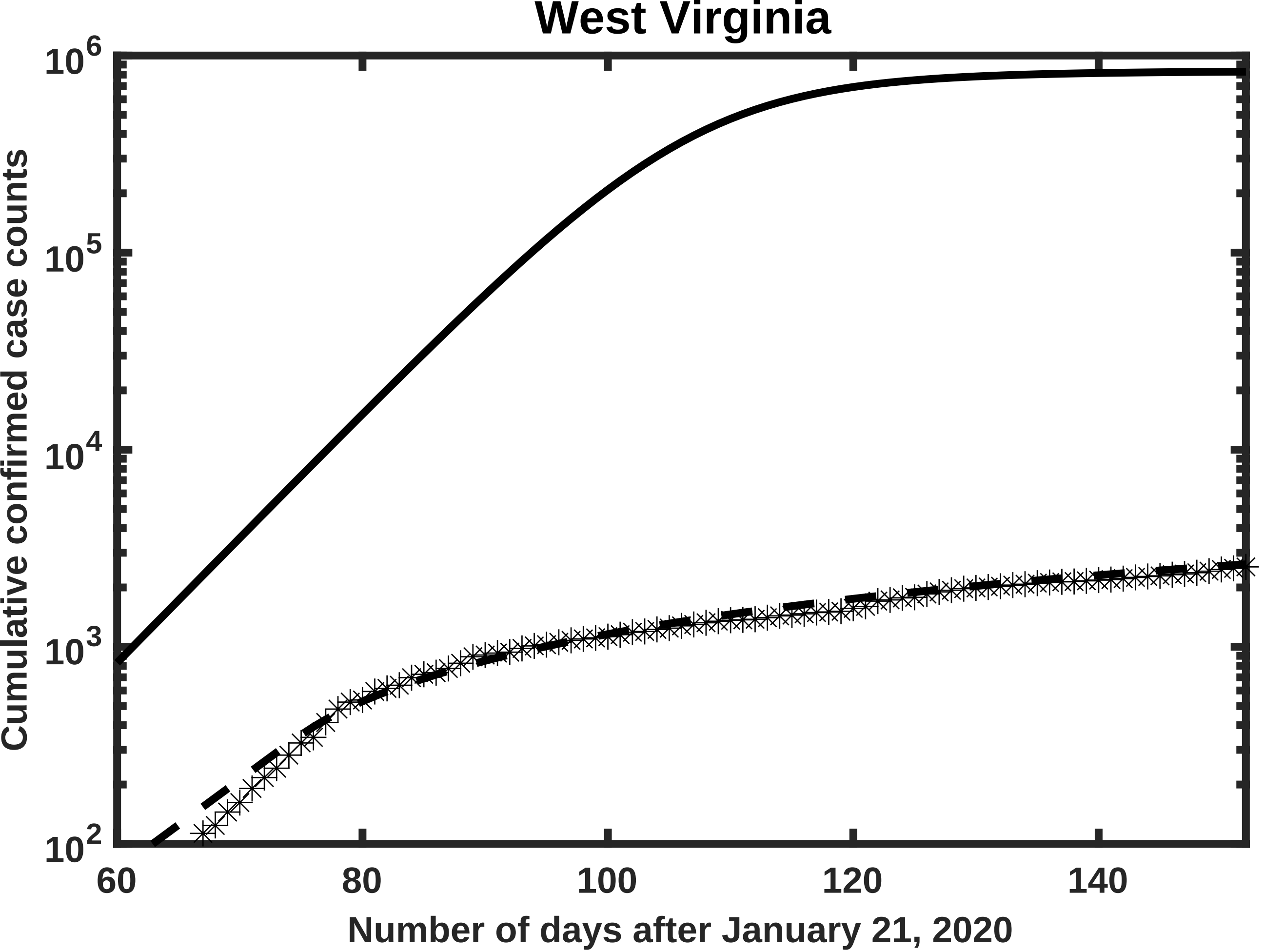
West Virginia
Cumulative confirmed case counts
10^6
10^5
10^4
10^3
10^2
60
80
100
120
140
Number of days after January 21, 2020

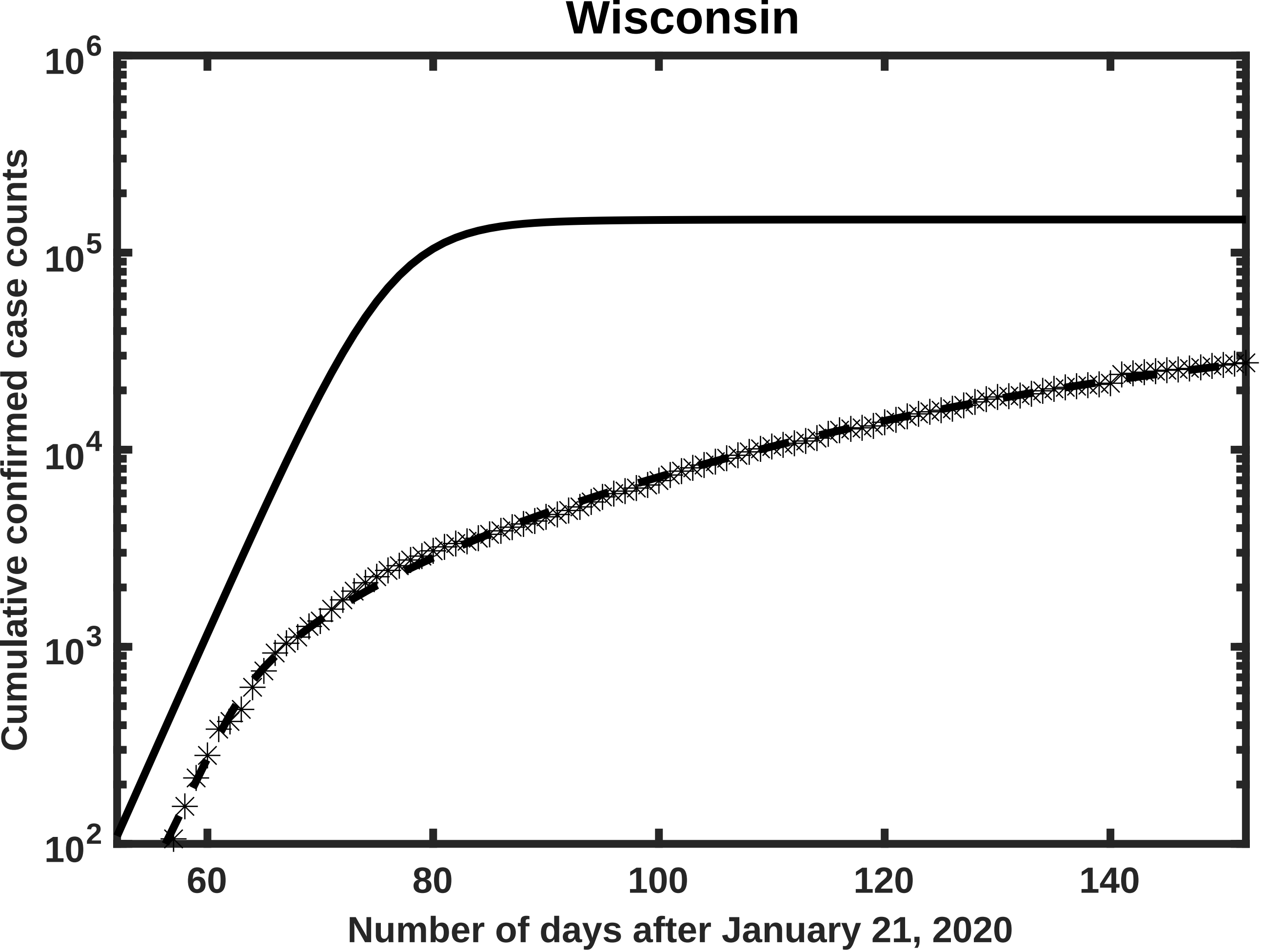
Wisconsin
Cumulative confirmed case counts
10^6
10^5
10^4
10^3
10^2
60
80
100
120
140
Number of days after January 21, 2020
